# Visible light induced alkene aminopyridylation using N-aminopyridinium salts as bifunctional reagents

**DOI:** 10.1038/s41467-019-12216-3

**Published:** 2019-09-11

**Authors:** Yonghoon Moon, Bohyun Park, Inwon Kim, Gyumin Kang, Sanghoon Shin, Dahye Kang, Mu-Hyun Baik, Sungwoo Hong

**Affiliations:** 1 Department of Chemistry, Korea Advanced Institute of Science and Technology (KAIST), Daejeon, 34141 Korea; 2 Center for Catalytic Hydrocarbon Functionalizations, Institute for Basic Science (IBS), Daejeon, 34141 Korea

**Keywords:** Catalytic mechanisms, Synthetic chemistry methodology, Photocatalysis

## Abstract

The development of intermolecular alkene aminopyridylation has great potential for quickly increasing molecular complexity with two valuable groups. Here we report a strategy for the photocatalytic aminopyridylation of alkenes using a variety of N-aminopyridinium salts as both aminating and pyridylating reagents. Using Eosin Y as a photocatalyst, amino and pyridyl groups are simultaneously incorporated into alkenes, affording synthetically useful aminoethyl pyridine derivatives under mild reaction conditions. Remarkably, the C4-regioselectivity in radical trapping with N-aminopyridinium salt can be controlled by electrostatic interaction between the pyridinium nitrogen and sulfonyl group of β-amino radical. This transformation is characterized by a broad substrate scope, good functional group compatibility, and the utility of this transformation was further demonstrated by late-stage functionalization of complex biorelevant molecules. Combining experiments and DFT calculations on the mechanism of the reaction is investigated to propose a complete mechanism and regioselectivity.

## Abstract (web summary)

A key difficulty to overcome in pyridinium salts functionalization is the regioselectivity at the two competing C2 and C4 sites. Here, the authors show bifunctional N-aminopyridinium salts reagents delivering both the aminyl radical and the pyridyl group to an olefin with C4-pyridyl regioselectivity.

## Introduction

**Fig. 1:**
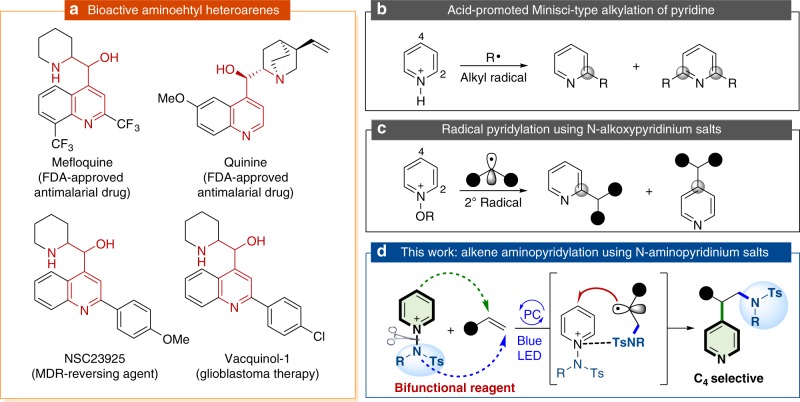
Project background and design plan for photocatalytic aminopyridylation of olefins. **a** Bioactive aminoethyl heteroarenes. **b** Minisci-type alkylation of pyridine core. **c** Radical pyridylation using N-alkoxypyridinium salts. **d** Aminopyridylation of olefins using N-aminopyridinium salts as bifunctional reagents. *PC* photocatalyst. *Ts* toluenesulfonyl

Alkenes are ubiquitous in organic chemistry and the aminative difunctionalization of olefins is a convenient strategy for directly accessing nitrogen-containing molecules^1–11^. However, adding hard Lewis basic amino functionalities to carbon–carbon double bonds that are of course soft Lewis bases on their own right is fundamentally problematic and requires a non-classical strategy because simply using an amino group as a nucleophile is not promising. Recently, photoredox catalysis^12–19^ proved to be highly efficient for generating N-centered radicals^20–28^ that could be employed to drive anti-Markovnikov-selective alkene carbo-, hydroxyl-, and fluoro-amination/amidation reactions and form synthetically and biologically valuable linear amines^29–31^. The pyridine moiety is a basic structural unit of numerous natural products, pharmaceuticals, agrochemicals, and fine chemicals^32–34^. Therefore, a number of synthetic strategies have been explored to install pyridyl groups on molecules^35–44^. Bringing together these two valuable but challenging methodological avenues, we questioned whether aminopyridylation of olefins can be achieved using the power of photoredox catalysis techniques to offer an efficient, direct and environmentally benign synthetic route to these valuable products (Fig. 1a). Moreover, the development of intermolecular alkene difunctionalization with a bifunctional reagent^29,30^ has great potential for quickly increasing molecular complexity in a single operation with high levels of atom and step economy.

The Minisci-type C–H alkylation of pyridines by directly adding alkyl radicals to the C2-position of the pyridine core is well-established, but overalkylation at the C6-position is a common problem resulting in mixtures of mono- and disubstituted products (Fig. 1b)^45–52^. Recently, the N-alkoxypyridinium salts emerged as easily accessible, versatile, and bench-stable pyridine surrogates that in principle may offer increased levels of selectivity and reactivity^40–44^. For example, Herzon reported a cobalt-mediated reductive cross-coupling of alkenes with N-methoxypyridinium salts^53,54^, and we developed remote C–H pyridylation reactions^55^ by using N-alkoxypyridinium salts as substrates (Fig. 1c). Unfortunately, these reactions were found to be modestly regioselective (C2 vs. C4) when secondary alkyl radicals were employed. Clearly, a key difficulty to overcome when developing the proposed alkene aminopyridylation lies in the regioselectivity of the two competing sites (C2 vs. C4) on the pyridinium salts.

The generation of N-centered radicals from N-aminopyridinium salts proved highly efficient in photocatalytic systems^56–59^, although fragmentation of the prefunctionalized N-radical precursor by single electron transfer (SET) reduction results in an inevitable loss of pyridine as chemical waste^56–67^. We envisioned that N-aminopyridinium salts may serve as bifunctional reagents to deliver both the aminyl radical and the pyridyl group to an olefin. In this scheme, the aminyl radicals generated from a fragmentation of the N-aminopyridinium salt react with the alkene to form a new C–N bond and an adjacent β-amino radical. The resultant alkyl radical may then trap N-aminopyridinium salt to install a pyridyl group, affording synthetically and biologically valuable aminoethyl pyridines (Fig. 1d)^68,69^. Herein, we report an efficient aminopyridylation of alkenes using a variety of N-aminopyridinium salts as bifunctional reagents for simultaneous amination and pyridylation under mild reaction conditions. Moreover, this catalytic platform does not require any oxidant and allows excellent C4 regiocontrol over radical trapping with N-aminopyridinium salts.

## Results

### Reaction discovery and optimization

We tested the viability of our proposed approach using vinyl ether **1a** and N-aminopyridinium salt **2a** as model reactants under blue LED irradiation, and selected data are enumerated in Table 1. After evaluating different reaction conditions, we were pleased to find that the use of Ir(ppy)_3_ in acetonitrile initiated 1,2-aminopyridylation at room temperature to afford the desired product **3a** in 30% yield (entry 1), indicating that the proposed overall process involving tandem amination and pyridylation is possible. Notably, radical trapping with **2a** occurs selectively at the C4 position of the pyridine core. Significant turnover was observed with the Ru(phen)_3_Cl_2_ catalyst, and product **3a** was formed in 75% yield (entry 3). From the viewpoint of green chemistry, organic photocatalysts attracted much attention recently, and promising results were obtained with Eosin Y (EY) and **Q**_**1**_^41,43,55^ (entries 7 and 8). The choice of solvent was critical for the reaction efficiency, and DMSO gave the best performance. Among the screened bases, K_3_PO_4_ was found to be most effective. We examined the influence of the light source and found that blue LEDs led to the highest yield. Next, we examined the influence of the amount of EY and found that catalyst loading could be reduced to as low as 0.5 mol% with a slightly increased product yield (entry 9, C4/C2 = 13:1). As a result, the reaction conditions described in entry 9 were considered optimal. Control experiments verified the essential role of light and the photocatalyst in this aminopyridylation reaction (see Supplementary Fig. 1). As expected, when the reaction was conducted in the presence of 2,2,6,6-tetramethyl-piperidine-1-oxyl (TEMPO), no desired product was observed (entry 11), supporting a radical mechanism being operative in this reaction. In the absence of the base, product **3a** was obtained in 75% yield, which shows that the use of an external base is not required, and product pyridine may function as a base during the reaction (entry 12).

**Table 1:** Optimization of Reaction Conditions^a^


entry	photocatalyst	base	solvent	yield[%]^b^
1	Ir(ppy)_3_ (2 mol%)	K_3_PO_4_	MeCN	30
2	Ir(dFCF_3_ppy)_2_(bpy)PF_6_ (2 mol%)	K_3_PO_4_	MeCN	64
3	Ru(phen)_3_Cl_2_ ∙ xH_2_O (2 mol%)	K_3_PO_4_	MeCN	75
4	Eosin Y (2 mol%)	K_3_PO_4_	MeCN	57
5	Eosin Y (2 mol%)	K_2_HPO_4_	MeCN	46
6	Eosin Y (2 mol%)	Na_2_CO_3_	MeCN	52
7	Eosin Y (2 mol%)	K_3_PO_4_	DMSO	79
8	**Q**_**1**_ (2 mol%)	K_3_PO_4_	DMSO	79
9	Eosin Y (0.5 mol%)	K_3_PO_4_	DMSO	86
10	**Q**_**1**_ (0.5 mol%)	K_3_PO_4_	DMSO	76
11^c^	Eosin Y (0.5 mol%)	K_3_PO_4_	DMSO	0
12	Eosin Y (0.5 mol%)	−	DMSO	75

^a^Reaction conditions: **1a** (0.05 mmol), **2a** (0.075 mmol), and base (0.06 mmol) in solvent (0.5 mL) under irradiation using blue LEDs at rt for 3 h under N_2_. ^b^Yields were determined by ^1^H NMR spectroscopy. ^c^TEMPO (1.0 equiv) was added. **Q**_**1**_ 3-(diphenylphosphoryl)-6-methoxy-1-methylquinolin-2(1 H)-one, DMSO dimethyl sulfoxide.

### Mechanistic studies

Whereas these initial findings are encouraging, they raise several fundamental questions. In previous studies with N-alkoxypyridinium salts, tertiary alkyl radicals predominantly afforded C4-products while secondary alkyl radicals gave rise to a modest preference for the C2-product^40,41,53–55^. Given the relatively modest steric demand at the olefinic carbon, the high selectivity for the C4-product is curious. Mechanistically, several reaction steps including radical formation, N–N bond cleavage, amination, and pyridylation must be performed, but the sequence of these reaction steps is unclear. To conceptually understand the catalytic mechanism and identify the features governing the regiocontrol in this reaction, we carried out quantum chemical calculations based on density functional theory (DFT).

**Fig. 2:**
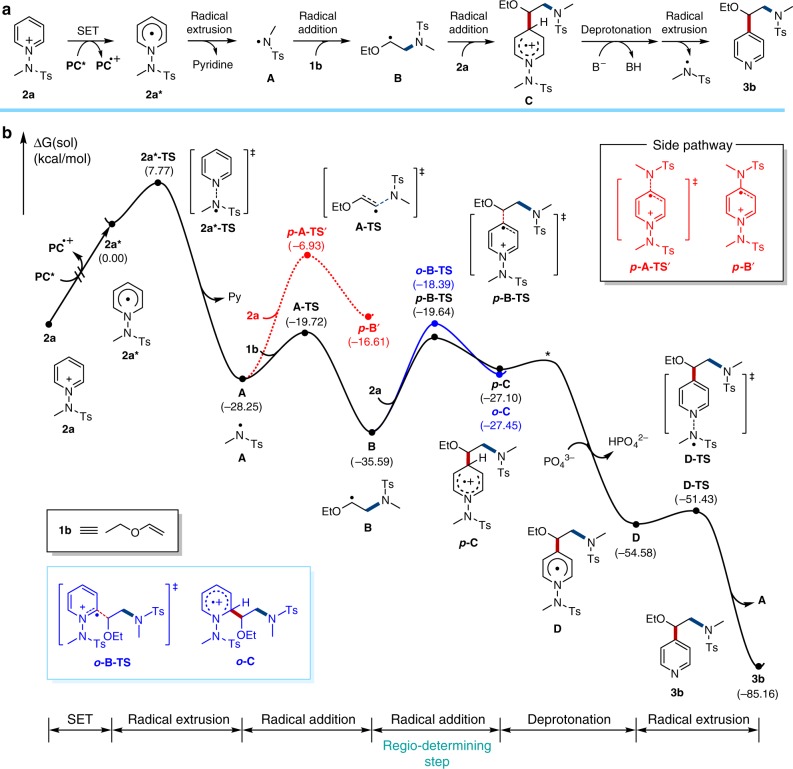
Propose reaction pathway and related free energy profile. **a** Proposed reaction pathway. **b** Free energy profile for the formation of **3b** that is described with a solid black line. Blue and red traces represent the side pathway toward *ortho*(C2)-product generation and direct N radical attack on **2a**, respectively. Energy profile for *ortho*(C2)-product is only shown in a radical addition step as it has a very similar energy trend with that of ***p*-B**’ after radical addition and does not affect the regioselectivity

In these calculations, the prototype reactant **2a**, N-(methyltosyl)aminopyridinium, is employed to form the product **3b**. As illustrated in Fig. 2, the proposed mechanism is fundamentally different from the previously studied alkylation reactions. The catalytic cycle is initiated by the excitation of the photocatalyst **PC** to PC*, which subsequently transfers a single electron to **2a** to form the unstable N-pyridine radical 2a*. Homolytic cleavage of the N–N bond liberates pyridine and produces the activated N-centered radical **A**. This reaction is calculated to have a step barrier of 7.8 kcal/mol and is irreversible with a driving force of 28.3 kcal/mol. From the N-centered radical **A**, we considered two plausible reaction pathways invoking the attack of either the alkene **1b** or a second equivalent of the pyridinium **2a**. Interestingly, attacking **2a** is both kinetically and thermodynamically disfavored and requires a step barrier of 21.3 kcal/mol associated with the transition state ***p*-A-TS’** to afford the putative intermediate ***p*-B’**, found to be 11.6 kcal/mol higher in energy than **A**, as shown in red dotted line in Fig. 2. Our calculations indicate that the N-centered radical **A** prefers to attack the olefin substrate **1b** to give an intermediate **B** with a barrier of only 8.5 kcal/mol and this step is energetically downhill by 7.3 kcal/mol. It is interesting that the N-centered radical **A** acts as an electrophile to attack the electron-rich olefin substrate in this instance, rather than behaving like a nucleophile that may have preferred to react with the electron-poor pyridinium substrate. Although it is easy to recognize electrophilic and nucleophilic reactants in closed-shell molecules, it is rather difficult to predict if radical species prefer to engage an electron-rich or an electron-poor substrate. Whereas DFT calculations can capture these effects, as we demonstrate, it is desirable to understand these results in a more intuitive sense.

**Fig. 3:**
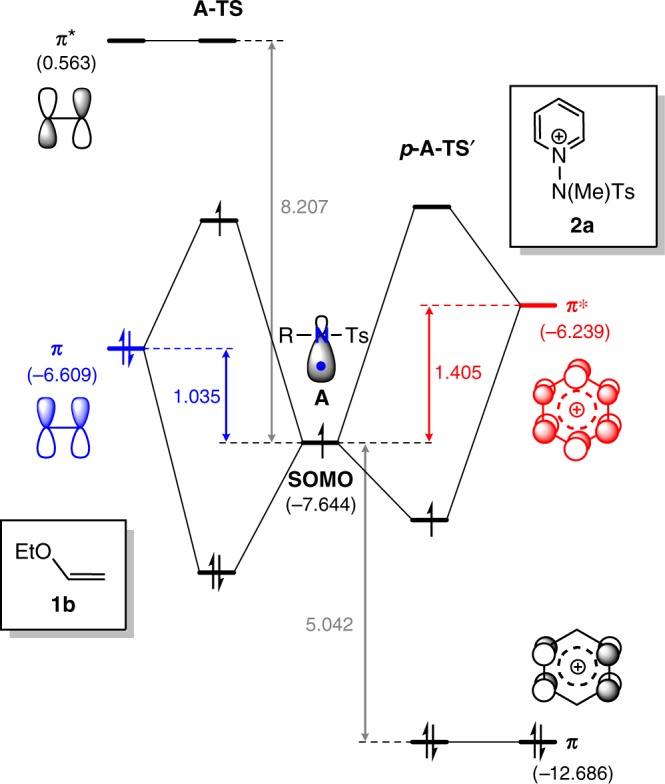
MO diagram. Qualitative MO diagram of N-centered radical interaction with the enolether **1b** or the pyridinium salt **2a**. Energies are given in eV

The frontier molecular orbitals of **A**, **1b**, and **2a** displayed in Fig. 3 illustrate that the singly occupied molecular orbital (SOMO) of A is at –7.64 eV, whereas the highest π and the lowest π* MOs of **1b** are found at −6.61 and +0.56 eV, respectively. The analogous orbitals of **2a** are located at –12.69 and –6.24 eV, respectively. Because the SOMO of **A** is closest in energy to the occupied π orbital of the olefin substrate **1b**, as highlighted in blue in Fig. 3, these two MOs engage in a 2-orbital-3-electron interaction. As a consequence, the N-centered radical acts as an electrophile towards the olefin. The orbitals of the pyridinium substrate **2a**, on the other hand, are much lower in energy because of the positive molecular charge and the closest frontier orbital to the SOMO of A is the π* orbital, as highlighted in red in Fig. 3. Consequently, these two MOs give a 2-orbital-1-electron interaction and the radical acts as a nucleophile towards the pyridinium substrate. The strengths of these interactions are governed by the overlap of the interacting orbitals and their energy difference. Large orbital overlap and small energy difference will increase the interaction strength. As illustrated in Fig. 3, the energy difference of the interacting frontier orbital is 1.04 eV for the olefin **1b**, whereas 1.41 eV is found for the pyridinium substrate. Moreover, the π orbital of the olefin is much more localized than the π* orbital of the pyridinium, thus the orbital overlap will give notable preference to the olefin. Thus, both components favor the radical-olefin interaction, which explains the transition state and intermediate energies presented above.

To push the reaction forward, it is necessary that the alkyl radical **B** attacks a pyridinium species to form an unstable cationic radical intermediate **C** that can be deprotonated to afford the neutral radical species **D**. Note that the deprotonation transition state was not specifically modeled, but we assume this step to be fast and unproblematic considering the small kinetic isotope effect (Fig. 8a, *K*_*H*_*/K*_*D*_ = 1.2). The final product is formed by N–N bond cleavage to extrude the aminyl radical, which can start a new catalytic cycle. Our calculations indicate that the radical addition step **B** → **C** is likely rate-determining and is also responsible for the regiocontrol. Although free energies of two regioisomers ***p*-C** and ***o*-C** are almost identical at –27.1 and –27.4 kcal/mol, respectively, the transition states ***p*-B-TS** leading to the C4-intermediate ***p*-C** is 1.3 kcal/mol lower in energy than the C2-alternative, in good agreement with the experimentally observed regioselectivity.

**Fig. 4:**
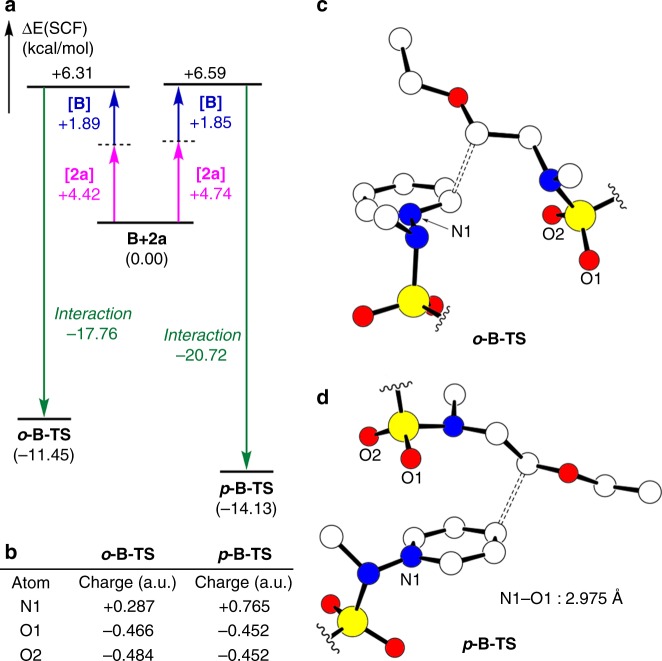
Distortion-interaction analysis. **a** Distortion-interaction analysis of ***o*-B-TS** and ***p*-B-TS**. [**2a**] and [**B**] indicate the structural distortion energy of radical and pyridinium fragments from the corresponding intermediates **2a** and **B** to each transition state geometries. **b** Atomic charges from the electrostatic potential of ***o*-B-TS** and ***p*-B-TS**. Charges are shown in the atomic unit (a.u.). **c** Optimized structure of ***o*-B-TS**. **d** ***p*-B-TS**. *p*-tolyl groups and hydrogen atoms are omitted for clarity

To understand the origin of the regioselectivity, a distortion-interaction analysis was conducted, as shown in Fig. 4a, where the pyridinium and the alkyl fragments are calculated at the geometry found in the transition state, but separated from each other. By examining their energies, we can estimate the energy required to distort these molecular components to the structure needed at the transition state and also calculate the interaction energy between these distorted molecular fragments. Note that we use electronic energies for this purpose and the transition state ***p*-B-TS** is found to be 2.7 kcal/mol lower in energy than ***o*-B-TS**, which is slightly more pronounced than 1.3 kcal/mol estimated for the solution phase free energy. Interestingly, the amount of structural distortions that the two reactants must undergo to reach the transition state structures are nearly identical at 6.3 and 6.6 kcal/mol. The interaction energies were found to be –17.8 and –20.7 kcal/mol for the ***o*-B-TS** and ***p*-B-TS**, respectively. Curiously, the lengths of the C–C bonds that are being formed are practically identical at 2.23 and 2.25 Å, which is inconsistent with the interaction energy difference of 2.9 kcal/mol. Further bonding analysis presented in the Supplementary information reveals that the interaction energy difference stems from an additional non-covalent attraction between the partially negatively charged sulfonyl oxygens and the positively polarized nitrogens of the pyridinium substrate across a distance of 2.98 Å, as illustrated in Fig. 4. This interaction is structurally not possible in ***o*-B-TS**.

### Substrate scope studies

**Fig. 5:**
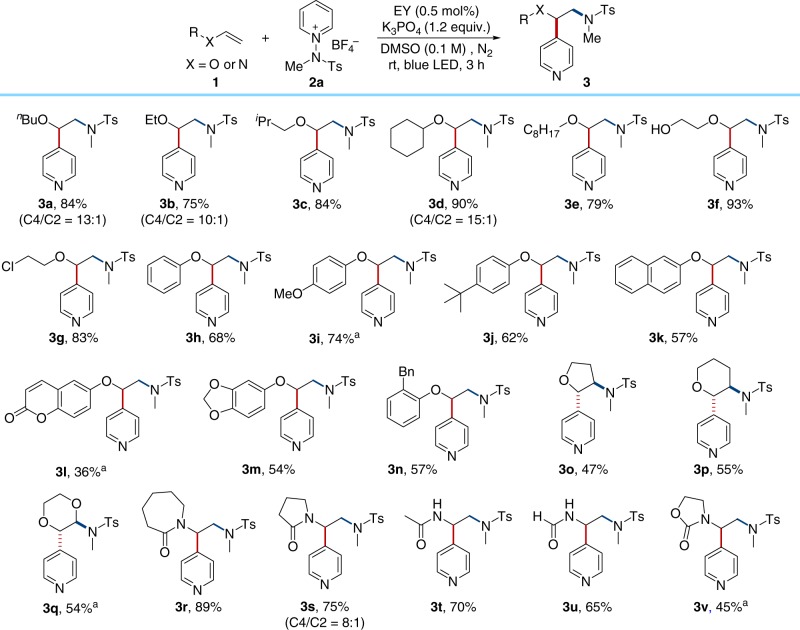
Substrate scope of alkenes. Reaction conditions: **1** (0.1 mmol), **2a** (0.15 mmol), EY (0.5 mol%), K_3_PO_4_ (0.12 mmol), and DMSO (1.0 mL) using blue LEDs at rt for 3 h under N_2_. Yields of isolated products. C4/C2 ratio was measured by ^1^H NMR spectroscopy. Unless indicated, C4/C2 > 15:1. ^a^ Ru(phen)_3_Cl_2_ ⋅ xH_2_O (1.0 mol%) was used in MeCN

Encouraged by the aforementioned experimental findings and with a solid understanding of the mechanism based on our DFT calculations in hand, we investigated the substrate scope to test the utility and versatility of the current photocatalytic tandem reaction. First, the generality with respect to alkene substrates was investigated, as illustrated in Fig. 5. Various electron-rich alkenes, such as vinyl ether and enamides reacted readily, leading to the formation of β-aminopyridine derivatives with excellent C4-regiocontrol. Aliphatic vinyl ethers with various chain lengths and sizes reacted readily (**3a**–**3e**). Substrates bearing polar functionalities, such as hydroxyl and chloro groups were also well-tolerated to provide the desired products in good yield (**3f** and **3g** ). We subsequently assessed the applicability of this transformation with respect to the vinyl substrates bearing aryl ethers (**3h**–**3n**). For example, the substrates containing naphthyl or coumarin groups could be employed in this transformation to afford the desired products **3k** and **3l**, although the latter was generated with lower yields. The current protocol can be extended to the functionalization of internal olefins, providing the desired difunctionalized products in moderate yield (**3o**–**3q**). It is also noteworthy that the reaction can also be extended to vinyl amides, formamide, and carbamate to yield the desired products (**3r**–**3v**). Low reactivity was observed with unactivated alkenes under the standard reaction conditions (see Supplementary Fig. 1). To demonstrate the practical applicability of this method, we subsequently performed the reaction with alkene **1a** and **2a**, on the 2.0 mmol scale, and the desired product **3a** was formed in 83% yield.

**Fig. 6:**
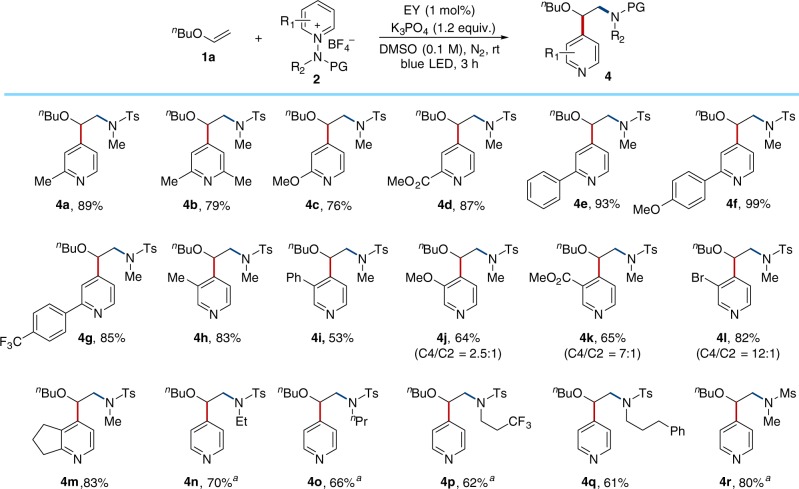
Substrate scope of bifunctional reagents. Reaction conditions: **1a** (0.1 mmol), **2** (0.15 mmol), EY (1 mol%), K_3_PO_4_ (0.12 mmol), and DMSO (1.0 mL) using blue LEDs at rt for 3 h under N_2_. Yields of isolated products. C4/C2 ratio was measured by ^1^H NMR spectroscopy. Unless indicated, C4/C2 > 15:1. ^a^ Ru(phen)_3_Cl_2_ ⋅ xH_2_O (1.0 mol%) was used in MeCN

To further test the generality of this reaction, we investigated the scope with respect to the bifunctional reagents and prepared a series of N-aminopyridinium salts. As summarized in Fig. 6, we observed that substrates bearing electron-rich or electron-deficient groups, such as methyl (**4a** and **4b**), methoxy (**4c**), and ester (**4d**) groups on the pyridyl ring successfully afforded the desired products. Concerning the importance of the 2-arylpyridine structural motif in pharmaceutical chemistry and materials science, we next evaluated the scope of the reaction with substrates containing a variety of aryl groups (**4e**–**4g**). The reactions of pyridinium substrates bearing methyl, phenyl, methoxy, ester or bromo groups at the C3 positions proceeded well to provide **4h**, **4i**, **4j**, **4k**, and **4l**. Bicyclic pyridinium substrate was also tolerable to give the desired product **4** **m**. Expanding the scope from the methyl substituent of N-tosylamide to other groups, such as ethyl (**4n**), ^n^propyl (**4o**), trifluoroethyl (**4p**), or phenyl propyl groups (**4q**) was also possible, leading to the formation of the corresponding products. Besides, the scope could be expanded to an N-mesyl group, which afforded the desired product in good yield (**4r**).

**Fig. 7:**
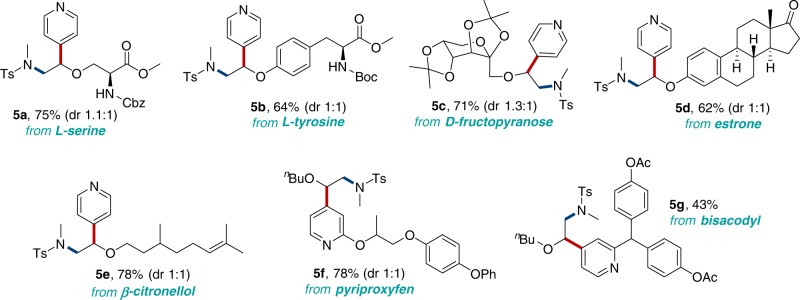
Late-stage aminopyridylation of biorelevant molecules. The reactions of derivatives of L-serine, L-tyrosine, D-fructopyranose, estrone, β-citronellol, pyriproxyfen, and bisacodyl

To demonstrate the broad synthetic utility of the current method, we assessed the potential of this convenient method for the late-stage modification of seven different classes of biorelevant complex molecules, as highlighted in Fig. 7. Specifically, the reaction of the amino acid derivatives, L-serine, and L-tyrosine under the standard reaction conditions effectively provided the corresponding products **5a** and **5b**. D-fructopyranose, estrone, and β-citronellol derivatives all underwent aminopyridylation smoothly, affording the desired products **5c**, **5d**, and **5e**. In addition, derivatives of pyriproxyfen and bisacodyl were successfully employed to provide the corresponding products **5f** and **5g** in moderate to good yield. Therefore, these results highlight the synthetic utility of the current aminopyridylation reactions with respect to the high tolerance of functional groups, such as ester, ketone, amide, acetal, ether, and pyridines in the complex molecules.

### Control experiments

**Fig. 8:**
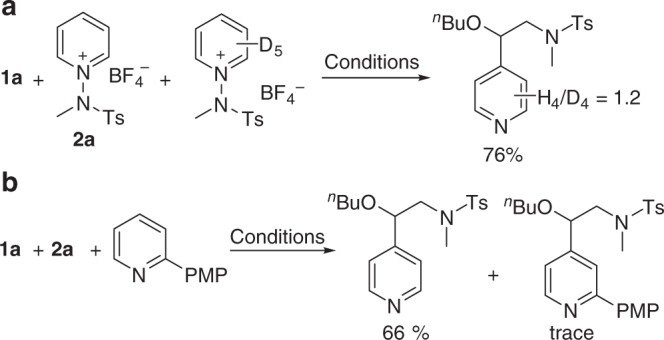
Control experiment. **a** Kinetic isotope experiments. **b** Reaction with mixtures of **2a** and 2-*p*-methoxyphenyl (PMP)-substituted pyridine

Having established that our developed method is generally applicable and broad in scope, we wished to scrutinize the proposed mechanism (see Supplementary Fig. 9). First, kinetic isotope experiments indicated that the deprotonation step is not rate-determining (Fig. 8a). Next, to determine whether the pyridine species generated in situ by single-electron transfer could act as a substrate, mixtures of **2a** and 2-*p*-methoxyphenyl (PMP)-substituted pyridine were subjected to the standard reaction conditions (Fig. 8b). As anticipated, the reaction occurred only with the pyridinium salt **2a**, whereas pyridine was not involved in the radical trapping step. Stern-Volmer quenching experiments were conducted to reveal that the quenching of the excited state of EY is directly proportional to the concentration of **2a** (see Supplementary Fig. 3 and 4). In addition, we measured the cyclic voltammetry of pyridinium salt **2a**, and the reduction potential of **2a** was –0.73 V (peak potential vs. Ag/Ag^+^, see Supplementary Fig. 6), indicating that the photocatalytic cycle could operate under oxidative quenching mechanism. The possibility of any formation of the EDA complex could be ruled out by absorption spectra measurements (see Supplementary Fig. 5).

## Discussion

In summary, we developed a direct 1,2-aminopyridylation of olefins induced by visible light using a variety of N-aminopyridinium salts as efficient bifunctional reagents to give straightforward access to synthetically and biologically relevant β-aminopyridine structural motifs under mild reaction conditions. In the process, N-aminopyridinium salts serve as both aminating and pyridylating reagents, which directly incorporate vicinal amino and pyridyl groups on the olefins in a controllable and selective manner. Combining experimental and computational methods, a detailed mechanistic understanding of the origin of the regioselectivity in the radical trapping step was obtained. The electrostatic attraction between the pyridinium nitrogen and sulfonyl group of β-amino radical directs radical trapping at the C4-position of pyridinium substrates. This strategy provides a convenient and powerful synthetic tool for the alkene aminopyridylation and is well suited for the late-stage modification of complex biorelevant molecules.

## Methods

### Representative procedure for the aminopyridylation of alkenes

Reactions were conducted in a test tube (16 mL) sealed with rubber septa. *N*-protected 1-aminopyridinium tetrafluoroborate (**2a**) (0.15 mmol), Eosin Y (0.5 mol%), and K_3_PO_4_ (0.12 mmol) were combined under N_2_ atmosphere. To the reaction mixture was added *n*-butylvinylether (**1a**) (0.1 mmol) in dimethylsulfoxide (DMSO, 1.0 mL). The sealed test tube was sonicated for 10 s and immediately placed at a reaction bath equipped with Kessil PR160 440 nm blue LEDs (25% intensity). The resulting mixture was stirred at room temperature for 3 h, diluted with ethyl acetate and washed with water for 3 times. The organic layer was dried over magnesium sulfate and filtered. The resulting mixture was concentrated under reduced pressure and purified by flash column chromatography on silica gel (acetone:*n*-hexane = 1:4) to obtain the desired product **3a** (84%, 30.5 mg) as a colorless oil.

## Supplementary information

Supplementary Information

Description of Additional Supplementary Files

Supplementary Data 1

Supplementary Data 2

Supplementary Data 3

## Data Availability

Experimental procedure and characterization data of new compounds are available within Supplementary Information. Computational details, optimized Cartesian coordinates of all structures, vibrational frequencies, and energy components. This material is available free of charge via the Internet.
