# Supplementary material for: Visible light induced alkene aminopyridylation using N-aminopyridinium salts as bifunctional reagents
Source: Nat Commun. 2019 Sep 11;10:4117. doi: 10.1038/s41467-019-12216-3 (PMC6739411; doi:10.1038/s41467-019-12216-3)
Supplement: Supplementary file 1 — Supplementary Information [file 41467_2019_12216_MOESM1_ESM.pdf]

Supplementary Information

Visible-Light-Induced Alkene Aminopyridylation Using N-Aminopyridinium Salts as Bifunctional Reagents

Moon et al.

Supplementary Methods

General Information

Unless stated otherwise, reactions were performed in flame-dried glassware. Analytical thin layer chromatography (TLC) was performed on precoated silica gel 60 F254 plates and visualization on TLC was achieved by UV light (254 and 365 nm). Flash column chromatography was performed on silica gel (400-630 mesh) or a CombiFlash® Rf + system with RediSep® Rf silica columns (230-400 mesh) using a proper eluent. ^{1}H NMR was recorded on Agilent Technologies DD2 600 MHz. Chemical shifts were quoted in parts per million (ppm) referenced to the appropriate solvent peak or 0.0 ppm for tetramethylsilane. The following abbreviations were used to describe peak splitting patterns when appropriate: br = broad, s = singlet, d = doublet, t = triplet, q = quartet, m = multiplet, dd = doublet of doublet, td = triplet of doublet, ddd = doublet of doublet of doublet. Coupling constants, J, were reported in hertz unit (Hz). ^{13}C NMR was recorded on Agilent Technologies DD2 150 MHz and was fully decoupled by broad band proton decoupling. ^{19}F NMR was recorded on Agilent Technologies DD2 564 MHz. Chemical shifts were reported in ppm referenced to the centerline of a triplet at 77.0 ppm of $CDCl_3$. High-resolution mass spectra were obtained by using EI method from Korea Basic Science Institute (Daegu) or ESI from KAIST Research Analysis Center (Daejeon). Commercial grade reagents and solvents were used without further purification except as indicated below.

Experimental Procedure

General procedure for visible-light-induced aminopyridylation of olefin

Reactions were conducted in test tube (16 ml) sealed with rubber septa. N-protected 1-aminopyridinium tetrafluoroborate (**2a**) (0.15 mmol), Eosin Y (0.5 mol%), and K_3PO_4 (0.12 mmol) were combined under N_2 atmosphere. To the reaction mixture was added n-butylvinylether (**1a**) (0.1 mmol) in dimethylsulfoxide (DMSO, 1.0 ml). The sealed test tube was sonicated for 10 seconds, and immediately placed at a reaction bath equipped with Kessil PR160-440 nm blue LEDs (25% intensity). The resulting mixture was stirred at room temperature for 3 h, diluted with ethyl acetate and washed with water for 3 times. The organic layer was dried over magnesium sulfate and filtered. The resulting mixture was concentrated under reduced pressure and purified by flash column chromatography on silica gel (acetone : n-hexane = 1:4) to obtain the desired product **3a** (84%, 30.5 mg) as a colorless oil.

2.0 mmol scale procedure for visible-light-induced metal-free amino pyridylation of olefin

Reactions were conducted in round-bottom-flask (100 ml) sealed with rubber septa. N-protected 1-aminopyridinium tetrafluoroborate (**2a**) (3.0 mmol), Eosin Y (0.01 mmol), and K_3PO_4 (2.4 mmol) were combined under N_2 atmosphere. To the reaction mixture was added n-butylvinylether (**1a**) (2.0 mmol) in dimethylsulfoxide (DMSO, 20.0 ml). The sealed test tube was sonicated for 10 seconds, and immediately placed at a reaction bath equipped with Kessil PR160-

440nm blue LEDs (100% intensity). The resulting mixture was stirred at room temperature for 6 h, diluted with ethyl acetate and washed with water for 3 times. The organic layer was dried over magnesium sulfate and filtered. The resulting mixture was concentrated under reduced pressure and purified by flash column chromatography on silica gel (acetone : n-hexane = 1:4) to obtain the desired product **3a** (83%, 598.7 mg) as a colorless oil.

Procedure for visible-light-induced metal-free amino pyridylation of biorelevant complex alkenes (5a-e)

Reactions were conducted in test tube (16 ml) sealed with rubber septa. N-protected 1-aminopyridinium tetrafluoroborate (**2a**) (0.30 mmol), Eosin Y (0.5 mol% when **5b** and **5c**, 2.0 mol% when **5a**, **5d** and **5e**), and K_3PO_4 (0.24 mmol) were combined under N_2 atmosphere. To the reaction mixture was added alkene (0.2 mmol) in dimethylsulfoxide (DMSO, 2.0 ml). The sealed test tube was sonicated for 10 seconds, and immediately placed at a reaction bath equipped with Kessil PR160-440 nm blue LEDs (25% intensity). The resulting mixture was stirred at room temperature for 4 h, diluted with ethyl acetate and washed with water for 3 times. The organic layer was dried over magnesium sulfate and filtered. The resulting mixture was concentrated under reduced pressure and purified by flash column chromatography on silica gel to obtain the desired product **5a-e**.

Procedure for visible-light-induced metal-free amino pyridylation using complex pyridine derivatives (5f and **5g)**

Reactions were conducted in test tube (16 ml) sealed with rubber septa. Complex pyridinium salt (0.10 mmol), Eosin Y (1.0 mol%), and K_3PO_4 (0.12 mmol) were combined under N_2 atmosphere. To the reaction mixture was added n-butylvinylether (**1a**) (0.20 mmol) in dimethylsulfoxide (DMSO, 1.0 ml). The sealed test tube was sonicated for 10 seconds, and immediately placed at a reaction bath equipped with Kessil PR160-440 nm blue LEDs (25% intensity). The resulting mixture was stirred at room temperature for 3 h, diluted with ethyl acetate and washed with water for 3 times. The organic layer was dried over magnesium sulfate and filtered. The resulting mixture was concentrated under reduced pressure and purified by flash column chromatography on silica gel to obtain the desired product **5f** and **5g**.

Preparation of Alkenes

Vinyl ethers and amides were purchased from commercial source or synthesized by using the following vinylation method[1].

General procedure for N-tosyl 1-aminopyridinium ylides.

To a solution of 1-aminopyridinium (1 equiv) in dichloromethane (0.2 M) were added triethylamine (2.2 equiv) and tosyl chloride (1.0 equiv) at room temperature. The reaction mixture was stirred at room temperature for 6 h. The resulting mixture was extracted with dichloromethane and water for three times, and washed with water. The combined organic layers were dried over magnessium sulfate, filtered and concentrated in vacuum. The resulting mixture was purified by flash column chromatography on silica gel (CH_2Cl_2 : MeOH = 20 : 1) to obtain N-tosyl 1-aminopyridinium ylides.

Large scale preparation of N-tosyl 1-aminopyridinium ylide

To a solution of 1-aminopyridinium iodide (40 mmol) in dichloromethane (0.2 M) were added triethylamine (2.2 equiv) and tosyl chloride (1.0 equiv) at room temperature. The reaction mixture was stirred at room temperature for 6 h. The resulting mixture was extracted with dichloromethane and water for three times, and washed with water. The combined organic layers were dried over magnessium sulfate, filtered and concentrated in vacuum. The resulting mixture was purified by flash column chromatography on silica gel (CH_2Cl_2 : MeOH = 20 : 1) to obtain N-tosyl 1-aminopyridinium ylides (8.47 g, 85%).

General procedures for N-tosyl 1-aminopyridinium tetrafluoroborate salts (when N-Me or Et)

To a solution of N-tosyl 1-aminopyridinium ylide (1 equiv) in dichloromethane (0.1 M) were added trimethyloxonium tetrafluoroborate (meerwein's reagent, 1.1 equiv) at room temperature. The reaction mixture was stirred at room temperature for 3 h. The resulting mixture was concentrated under reduced pressure. The product was recrystallized with diethylether from CH_2Cl_2 and MeOH (20:1) solution at -20 ℃. A solide product was obtained.

Large scale preparation of 2a

To a solution of N-tosyl 1-aminopyridinium ylide (20 mmol) in dichloromethane (0.1 M) were added trimethyloxonium tetrafluoroborate (meerwein's reagent, 1.1 equiv) at room temperature. The reaction mixture was stirred at room temperature for 3 h. The resulting mixture was concentrated under reduced pressure. The product was recrystallized twice with diethylether from CH_2Cl_2 and MeOH (20:1) solution at -20 ℃. A solide product (4.54 g, 65%) was obtained.

General procedures for N-protected 1-aminopyridinium tetrafluoroborate salts (when N-alkyl)

To a solution of N-tosyl 1-aminopyridinium ylide (1.0 equiv) in acetonitrile (1.0 M) was added alkyl iodide (2.0 equiv, or 3.0 equiv of alkyl bromides and 2.0 equiv of sodium iodide can be used instead of alkyl iodides) at room temperature. The reaction mixture was stirred at 70 ℃ for overnight. The resulting mixture was dilluted with dichloromethane and washed with water for three times. The combined organic layers were dried over magnessium sulfate, filtered and concentrated in vacuum. The resulting mixture was purified by flash colum chromatography on silical gel (CH_2Cl_2 : MeOH = 10 : 1) to obatain N-tosyl N-alkylated pyridinium iodide.

To a solution of N-tosyl N-alkylated pyridinium iodide in MeOH (0.2 M), was added silver tetrafluoroborate (1.2 equiv). The reaction mixture was sonicated at room temperature for 30 seconds. The resulting mixture was filtered with celite, extracted with dichloromethane. The solution was concentrated in vacuum. A solid product was obatained.

Preparation of N-amino pyridinium salts from pyridines

Amination of pyridine was conducted using the previous developed method with hydroxylamine-O-sulfonic acid[2] or O-mesitylsulfonylhydroxylamine (MSH)[3]. MSH was synthesized by using previous developed method[4].

Control experiments

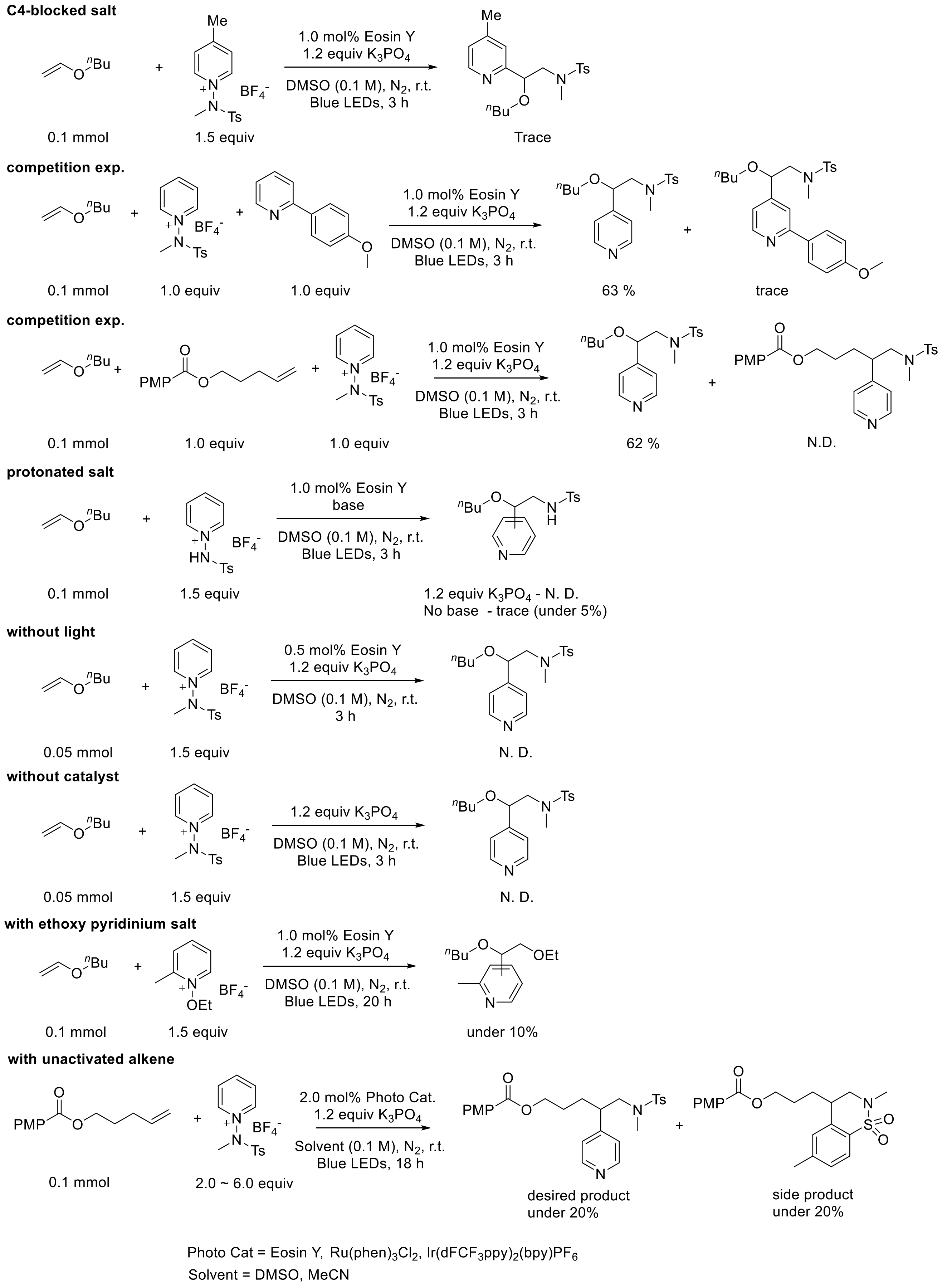

Supplementary Figure 1. Control experiments conducted in this study

TEMPO exp.

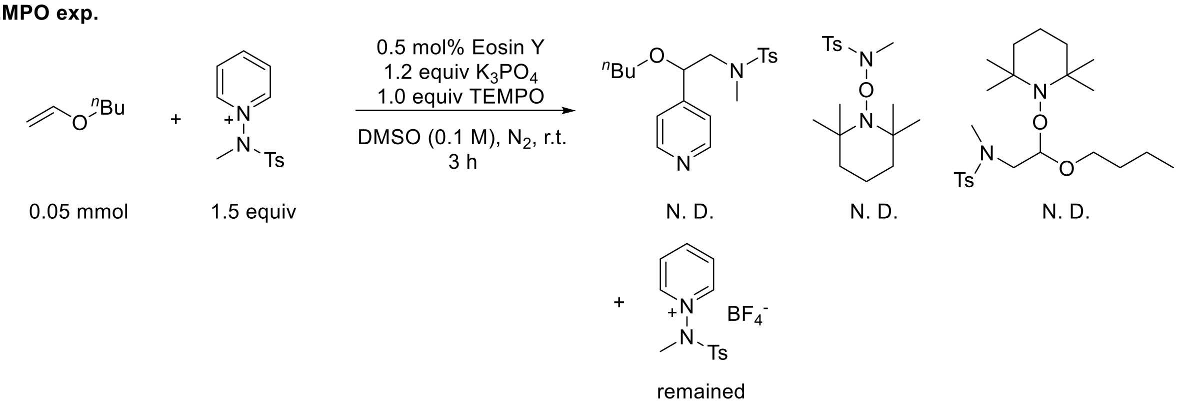

TEMPO exp. with 1.0 equiv of eosin Y

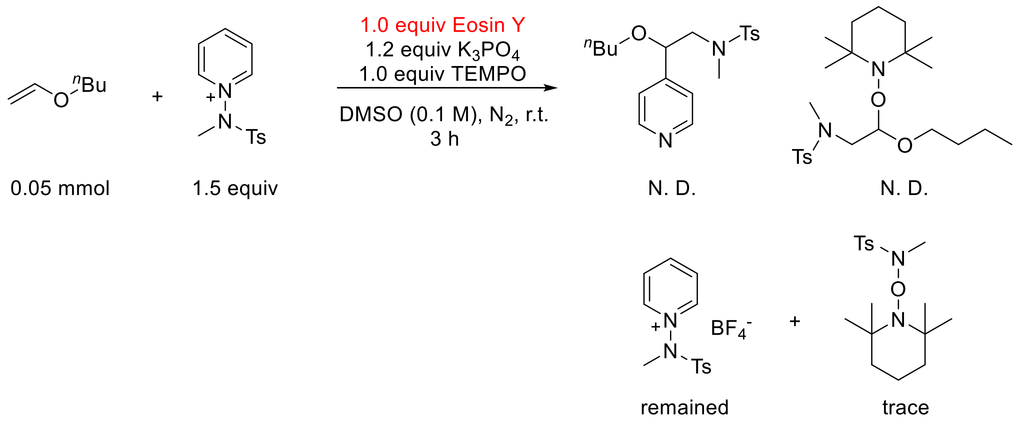

#	m/z	I
1	107.0617	126
2	112.1125	128
3	140.1437	231
4	142.1602	199
5	156.1385	163
6	158.1536	172
7	180.1355	131
8	238.0498	92
9	263.0857	138748
10	264.0859	11806
11	265.0812	2097
12	266.0841	141
13	271.0517	85
14	321.2175	94
15	363.1724	88
16	377.0596	311
17	410.1886	533
18	411.1931	109
19	412.1977	107
20	429.2244	95
21	430.2185	161
22	445.2705	102
23	475.3253	355
24	476.3248	99
25	506.2157	94
26	516.1973	94
27	525.1626	87
28	528.5126	100
29	560.0959	166
30	692.6791	107

→ **Mass of remained pyridinium salt** (#9)

→ (#15)

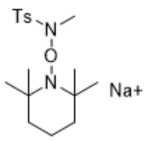

Chemical Formula: $C_{17}H_{28}N_2NaO_3S^+$
Exact Mass: 363.1713

Supplementary Figure 2. HRMS measurement for detection of TEMPO adduct

Stern-Volmer Quenching Experiment

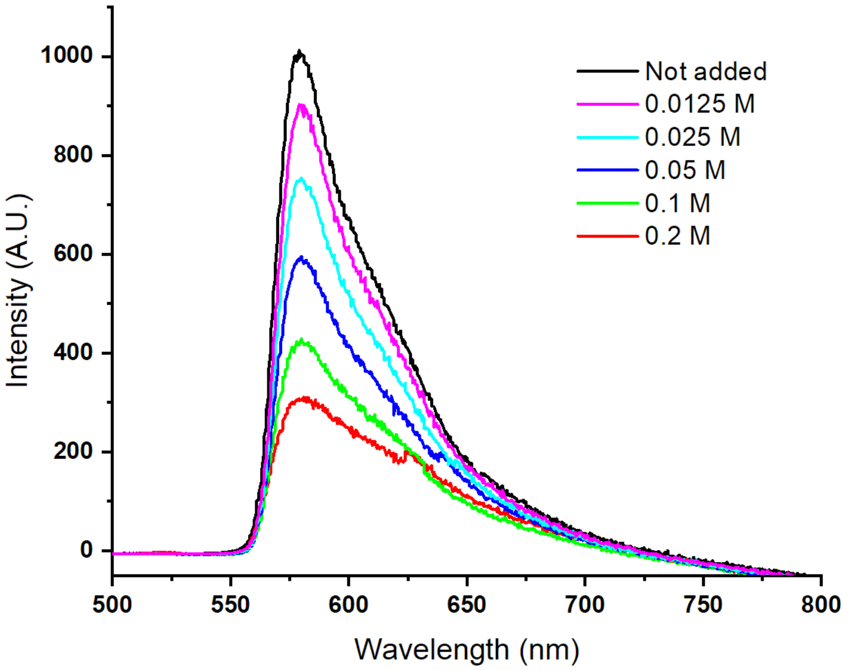

Supplementary Figure 3. Fluorescence quenching of the emission of Eosin Y (0.167 mM in DMSO) in the presence of increasing amounts of pyridinium salt (**2a**) at room temperature. Excitation wavelength : 440 nm.

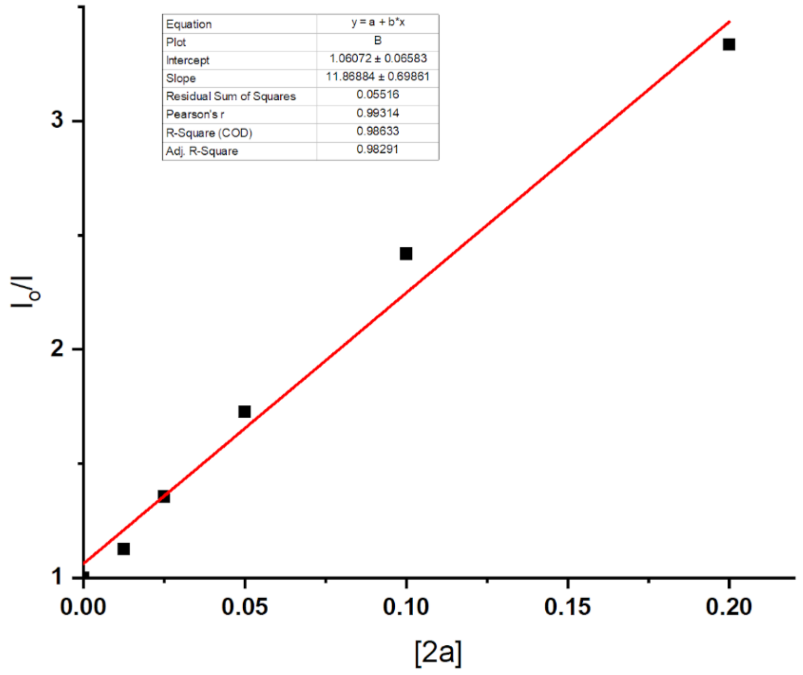

Supplementary Figure 4. Stern-Volmer quenching plot**.**

Absorption Spectra

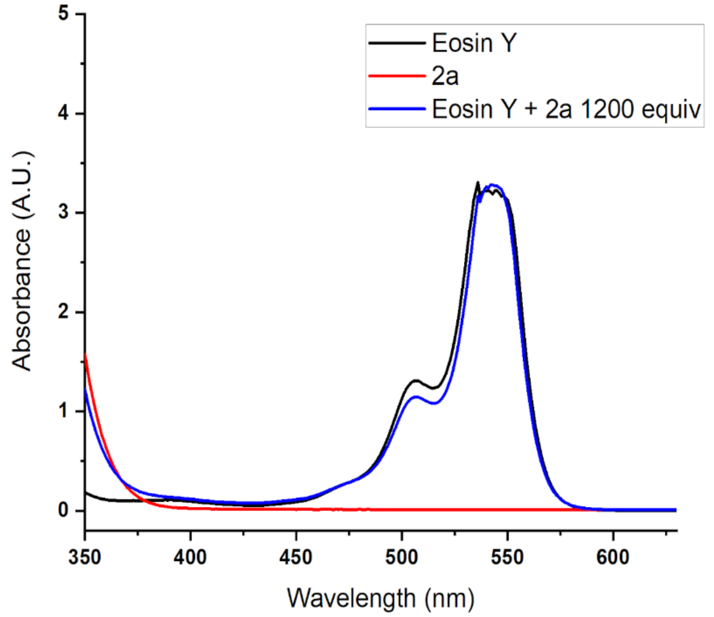

Supplementary Figure 5. Absorption spectra of the Eosin Y (0.056 mM in DMSO), pyridinium salt (**2a**, 1200 equiv), and mixture of Eosin Y and **2a** in DMSO at room temperature.

Cyclic Voltammetry

Cyclic voltammetry was measured by a potentiostat (CH instrument, 600E) with conventional three electrode system (Reference electrode: Ag/Ag+, working electrode : Glassy carbon, counter electrode : Pt wire, Supporting electrolyte : 0.1 M NBu_4PF_6 in CH_3CN) at 100 mV/sec of scan rate.

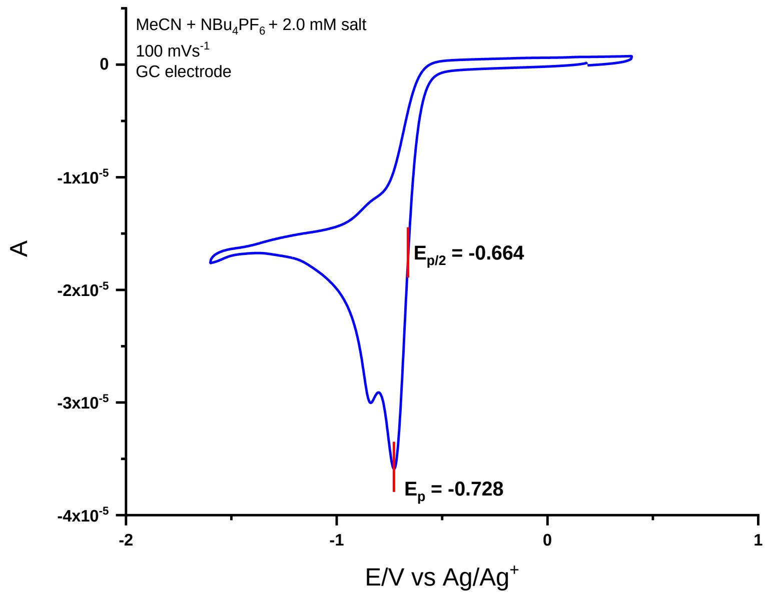

Supplementary Figure 6. CV of **2a** (2.0 mM in CH_3CN)

Quantum yield measurements

Kessil PR160-456nm was used for measurement of quantum yield

Determination of the light intensity at 456 nm

According to the procedure of Yoon, the photon flux of the LED[5] was determined by standard ferrioxalate actinometry. A 0.15 M solution of ferrioxalate was prepared by dissolving potassium ferrioxalate hydrate (0.737 g) in H_2SO_4 (10 mL of a 0.05 M solution). A buffered solution of 1,10-phenanthroline was prepared by dissolving 1,10-phenanthroline (5.0 mg) and sodium acetate (1.13 g) in H_2SO_4 (5.0 mL of a 0.5 M solution). Both solutions were stored in the dark. To determine the photon flux of the LED, the ferrioxalate solution (2.0 mL) was placed in a cuvette and irradiated for 90 seconds at λmax = 456 nm. After irradiation, the phenanthroline solution (0.35 mL) was added to the cuvette and the mixture was allowed to stir in the dark for 1 h to allow the ferrous ions to completely coordinate to the phenanthroline. The absorbance of the solution was measured at 510 nm. A non-irradiated sample was also prepared and the absorbance at 510 nm was measured. Conversion was calculated using Supplementary Equation 1.

$$mol\ of\ Fe^{2+} = \frac{V \times \Delta A_{510\,nm}}{l \times \epsilon} = \frac{(0.00235\ L) \times (1.427)}{(1.00\ cm) \times (11{,}100 \frac{L}{mol} \times cm^{-1})} = 3.02 \times 10^{-7} mol \quad (1)$$

V is the total volume (0.00235 L) of the solution after addition of phenanthroline, ΔA is the difference in absorbance at 510 nm between the irradiated and non-irradiated solutions, l is the path length (1.00 cm), and ε is the molar absorptivity of the ferrioxalate actinometer at 510 nm (11,100 $Lmol^{-1}$ cm^{-1}).[6] The photon flux can be calculated using Supplementary Equation 2.

$$\text{Photon flux} = \frac{mol\ of\ Fe^{2+}}{\emptyset \times t \times f} = \frac{3.02 \times 10^{-7}\ mol}{(0.84) \times (90\ s) \times (0.954)} = 4.19 \times 10^{-9}\ einstein/s \quad (2)$$

Where Φ is the quantum yield for the ferrioxalate actinometer (0.84 at λ = 456 nm)[7] is the irradiation time (90 s), and f is the fraction of light absorbed at 456 nm by the ferrioxalate actinometer. This value is calculated using Supplementary Equation 3 where $A_{456\ nm}$ is the absorbance of the ferrioxalate solution at 456 nm. An absorption spectrum gave an $A_{456\ nm}$ value of 1.34, indicating that the fraction of absorbed light (f) is 0.954.

$$f = 1 - 10^{-A_{456\,nm}} = 0.954 \quad (3)$$

The photon flux was thus calculated (average of three experiments) to be $4.19 \times 10^{-9}\ einstein/s$

Determination of the reaction quantum yield.

The reaction mixture was stirred and irradiated by Kessil PR160-456nm blue LED (25% intensity) for 600 s. After irradiation, the reaction mixture was diluted with ethyl acetate and washed with water for 3 times. The resulting organic layer dried over magnessium sulfate and solvent was removed under vacuum. The yield of product was determined by ^{1}H NMR analysis using dimethylterephthalate as an internal standard. The yield of **3a** was determined

to be 79% (7.9×10^{-5} $mol\ of\ \mathbf{3a}$). The reaction quantum yield (Φ) was determined using Supplementary Equation 4 where the photon flux is 4.19×10^{-9} $einstein/s$ (determined by actinometry as described above), t is the reaction time (600 s) and f is the fraction of incident light absorbed by the catalyst, determined using Supplementary Equation 3. An absorption spectrum of the catalyst (0.0005 M) gave an absorbance value of 1.215 at 456 nm (Supplementary Figure 8), indicating that the fraction of light absorbed by the photocatalyst (f) is 0.939

$$\Phi = \frac{mol\ of\ product}{flux \times t \times f} \quad (4)$$

$$\Phi = \frac{7.9 \times 10^{-5}\ mol}{4.19 \times 10^{-9} einstein \cdot s^{-1} \times 600\ s \times 0.939} = 33.5$$

The reaction quantum yield (Φ) was calculated to be 33.5

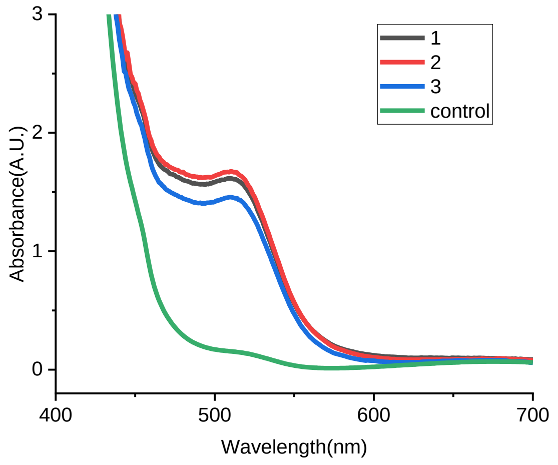

Supplementary Figure 7. Absorption spectra of three irradiation experiments and non-irradiation experiments

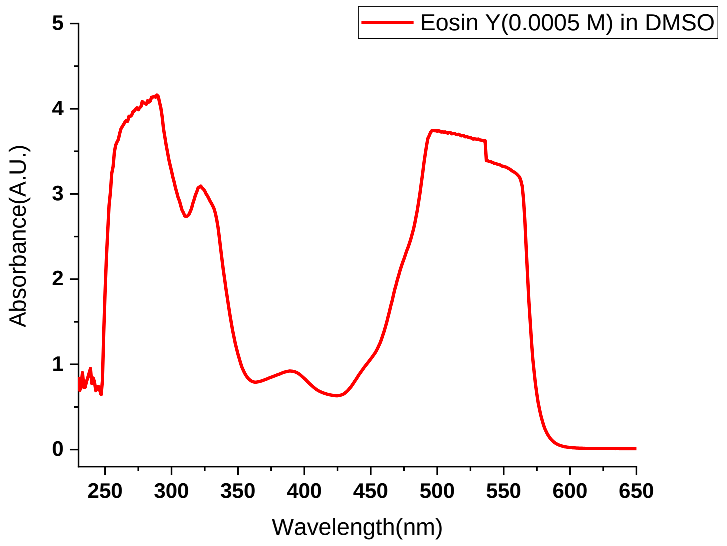

Supplementary Figure 8. Absorption spectra Eosin Y(0.0005 M) in DMSO

Proposed Mechanism

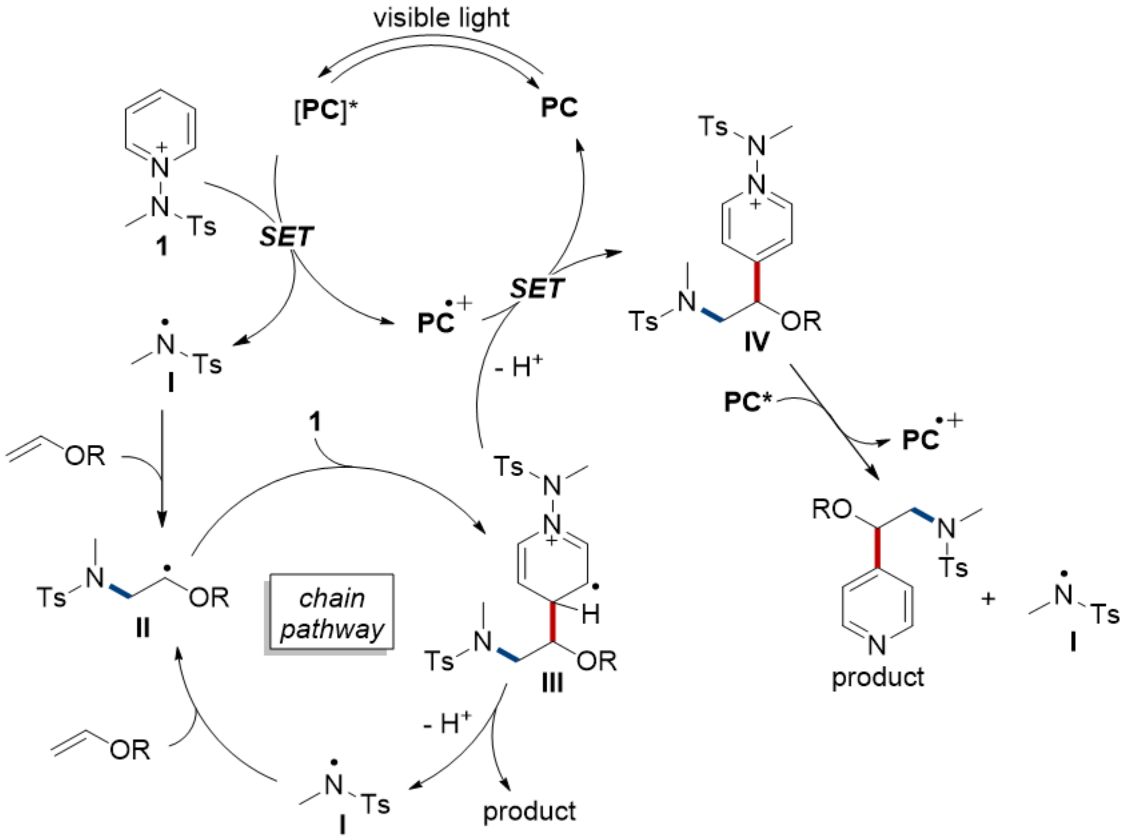

Supplementary Figure 9. Plausible mechanism

On the basis of control experiments and computational studies, a plausible mechanism for the alkene aminopyridylation is proposed in Supplementary Figure 9. Upon irradiation with visible light, the excited state EY* photoreduces the N-aminopyridinium salt by SET to generate N-centered radical **I**. The resulting aminyl radical **I** reacts with an electron-rich olefin to afford alkyl radical intermediate **II**. Subsequently, intermediate **II** adds to the C4-position of another pyridinium salt to form intermediate **III**, which undergoes deprotonation and N–N bond cleavage to form the final product and aminyl radical **I**. The resultant radical **I** initiates the radical chain pathway. An alternate reaction pathway involving rearomatization and reduction by SET events in the photoredox catalytic cycle can be envisioned. The measured high reaction quantum yield of Φ = 33.5 indicates that the radical chain pathway was quite productive in the overall reaction.

GC-MS Analysis

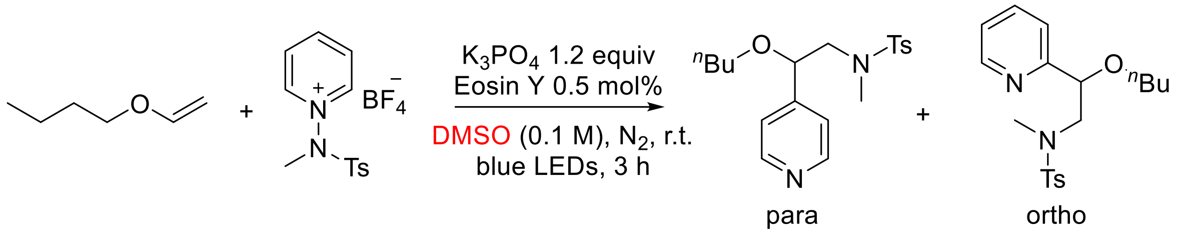

para : ortho = 13 : 1

Signal : TIC: MYH09-89-1.D\data.ms

peak #	R.T. min	first scan	max scan	last scan	PK TY	peak height	corr. area	corr. % max.	% of total
1	21.979	3369	3389	3409	BB 3	1703005	113965226	7.97%	7.380%
2	22.558	3412	3490	3568	BB 2	5417792	1430307065	100.00%	92.620%

Sum of corrected areas: 1544272290

YLJ_Acylcouling.M Fri May 10 16:08:08 2019

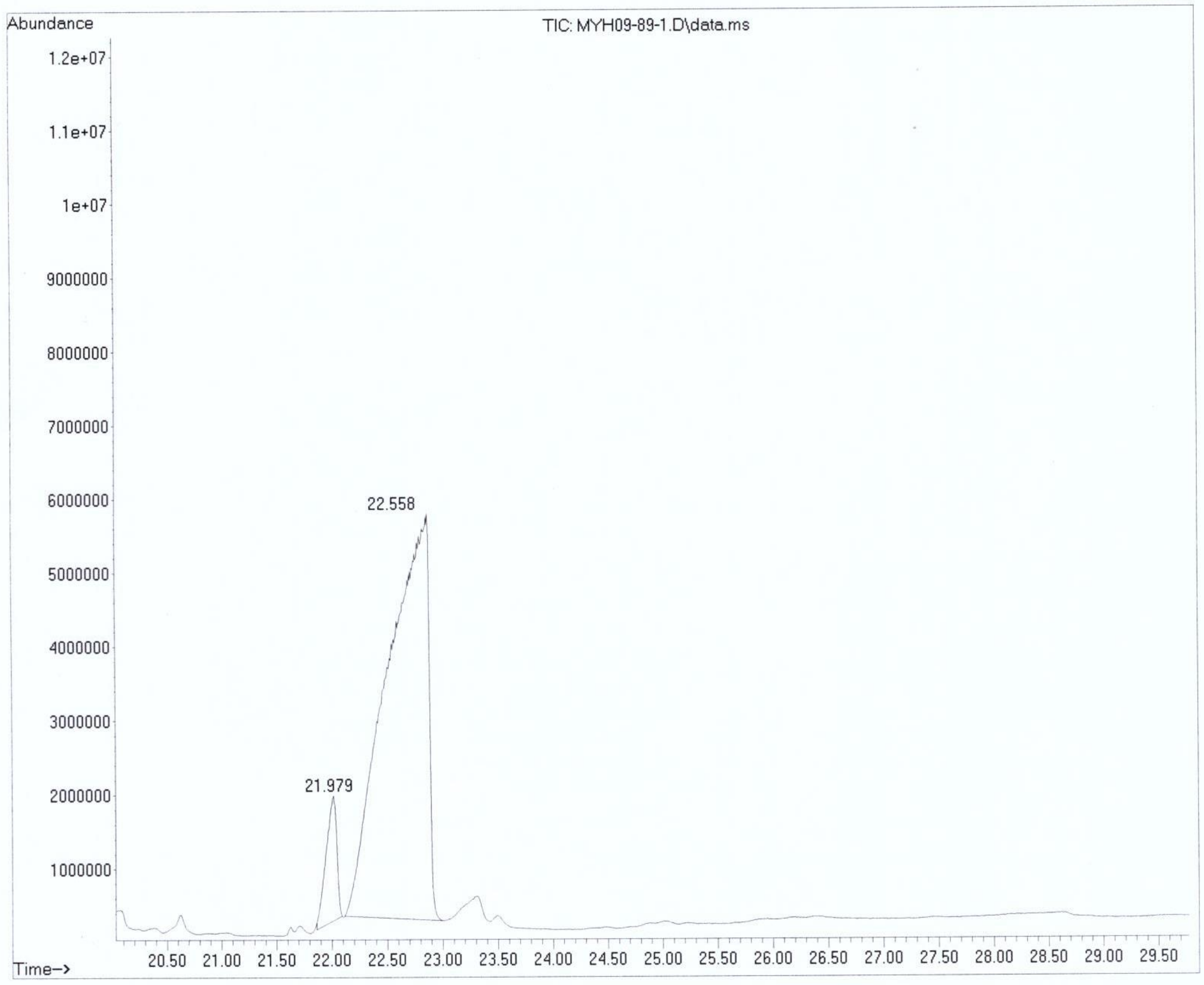

Supplementary Figure 10. C4/C2 ratio of **3a** in DMSO solvent. Regioisomeric was measured by GC-MS.

```
File       :D:\MassHunter\GCMS\3\data\MYH\MYH09-89-1.D
Operator   : MYH
Acquired   : 10 Apr 2019  14:54      using AcqMethod KIW-03.M
Instrument :   GCMSD
Sample Name: MYH09-89-1-crude
Misc Info  :
Vial Number: 13
```

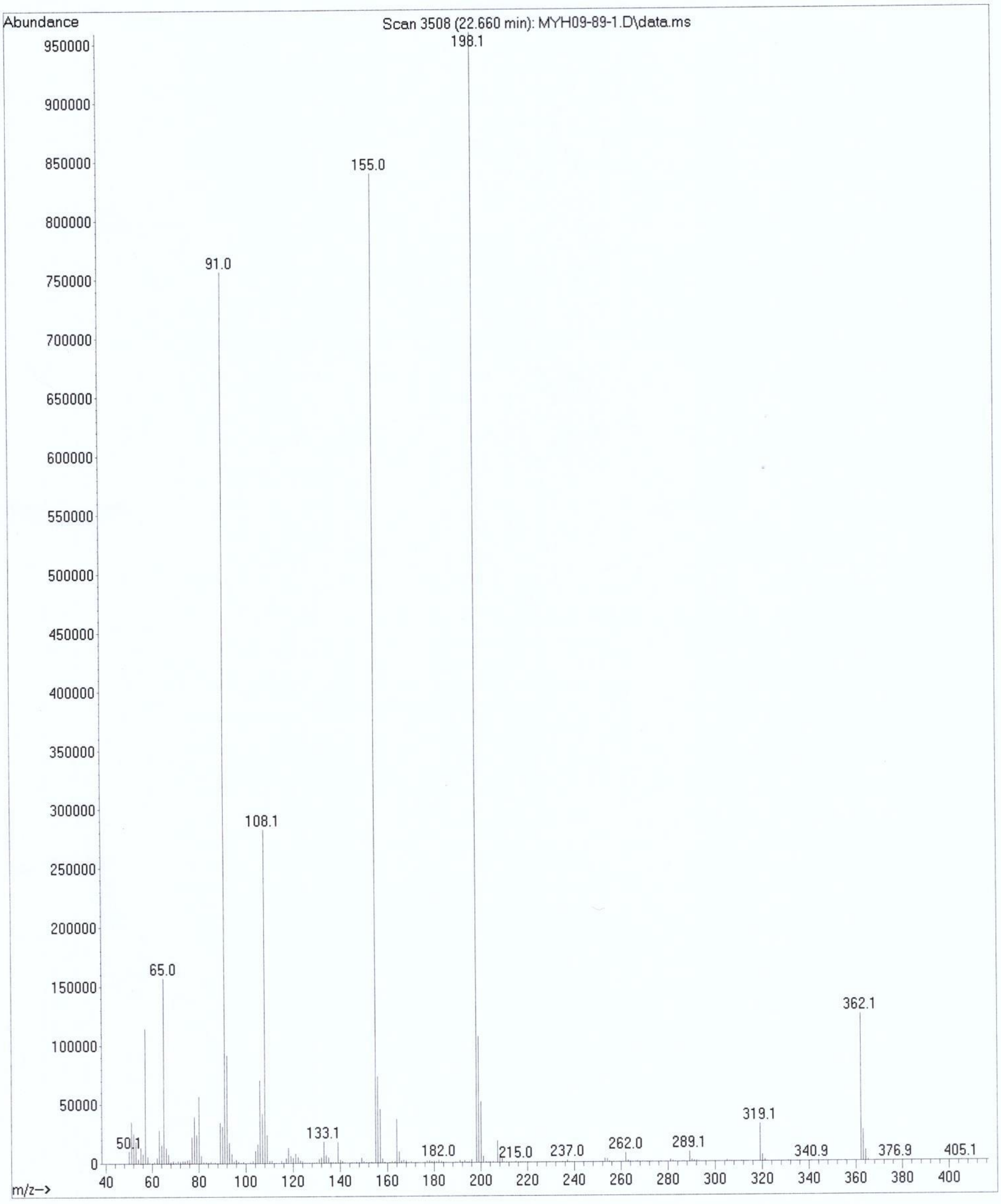

Supplementary Figure 11. Mass spectrum of **3a** determined by GC-MS.

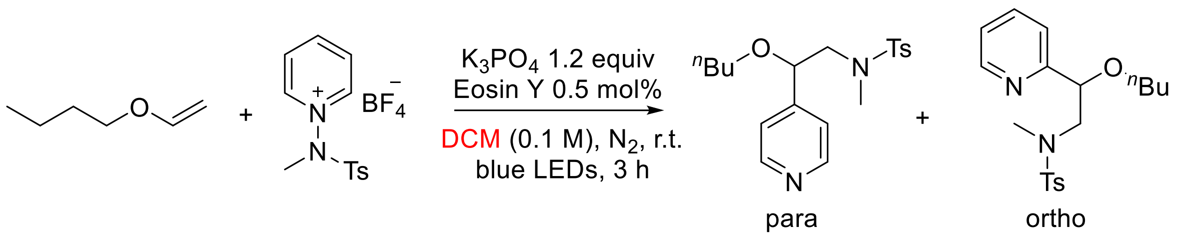

para : ortho = 47 : 1

Signal : TIC: MYH09-89-2.D\data.ms

peak #	R.T. min	first scan	max scan	last scan	PK TY	peak height	corr. area	corr. % max.	% of total
1	21.810	3343	3359	3376	BB 2	449502	19904667	2.11%	2.068%
2	22.370	3386	3457	3528	BB 4	5990833	942792145	100.00%	97.932%

Sum of corrected areas: 962696812

YLJ_Acylcouling.M Fri May 10 16:03:11 2019

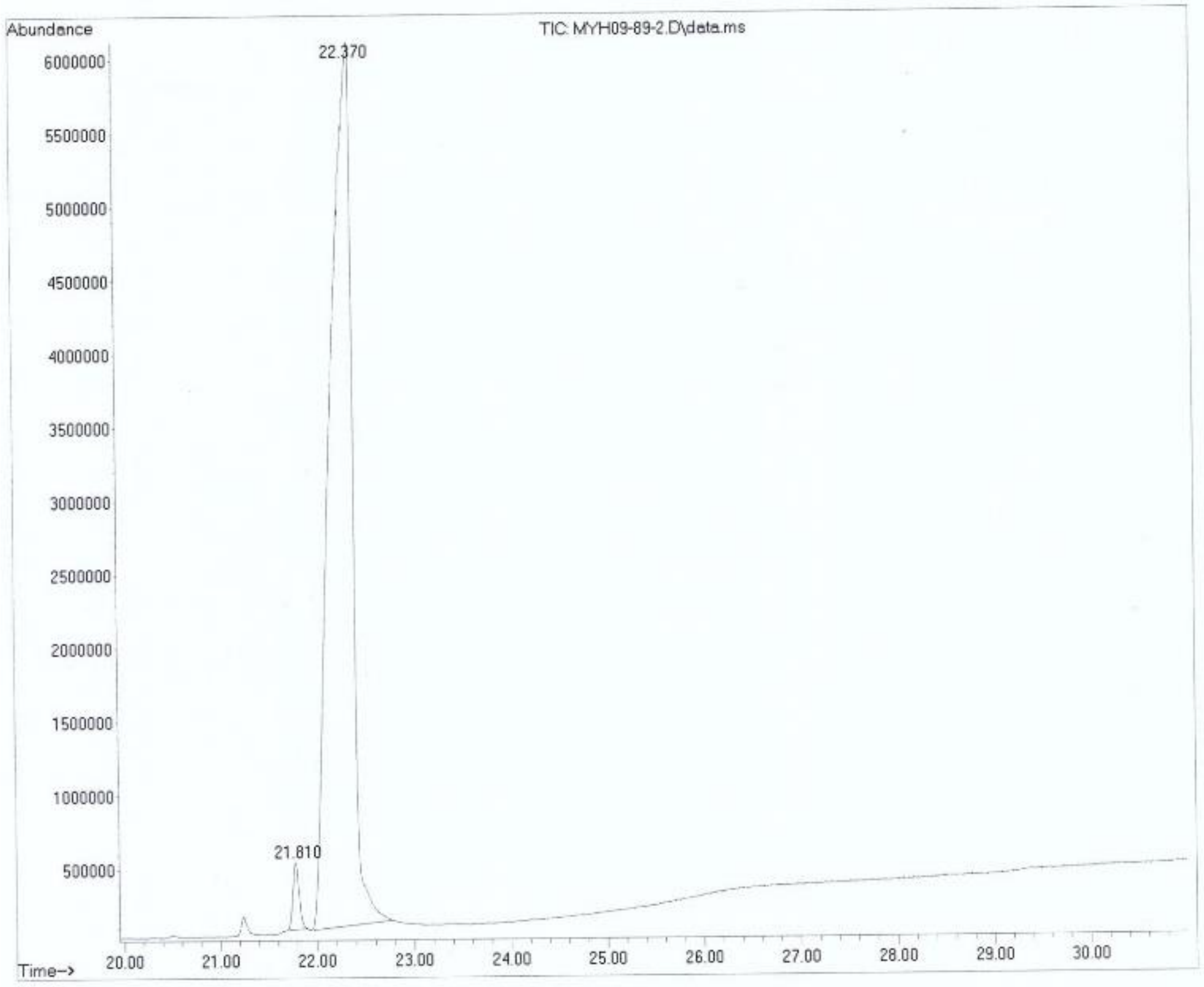

Supplementary Figure 12. C4/C2 ratio of **3a** in DCM solvent. Regioisomeric was measured by GC-MS.

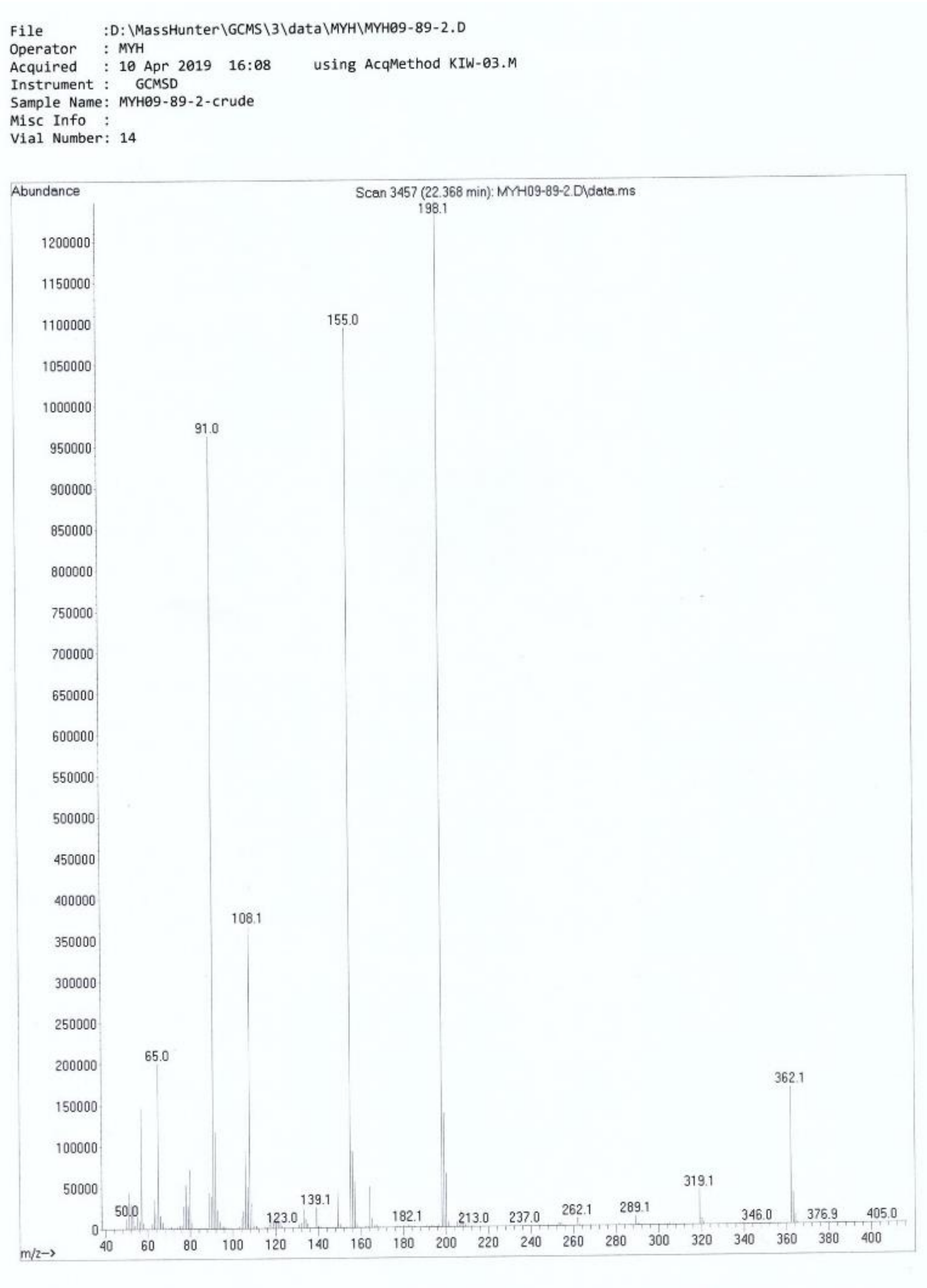

Supplementary Figure 13. Mass spectrum of **3a** determined by GC-MS.

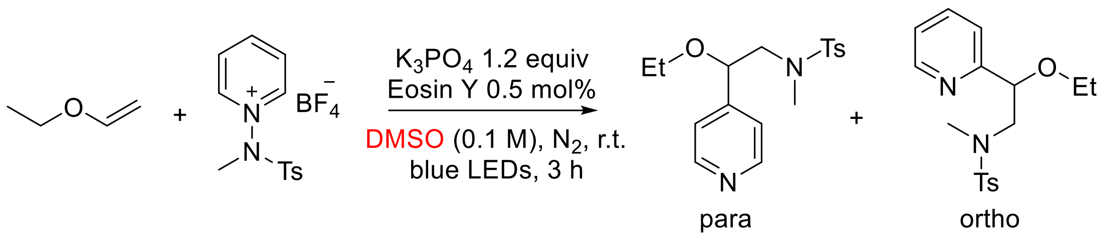

para : ortho = 10 : 1

Signal : TIC: MYH10-10-1.D\data.ms

peak #	R.T. min	first scan	max scan	last scan	PK TY	peak height	corr. area	corr. % max.	% of total
1	23.583	3651	3669	3688	BB	2684297	155131618	9.73%	8.866%
2	24.124	3698	3764	3830	BB 3	8367505	1594684343	100.00%	91.134%

Sum of corrected areas: 1749815961

YLJ_Acylcouling.M Fri May 10 15:54:59 2019

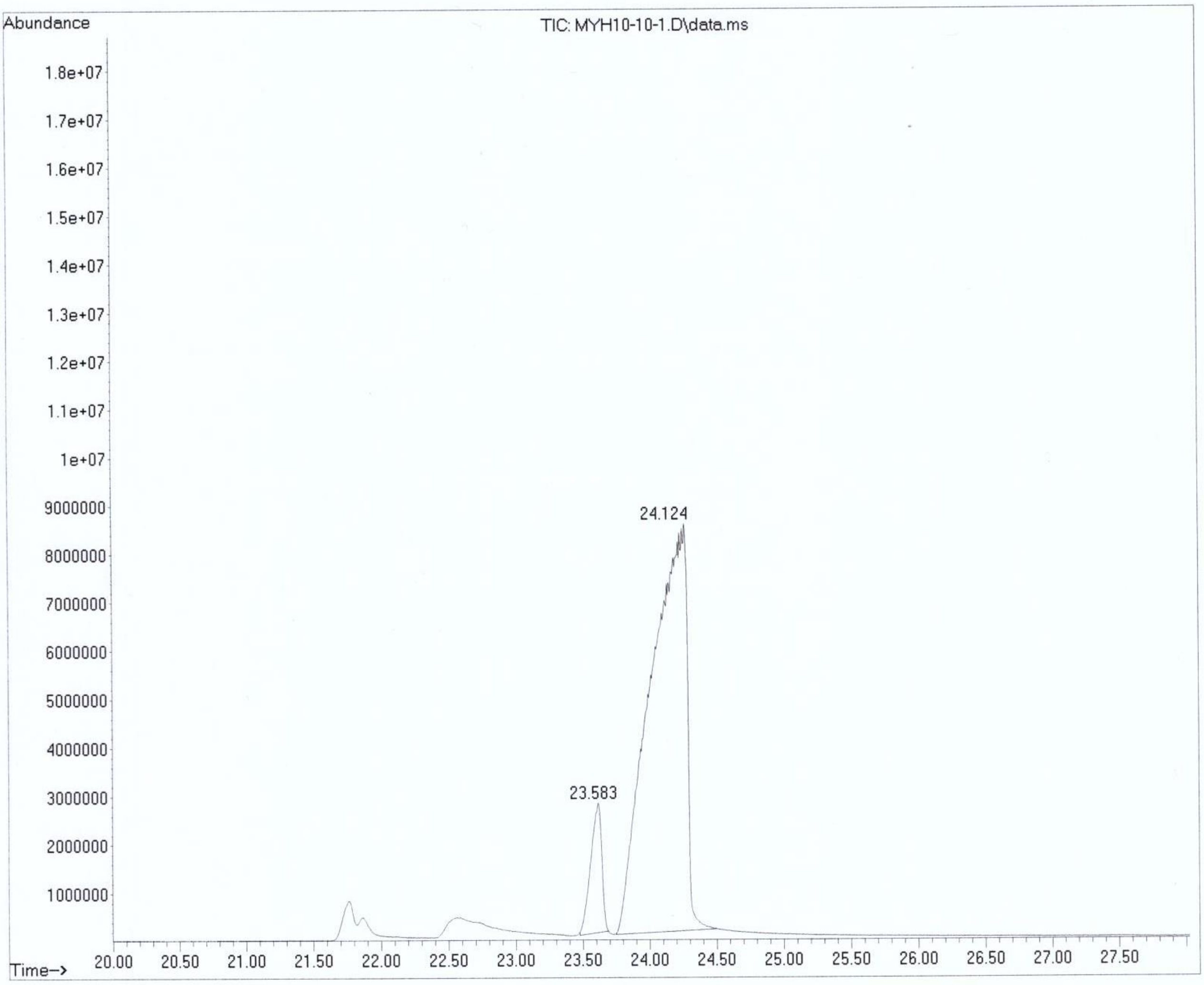

Supplementary Figure 14. C4/C2 ratio of **3b** in DMSO solvent. Regioisomeric was measured by GC-MS.

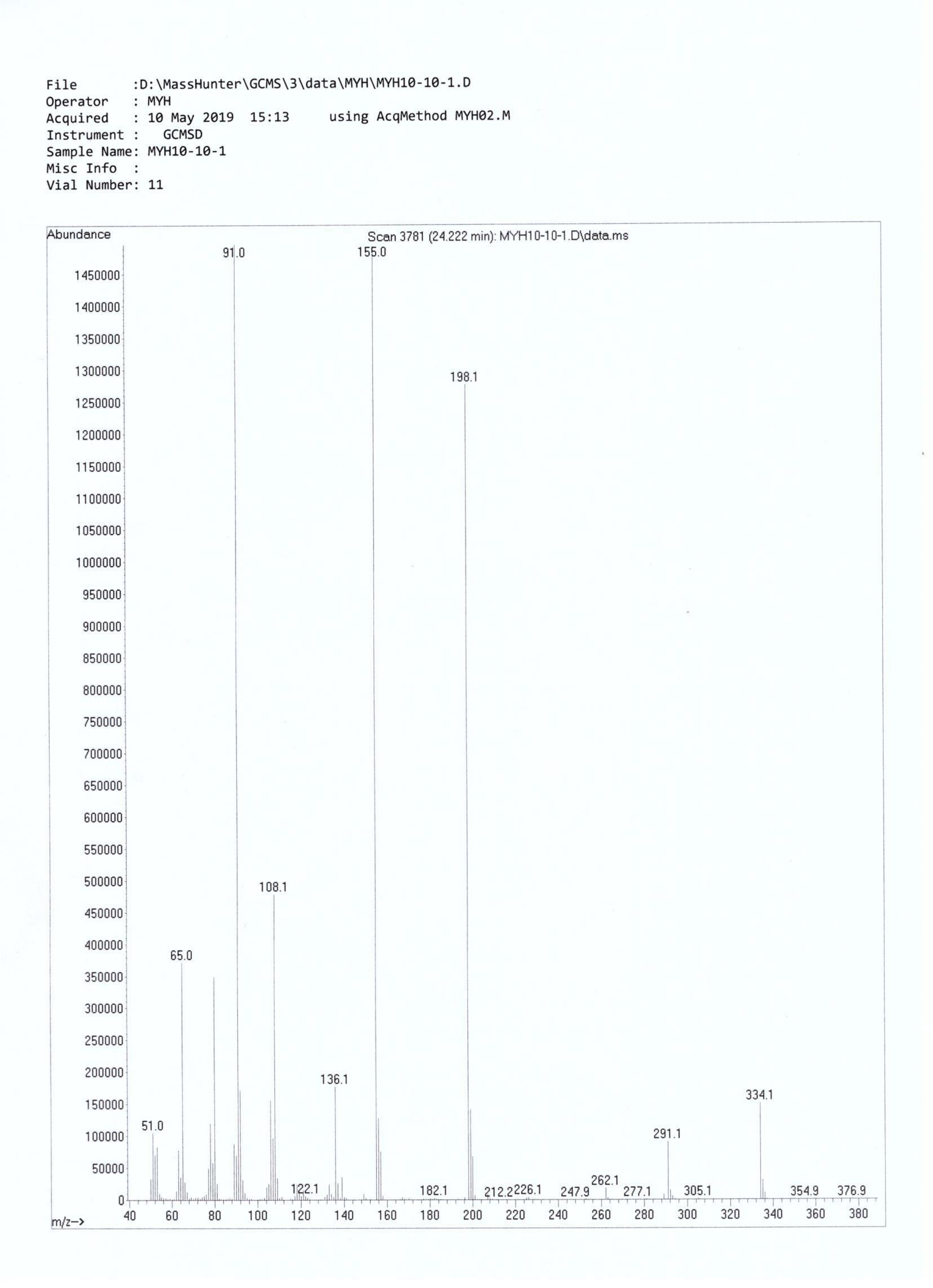

Supplementary Figure 15. Mass spectrum of **3b** determined by GC-MS.

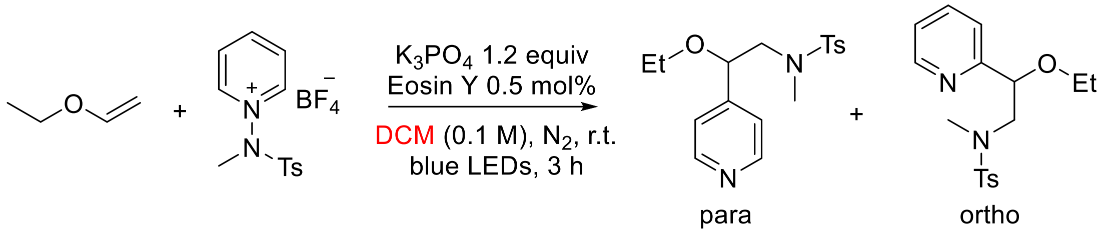

para : ortho = 38 : 1

Signal : TIC: MYH10-10-2-crude.D\data.ms

peak #	R.T. min	first scan	max scan	last scan	PK TY	peak height	corr. area	corr. % max.	% of total
1	23.583	3651	3669	3688	BB 4	214667	9563704	2.62%	2.554%
2	24.374	3698	3808	3917	BB 2	2803351	364932727	100.00%	97.446%

Sum of corrected areas: 374496431

YLJ_Acylcouling.M Fri May 10 15:57:31 2019

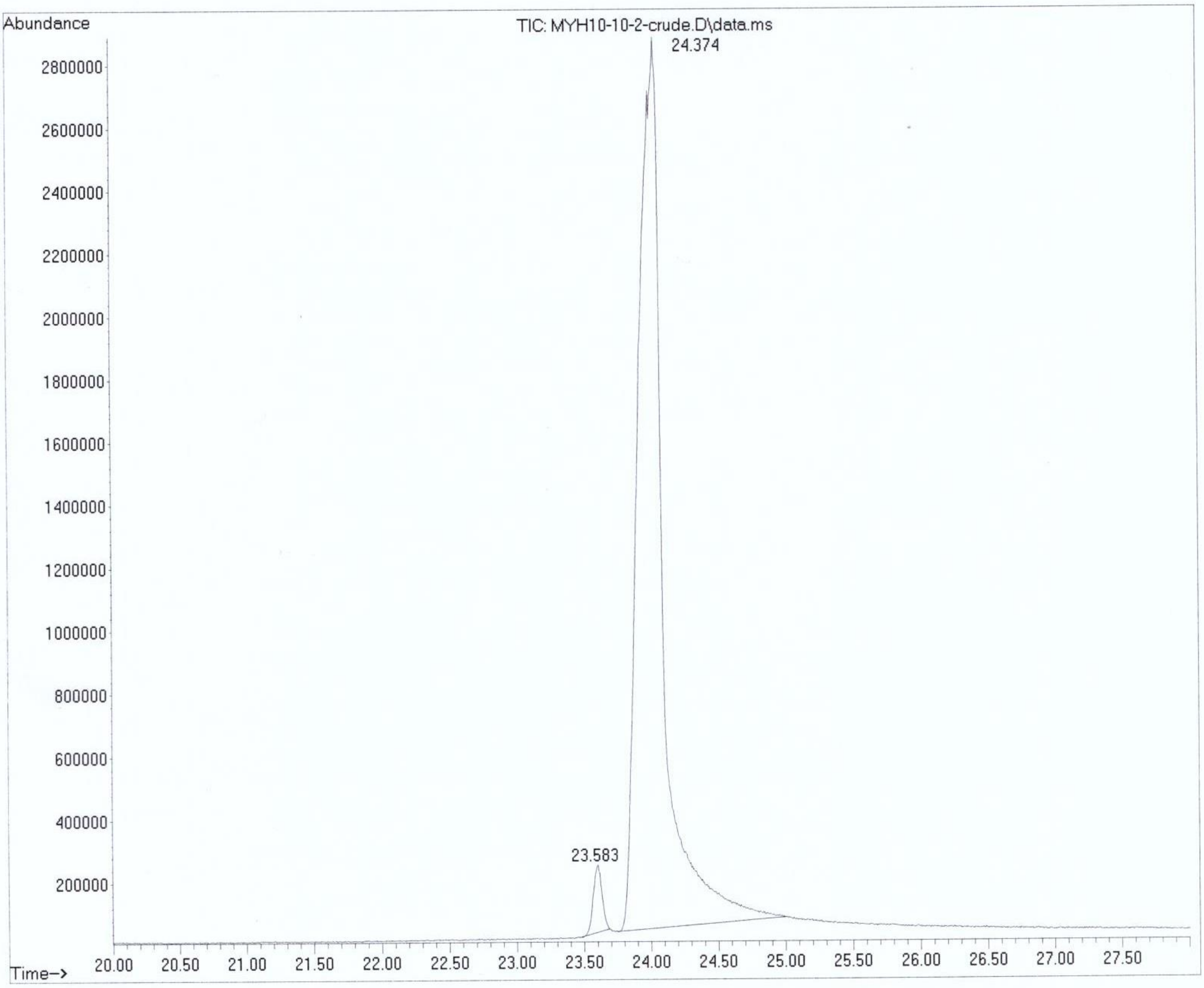

Supplementary Figure 16. C4/C2 ratio of **3b** in DCM solvent. Regioisomeric was measured by GC-MS.

File   :D:\MassHunter\GCMS\3\data\MYH\MYH10-10-2-crude.D
Operator   : MYH
Acquired   : 10 May 2019 11:38   using AcqMethod MYH02.M
Instrument :   GCMSD
Sample Name: MYH10-10-2-crude
Misc Info :
Vial Number: 13

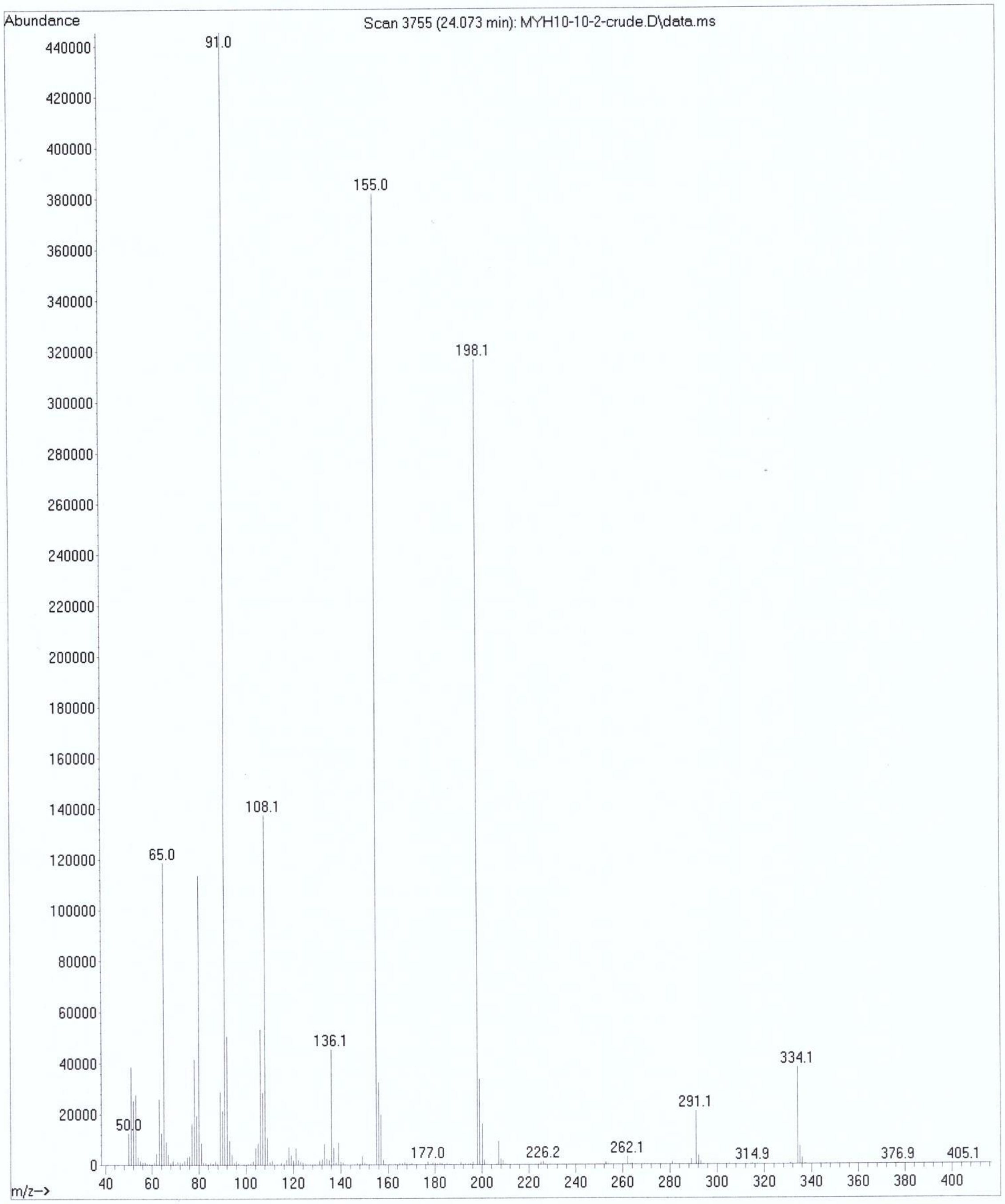

Supplementary Figure 17. Mass spectrum of **3b** determined by GC-MS.

Computational Details

All calculations except single point calculations were conducted using DFT[8] as implemented in the Jaguar 9.1 suite[9] of ab initio quantum chemistry programs with Becke's BHandHLYP levels of theory.[10] Geometry optimizations were proceeded using the 6-311G** basis set. Analytical vibrational frequencies within the harmonic approximation were calculated using the 6-311G** basis to confirm proper convergence to well-defined minima or saddle points on the potential energy surface. Solvation energies were calculated using a self-consistent reaction field (SCRF)[11-13] approach based on accurate numerical solutions of the Poisson-Boltzmann equation and were performed with the 6-311G** basis at the optimized gas-phase geometry with the dielectric constant of $\varepsilon = 46.48$ for dimethylsulfoxide and $\varepsilon = 9.08$ for dichloromethane. As is the case for all continuum models, the solvation energies are subject to the empirical parametrization of the atomic radii that are used to generate the solute surface. The standard set of optimized radii in Jaguar was used for H (1.150 Å), C (1.900 Å), N (1.600 Å), O (1.600 Å), and S (1.900 Å).[14] At last, the energies of the optimized structures were reevaluated by additional single point calculations on each optimized geometry using DFT as implemented in the ORCA 4.0.1 suite[15] of ab initio quantum chemistry programs with Truhlar's PW6B95 functional[16] including Grimme's D3 dispersion correction and Weigend's quadruple-ζ quality basis set def2-QZVP.[17,18] The Gibbs free energies in solution phase G(sol) were computed with the following protocol.

$$G(sol) = G(gas) + G^{solv} \quad (5)$$

$$G(gas) = H(gas) - TS(gas) \quad (6)$$

$$H(gas) = E(SCF) + ZPE \quad (7)$$

$$\Delta E(SCF) = \Sigma E(SCF) \text{ for products} - \Sigma E(SCF) \text{ for reactants} \quad (8)$$

$$\Delta G(sol) = \Sigma G(sol) \text{ for products} - \Sigma G(sol) \text{ for reactants} \quad (9)$$

G(gas) is the free energy in gas phase; G^{solv} is the free energy of solvation; H(gas) is the enthalpy in gas phase; T is the temperature (298.15K); S(gas) is the entropy in gas phase; E(SCF) is "raw" electronic energy as computed from the SCF procedure which is the self-consistent field energy, and ZPE is the zero point energy. The entropy we refer is specifically vibrational/rotational/translational entropy of the solute(s), and the entropy of the solvent is implicitly comprised in the continuum solvation model.

Orbital energies and distortion-interaction energies in Supplementary Figure 3 and 4 were calculated with PW6B95-D3/def2-QZVP level of theory. And the atomic charges from electrostatic potential were examined in the aid of CHELPG program developed by Breneman and Wiberg,[19] which is implemented in ORCA. Bonding energy decomposition analysis[20] was conducted as implemented in Amsterdam Density Functional (ADF) 2018 suite of ab initio quantum chemistry programs.[21,22] Calculation were conducted using BHandHLYP functional and QZ4P basis set with no frozen core and supported by zeroth-order regular approximation (ZORA) which supports scalar level of relativistic effect correction.

Transition State Conformation

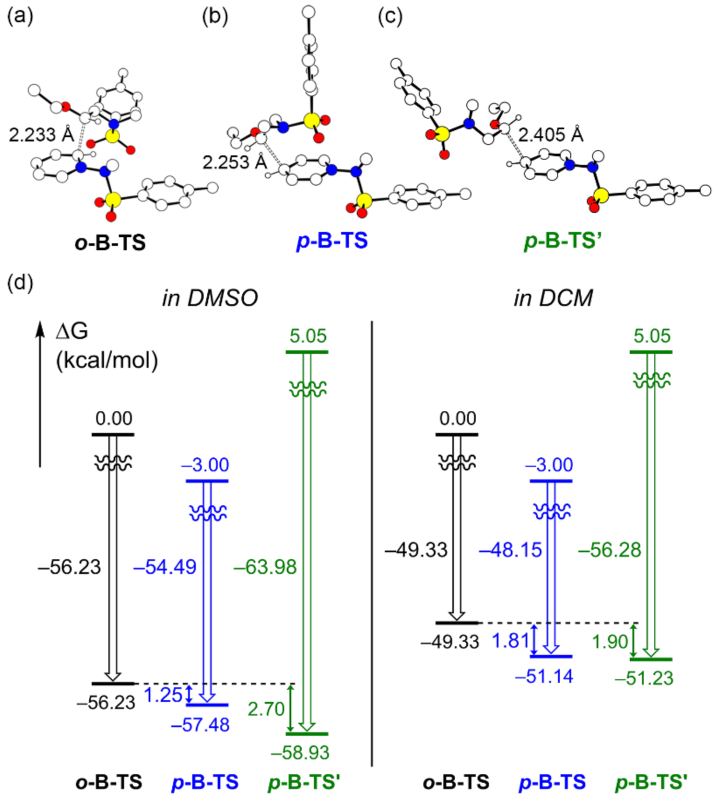

Supplementary Figure 18. Optimized structure of (a) ***o*-B-TS**, (b) ***p*-B-TS**, and (c) ***p*-B-TS'**. Unimportant hydrogen atoms are omitted for clarity. (d) Relative Gibbs free energies of ***o*-B-TS**, ***p*-B-TS** and ***p*-B-TS'**. Numeric at the tail, body, and head of arrows indicates gas-phase free energies (ΔG(gas)), solvation free energies ($\Delta\Delta G^{solv}$) and solution phase free energies (ΔG(sol)), respectively. Solvation free energies were calculated with Poisson-Boltzmann solver using dielectric constants of 46.48 and 9.08 for DMSO and DCM, respectively.

In order to find the genuine origin of regioselectivity, we firstly sought for the most plausible transition state structure of radical addition step. After an extensive trial, we found that the structure of ***o*-B-TS** is the lowest energy conformer of *ortho*-addition. However, we found two conformationally distinguishable and meaningful transition state structure for para-addition, named ***p*-B-TS** and ***p*-B-TS'**. In the main text, we adopted ***p*-B-TS** structure and did not considered ***p*-B-TS'**. In fact, our calculation results showed that the ΔG(sol) of ***p*-B-TS'** is 1.5 kcal/mol lower than ***p*-B-TS**. Nevertheless, the reason we discarded ***p*-B-TS'** as an improper structure is that ***p*-B-TS'** is the worst candidate in the viewpoint of ΔG(gas), which has 8.1 kcal/mol higher than ***p*-B-TS**, but the energy lowering was totally from the solvation advantage as illustrated in Supplementary Figure 18. This high solvation free energy of ***p*-B-TS'** is, of course, natural and easy to understand, because ***p*-B-TS'** has the largest surface area, thus has the biggest dipole moment (7.44, 13.78 D for ***p*-B-TS** and ***p*-B-TS'**, respectively). In this case, we have to very carefully interpret these calculation results. All three structures that we are comparing are calculated as transition states, meaning real molecules *in vitro* just pass by these states in a very short time and don't give any chance for the solvent to reorganize the solvent shell and maximize the stabilization effect. Therefore, the different solvation effect is hard to be fully applicable in this situation, and it is better to conclude that molecules follow intrinsically favorable free energy

trajectory with ***p*-B-TS**, which has the lowest ΔG(gas). To verify our theoretical reasoning, we conducted control experiments for the solvation effect. Originally, the experimental result showed **3b** has C4:C2 selectivity of 10:1 in DMSO. We changed the solvent to less polar dichloromethane (DCM). If the solvent effect is critical on the transition state, the solvation advantage on ***p*-B-TS'** will be reduced in DCM and the C4 product ratio should be decreased. In contrast, if radical attacks C4-site with ***p*-B-TS** fashion, the C4 product ratio should be increased as it endures less solvation penalty under DCM solvent, as described in Supplementary Figure 18d. We obtained the C4:C2 ratio in DCM condition which was increased to 38:1, definitely supporting that the radical **B** approaches **2a** with gas-phase energetically favorable ***p*-B-TS**, rather than sterically advantageous ***p*-B-TS'**. This result also clearly shows that the regioselectivity is not controlled by the steric effect of the N-methyl tosyl group.

Non-orbital Interaction Analysis of *o*-B-TS and *p*-B-TS

In the main text, we mentioned that the interaction energies between the radical fragment and the pyridinium fragment on ***o*-B-TS** and ***p*-B-TS** are 2.9 kcal/mol different although the lengths of the C–C bonds that are being formed are almost identical at 2.23 and 2.25 Å as shown in Supplementary Figure 18a. Therefore, the covalent energy contribution for the interaction energies should be nearly identical and the difference of 2.9 kcal/mol must be originated from the non-covalent interaction. In order to verify our hypothesis and obtain information of orbital / non-orbital interaction between pyridinium and alkyl radical, bonding energy decomposition analysis was conducted. In good agreement with distortion-interaction analysis result, total bonding energy of ***o*-B-TS** and ***p*-B-TS** was –16.69, –19.02 kcal/mol, respectively. Next, we dug deeper into the total bonding energy difference and found the orbital interactions give stabilization of –43.6 and –40.8 kcal/mol and total non-orbital interactions (ΔE^0) give destabilization of 26.9 and 21.7 kcal/mol for ***o*-B-TS** and ***p*-B-TS**, respectively. This result clearly claims that the regioselectivity is determined by $\Delta\Delta E^0$ of –5.2 kcal/mol, which includes electrostatic interaction term, overwhelms *ortho*-preferred orbital-interaction energy difference of 2.9 kcal/mol. So the total bonding energies, which is a sum of the orbital interaction energy and ΔE^0, were –16.7 and –19.0 kcal/mol, which are the major portion of the interaction energy term in ***o*-B-TS** and ***p*-B-TS**. This ΔE^0 difference is intuitively understandable with the atomic charges from electrostatic potential. Some notable charges are illustrated in Supplementary Figure 4b. As mentioned, **B** approaches to **2a** in N1–O1 overlapping fashion in ***p*-B-TS** (Supplementary Figure 4c). In this transition state, N1 has a positive charge of +0.77 and O1 has a contradiction of –0.45 in the atomic unit, with the close-enough N1–O1 distance of 2.98 Å. Thus ***p*-B-TS** could be electrostatically stabilized, while ***o*-B-TS** could not take this advantage due to geometrically apart N1 and O1. In sum, the regioselectivity of the reaction is fundamentally determined by the non-orbital interaction between positively charged nitrogen and the electronegative oxygen, and the well-designed substrates guide the radical to approach C4 site to ultimately engenders *para*-product.

Orbital Interaction Energy Analysis of *o*-B-TS and *p*-B-TS based on PMO Theory

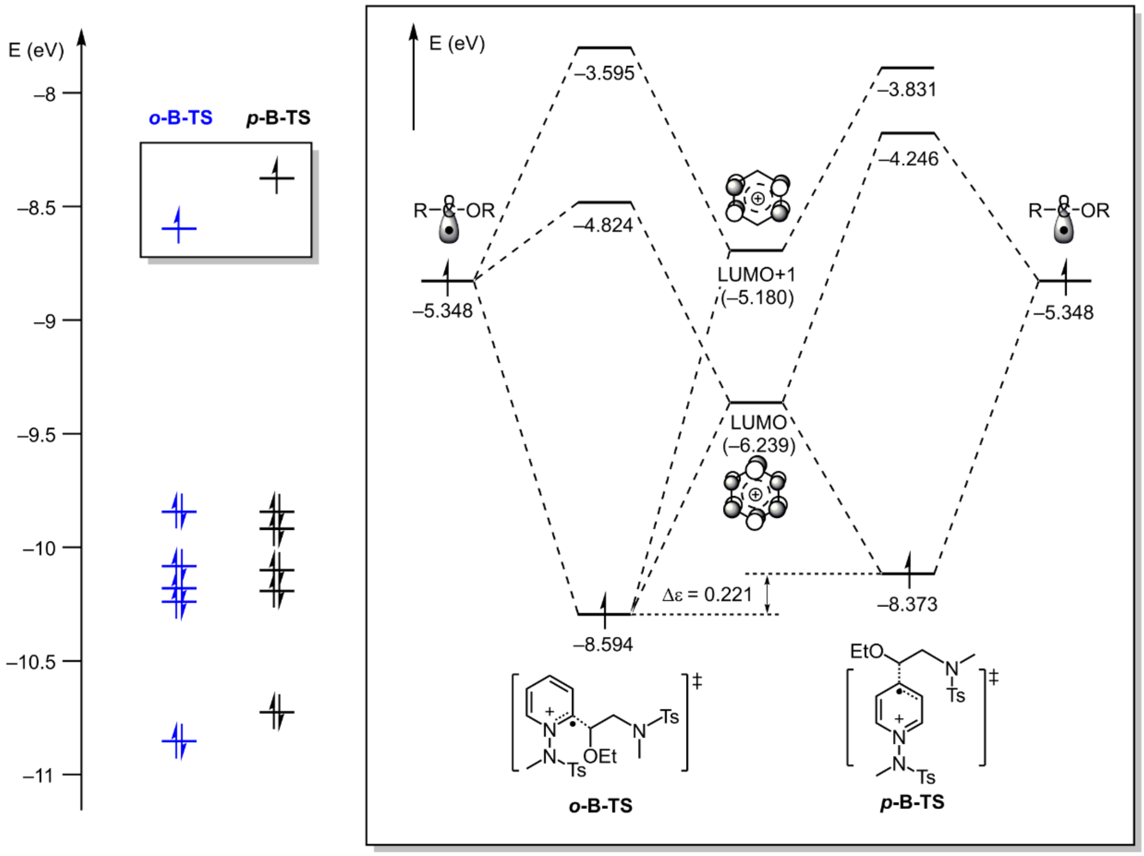

Supplementary Figure 19. Orbital energy levels and PMO diagram of ***o*-B-TS** and ***p*-B-TS**.

In order to understand the *ortho*-preferred orbital interaction between pyridinium and alkyl radical, perturbation molecular orbital (PMO) theory was adopted (Supplementary Figure 19). Basically, the SOMO of alkyl radical **B** is at an ordinary energy level, –5.35 eV. Due to the positively charged environment, whole orbital energies of pyridinium **1b** are significantly decreased compared to ordinary pyridine. More detailed, energies of LUMO and LUMO+1 are pulled down to –6.24 and –5.18 eV respectively, nearby **B** SOMO. In this situation, the energy of **B** SOMO lies between **1b** LUMO and LUMO+1 and could interact with both of them. In general, pyridinium is likely attacked on its C4 site due to the fact that C4 p orbital is the major component of the LUMO. This high contribution of C4 p orbital maximizes the orbital interaction and electron stabilization with the orbital of the approaching nucleophile. However, in this case, the energy difference between **B** SOMO and **1b** LUMO+1 is comparably very small with the difference of 0.17 eV, while the energy gap between **B** SOMO and **1b** LUMO+1 is 0.89 eV, so we cannot ignore the interaction with LUMO+1 as it is a promising orbital for stabilization. Therefore, 3-center-1-electron interaction occurs on ***o*-B-TS**, enhancing C2 component on the bonding orbital, while LUMO+1 doesn’t intervene on ***p*-B-TS** because it has no C4 contribution. As a result, frontier molecular orbital energy is more stabilized by –0.22 eV in ***o*-B-TS**.

Compound Characterizations:

Characterization of representative example of N-tosyl 1-aminopyridinium tetrafluoroborate salts

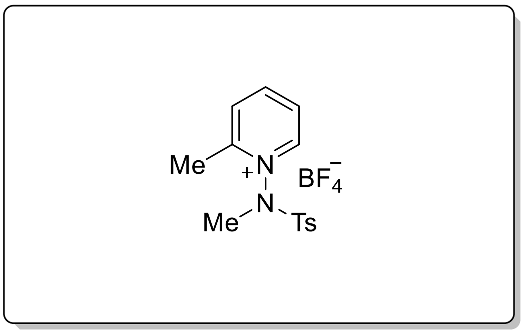

1-((N,4-dimethylphenyl)sulfonamido)-2-methylpyridin-1-ium tetrafluoroborate (2b). White solid. ^{1}H NMR (600 MHz, CD_2Cl_2) δ 8.49 (td, J = 7.8, 1.3 Hz, 1H), 8.06 (dd, J = 6.6, 1.4 Hz, 1H), 8.02 (dd, J = 8.1, 1.8 Hz, 1H), 7.89 (td, J = 7.1, 6.5, 1.8 Hz, 1H), 7.64 (d, J = 8.4 Hz, 2H), 7.51 (d, J = 8.0 Hz, 2H), 3.49 (s, 3H), 2.95 (s, 3H), 2.53 (s, 3H).^{13}C NMR (150 MHz, CD_2Cl_2) δ 161.5, 149.0, 148.5, 144.0, 131.8, 131.6, 129.7, 129.3, 127.9, 40.3, 22.2, 20.9. HRMS (ESI^{+}) m/z calcd. $C_{14}H_{17}N_2O_2S^{+}$ [M-BF_4^{-}]$^{+}$: 277.1005, found: 277.0996.

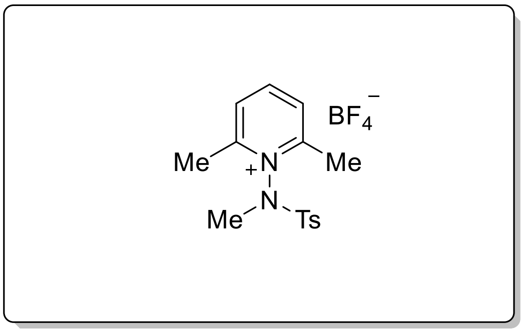

1-((N,4-dimethylphenyl)sulfonamido)-2,6-dimethylpyridin-1-ium tetrafluoroborate (2c). Pale yellow solid. ^{1}H NMR (600 MHz, CD_2Cl_2) δ 8.33 (t, J = 7.9 Hz, 1H), 7.80 (d, J = 7.9 Hz, 2H), 7.75 (d, J = 8.4 Hz, 2H), 7.49 (d, J = 8.1 Hz, 2H), 3.60 (s, 3H), 2.66 (s, 6H), 2.50 (s, 3H). ^{13}C NMR (150 MHz, CD_2Cl_2) δ 161.3, 147.8, 147.6, 134.6, 131.5, 129.3, 128.2, 38.6, 22.1, 21.0. HRMS (ESI^{+}) m/z calcd. $C_{15}H_{19}N_2O_2S^{+}$ [M-BF_4^{-}]$^{+}$: 291.1162, found: 291.1165.

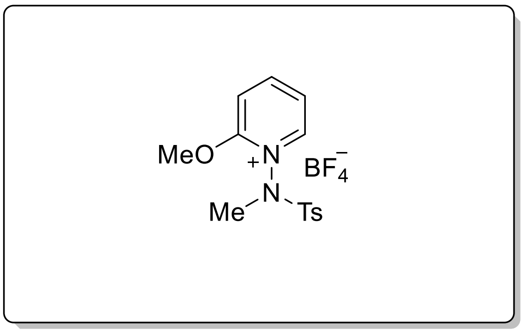

1-((N,4-dimethylphenyl)sulfonamido)-2-methoxypyridin-1-ium tetrafluoroborate (2d). White solid. ^{1}H NMR (600 MHz, CD_2Cl_2, MeOD cosolvent) δ 8.56 (t, J = 8.2 Hz, 1H), 8.31 (d, J = 6.6 Hz, 1H), 7.66 (d, J = 8.0 Hz, 2H), 7.61 (d, J = 9.0 Hz, 1H), 7.54 (t, J = 7.1 Hz, 1H), 7.46 (d, J = 8.0 Hz, 2H), 3.96 (s, 3H), 3.48 (s, 3H), 2.48 (s, 3H). ^{13}C NMR (150 MHz, CD_2Cl_2, MeOD, cosolvent) δ 162.6, 151.8, 147.7, 145.6, 133.1, 131.2, 128.9, 120.2, 113.4, 60.6, 39.0, 21.9. HRMS (ESI$^+$) m/z calcd. $C_{14}H_{17}N_2O_3S^+$ [M-BF_4^-]$^+$: 293.0954, found: 293.0960.

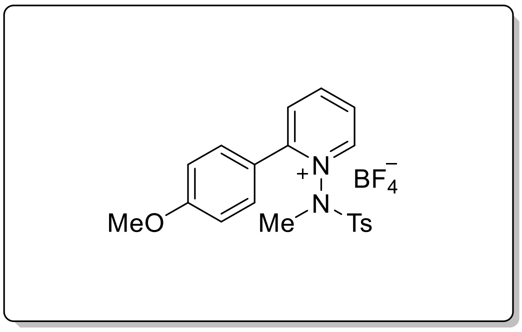

1-((N,4-dimethylphenyl)sulfonamido)-2-(4-methoxyphenyl)pyridin-1-ium tetrafluoroborate (2e). White solid. ^{1}H NMR (600 MHz, CD_2Cl_2) δ 8.61 (t, J = 7.8 Hz, 1H), 8.50 (d, J = 6.5 Hz, 1H), 8.09 – 8.02 (m, 2H), 7.49 (d, J = 8.5 Hz, 2H), 7.32 (d, J = 8.0 Hz, 2H), 7.27 (d, J = 7.9 Hz, 2H), 7.08 (d, J = 8.5 Hz, 2H), 3.93 (s, 3H), 3.55 (s, 3H), 2.47 (s, 3H). ^{13}C NMR (150 MHz, CD_2Cl_2) δ 163.3, 160.1, 148.6, 148.5, 144.8, 132.2, 131.6, 131.2, 129.8, 129.5, 128.0, 122.8, 115.2, 56.3, 40.2, 22.1. HRMS (ESI$^+$) m/z calcd. $C_{20}H_{21}N_2O_3S^+$ [M-BF_4^-]$^+$: 369.1267, found: 369.1272.

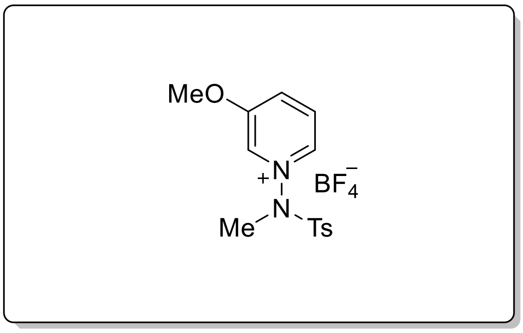

1-((N,4-dimethylphenyl)sulfonamido)-3-methoxypyridin-1-ium tetrafluoroborate (2f). White solid. ^{1}H NMR (600 MHz, CD_2Cl_2, MeOD cosolvent) δ 8.21 – 8.17 (m, 2H), 8.13 (d, J = 6.3 Hz, 1H), 8.01 (dd, J = 9.6, 6.2 Hz, 1H), 7.60 (d, J = 8.2 Hz, 2H), 7.48 (d, J = 8.0 Hz, 2H), 4.01 (s, 3H), 3.54 (s, 3H), 2.51 (s, 3H). ^{13}C NMR (150 MHz, CD_2Cl_2, MeOD cosolvent) δ 160.1, 149.1, 137.8, 134.6, 133.5, 131.7, 129.8, 129.8, 127.9, 58.5, 40.2, 22.2. HRMS (ESI$^+$) m/z calcd. $C_{14}H_{17}N_2O_3S^+$ [M-BF_4^-]$^+$: 293.0954, found: 293.0953.

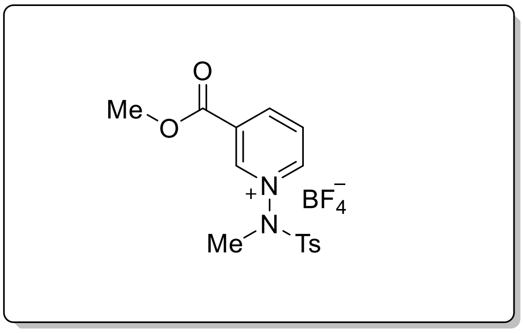

1-((N,4-dimethylphenyl)sulfonamido)-3-(methoxycarbonyl)pyridin-1-ium tetrafluoroborate (2g). White solid. ^{1}H NMR (600 MHz, CD_2Cl_2) δ 9.11 (d, J = 8.1 Hz, 1H), 9.01 (s, 1H), 8.64 (d, J = 6.4 Hz, 1H), 8.25 (dd, J = 7.8, 6.6 Hz, 1H), 7.56 (d, J = 8.4 Hz, 2H), 7.49 (d, J = 8.1 Hz, 2H), 4.04 (s, 3H), 3.56 (s, 3H), 2.52 (s, 3H). ^{13}C NMR (150 MHz, CD_2Cl_2) δ 161.1, 149.5, 149.0, 148.3, 147.3, 132.5, 131.8, 130.6, 129.8, 127.4, 54.7, 40.0, 22.2. HRMS (ESI$^+$) m/z calcd. $C_{15}H_{17}N_2O_4S^+$ [M-BF_4^-]$^+$: 321.0904, found: 321.0905.

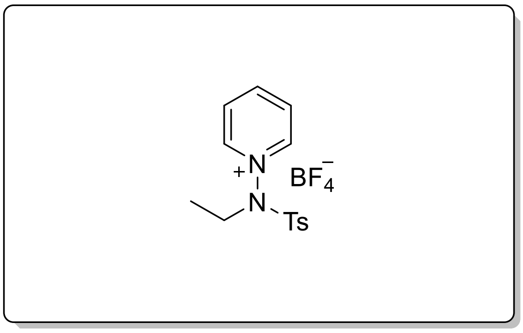

1-((N-ethyl-4-methylphenyl)sulfonamido)pyridin-1-ium tetrafluoroborate (2h). White solid. ^{1}H NMR (600 MHz, CD_2Cl_2) δ 8.74 (t, J = 7.8 Hz, 1H), 8.57 (d, J = 6.2 Hz, 2H), 8.21 (t, J = 7.1 Hz, 2H), 7.57 (d, J = 8.0 Hz, 2H), 7.49 (d, J = 8.1 Hz, 2H), 3.95 (q, J = 7.1 Hz, 2H), 2.52 (s, 3H), 1.15 (t, J = 7.1 Hz, 3H).^{13}C NMR (150 MHz, CD_2Cl_2) δ 149.7, 149.3, 146.8, 131.8, 130.4, 129.4, 128.6, 48.2, 22.2, 12.4. HRMS (ESI$^+$) m/z calcd. $C_{14}H_{17}N_2O_2S^+$ [M-BF_4^-]$^+$: 277.1005, found: 277.0996.

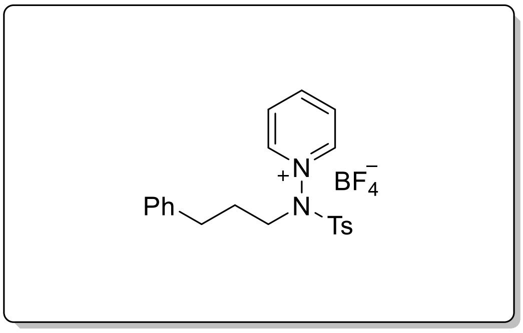

1-((4-methyl-N-(3-phenylpropyl)phenyl)sulfonamido)pyridin-1-ium tetrafluoroborate (2i). Brown solid. ^{1}H NMR (600 MHz, CD_2Cl_2) δ 8.68 (t, J = 7.8 Hz, 1H), 8.41 (d, J = 6.1 Hz, 2H), 8.13 (t, J = 7.1 Hz, 2H), 7.53 (d, J = 8.1 Hz, 2H), 7.47 (d, J = 8.0 Hz, 2H), 7.30 (t, J = 7.4 Hz, 2H), 7.23 (t, J = 7.3 Hz, 1H), 7.19 (d, J = 7.4 Hz, 2H), 3.92 (t, J = 7.0 Hz, 2H), 2.71 (t, J = 7.5 Hz, 2H), 2.51 (s, 3H), 1.76 (p, J = 7.3 Hz, 2H). ^{13}C NMR (150 MHz, CD_2Cl_2) δ

149.9, 149.1, 146.6, 141.8, 131.8, 130.4, 129.5, 128.8, 128.6, 128.1, 125.9, 52.4, 32.6, 28.6, 22.2. HRMS (ESI$^+$) m/z calcd. $C_{21}H_{23}N_2O_2S^+$ [M-BF_4^-]$^+$: 367.1475, found: 367.1476.

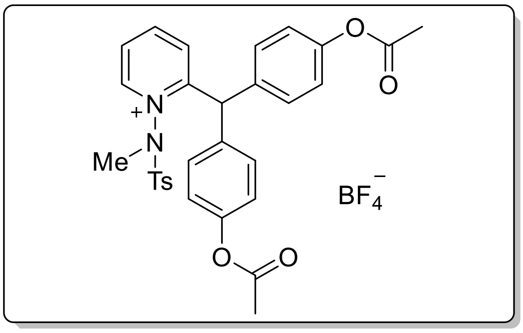

2-(bis(4-acetoxyphenyl)methyl)-1-((N,4-dimethylphenyl)sulfonamido)pyridin-1-ium tetrafluoroborate (2j). Yellow solid. ^{1}H NMR (600 MHz, CD_2Cl_2) δ 8.55 (t, J = 7.9 Hz, 1H), 8.16 (d, J = 6.4 Hz, 1H), 8.08 (t, J = 7.0 Hz, 1H), 7.67 (d, J = 8.2 Hz, 1H), 7.64 (d, J = 7.0 Hz, 2H), 7.55 (d, J = 8.0 Hz, 2H), 7.26 (d, J = 8.2 Hz, 2H), 7.22 – 7.17 (m, 4H), 7.13 (d, J = 8.2 Hz, 2H), 6.68 (s, 1H), 2.85 (s, 3H), 2.55 (s, 3H), 2.30 (s, 3H), 2.27 (s, 3H). ^{13}C NMR (150 MHz, CD_2Cl_2) δ 169.7, 169.6, 164.4, 151.7, 151.4, 149.7, 149.3, 146.1, 134.8, 134.4, 132.1, 131.1, 131.0, 130.7, 129.8, 129.4, 128.4, 123.8, 123.4, 52.9, 40.2, 22.3, 21.5, 21.4. HRMS (ESI$^+$) m/z calcd. $C_{30}H_{29}N_2O_6S^+$ [M-BF_4^-]$^+$: 545.1741, found: 545.1749.

Characterization of aminoethyl pyridine products

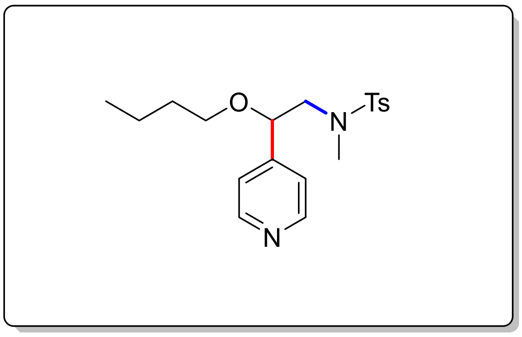

N-(2-butoxy-2-(pyridin-4-yl)ethyl)-N,4-dimethylbenzenesulfonamide (3a). Yield 84%. 30.5 mg. Colorless oil. ^{1}H NMR (600 MHz, CD_2Cl_2) δ 8.57 (d, J = 5.2 Hz, 2H), 7.62 (d, J = 8.1 Hz, 2H), 7.32 (d, J = 7.9 Hz, 2H), 7.26 (d, J = 5.6 Hz, 2H), 4.50 (dd, J = 8.2, 4.1 Hz, 1H), 3.36 – 3.23 (m, 3H), 3.06 (dd, J = 14.4, 8.2 Hz, 1H), 2.82 (s, 3H), 2.41 (s, 3H), 1.56 – 1.47 (m, 2H), 1.40 – 1.30 (m, 2H), 0.89 (t, J = 7.4 Hz, 3H). ^{13}C NMR (150 MHz, CD_2Cl_2) δ 150.6, 149.5, 144.2, 135.8, 130.3, 127.8, 122.3, 81.6, 70.2, 56.7, 37.6, 32.5, 21.8, 19.9, 14.2. HRMS (EI$^+$) m/z calcd. $C_{19}H_{26}N_2O_3S^+$ [M]$^+$: 362.1664, found: 362.1668.

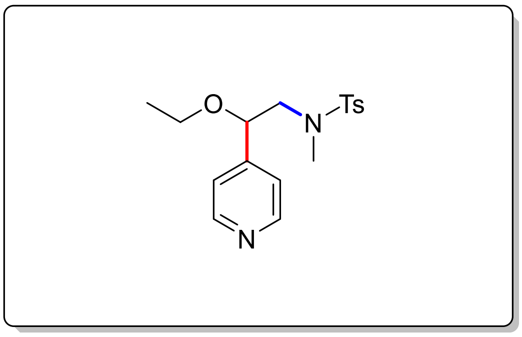

N-(2-ethoxy-2-(pyridin-4-yl)ethyl)-N,4-dimethylbenzenesulfonamide (3b). Yield 75%. 25.0 mg. Colorless oil. ^{1}H NMR (600 MHz, CD_2Cl_2) δ 8.56 (d, J = 5.0 Hz, 2H), 7.62 (d, J = 7.9 Hz, 2H), 7.32 (d, J = 7.9 Hz, 2H), 7.26 (d, J = 5.7 Hz, 2H), 4.51 (dd, J = 8.2, 4.3 Hz, 1H), 3.43 – 3.34 (m, 2H), 3.23 (dd, J = 14.4, 4.1 Hz, 1H), 3.08 (dd, J = 14.4, 8.0 Hz, 1H), 2.81 (s, 3H), 2.41 (s, 3H), 1.16 (t, J = 7.0 Hz, 3H). ^{13}C NMR (150 MHz, CD_2Cl_2) δ 150.6, 149.5, 144.2, 135.7, 130.3, 127.8, 122.3, 81.3, 65.8, 56.7, 37.6, 21.8, 15.6. HRMS (ESI^+) m/z calcd. $C_{17}H_{22}N_2O_3S^+$ $[M]^+$: 334.1351, found: 334.1353.

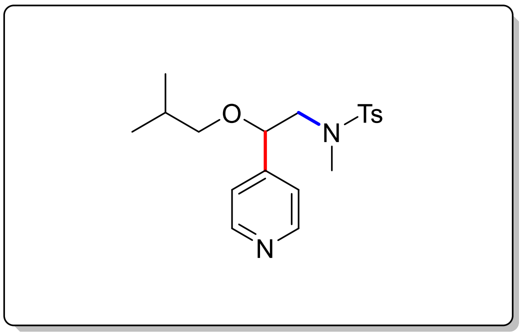

N-(2-isobutoxy-2-(pyridin-4-yl)ethyl)-N,4-dimethylbenzenesulfonamide (3c). Yield 84%. 30.4 mg. Colorless oil. ^{1}H NMR (600 MHz, CD_2Cl_2) δ 8.56 (d, J = 6.0 Hz, 2H), 7.62 (d, J = 8.4 Hz, 2H), 7.32 (d, J = 8.0 Hz, 2H), 7.26 (d, J = 6.1 Hz, 2H), 4.50 (dd, J = 8.2, 4.0 Hz, 1H), 3.28 (dd, J = 14.4, 4.0 Hz, 1H), 3.12 – 3.02 (m, 3H), 2.83 (s, 3H), 2.41 (s, 3H), 1.88 – 1.77 (m, 1H), 0.89 (t, J = 6.6 Hz, 6H). ^{13}C NMR (150 MHz, CD_2Cl_2) δ 150.5, 149.5, 144.2, 135.7, 130.3, 127.8, 122.3, 81.8, 77.1, 56.7, 37.7, 29.3, 21.8, 19.7, 19.6. HRMS (EI^+) m/z calcd. $C_{19}H_{26}N_2O_3S^+$ $[M]^+$: 362.1664, found: 362.1666.

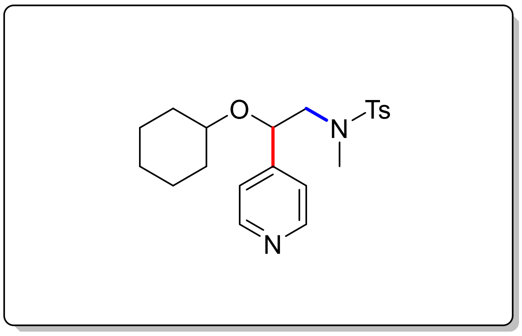

N-(2-(cyclohexyloxy)-2-(pyridin-4-yl)ethyl)-N,4-dimethylbenzenesulfonamide (3d). Yield 90%. 35.0 mg. White solid. mp 86-88 °C. ^{1}H NMR (600 MHz, CD_2Cl_2) δ 8.55 (d, J = 6.0 Hz, 2H), 7.62 (d, J = 8.3 Hz, 2H), 7.32 (d, J = 8.0 Hz, 2H), 7.29 (d, J = 6.0 Hz, 2H), 4.69 (dd, J = 8.3, 4.0 Hz, 1H), 3.26 – 3.15 (m, 2H), 3.01 (dd, J = 14.4, 8.3 Hz, 1H),

2.83 (s, 3H), 2.41 (s, 3H), 1.92 – 1.85 (m, 1H), 1.74 – 1.67 (m, 2H), 1.66 – 1.61 (m, 1H), 1.50 – 1.43 (m, 1H), 1.34 – 1.23 (m, 2H), 1.23 – 1.11 (m, 3H). ^{13}C NMR (150 MHz, CD_2Cl_2) δ 150.4, 150.4, 144.1, 135.8, 130.3, 127.8, 122.3, 78.6, 77.2, 57.1, 37.8, 33.9, 32.0, 26.2, 24.6, 24.4, 21.8. HRMS (EI^+) m/z calcd. $C_{21}H_{28}N_2O_3S^+$ $[M]^+$: 388.1821, found: 388.5260.

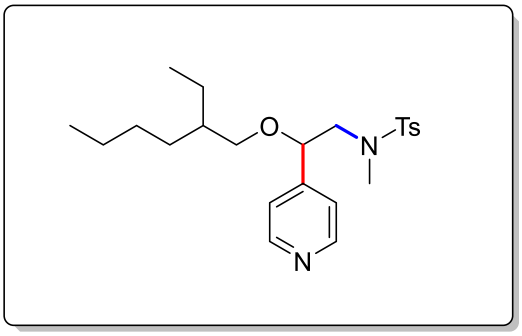

N-(2-((2-ethylhexyl)oxy)-2-(pyridin-4-yl)ethyl)-N,4-dimethylbenzenesulfonamide (3e). Diastereomeric mixture (1:1) Yield 79%. 33.1 mg. Pale yellow oil. ^{1}H NMR (600 MHz, CD_2Cl_2) δ 8.57 (d, J = 6.0 Hz, 2H), 7.62 (d, J = 8.3 Hz, 2H), 7.32 (d, J = 8.0 Hz, 2H), 7.25 (d, J = 6.0 Hz, 2H), 4.48 (dzd, J = 8.2, 4.0 Hz, 1H), 3.28 (dd, J = 14.4, 4.0 Hz, 1H), 3.24 – 3.18 (m, 2H), 3.03 (ddd, J = 14.4, 8.2, 1.9 Hz, 1H), 2.83 (s, 3H), 2.41 (s, 3H), 1.50 – 1.16 (m, 9H), 0.91 – 0.80 (m, 6H). ^{13}C NMR (150 MHz, CD_2Cl_2) δ 150.6, 149.5, 144.2, 135.7, 130.3, 127.8, 122.4, 122.3, 82.1, 82.1, 73.0, 72.9, 56.7, 40.6, 40.6, 37.8, 31.0, 31.0, 29.7, 29.5, 24.4, 24.4, 23.6, 21.8, 14.4, 11.5, 11.4. HRMS (EI^+) m/z calcd. $C_{23}H_{34}N_2O_3S^+$ $[M]^+$: 418.2290, found: 418.2288.

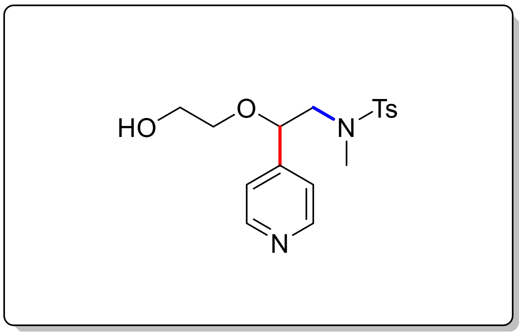

N-(2-(2-hydroxyethoxy)-2-(pyridin-4-yl)ethyl)-N,4-dimethylbenzenesulfonamide (3f). Yield 93%. 32.6 mg. Pale yellow oil. ^{1}H NMR (600 MHz, CD_2Cl_2) δ 8.56 (d, J = 6.1 Hz, 2H), 7.63 (d, J = 8.3 Hz, 2H), 7.32 (d, J = 8.0 Hz, 2H), 7.29 (d, J = 6.1 Hz, 2H), 4.59 (dd, J = 8.3, 4.2 Hz, 1H), 3.70 (t, J = 4.6 Hz, 2H), 3.51 (dt, J = 10.2, 4.4 Hz, 1H), 3.44 (dt, J = 9.9, 4.8 Hz, 1H), 3.25 (dd, J = 14.5, 8.3 Hz, 1H), 3.18 (dd, J = 14.5, 4.2 Hz, 1H), 2.79 (s, 3H), 2.77 (br, 1H), 2.41 (s, 3H). ^{13}C NMR (150 MHz, CD_2Cl_2) δ 150.5, 149.0, 144.3, 135.5, 130.3, 127.8, 122.3, 81.1, 72.0, 62.2, 56.6, 37.2, 21.8. HRMS (EI^+) m/z calcd. $C_{17}H_{22}N_2O_4S^+$ $[M]^+$: 350.1300, found: 350.1303.

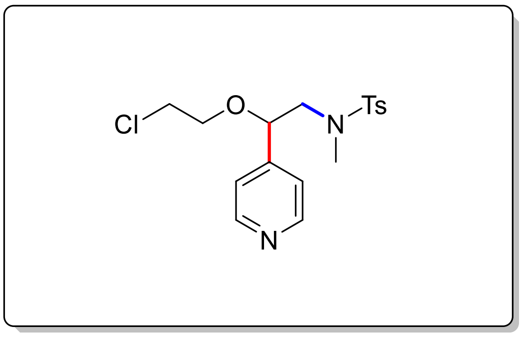

N-(2-(2-chloroethoxy)-2-(pyridin-4-yl)ethyl)-N,4-dimethylbenzenesulfonamide (3g). Yield 83%. 30.6 mg. Colorless oil. ^{1}H NMR (600 MHz, CD_2Cl_2) δ 8.58 (d, J = 6.0 Hz, 2H), 7.63 (d, J = 8.1 Hz, 2H), 7.32 (d, J = 8.0 Hz, 2H), 7.29 (d, J = 6.0 Hz, 2H), 4.61 (dd, J = 8.2, 4.0 Hz, 1H), 3.65 – 3.58 (m, 4H), 3.29 (dd, J = 14.5, 4.1 Hz, 1H), 3.10 (dd, J = 14.6, 8.2 Hz, 1H), 2.84 (s, 3H), 2.41 (s, 3H). ^{13}C NMR (150 MHz, CD_2Cl_2) δ 150.7, 148.4, 144.3, 135.6, 130.3, 127.8, 122.2, 82.1, 70.4, 56.7, 43.7, 37.8, 21.8. HRMS (EI$^+$) m/z calcd. $C_{17}H_{21}ClN_2O_3S^+$ [M]$^+$: 368.0961, found: 368.0963.

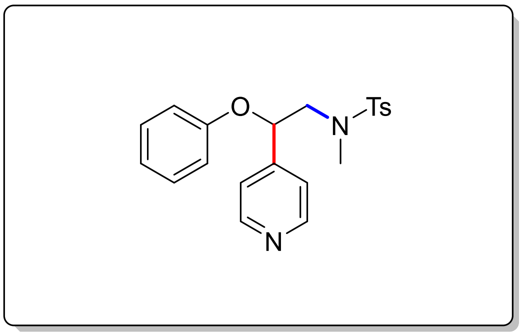

N,4-dimethyl-N-(2-phenoxy-2-(pyridin-4-yl)ethyl)benzenesulfonamide (3h). Yield 68% 26.0 mg. Colorless gum. ^{1}H NMR (600 MHz, CD_2Cl_2) δ 8.57 (d, J = 5.2 Hz, 2H), 7.64 (d, J = 8.3 Hz, 2H), 7.34 (d, J = 6.0 Hz, 2H), 7.30 (d, J = 8.0 Hz, 2H), 7.21 (t, J = 8.0 Hz, 2H), 6.93 (t, J = 7.4 Hz, 1H), 6.80 (d, J = 7.6 Hz, 2H), 5.40 (dd, J = 8.1, 4.1 Hz, 1H), 3.47 (dd, J = 14.7, 4.1 Hz, 1H), 3.36 (dd, J = 14.7, 8.1 Hz, 1H), 2.87 (s, 3H), 2.40 (s, 3H). ^{13}C NMR (150 MHz, CD_2Cl_2) δ 157.8, 150.8, 148.3, 144.4, 135.6, 130.4, 130.1, 127.7, 122.2, 121.8, 116.3, 79.5, 56.8, 37.9, 21.8. HRMS (EI$^+$) m/z calcd. $C_{21}H_{22}N_2O_3S^+$ [M]$^+$: 382.1351, found: 382.1351.

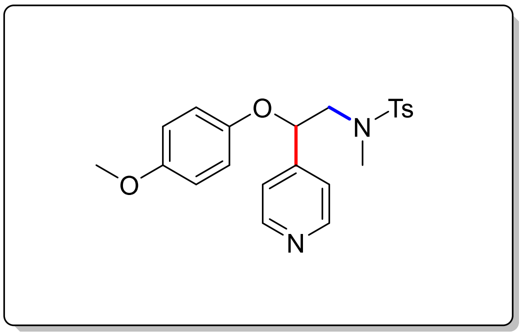

N-(2-(4-methoxyphenoxy)-2-(pyridin-4-yl)ethyl)-N,4-dimethylbenzenesulfonamide (3i). Yield 74%. 30.5 mg. Pale yellow oil. ^{1}H NMR (600 MHz, CD_2Cl_2) δ 8.57 (d, J = 6.2 Hz, 2H), 7.64 (d, J = 8.3 Hz, 2H), 7.33 (d, J = 6.0 Hz, 2H), 7.31 (d, J = 8.0 Hz, 2H), 6.74 (s, 4H), 5.29 (dd, J = 8.1, 4.1 Hz, 1H), 3.70 (s, 3H), 3.44 (dd, J = 14.7, 4.2 Hz,

1H), 3.34 (dd, J = 14.6, 8.1 Hz, 1H), 2.87 (s, 3H), 2.40 (s, 3H). ^{13}C NMR (150 MHz, CD_2Cl_2) δ 155.1, 151.8, 150.7, 148.4, 144.3, 135.5, 130.3, 127.7, 121.9, 117.4, 115.1, 80.4, 56.8, 56.1, 37.8, 21.8. HRMS (EI$^+$) m/z calcd. $C_{22}H_{24}N_2O_4S^+$ [M]$^+$: 412.1457, found: 412.1455.

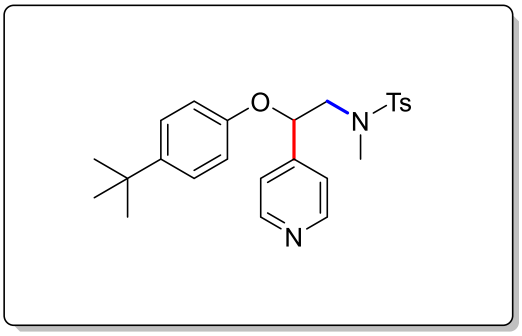

N-(2-(4-(tert-butyl)phenoxy)-2-(pyridin-4-yl)ethyl)-N,4-dimethylbenzenesulfonamide (3j). Yield 62%. 27.2 mg. Colorless gum. ^{1}H NMR (600 MHz, CD_2Cl_2) δ 8.57 (d, J = 6.1 Hz, 2H), 7.64 (d, J = 8.3 Hz, 2H), 7.33 (d, J = 6.1 Hz, 2H), 7.30 (d, J = 7.9 Hz, 2H), 7.22 (d, J = 8.8 Hz, 2H), 6.73 (d, J = 8.9 Hz, 2H), 5.36 (dd, J = 8.2, 4.1 Hz, 1H), 3.46 (dd, J = 14.7, 4.1 Hz, 1H), 3.33 (dd, J = 14.7, 8.1 Hz, 1H), 2.87 (s, 3H), 2.40 (s, 3H), 1.25 (s, 9H). ^{13}C NMR (150 MHz, CD_2Cl_2) δ 155.5, 150.8, 148.4, 145.0, 144.3, 135.6, 130.3, 127.8, 126.9, 121.8, 115.6, 79.6, 56.8, 37.8, 34.5, 31.7, 21.8. HRMS (EI$^+$) m/z calcd. $C_{25}H_{30}N_2O_3S^+$ [M]$^+$: 438.1977, found: 438.1981.

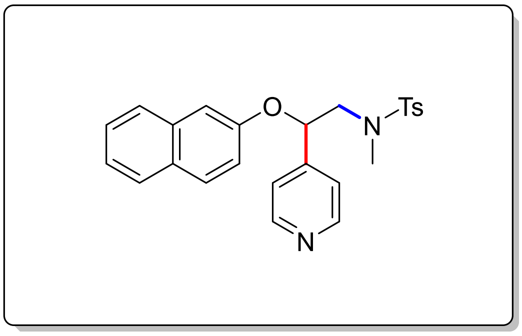

N,4-dimethyl-N-(2-(naphthalen-2-yloxy)-2-(pyridin-4-yl)ethyl)benzenesulfonamide (3k). Yield 57%. 24.6 mg. Colorless gum. ^{1}H NMR (600 MHz, CD_2Cl_2) δ 8.59 (d, J = 4.3 Hz, 2H), 7.77 – 7.72 (m, 2H), 7.65 (d, J = 7.9 Hz, 2H), 7.59 (d, J = 8.2 Hz, 1H), 7.42 – 7.37 (m, 3H), 7.33 (t, J = 7.5 Hz, 1H), 7.27 (d, J = 7.8 Hz, 2H), 7.15 (d, J = 8.8 Hz, 1H), 6.94 (s, 1H), 5.55 (dd, J = 8.6, 4.0 Hz, 1H), 3.54 (dd, J = 14.7, 3.8 Hz, 1H), 3.43 (dd, J = 14.7, 8.1 Hz, 1H), 2.91 (d, J = 1.7 Hz, 3H), 2.33 (s, 3H). ^{13}C NMR (150 MHz, CD_2Cl_2) δ 155.5, 150.8, 148.0, 144.4, 135.6, 134.8, 130.3, 130.2, 129.9, 128.1, 127.7, 127.3, 127.1, 124.7, 121.8, 119.3, 109.6, 79.5, 56.8, 38.0, 21.7. HRMS (EI$^+$) m/z calcd. $C_{25}H_{24}N_2O_3S^+$ [M]$^+$: 432.1508, found: 432.1508.

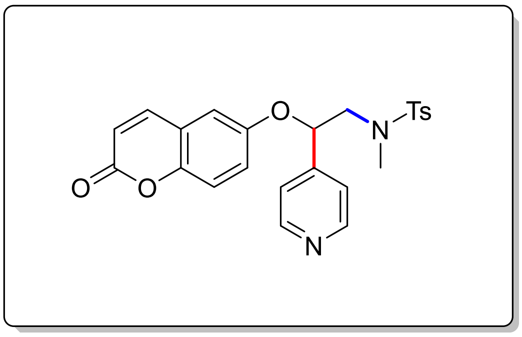

N,4-dimethyl-N-(2-((2-oxo-2H-chromen-6-yl)oxy)-2-(pyridin-4-yl)ethyl)benzenesulfonamide (3l). Yield 36%. 16.2 mg. yellow solid. mp 69-71 °C. ^{1}H NMR (600 MHz, CD_2Cl_2) δ 8.61 (br, 2H), 7.64 (d, J = 8.0 Hz, 2H), 7.56 (d, J = 9.6 Hz, 1H), 7.36 (d, J = 4.9 Hz, 2H), 7.30 (d, J = 8.0 Hz, 2H), 7.20 (d, J = 9.0 Hz, 1H), 7.07 (dd, J = 9.0, 2.9 Hz, 1H), 6.84 (d, J = 2.9 Hz, 1H), 6.35 (d, J = 9.6 Hz, 1H), 5.42 (dd, J = 8.1, 4.1 Hz, 1H), 3.46 (dd, J = 14.8, 4.1 Hz, 1H), 3.37 (dd, J = 14.8, 8.1 Hz, 1H), 2.87 (s, 3H), 2.39 (s, 3H). ^{13}C NMR (150 MHz, CD_2Cl_2) δ 160.9, 154.1, 150.9, 149.6, 147.5, 144.5, 143.4, 135.4, 130.4, 127.7, 121.9, 121.1, 119.9, 118.4, 117.8, 113.4, 80.6, 56.8, 38.1, 21.8. HRMS (EI^+) m/z calcd. $C_{24}H_{22}N_2O_5S^+$ $[M]^+$: 450.1249, found: 450.1252.

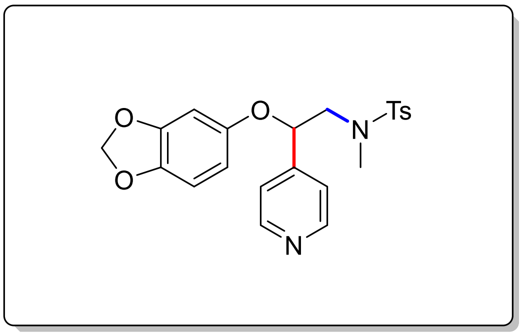

N-(2-(benzo[d][1,3]dioxol-5-yloxy)-2-(pyridin-4-yl)ethyl)-N,4-dimethylbenzenesulfonamide (3m). Yield 54%. 23.0 mg. Colorless gum. ^{1}H NMR (600 MHz, CD_2Cl_2) δ 8.57 (d, J = 6.0 Hz, 2H), 7.64 (d, J = 8.3 Hz, 2H), 7.32 – 7.29 (m, 4H), 6.60 (d, J = 8.5 Hz, 1H), 6.39 (d, J = 2.5 Hz, 1H), 6.20 (dd, J = 8.5, 2.6 Hz, 1H), 5.88 (s, 2H), 5.25 (dd, J = 8.2, 4.1 Hz, 1H), 3.42 (dd, J = 14.7, 4.1 Hz, 1H), 3.31 (dd, J = 14.7, 8.2 Hz, 1H), 2.86 (s, 3H), 2.40 (s, 3H). ^{13}C NMR (150 MHz, CD_2Cl_2) δ 153.1, 150.8, 148.9, 148.1, 144.4, 143.0, 135.6, 130.4, 127.7, 121.8, 108.4, 108.1, 102.1, 99.6, 80.7, 56.8, 37.8, 21.8. HRMS (EI^+) m/z calcd. $C_{22}H_{22}N_2O_5S^+$ $[M]^+$: 426.1249, found: 426.1251.

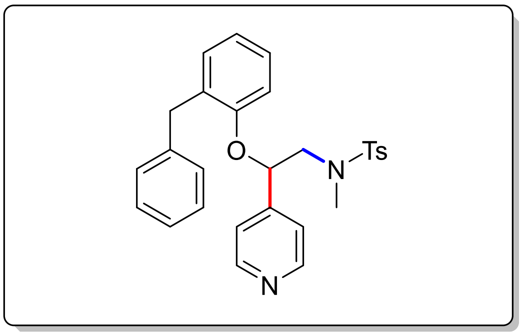

N-(2-(2-benzylphenoxy)-2-(pyridin-4-yl)ethyl)-N,4-dimethylbenzenesulfonamide (3n). 1 mol% Eosin Y was used. Yield 57%. 26.9 mg. Colorless gum. ^{1}H NMR (600 MHz, CD_2Cl_2) δ 8.46 (d, J = 3.8 Hz, 2H), 7.62 (d, J = 8.4

Hz, 2H), 7.31 (d, J = 7.9 Hz, 2H), 7.28 (t, J = 7.7 Hz, 2H), 7.23 – 7.19 (m, 3H), 7.17 (dd, J = 7.5, 1.7 Hz, 1H), 7.04 (t, J = 7.8 Hz, 1H), 7.00 (d, J = 4.5 Hz, 2H), 6.88 (t, J = 7.4 Hz, 1H), 6.57 (d, J = 8.2 Hz, 1H), 5.44 (t, J = 6.1 Hz, 1H), 4.11 (d, J = 15.0 Hz, 1H), 4.02 (d, J = 15.0 Hz, 1H), 3.35 – 3.26 (m, 2H), 2.72 (s, 3H), 2.41 (s, 3H). ^{13}C NMR (150 MHz, CD_2Cl_2) δ 155.2, 150.7, 147.9, 144.4, 141.6, 135.3, 131.7, 130.4, 130.3, 129.4, 128.9, 128.0, 127.8, 126.5, 121.8, 121.7, 113.0, 78.5, 56.8, 37.9, 37.2, 21.8. HRMS (EI$^+$) m/z calcd. $C_{28}H_{28}N_2O_3S^+$ [M]$^+$: 472.1821, found: 472.1820.

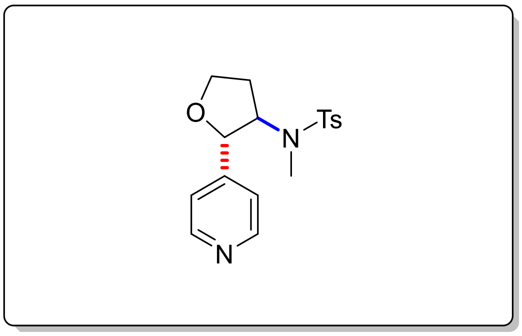

***trans*-N,4-dimethyl-N-(2-(pyridin-4-yl)tetrahydrofuran-3-yl)benzenesulfonamide (3o).** Yield 47%. 15.5 mg. Colorless gum. ^{1}H NMR (600 MHz, CD_2Cl_2) δ 8.51 (d, J = 5.2 Hz, 2H), 7.58 (d, J = 7.9 Hz, 2H), 7.28 (d, J = 7.9 Hz, 2H), 7.24 (d, J = 5.4 Hz, 2H), 4.71 (d, J = 5.9 Hz, 1H), 4.53 – 4.43 (m, 1H), 4.06 – 3.91 (m, 2H), 2.89 (s, 3H), 2.42 (s, 3H), 1.91 – 1.83 (m, 1H), 1.77 – 1.69 (m, 1H). ^{13}C NMR (150 MHz, CD_2Cl_2) δ 150.4, 150.2, 144.5, 136.2, 130.4, 127.7, 121.0, 80.6, 68.5, 65.0, 30.0, 27.6, 21.8. HRMS (EI$^+$) m/z calcd. $C_{17}H_{20}N_2O_3S^+$ [M]$^+$: 332.1195, found: 332.1192.

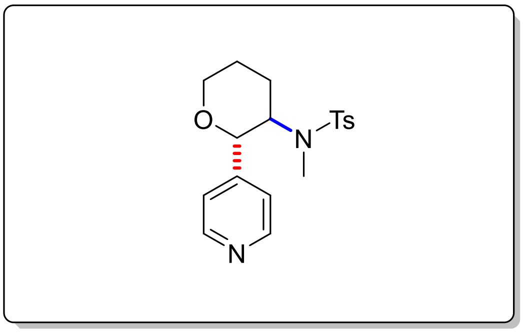

***trans*-N,4-dimethyl-N-(2-(pyridin-4-yl)tetrahydro-2H-pyran-3-yl)benzenesulfonamide (3p).** Yield 55%. 19.0 mg. Colorless gum. ^{1}H NMR (600 MHz, CD_2Cl_2) δ 8.47 (d, J = 6.0 Hz, 2H), 7.30 (d, J = 8.2 Hz, 2H), 7.20 – 7.16 (m, 4H), 4.20 (d, J = 9.8 Hz, 1H), 4.00 (dd, J = 11.5, 4.5 Hz, 1H), 3.86 (td, J = 11.8, 10.9, 3.9 Hz, 1H), 3.43 (td, J = 11.9, 2.1 Hz, 1H), 2.73 (s, 3H), 2.39 (s, 3H), 1.90 – 1.82 (m, 1H), 1.81 – 1.66 (m, 3H). ^{13}C NMR (150 MHz, CD_2Cl_2) δ 150.2, 148.7, 144.0, 137.2, 130.1, 127.4, 122.8, 80.9, 68.8, 59.3, 30.2, 27.6, 26.6, 21.8. HRMS (EI$^+$) m/z calcd. $C_{18}H_{22}N_2O_3S^+$ [M]$^+$: 346.1351, found: 346.1349.

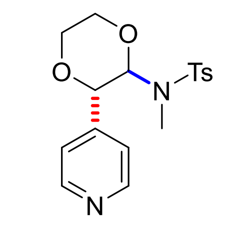

***trans*-N,4-dimethyl-N-(3-(pyridin-4-yl)-1,4-dioxan-2-yl)benzenesulfonamide (3q).** Yield 54%. 18.8 mg. Pale yellow gum. ^{1}H NMR (600 MHz, CD_2Cl_2) δ 8.57 (d, J = 5.1 Hz, 2H), 7.45 (d, J = 8.0 Hz, 2H), 7.29 (d, J = 5.1 Hz, 2H), 7.23 (d, J = 8.0 Hz, 2H), 5.16 (d, J = 8.7 Hz, 1H), 4.43 (d, J = 8.7 Hz, 1H), 3.98 (td, J = 11.9, 2.9 Hz, 1H), 3.90 – 3.82 (m, 2H), 3.71 (td, J = 11.9, 2.8 Hz, 1H), 2.79 (s, 3H), 2.40 (s, 3H). ^{13}C NMR (150 MHz, CD_2Cl_2) δ 150.4, 145.9, 144.4, 136.2, 130.0, 128.0, 122.7, 86.6, 78.5, 67.6, 66.7, 29.9, 21.8. HRMS (EI$^+$) m/z calcd. $C_{17}H_{20}N_2O_4S^+$ $[M]^+$: 348.1144, found: 348.1143.

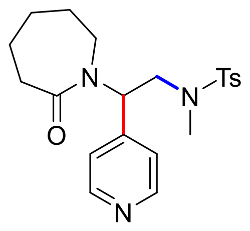

N,4-dimethyl-N-(2-(2-oxoazepan-1-yl)-2-(pyridin-4-yl)ethyl)benzenesulfonamide (3r). Yield 89%. 35.7 mg. Pale Yellow gum. ^{1}H NMR (600 MHz, CD_2Cl_2) δ 8.56 (d, J = 5.9 Hz, 2H), 7.67 (d, J = 8.2 Hz, 2H), 7.38 (d, J = 8.0 Hz, 2H), 7.27 (d, J = 5.4 Hz, 2H), 5.99 (dd, J = 9.9, 6.1 Hz, 1H), 3.71 (dd, J = 13.0, 9.9 Hz, 1H), 3.29 (dd, J = 15.5, 9.4 Hz, 1H), 3.21 (dd, J = 13.0, 6.1 Hz, 1H), 3.14 (dd, J = 15.4, 7.8 Hz, 1H), 2.72 (s, 3H), 2.62 – 2.51 (m, 2H), 2.44 (s, 3H), 1.76 – 1.54 (m, 5H), 1.18 – 1.12 (m, 1H). ^{13}C NMR (150 MHz, CD_2Cl_2) δ 177.1, 150.6, 147.4, 144.5, 134.4, 130.4, 128.0, 123.6, 53.2, 49.6, 45.5, 38.0, 35.5, 30.3, 29.3, 24.0, 21.8. HRMS (EI$^+$) m/z calcd. $C_{21}H_{27}N_3O_3S^+$ $[M]^+$: 401.1773, found: 401.1774.

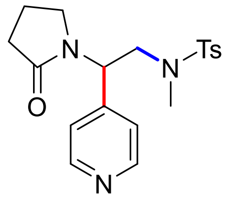

N,4-dimethyl-N-(2-(2-oxopyrrolidin-1-yl)-2-(pyridin-4-yl)ethyl)benzenesulfonamide (3s). Yield 75%. 28.0 mg. Pale yellow gum. ^{1}H NMR (600 MHz, CD_2Cl_2) δ 8.57 (d, J = 6.2 Hz, 2H), 7.66 (d, J = 8.3 Hz, 2H), 7.37 (d, J = 8.0

Hz, 2H), 7.20 (d, J = 6.2 Hz, 2H), 5.46 (dd, J = 10.9, 5.2 Hz, 1H), 3.93 (dd, J = 13.2, 10.9 Hz, 1H), 3.60 (td, J = 8.7, 5.8 Hz, 1H), 3.13 – 2.97 (m, 2H), 2.74 (s, 3H), 2.47 – 2.30 (m, 5H), 2.13 – 2.02 (m, 1H), 1.98 – 1.91 (m, 1H). ^{13}C NMR (150 MHz, CD_2Cl_2) δ 176.1, 150.8, 146.1, 144.5, 134.5, 130.4, 127.9, 123.1, 50.9, 49.6, 43.4, 35.4, 31.5, 21.8, 18.8. HRMS (EI^+) m/z calcd. $C_{19}H_{23}N_3O_3S^+$ $[M]^+$: 373.1460, found: 373.1461.

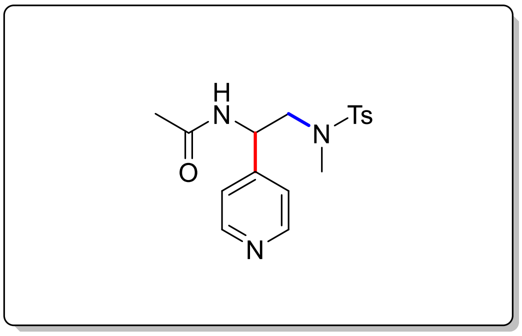

N-(2-((N,4-dimethylphenyl)sulfonamido)-1-(pyridin-4-yl)ethyl)acetamide (3t). Yield 70%. 24.3 mg. Yellow gum. ^{1}H NMR (600 MHz, CD_2Cl_2) δ 8.53 (d, J = 6.1 Hz, 2H), 7.62 (d, J = 8.3 Hz, 2H), 7.34 (d, J = 8.0 Hz, 2H), 7.24 (d, J = 6.2 Hz, 2H), 6.94 (br, 1H), 5.06 – 5.01 (m, 1H), 3.38 (dd, J = 14.4, 9.4 Hz, 1H), 3.03 (dd, J = 14.3, 4.3 Hz, 1H), 2.70 (s, 3H), 2.41 (s, 3H), 2.04 (s, 3H). ^{13}C NMR (150 MHz, CD_2Cl_2) δ 170.7, 150.7, 149.3, 144.7, 134.9, 130.5, 127.7, 122.2, 54.9, 51.6, 36.5, 23.5, 21.8. HRMS (EI^+) m/z calcd. $C_{17}H_{21}N_3O_3S^+$ $[M]^+$: 347.1304, found: 347.1300.

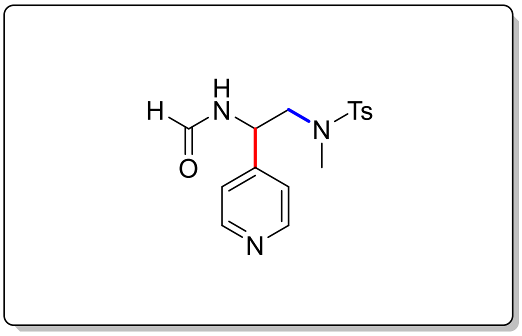

N-(2-((N,4-dimethylphenyl)sulfonamido)-1-(pyridin-4-yl)ethyl)formamide (3u). 1 mol% Eosin Y was used. Yield 65%. 21.7 mg. Brown gum. ^{1}H NMR (600 MHz, CD_2Cl_2) δ 8.56 (d, J = 4.4 Hz, 2H), 8.31 (s, 1H), 7.63 (d, J = 7.9 Hz, 2H), 7.34 (d, J = 7.9 Hz, 2H), 7.25 (d, J = 4.5 Hz, 2H), 7.01 (d, J = 6.3 Hz, 1H), 5.09 (ddd, J = 10.1, 6.4, 4.2 Hz, 1H), 3.43 (dd, J = 14.4, 9.5 Hz, 1H), 3.03 (dd, J = 14.5, 4.1 Hz, 1H), 2.70 (s, 3H), 2.42 (s, 3H). ^{13}C NMR (150 MHz, CD_2Cl_2) δ 161.7, 150.7, 148.6, 144.9, 134.7, 130.5, 127.7, 122.1, 54.9, 50.6, 36.6, 21.8. HRMS (EI^+) m/z calcd. $C_{16}H_{19}N_3O_3S^+$ $[M]^+$: 333.1147, found: 333.1145.

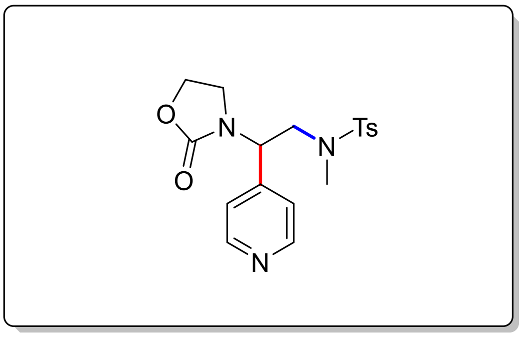

N,4-dimethyl-N-(2-(2-oxooxazolidin-3-yl)-2-(pyridin-4-yl)ethyl)benzenesulfonamide (3v). Yield 45%. 16.8 mg. Yellow oil. ^{1}H NMR (600 MHz, CD_2Cl_2) δ 8.61 (d, J = 5.0 Hz, 2H), 7.68 (d, J = 8.2 Hz, 2H), 7.38 (d, J = 8.0 Hz, 2H), 7.24 (d, J = 5.5 Hz, 2H), 5.18 (dd, J = 11.0, 5.0 Hz, 1H), 4.38 (q, J = 8.7 Hz, 1H), 4.30 (td, J = 8.9, 6.3 Hz, 1H), 4.00 (dd, J = 13.5, 11.1 Hz, 1H), 3.88 – 3.79 (m, 1H), 3.27 (q, J = 8.1 Hz, 1H), 3.02 (dd, J = 13.5, 5.1 Hz, 1H), 2.78 (s, 3H), 2.44 (s, 3H). ^{13}C NMR (150 MHz, CD_2Cl_2) δ 159.0, 151.0, 145.2, 144.7, 134.4, 130.5, 127.9, 123.0, 63.1, 53.4, 49.8, 41.1, 35.5, 21.8. HRMS (EI$^+$) m/z calcd. $C_{18}H_{21}N_3O_4S^+$ [M]$^+$: 375.1253, found: 375.1250.

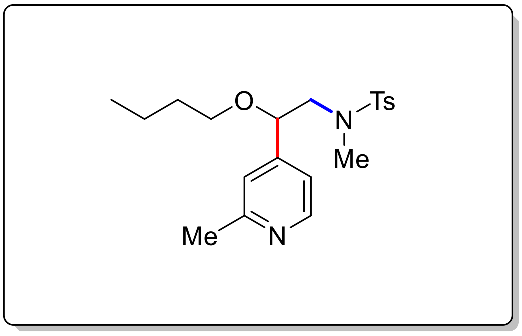

N-(2-butoxy-2-(2-methylpyridin-4-yl)ethyl)-N,4-dimethylbenzenesulfonamide (4a). Yield 89%. 33.5 mg. Colorless oil. ^{1}H NMR (600 MHz, CD_2Cl_2) δ 8.43 (d, J = 5.1 Hz, 1H), 7.62 (d, J = 8.0 Hz, 2H), 7.31 (d, J = 7.9 Hz, 2H), 7.10 (s, 1H), 7.04 (d, J = 5.1 Hz, 1H), 4.44 (dd, J = 8.4, 4.0 Hz, 1H), 3.34 – 3.22 (m, 3H), 3.03 (dd, J = 14.4, 8.3 Hz, 1H), 2.82 (s, 3H), 2.52 (s, 3H), 2.41 (s, 3H), 1.54 – 1.47 (m, 2H), 1.38 – 1.29 (m, 2H), 0.88 (t, J = 7.4 Hz, 3H). ^{13}C NMR (150 MHz, CD_2Cl_2) δ 159.4, 149.9, 149.7, 144.1, 135.8, 130.2, 127.8, 121.7, 119.3, 81.7, 70.1, 56.7, 37.6, 32.5, 24.8, 21.8, 19.9, 14.2. HRMS (EI$^+$) m/z calcd. $C_{20}H_{28}N_2O_3S^+$ [M]$^+$: 376.1821, found: 376.1824.

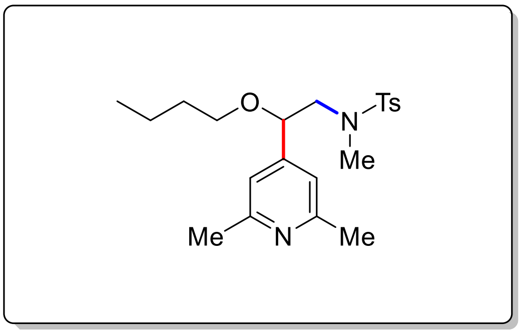

N-(2-butoxy-2-(2,6-dimethylpyridin-4-yl)ethyl)-N,4-dimethylbenzenesulfonamide (4b). Yield 79%. 30.8 mg. White solid. mp 68-70 °C. ^{1}H NMR (600 MHz, CD_2Cl_2) δ 7.61 (d, J = 8.2 Hz, 2H), 7.31 (d, J = 7.9 Hz, 2H), 6.89 (s, 2H), 4.39 (dd, J = 8.5, 3.8 Hz, 1H), 3.33 – 3.28 (m, 1H), 3.26 – 3.21 (m, 2H), 3.00 (dd, J = 14.4, 8.5 Hz, 1H), 2.82

(s, 3H), 2.47 (s, 6H), 2.41 (s, 3H), 1.53 – 1.47 (m, 2H), 1.38 – 1.30 (m, 2H), 0.88 (t, J = 7.4 Hz, 3H). ^{13}C NMR (150 MHz, CD_2Cl_2) δ 158.6, 150.0, 144.1, 135.8, 130.2, 127.8, 118.6, 81.7, 70.0, 56.8, 37.6, 32.5, 24.7, 21.8, 19.9, 14.2.. HRMS (EI^+) m/z calcd. $C_{21}H_{30}N_2O_3S^+$ $[M]^+$: 390.1977, found: 390.1975.

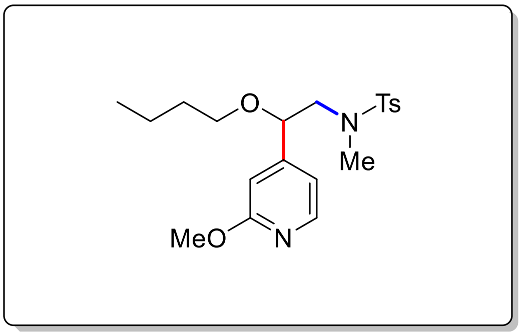

N-(2-butoxy-2-(2-methoxypyridin-4-yl)ethyl)-N,4-dimethylbenzenesulfonamide (4c). Yield 76%. 29.8 mg. Colorless oil. ^{1}H NMR (600 MHz, CD_2Cl_2) δ 8.11 (d, J = 5.2 Hz, 1H), 7.61 (d, J = 8.2 Hz, 2H), 7.31 (d, J = 8.0 Hz, 2H), 6.99 – 6.79 (m, 1H), 6.68 (s, 1H), 4.43 (dd, J = 8.2, 4.0 Hz, 1H), 3.91 (s, 3H), 3.36 – 3.31 (m, 1H), 3.29 – 3.22 (m, 2H), 3.02 (dd, J = 14.4, 8.3 Hz, 1H), 2.82 (s, 3H), 2.41 (s, 3H), 1.53 – 1.46 (m, 2H), 1.38 – 1.30 (m, 2H), 0.88 (t, J = 7.4 Hz, 3H). ^{13}C NMR (150 MHz, CD_2Cl_2) δ 165.2, 152.6, 147.7, 144.1, 135.7, 130.2, 127.8, 115.6, 109.1, 81.5, 70.1, 56.6, 53.9, 37.6, 32.5, 21.8, 19.9, 14.2. HRMS (EI^+) m/z calcd. $C_{20}H_{28}N_2O_4S^+$ $[M]^+$: 392.1770, found: 392.1772.

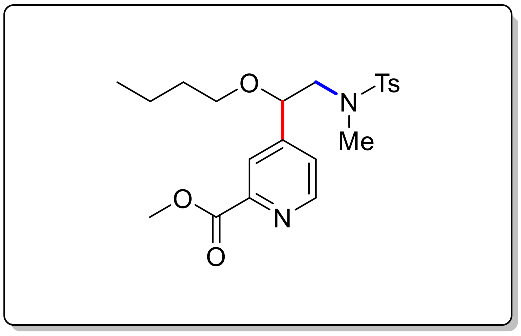

methyl 4-(1-butoxy-2-((N,4-dimethylphenyl)sulfonamido)ethyl)picolinate (4d). Yield 87%. 36.6 mg. Colorless oil. ^{1}H NMR (600 MHz, CD_2Cl_2) δ 8.68 (d, J = 4.8 Hz, 1H), 8.03 (s, 1H), 7.61 (d, J = 8.0 Hz, 2H), 7.46 (d, J = 4.5 Hz, 1H), 7.31 (d, J = 7.9 Hz, 2H), 4.57 (dd, J = 7.6, 4.2 Hz, 1H), 3.96 (s, 3H), 3.32 (t, J = 6.5 Hz, 2H), 3.26 (dd, J = 14.4, 4.0 Hz, 1H), 3.09 (dd, J = 14.4, 8.0 Hz, 1H), 2.82 (s, 3H), 2.41 (s, 3H), 1.55 – 1.49 (m, 2H), 1.39 – 1.31 (m, 2H), 0.88 (t, J = 7.3 Hz, 3H). ^{13}C NMR (150 MHz, CD_2Cl_2) δ 166.2, 151.0, 150.6, 149.1, 144.2, 135.6, 130.3, 127.8, 125.5, 123.6, 81.3, 70.4, 56.5, 53.1, 37.7, 32.4, 21.8, 19.8, 14.2. HRMS (EI^+) m/z calcd. $C_{21}H_{28}N_2O_5S^+$ $[M]^+$: 420.1719, found: 420.1720.

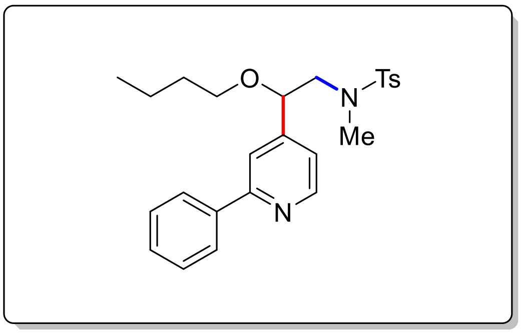

N-(2-butoxy-2-(2-phenylpyridin-4-yl)ethyl)-N,4-dimethylbenzenesulfonamide (4e). Yield 93%. 40.8 mg. Colorless oil. ^{1}H NMR (600 MHz, CD_2Cl_2) δ 8.65 (d, J = 4.9 Hz, 1H), 8.04 (d, J = 7.3 Hz, 2H), 7.72 (s, 1H), 7.63 (d, J = 8.2 Hz, 2H), 7.49 (t, J = 7.5 Hz, 2H), 7.43 (t, J = 7.3 Hz, 1H), 7.31 (d, J = 8.0 Hz, 2H), 7.22 (d, J = 4.9 Hz, 1H), 4.57 (dd, J = 8.1, 4.1 Hz, 1H), 3.42 – 3.27 (m, 3H), 3.12 (dd, J = 14.4, 8.2 Hz, 1H), 2.85 (s, 3H), 2.40 (s, 3H), 1.59 – 1.52 (m, 2H), 1.43 – 1.33 (m, 2H), 0.90 (t, J = 7.4 Hz, 3H). ^{13}C NMR (150 MHz, CD_2Cl_2) δ 158.0, 150.4, 144.1, 139.8, 135.8, 130.3, 129.6, 129.2, 127.8, 127.4, 120.9, 118.8, 81.8, 70.2, 56.7, 37.6, 32.5, 21.8, 19.9, 14.2. HRMS (EI$^+$) m/z calcd. $C_{25}H_{30}N_2O_3S^+$ [M]$^+$: 438.1977, found: 438.1981.

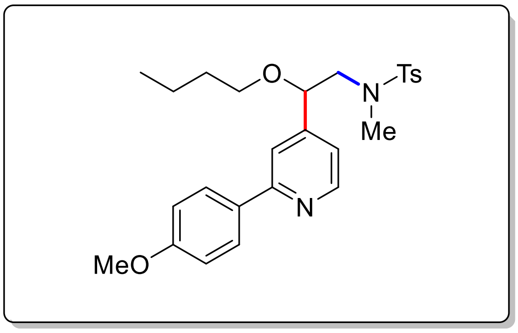

N-(2-butoxy-2-(2-(4-methoxyphenyl)pyridin-4-yl)ethyl)-N,4-dimethylbenzenesulfonamide (4f). Yield 99%. 46.4 mg. Colorless oil. ^{1}H NMR (600 MHz, CD_2Cl_2) δ 8.60 (d, J = 4.9 Hz, 1H), 8.00 (d, J = 8.8 Hz, 2H), 7.67 – 7.53 (m, 3H), 7.31 (d, J = 8.0 Hz, 2H), 7.16 – 7.13 (m, 1H), 7.01 (d, J = 8.8 Hz, 2H), 4.55 (dd, J = 8.2, 4.0 Hz, 1H), 3.86 (s, 3H), 3.41 – 3.26 (m, 3H), 3.12 (dd, J = 14.4, 8.3 Hz, 1H), 2.85 (s, 3H), 2.40 (s, 3H), 1.59 – 1.47 (m, 2H), 1.43 – 1.33 (m, 2H), 0.90 (t, J = 7.4 Hz, 3H). ^{13}C NMR (150 MHz, CD_2Cl_2) δ 161.3, 157.7, 150.3, 150.3, 144.1, 135.8, 132.3, 130.3, 128.7, 127.8, 120.2, 118.0, 114.6, 81.9, 70.2, 56.8, 55.9, 37.6, 32.5, 21.8, 19.9, 14.2. HRMS (EI$^+$) m/z calcd. $C_{26}H_{32}N_2O_4S^+$ [M]$^+$: 468.2083, found: 468.2085.

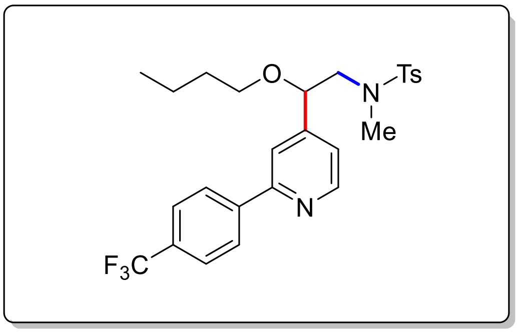

N-(2-butoxy-2-(2-(4-(trifluoromethyl)phenyl)pyridin-4-yl)ethyl)-N,4-dimethylbenzenesulfonamide (4g). Yield 85%. 43.1 mg. Colorless oil. ^{1}H NMR (600 MHz, CD_2Cl_2) δ 8.69 (d, J = 4.9 Hz, 1H), 8.19 (d, J = 8.2 Hz, 2H), 7.82 – 7.72 (m, 3H), 7.62 (d, J = 8.2 Hz, 2H), 7.31 (d, J = 8.2 Hz, 2H), 7.29 (d, J = 4.8 Hz, 1H), 4.60 (dd, J = 8.0, 4.2 Hz, 1H), 3.42 – 3.26 (m, 3H), 3.14 (dd, J = 14.4, 8.0 Hz, 1H), 2.85 (s, 3H), 2.39 (s, 3H), 1.59 – 1.50 (m, 2H), 1.44 – 1.32 (m, 2H), 0.90 (t, J = 7.4 Hz, 3H). ^{13}C NMR (150 MHz, CD_2Cl_2) δ 156.5, 150.8, 150.7, 144.2, 143.3, 135.7, 131.2 (q, J = 32.2 Hz), 130.3, 127.9, 127.8, 126.2 (q, J = 3.6 Hz), 125.0 (q, J = 270.7 Hz), 121.8, 119.3, 81.7, 70.4, 56.7, 37.7, 32.5, 21.8, 19.9, 14.2. ^{19}F NMR (564 MHz, CD_2Cl_2) δ -62.90. HRMS (EI^+) m/z calcd. $C_{26}H_{29}F_3N_2O_3S^+$ $[M]^+$: 506.1851, found: 506.1849.

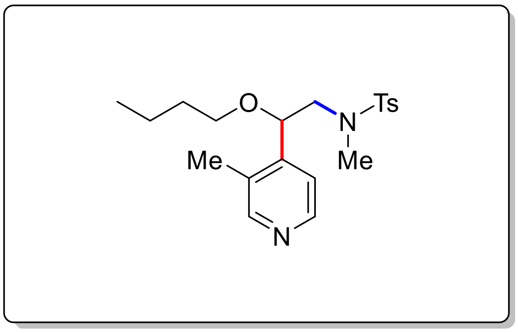

N-(2-butoxy-2-(3-methylpyridin-4-yl)ethyl)-N,4-dimethylbenzenesulfonamide (4h). Yield 83%. 30.9 mg. Colorless oil. ^{1}H NMR (600 MHz, CD_2Cl_2) δ 8.40 (d, J = 4.9 Hz, 1H), 8.37 (s, 1H), 7.62 (d, J = 8.3 Hz, 2H), 7.31 (d, J = 8.0 Hz, 2H), 7.26 (d, J = 4.9 Hz, 1H), 4.73 (dd, J = 8.6, 3.2 Hz, 1H), 3.35 – 3.17 (m, 3H), 2.89 (s, 3H), 2.87 (dd, J = 14.7, 8.8 Hz, 1H), 2.40 (s, 3H), 2.37 (s, 3H), 1.57 – 1.48 (m, 2H), 1.42 – 1.28 (m, 2H), 0.89 (t, J = 7.4 Hz, 3H). ^{13}C NMR (150 MHz, CD_2Cl_2) δ 151.8, 148.4, 147.3, 144.1, 135.8, 131.7, 130.3, 127.7, 120.8, 79.3, 70.0, 55.7, 37.9, 32.5, 21.8, 19.9, 16.3, 14.2. HRMS (EI^+) m/z calcd. $C_{20}H_{28}N_2O_3S^+$ $[M]^+$: 376.1821, found: 376.1820.

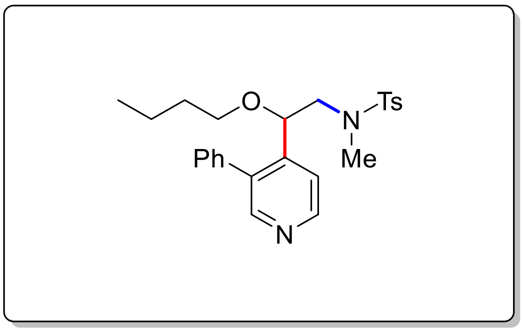

N-(2-butoxy-2-(3-phenylpyridin-4-yl)ethyl)-N,4-dimethylbenzenesulfonamide (4i). Yield 53%. 23.3 mg. Colorless oil. ^{1}H NMR (600 MHz, CD_2Cl_2) δ 8.60 (s, 1H), 8.45 (s, 1H), 7.54 – 7.49 (m, 2H), 7.49 – 7.40 (m, 4H), 7.34 – 7.31 (m, 2H), 7.26 (d, J = 8.0 Hz, 2H), 4.59 (dd, J = 8.4, 3.3 Hz, 1H), 3.25 – 3.13 (m, 2H), 3.07 – 3.02 (m, 1H), 2.98 (dd, J = 14.4, 8.5 Hz, 1H), 2.66 (s, 3H), 2.39 (s, 3H), 1.44 – 1.35 (m, 2H), 1.33 – 1.18 (m, 2H), 0.83 (t, J = 7.4 Hz, 3H). ^{13}C NMR (150 MHz, CD_2Cl_2) δ 150.9, 149.7, 146.7, 143.9, 137.4, 135.9, 130.1, 130.1, 129.2, 128.6, 127.7, 121.4, 77.2, 69.7, 55.9, 37.2, 32.4, 21.8, 19.8, 14.2.. HRMS (ESI^+) m/z calcd. $C_{25}H_{31}N_2O_3S^+$ $[M+H]^+$: 439.2050, found: 439.2057.

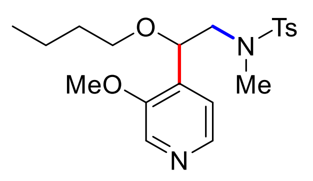

N-(2-butoxy-2-(3-methoxypyridin-4-yl)ethyl)-N,4-dimethylbenzenesulfonamide (4j). Yield 64%. 24.9 mg. Colorless oil. ^{1}H NMR (600 MHz, CD_2Cl_2) δ 8.26 – 8.21 (m, 2H), 7.61 (d, J = 7.9 Hz, 2H), 7.30 (d, J = 7.9 Hz, 2H), 7.28 (d, J = 4.7 Hz, 1H), 4.83 (dd, J = 8.2, 3.1 Hz, 1H), 3.94 (s, 3H), 3.35 – 3.22 (m, 3H), 3.06 (dd, J = 14.1, 8.1 Hz, 1H), 2.88 (s, 3H), 2.41 (s, 3H), 1.54 – 1.47 (m, 2H), 1.39 – 1.30 (m, 2H), 0.89 (t, J = 7.4 Hz, 3H). ^{13}C NMR (150 MHz, CD_2Cl_2) δ 153.6, 143.9, 143.4, 137.0, 136.0, 133.8, 130.1, 127.8, 121.5, 75.5, 70.3, 56.6, 54.9, 37.2, 32.5, 21.8, 19.9, 14.2. HRMS (ESI$^+$) m/z calcd. $C_{20}H_{29}N_2O_4S^+$ $[M+H]^+$: 393.1843, found: 393.1842.

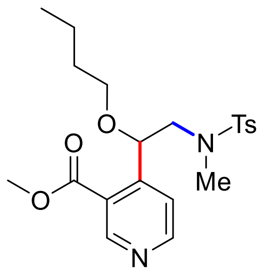

methyl 4-(1-butoxy-2-((N,4-dimethylphenyl)sulfonamido)ethyl)nicotinate (4k). Yield 65%. 27.1 mg. Colorless oil. ^{1}H NMR (600 MHz, CD_2Cl_2) δ 9.06 (s, 1H), 8.71 (d, J = 5.1 Hz, 1H), 7.60 (dd, J = 9.5, 6.8 Hz, 3H), 7.29 (d, J = 8.0 Hz, 2H), 5.35 (dd, J = 7.8, 3.2 Hz, 1H), 3.94 (s, 3H), 3.30 – 3.25 (m, 3H), 3.19 (dd, J = 14.0, 3.2 Hz, 1H), 2.88 (s, 3H), 2.40 (s, 3H), 1.53 – 1.46 (m, 2H), 1.38 – 1.29 (m, 2H), 0.88 (t, J = 7.4 Hz, 3H). ^{13}C NMR (150 MHz, CD_2Cl_2) δ 166.8, 153.5, 152.0, 151.6, 143.9, 135.7, 130.1, 127.9, 125.5, 122.3, 77.3, 70.5, 55.7, 53.0, 36.9, 32.4, 21.8, 19.9, 14.2. HRMS (EI$^+$) m/z calcd. $C_{21}H_{28}N_2O_5S^+$ $[M]^+$: 420.1719, found: 420.1717.

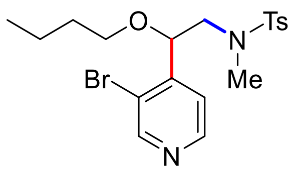

N-(2-(3-bromopyridin-4-yl)-2-butoxyethyl)-N,4-dimethylbenzenesulfonamide (4l). Yield 82%. 36.2 mg. Colorless oil. ^{1}H NMR (600 MHz, CD_2Cl_2) δ 8.65 (s, 1H), 8.50 (d, J = 4.9 Hz, 1H), 7.63 (d, J = 8.3 Hz, 2H), 7.39 (d, J = 4.9 Hz, 1H), 7.31 (d, J = 8.0 Hz, 2H), 4.82 (dd, J = 8.3, 3.1 Hz, 1H), 3.33 – 3.26 (m, 3H), 3.06 (dd, J = 14.4,

8.3 Hz, 1H), 2.92 (s, 3H), 2.41 (s, 3H), 1.54 – 1.48 (m, 2H), 1.39 – 1.30 (m, 2H), 0.89 (t, J = 7.4 Hz, 3H). ^{13}C NMR (150 MHz, CD_2Cl_2) δ 152.5, 149.2, 148.3, 144.1, 135.8, 130.2, 127.8, 123.3, 121.8, 80.0, 70.5, 54.7, 37.5, 32.4, 21.8, 19.8, 14.2. HRMS (EI$^+$) m/z calcd. $C_{19}H_{25}BrN_2O_3S^+$ [M]$^+$: 440.0769, found: 440.0770.

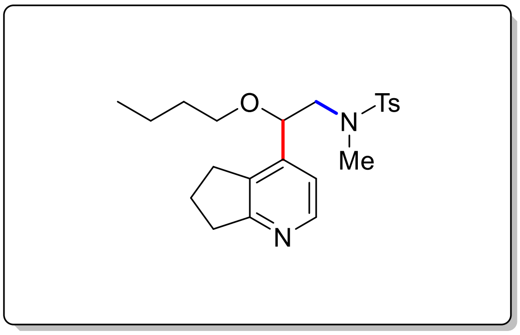

N-(2-butoxy-2-(6,7-dihydro-5H-cyclopenta[b]pyridin-4-yl)ethyl)-N,4-dimethylbenzenesulfonamide (4m). Yield 83%. 33.4 mg. Pale yellow oil. ^{1}H NMR (600 MHz, CD_2Cl_2) δ 8.31 (s, 1H), 7.61 (d, J = 7.7 Hz, 2H), 7.31 (d, J = 7.8 Hz, 2H), 7.09 (d, J = 4.3 Hz, 1H), 4.80 – 4.41 (m, 1H), 3.35 – 3.14 (m, 3H), 3.05 – 2.92 (m, 5H), 2.86 (s, 3H), 2.41 (s, 3H), 2.22 – 2.10 (m, 2H), 1.58 – 1.41 (m, 2H), 1.40 – 1.29 (m, 2H), 0.89 (t, J = 7.3 Hz, 3H). ^{13}C NMR (150 MHz, CD_2Cl_2) δ 166.2, 147.4, 145.9, 144.1, 136.1, 135.8, 130.3, 127.7, 118.9, 80.3, 70.2, 55.4, 37.8, 34.4, 32.5, 29.7, 23.6, 21.8, 19.9, 14.2. HRMS (ESI$^+$) m/z calcd. $C_{22}H_{31}N_2O_3S^+$ [M+H]$^+$: 403.2050, found: 403.2056.

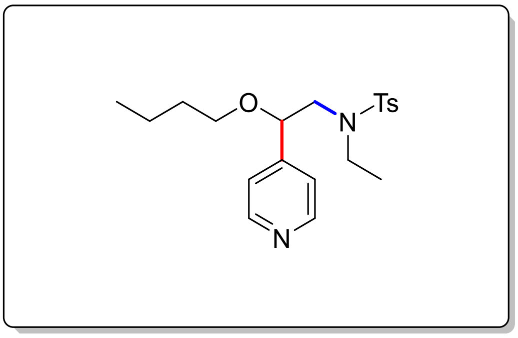

N-(2-butoxy-2-(pyridin-4-yl)ethyl)-N-ethyl-4-methylbenzenesulfonamide (4n). Yield 70%. 26.3 mg. Pale yellow oil. ^{1}H NMR (600 MHz, CD_2Cl_2) δ 8.56 (s, 2H), 7.66 (d, J = 8.3 Hz, 2H), 7.30 (d, J = 8.0 Hz, 2H), 7.26 (d, J = 5.2 Hz, 2H), 4.52 (dd, J = 8.3, 3.9 Hz, 1H), 3.38 – 3.15 (m, 6H), 2.41 (s, 3H), 1.55 – 1.45 (m, 2H), 1.39 – 1.29 (m, J = 7.2 Hz, 2H), 1.02 (t, J = 7.1 Hz, 3H), 0.88 (t, J = 7.4 Hz, 3H). ^{13}C NMR (150 MHz, CD_2Cl_2) 150.5, 149.7, 144.0, 137.9, 130.2, 127.6, 122.4, 81.6, 70.2, 45.2, 32.5, 21.8, 19.9, 14.2, 14.0. HRMS (EI$^+$) m/z calcd. $C_{20}H_{28}N_2O_3S^+$ [M]$^+$: 376.1821, found: 376.1823.

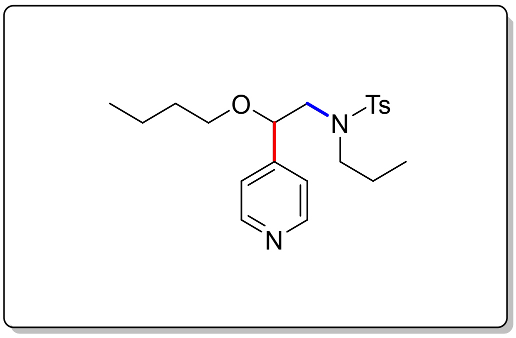

N-(2-butoxy-2-(pyridin-4-yl)ethyl)-4-methyl-N-propylbenzenesulfonamide (4o). Yield 66%. 25.7 mg. Pale yellow oil. ^{1}H NMR (600 MHz, CD_2Cl_2) δ 8.55 (d, J = 4.9 Hz, 2H), 7.67 (d, J = 8.3 Hz, 2H), 7.31 (d, J = 8.0 Hz, 2H), 7.25 (d, J = 5.7 Hz, 2H), 4.52 (dd, J = 8.5, 3.7 Hz, 1H), 3.32 – 3.19 (m, 4H), 3.14 (dd, J = 15.1, 8.5 Hz, 1H), 3.05 (ddd, J = 14.4, 9.5, 5.7 Hz, 1H), 2.41 (s, 3H), 1.57 – 1.44 (m, 4H), 1.38 – 1.25 (m, 2H), 0.88 (t, J = 7.4 Hz, 3H), 0.80 (t, J = 7.4 Hz, 3H). ^{13}C NMR (150 MHz, CD_2Cl_2) δ 150.6, 149.8, 144.0, 137.8, 130.2, 127.7, 122.3, 81.5, 70.2, 54.7, 52.3, 32.5, 22.0, 21.8, 19.9, 14.2, 11.4. HRMS (EI^+) m/z calcd. $C_{21}H_{30}N_2O_3S^+$ $[M]^+$: 390.1977, found: 390.1980.

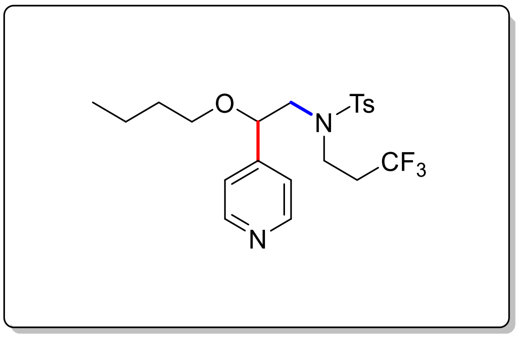

N-(2-butoxy-2-(pyridin-4-yl)ethyl)-4-methyl-N-(3,3,3-trifluoropropyl)benzenesulfonamide (4p). Yield 62%. 27.4 mg. Colorless oil. ^{1}H NMR (600 MHz, CD_2Cl_2) δ 8.58 (d, J = 4.9 Hz, 2H), 7.66 (d, J = 8.2 Hz, 2H), 7.33 (d, J = 8.0 Hz, 2H), 7.24 (d, J = 4.9 Hz, 2H), 4.51 (dd, J = 8.9, 2.8 Hz, 1H), 3.61 (ddd, J = 14.5, 10.1, 6.1 Hz, 1H), 3.45 (dd, J = 15.3, 2.8 Hz, 1H), 3.36 – 3.19 (m, 3H), 3.00 (dd, J = 15.2, 8.8 Hz, 1H), 2.64 – 2.51 (m, 2H), 2.42 (s, 3H), 1.58 – 1.45 (m, 2H), 1.41 – 1.15 (m, 2H), 0.88 (t, J = 7.4 Hz, 3H). ^{13}C NMR (150 MHz, CD_2Cl_2) δ 150.7, 149.0, 144.7, 136.6, 130.5, 127.7, 126.6 (q, J = 277.0 Hz), 122.2, 82.0, 70.2, 56.2, 44.6 (q, J = 3.8 Hz), 34.5 (q, J = 27.7 Hz), 32.4, 21.8, 19.8, 14.1. ^{19}F NMR (564 MHz, CD_2Cl_2) δ -65.94 (t, J = 10.8 Hz). HRMS (EI^+) m/z calcd. $C_{21}H_{27}F_3N_2O_3S^+$ $[M]^+$: 444.1694, found: 444.1696.

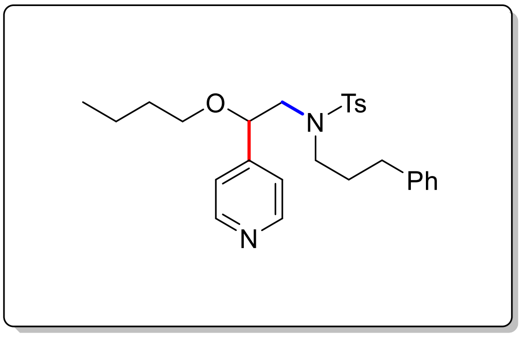

N-(2-butoxy-2-(pyridin-4-yl)ethyl)-4-methyl-N-(3-phenylpropyl)benzenesulfonamide (4q). Yield 61%. 28.5 mg. Colorless oil. ^{1}H NMR (600 MHz, CD_2Cl_2) δ 8.54 (d, J = 5.6 Hz, 2H), 7.63 (d, J = 8.2 Hz, 2H), 7.28 (dd, J = 16.6, 8.1 Hz, 4H), 7.24 – 7.16 (m, 3H), 7.13 (d, J = 7.2 Hz, 2H), 4.48 (dd, J = 8.5, 3.5 Hz, 1H), 3.36 – 3.23 (m, 3H), 3.22 – 3.08 (m, 3H), 2.58 – 2.47 (m, 2H), 2.41 (s, 3H), 1.89 – 1.74 (m, 2H), 1.51 – 1.41 (m, 2H), 1.36 – 1.24 (m, 2H), 0.86 (t, J = 7.4 Hz, 3H). ^{13}C NMR (150 MHz, CD_2Cl_2) δ 150.6, 149.7, 144.1, 142.1, 137.6, 130.3, 128.9, 128.9, 127.7, 126.5, 122.3, 81.5, 70.2, 54.8, 50.1, 33.4, 32.5, 30.2, 21.8, 19.8, 14.2. HRMS (ESI$^+$) m/z calcd. $C_{27}H_{35}N_2O_3S^+$ $[M+H]^+$: 467.2363, found: 467.2359.

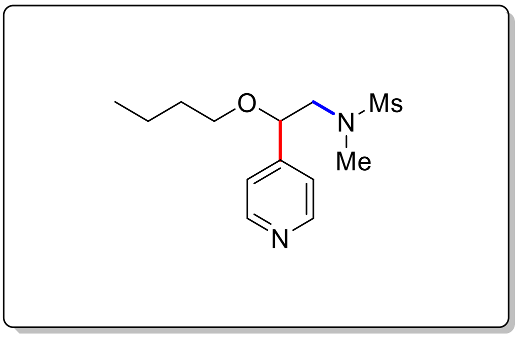

N-(2-butoxy-2-(pyridin-4-yl)ethyl)-N-methylmethanesulfonamide (4r). Yield 80%. 22.9 mg. Colorless oil. ^{1}H NMR (600 MHz, CD_2Cl_2) δ 8.59 (s, 2H), 7.30 (d, J = 4.1 Hz, 2H), 4.51 (dd, J = 7.8, 4.4 Hz, 1H), 3.37 (t, J = 6.5 Hz, 2H), 3.34 – 3.25 (m, 2H), 2.92 (s, 3H), 2.78 (s, 3H), 1.60 – 1.52 (m, 2H), 1.44 – 1.32 (m, 2H), 0.90 (t, J = 7.4 Hz, 3H). ^{13}C NMR (150 MHz, CD_2Cl_2) δ 150.3, 149.7, 122.4, 81.2, 70.3, 56.4, 36.9, 36.8, 32.5, 19.9, 14.2. HRMS (EI$^+$) m/z calcd. $C_{13}H_{22}N_2O_3S^+$ $[M]^+$: 286.1351, found: 286.1352.

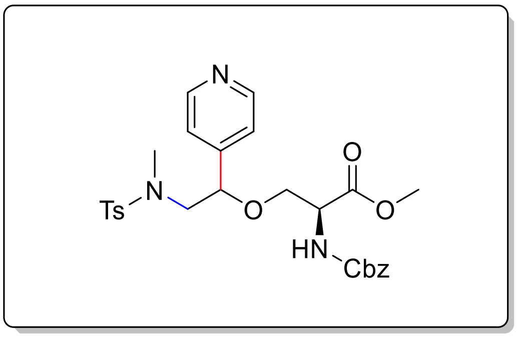

methyl N-((benzyloxy)carbonyl)-O-(2-((N,4-dimethylphenyl)sulfonamido)-1-(pyridin-4-yl)ethyl)-L-serinate (5a). From 0.2 mmol L-serine derivatives : Diastereomeric mixture (1.1:1) Yield 75%. 81.2 mg. Pale yellow solid. ^{1}H NMR (600 MHz, CD_2Cl_2) δ 8.60 (s, 2H), 7.61 (d, *J* = 8.2 Hz, 2H), 7.42 – 7.35 (m, 4H), 7.32 (dd, *J* = 11.0, 7.3 Hz, 3H), 7.18 (d, *J* = 4.6 Hz, 2H), 5.91 (d, *J* = 8.6 Hz, 1H), 5.13 (d, *J* = 2.5 Hz, 2H), 4.53 (t, *J* = 6.2 Hz, 1H), 4.48 (dt, *J* = 8.8, 3.2 Hz, 1H), 3.81 (dd, *J* = 9.4, 3.2 Hz, 1H), 3.72 (s, 3H), 3.66 (dd, *J* = 9.4, 3.2 Hz, 1H), 3.16 (d, *J* = 6.1 Hz, 2H), 2.72 (s, 3H), 2.40 (s, 3H). ^{13}C NMR (150MHz, CD_2Cl_2) δ 170.9, 156.4, 150.5, 148.1, 144.4, 137.3, 135.3, 130.3, 129.0, 128.6, 128.4, 127.8, 122.3, 81.6, 70.3, 67.4, 56.4, 55.0, 53.0, 37.5, 21.8. HRMS (ESI$^+$) m/z calcd. $C_{27}H_{32}N_3O_7S^+$ $[M+H]^+$: 542.1955, found: 542.1955.

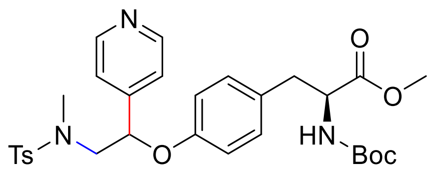

methyl (2S)-2-((tert-butoxycarbonyl)amino)-3-(4-(2-((N,4-dimethylphenyl)sulfonamido)-1-(pyridin-4-yl)ethoxy)phenyl)propanoate (5b). From 0.2 mmol L-tyrosine derivatives : Diastereomeric mixture (1:1) Yield 61%. 74.7 mg. White solid. ^{1}H NMR (600 MHz, CD_2Cl_2) δ 8.58 (d, *J* = 5.0 Hz, 2H), 7.72 – 7.62 (m, 2H), 7.34 (d, *J* = 5.3 Hz, 2H), 7.31 (d, *J* = 8.1 Hz, 2H), 6.98 (d, *J* = 8.2 Hz, 2H), 6.74 (d, *J* = 8.1 Hz, 2H), 5.37 (dd, *J* = 7.6, 4.0 Hz, 1H), 5.06 (d, *J* = 8.3 Hz, 1H), 4.46 (q, *J* = 6.8 Hz, 1H), 3.67 (s, 3H), 3.45 (ddd, *J* = 14.7, 4.3, 2.0 Hz, 1H), 3.35 (ddd, *J* = 14.7, 8.0, 1.8 Hz, 1H), 3.01 (dt, *J* = 11.2, 3.2 Hz, 1H), 2.91 (dt, *J* = 13.7, 6.6 Hz, 1H), 2.86 (s, 3H), 2.40 (s, 3H), 1.38 (s, 9H). ^{13}C NMR (150MHz, CD_2Cl_2) δ 172.8, 156.8, 155.5, 150.7, 148.2, 144.3, 135.5, 130.9, 130.3, 130.1, 127.7, 121.8, 116.2, 80.1, 79.6, 56.7, 55.1, 52.6, 37.8, 28.6, 21.8. HRMS (ESI$^+$) m/z calcd. $C_{30}H_{37}N_3O_7SNa^+$ $[M+Na]^+$: 606.2244, found: 606.2248.

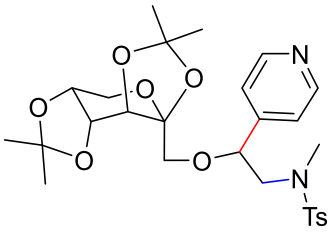

N,4-dimethyl-N-(2-(pyridin-4-yl)-2-(((3aS,5aR,8aR,8bS)-2,2,7,7-tetramethyltetrahydro-3aH-bis([1,3]dioxolo)[4,5-b:4',5'-d]pyran-3a-yl)methoxy)ethyl)benzenesulfonamide (5c). From 0.2 mmol D-fructopyranose derivatives : Diastereomeric mixture (1.3:1) Yield 71%. 77.8 mg. White solid. ^{1}H NMR (600 MHz, CD_2Cl_2) δ 8.58 (d, *J* = 4.9 Hz, 2H), 7.67 – 7.55 (m, 2H), 7.35 – 7.21 (m, 4H), 4.63 – 4.53 (m, 2H), 4.43 – 4.36 (m, 1H), 4.24 – 4.14 (m, 1H), 3.90 – 3.80 (m, 1H), 3.68 – 3.60 (m, 1H), 3.53 – 3.38 (m, 2H), 3.29 – 3.07 (m, 2H), 2.79 – 2.68 (m, 3H), 2.41 (s, 3H), 1.57 – 1.50 (m, 3H), 1.46 – 1.41 (m, 3H), 1.40 – 1.32 (m, 3H), 1.32 – 1.17 (m, 3H). ^{13}C NMR (150MHz, CD_2Cl_2) δ 150.5, 148.6, 144.3, 135.3, 130.3, 127.8, 122.4, 109.4, 109.2, 102.7, 81.2, 71.7, 71.6, 70.7, 70.6, 61.6, 56.6, 37.4, 26.9, 26.3, 25.9, 24.4, 21.8. HRMS (ESI$^+$) m/z calcd. $C_{27}H_{37}N_2O_8S^+$ $[M+H]^+$: 549.2265, found: 549.2266.

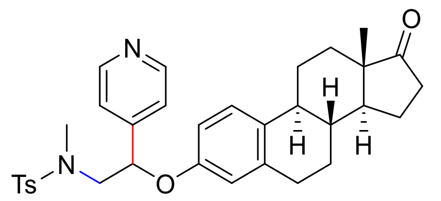

N,4-dimethyl-N-(2-(((8R,9S,13S,14S)-13-methyl-17-oxo-7,8,9,11,12,13,14,15,16,17-decahydro-6H-cyclopenta[a]phenanthren-3-yl)oxy)-2-(pyridin-4-yl)ethyl)benzenesulfonamide (5d). From 0.2 mmol estrone derivatives : Diastereomeric mixture (1:1) Yield 62%. 69.3 mg. Pale yellow solid. ^{1}H NMR (600 MHz, CD_2Cl_2) δ 8.61 (s, 2H), 7.64 (d, *J* = 7.8 Hz, 2H), 7.36 (s, 2H), 7.31 (d, *J* = 7.9 Hz, 2H), 7.11 (d, *J* = 8.6 Hz, 1H), 6.58 (d, *J* = 8.8 Hz, 1H), 6.55 (s, 1H), 5.40 – 5.34 (m, 1H), 3.45 (dd, *J* = 14.7, 4.0 Hz, 1H), 3.34 (dd, *J* = 14.7, 8.1 Hz, 1H), 2.87 (s, 3H), 2.85 – 2.73 (m, 2H), 2.45 (dd, *J* = 18.8, 8.9 Hz, 1H), 2.41 (s, 3H), 2.35 – 2.28 (m, 1H), 2.20 (td, *J* = 10.6, 3.9 Hz, 1H), 2.13 – 1.94 (m, 3H), 1.90 – 1.84 (m, 1H), 1.65 – 1.49 (m, 2H), 1.51 – 1.34 (m, 4H), 0.87 (s, 3H). ^{13}C NMR (150MHz, CD_2Cl_2) δ 220.7, 155.6, 150.6, 148.5, 144.3, 138.7, 135.6, 133.9, 130.3, 127.7, 126.9, 122.0, 116.3, 113.5, 79.4, 56.7, 50.9, 48.4, 44.5, 38.8, 37.8, 36.3, 32.2, 30.1, 27.0, 26.4, 22.0, 21.8, 14.2. HRMS (ESI^{+}) m/z calcd. $C_{33}H_{39}N_2O_4S^+$ [M+H]$^{+}$: 559.2625, found: 559.2630.

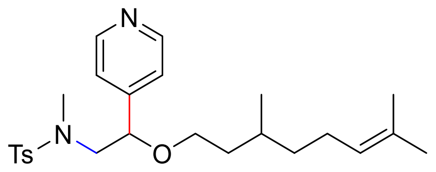

N-(2-((3,7-dimethyloct-6-en-1-yl)oxy)-2-(pyridin-4-yl)ethyl)-N,4-dimethylbenzenesulfonamide (5e). From 0.2 mmol beta-citronellol derivatives : Diastereomeric mixture (1:1) Yield 78%. 69.3 mg. Colorless gum. ^{1}H NMR (600 MHz, CD_2Cl_2) δ 8.59 (s, 2H), 7.62 (d, *J* = 7.9 Hz, 2H), 7.32 (d, *J* = 7.9 Hz, 2H), 7.27 (d, *J* = 4.7 Hz, 2H), 5.16 – 4.92 (m, 1H), 4.50 (dd, *J* = 8.2, 4.1 Hz, 1H), 3.41 – 3.28 (m, 2H), 3.26 (ddd, *J* = 14.4, 5.7, 4.2 Hz, 1H), 3.09 – 3.01 (m, 1H), 2.81 (d, *J* = 2.2 Hz, 3H), 2.41 (s, 3H), 1.96 (ddt, *J* = 36.0, 14.5, 7.6 Hz, 2H), 1.67 (s, 3H), 1.59 (s, 3H), 1.63 – 1.49 (m, 2H), 1.39 – 1.32 (m, 1H), 1.31 – 1.22 (m, 1H), 1.18 – 1.08 (m, 1H), [0.86 (d, *J* = 6.5 Hz, 3H), 0.83 (d, *J* = 6.6 Hz, 3H) diastereomer]. ^{13}C NMR (150MHz, CD_2Cl_2) δ 150.5, 149.5, 144.2, 135.7, 131.6, 130.3, 127.8, 125.3, 122.4, 81.7, 68.8, 56.7, 37.7, 37.6, 37.4, 30.0, 26.0, 26.0, 21.8, 19.8, 17.9. HRMS (ESI^{+}) m/z calcd. $C_{25}H_{37}N_2O_3S^+$ [M+H]$^{+}$: 445.2519, found: 445.2517.

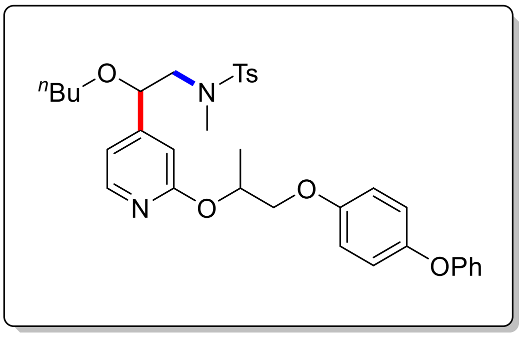

N-(2-butoxy-2-(2-((1-(4-phenoxyphenoxy)propan-2-yl)oxy)pyridin-4-yl)ethyl)-N,4-dimethylbenzenesulfonamide (5f). From 0.1 mmol Pyriproxyfen derivatives : Diastereomeric mixture (1:1) Yield 78%. 47.3 mg. Colorless gum. ^{1}H NMR (600 MHz, CD_2Cl_2) δ 8.12 (d, J = 5.2 Hz, 1H), 7.63 (d, J = 7.9 Hz, 2H), 7.34 – 7.27 (m, 4H), 7.05 (t, J = 7.5 Hz, 1H), 6.99 – 6.90 (m, 6H), 6.85 (d, J = 5.3 Hz, 1H), 6.69 (d, J = 3.1 Hz, 1H), 5.62 – 5.55 (m, 1H), 4.44 (dt, J = 7.0, 3.1 Hz, 1H), 4.18 (dt, J = 11.0, 6.2 Hz, 1H), 4.11 – 4.06 (m, 1H), 3.38 – 3.33 (m, 1H), 3.30 – 3.24 (m, 2H), 3.02 (dd, J = 14.4, 8.4 Hz, 1H), 2.84 (s, 3H), 2.41 (s, 3H), 1.51 (p, J = 7.0 Hz, 2H), 1.46 (t, J = 6.0 Hz, 3H), 1.38 – 1.31 (m, 2H), 0.89 (t, J = 7.3 Hz, 3H). ^{13}C NMR (150 MHz, CD_2Cl_2) δ 164.2, 159.1, 155.8, 152.9, 150.9, 147.6, 144.1, 135.8, 130.3, 130.2, 127.8, 123.0, 121.3, 118.2, 116.3, 115.7, 115.6, 109.8, 109.8, 81.6, 81.6, 71.7, 70.2, 70.1, 70.1, 56.6, 37.6, 32.5, 21.8, 19.9, 17.3, 14.2. HRMS (ESI$^+$) m/z calcd. $C_{34}H_{41}N_2O_6S^+$ $[M+H]^+$: 605.2680, found: 605.2678.

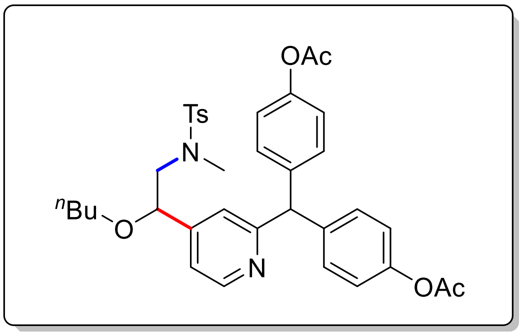

((4-(1-butoxy-2-((N,4-dimethylphenyl)sulfonamido)ethyl)pyridin-2-yl)methylene)bis(4,1-phenylene) diacetate (5g). From 0.1 mmol Bisacodyl derivatives : Yield 43%. 27.5 mg. yellow solid. mp 60-62 ºC. ^{1}H NMR (600 MHz, CD_2Cl_2) δ 8.56 (d, J = 5.0 Hz, 1H), 7.60 (d, J = 8.5 Hz, 2H), 7.31 (d, J = 7.9 Hz, 2H), 7.24 (dd, J = 8.5, 4.4 Hz, 4H), 7.16 (s, 1H), 7.14 (d, J = 5.1 Hz, 1H), 7.02 (d, J = 8.9 Hz, 4H), 5.63 (s, 1H), 4.46 (dd, J = 8.1, 4.1 Hz, 1H), 3.28 (t, J = 6.6 Hz, 2H), 3.23 (d, J = 4.1 Hz, 1H), 3.04 (dd, J = 14.4, 8.0 Hz, 1H), 2.78 (s, 3H), 2.41 (s, 3H), 2.26 (s, 6H), 1.47 (dq, J = 13.5, 6.8 Hz, 2H), 1.29 (dp, J = 21.2, 6.9 Hz, 2H), 0.86 (t, J = 7.3 Hz, 3H). ^{13}C NMR (150 MHz, CD_2Cl_2) δ 170.0, 163.3, 150.4, 150.3, 150.0, 150.0, 144.1, 140.9, 140.9, 135.7, 130.8, 130.8, 130.3, 127.8, 122.2, 122.1, 122.1, 120.4, 81.5, 70.2, 58.5, 56.7, 37.6, 32.4, 21.8, 21.5, 19.9, 14.2. HRMS (ESI$^+$) m/z calcd. $C_{36}H_{41}N_2O_7S^+$ $[M+H]^+$: 645.2629, found: 645.2628.

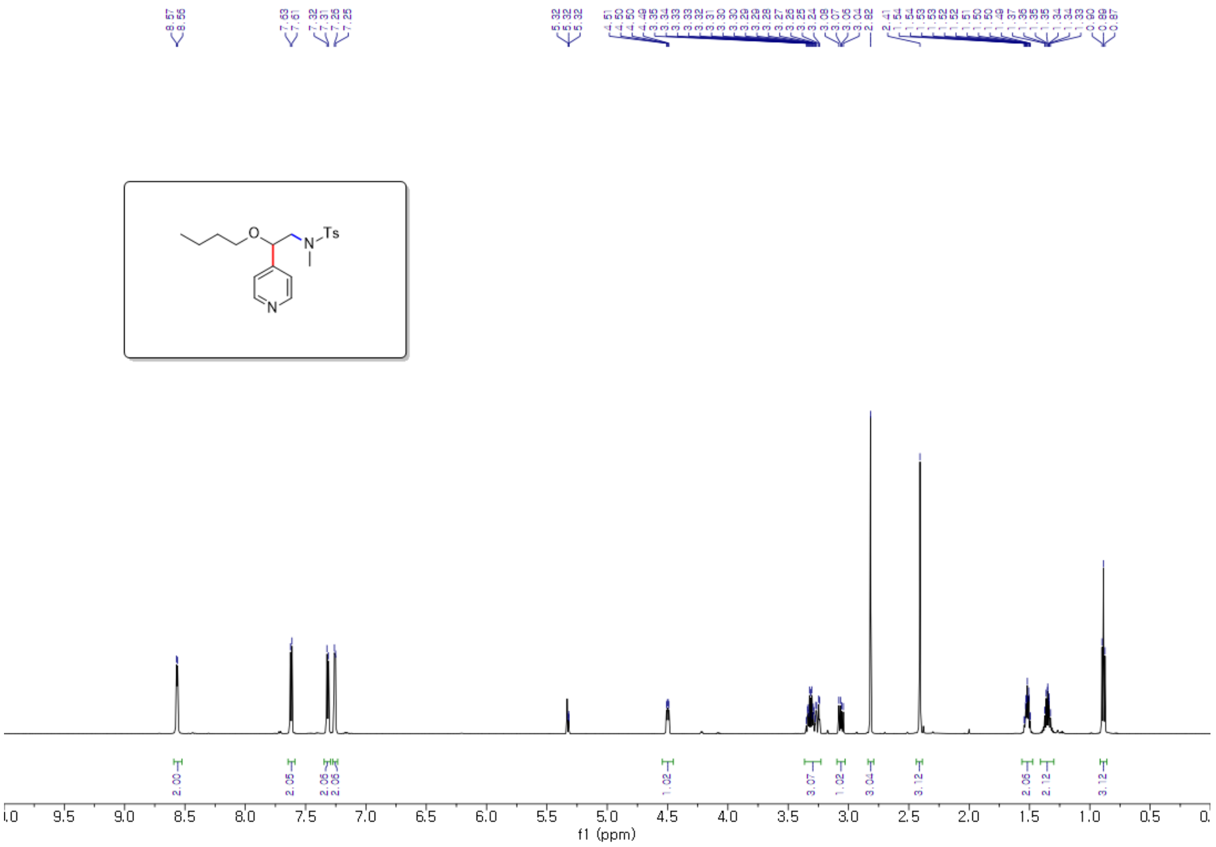

600 MHz, ^{1}H NMR in CD_2Cl_2

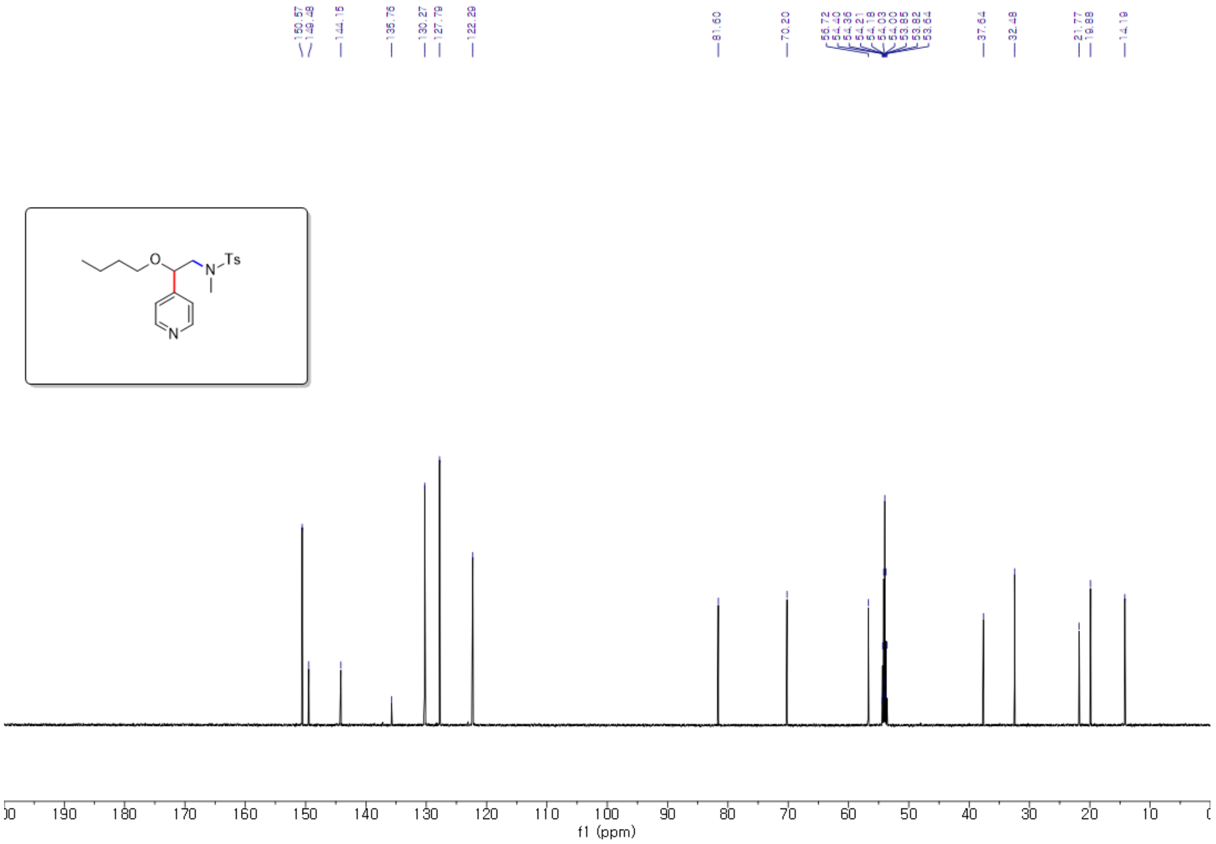

150 MHz, ^{13}C NMR in CD_2Cl_2

Supplementary Figure 20. 1H and 13C NMR of **3a**

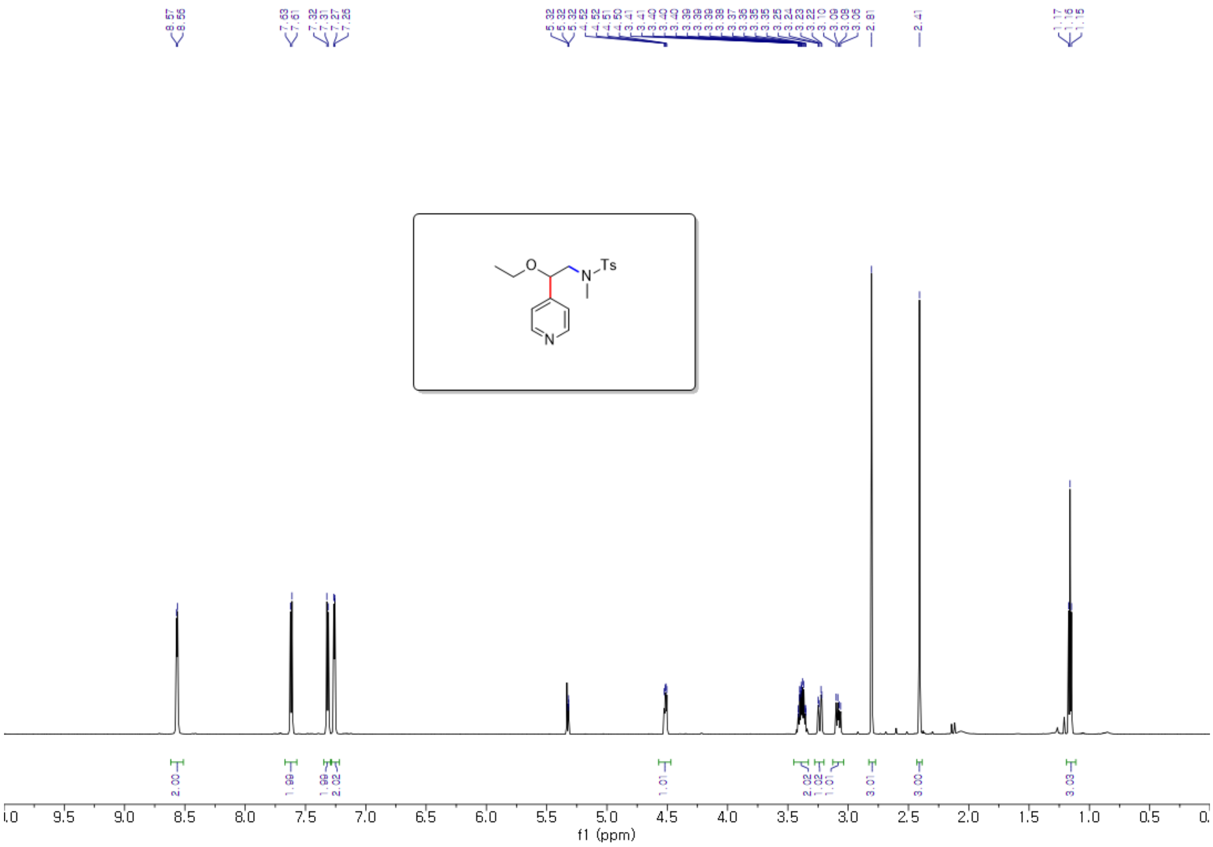

600 MHz, ^{1}H NMR in CD_2Cl_2

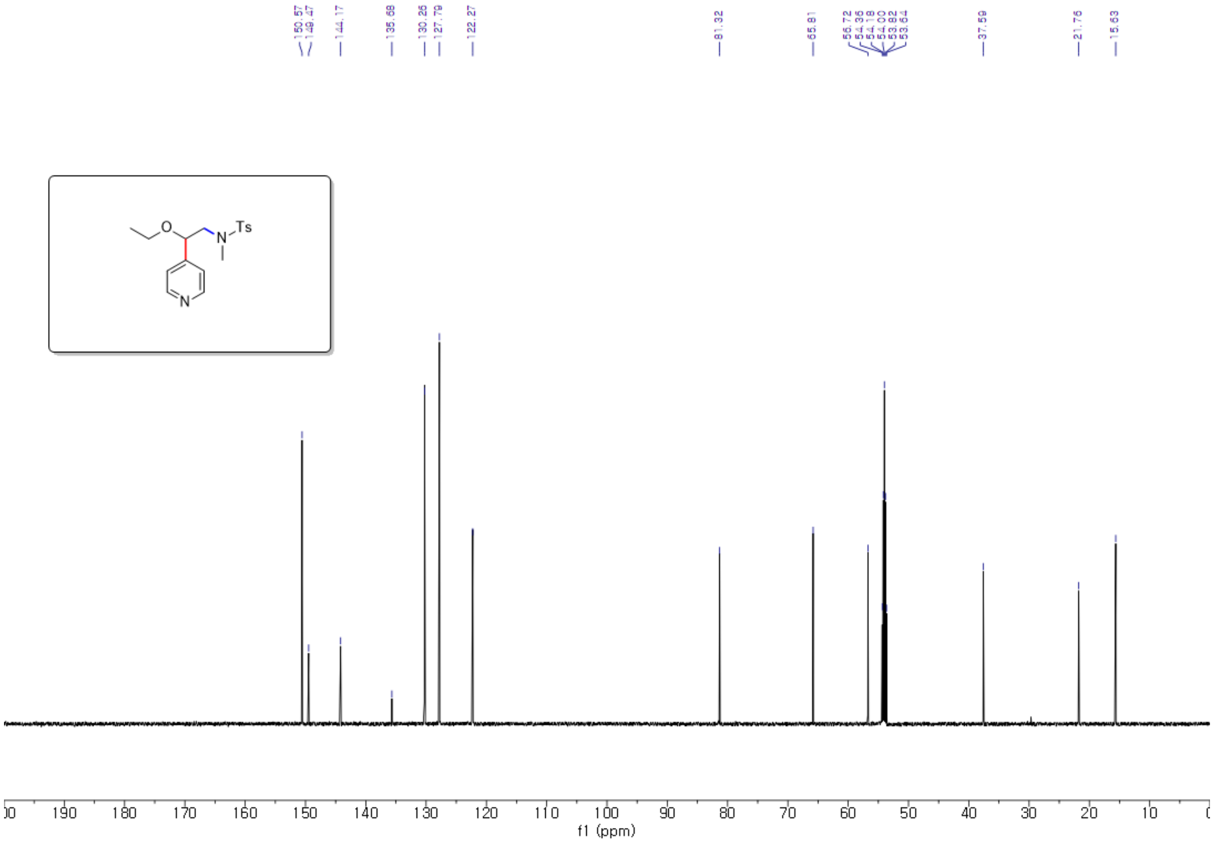

150 MHz, ^{13}C NMR in CD_2Cl_2

Supplementary Figure 21. 1H and 13C NMR of **3b**

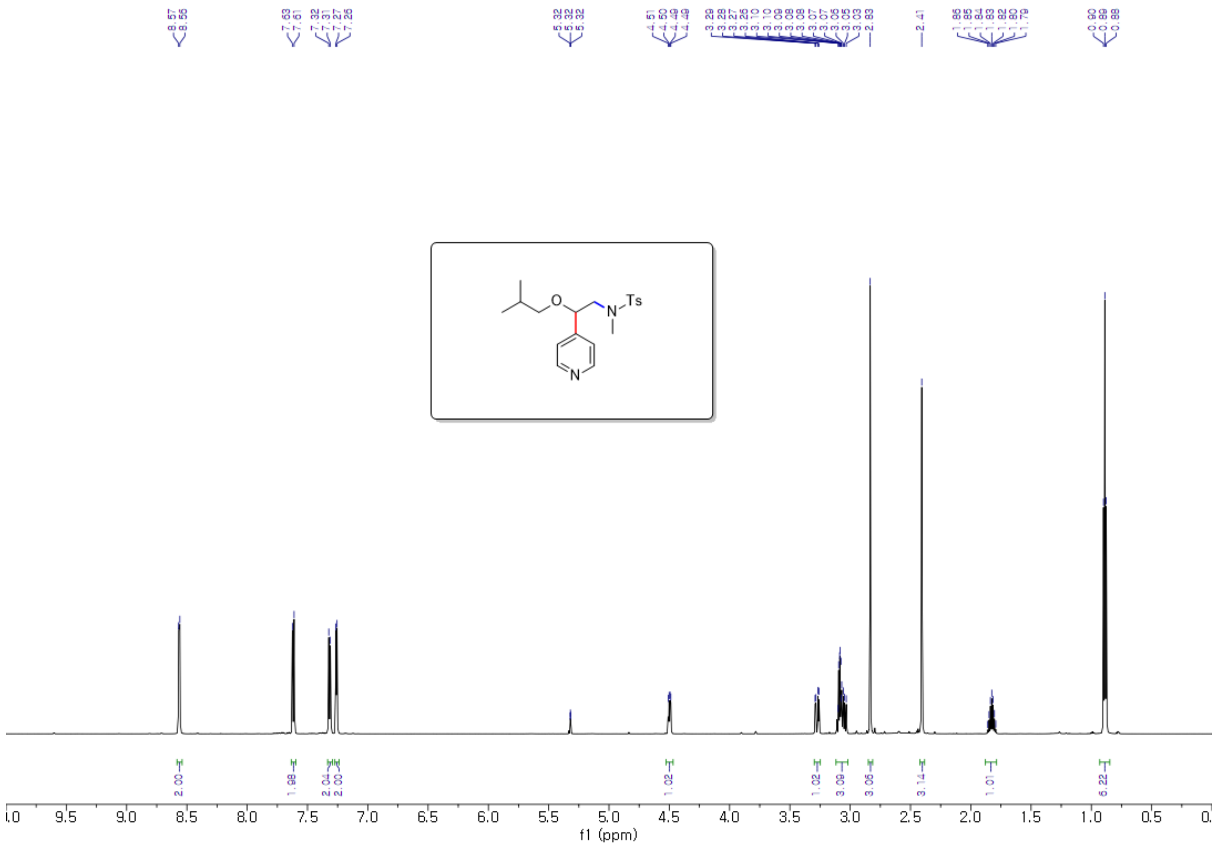

600 MHz, ^{1}H NMR in CD_2Cl_2

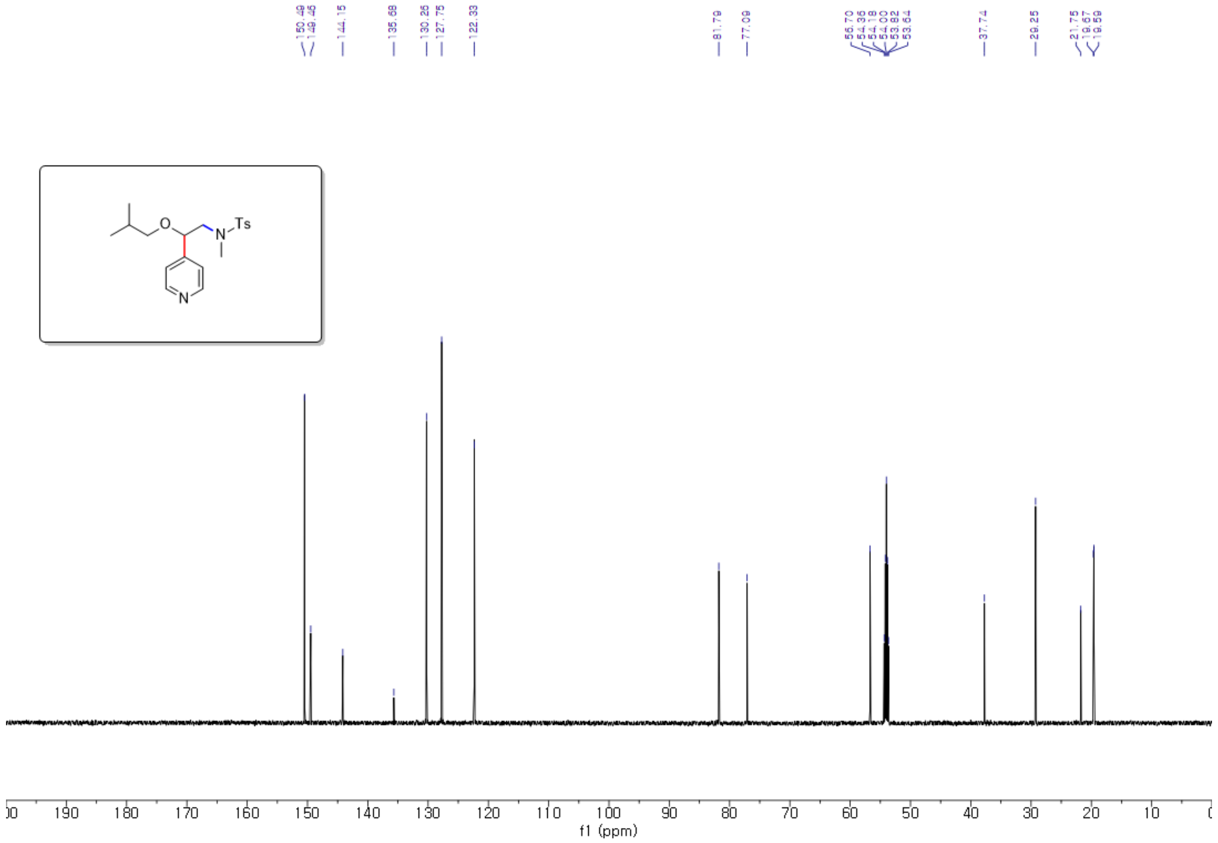

150 MHz, ^{13}C NMR in CD_2Cl_2

Supplementary Figure 22. 1H and 13C NMR of **3c**

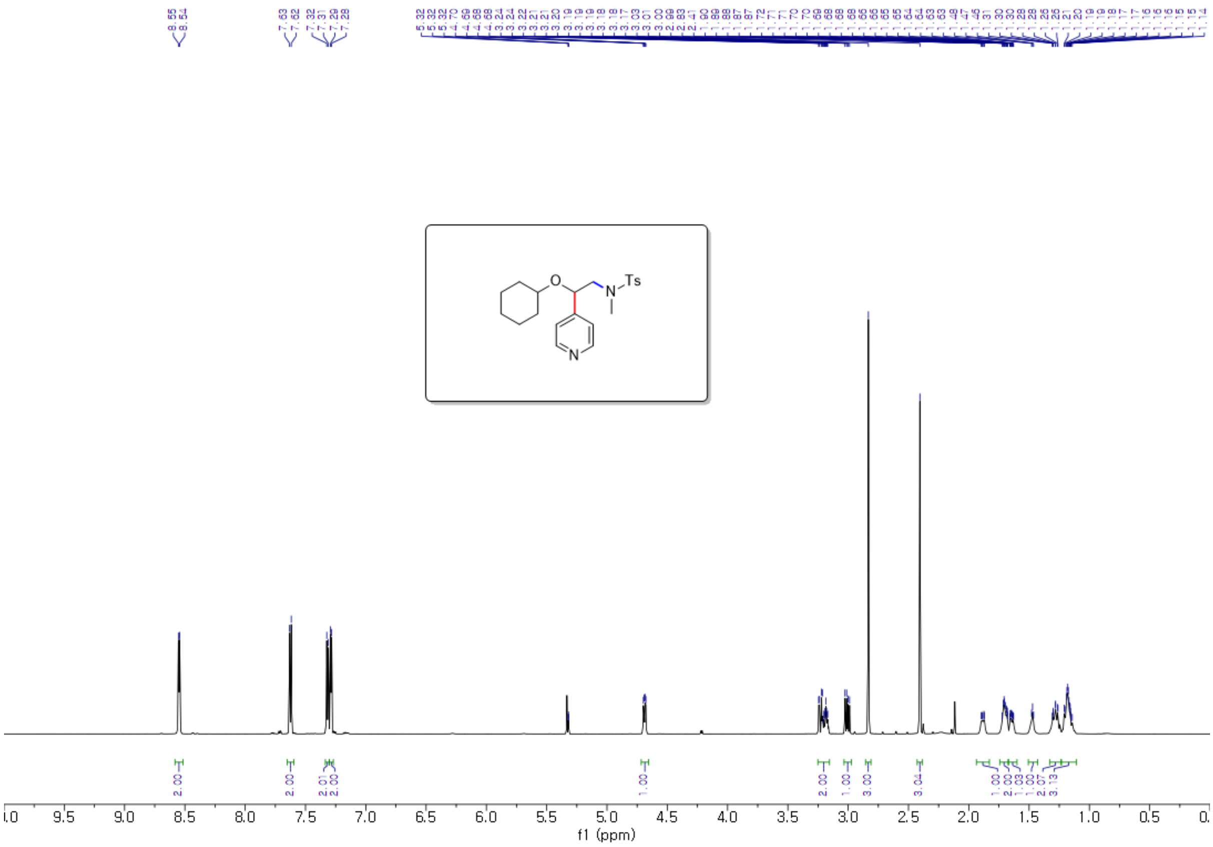

600 MHz, ^{1}H NMR in CD_2Cl_2

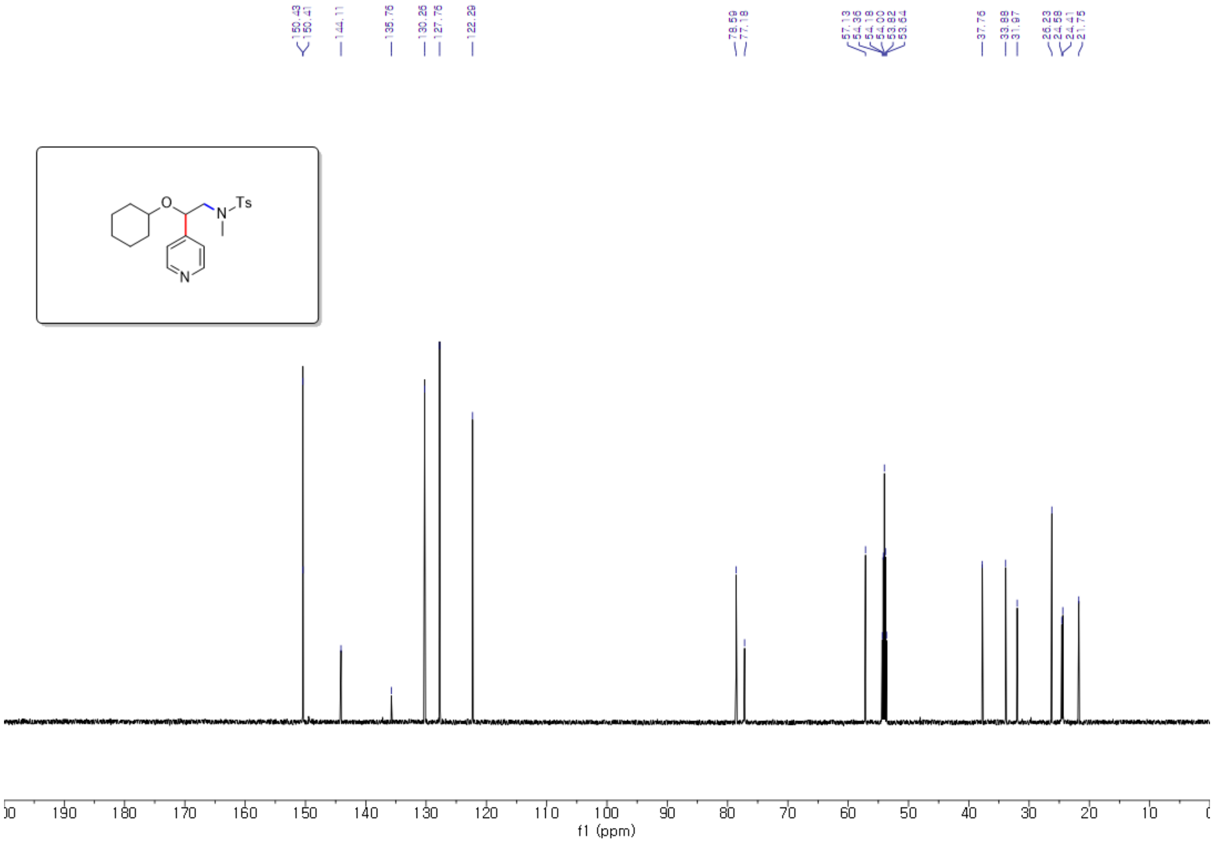

150 MHz, ^{13}C NMR in CD_2Cl_2

Supplementary Figure 23. 1H and 13C NMR of **3d**

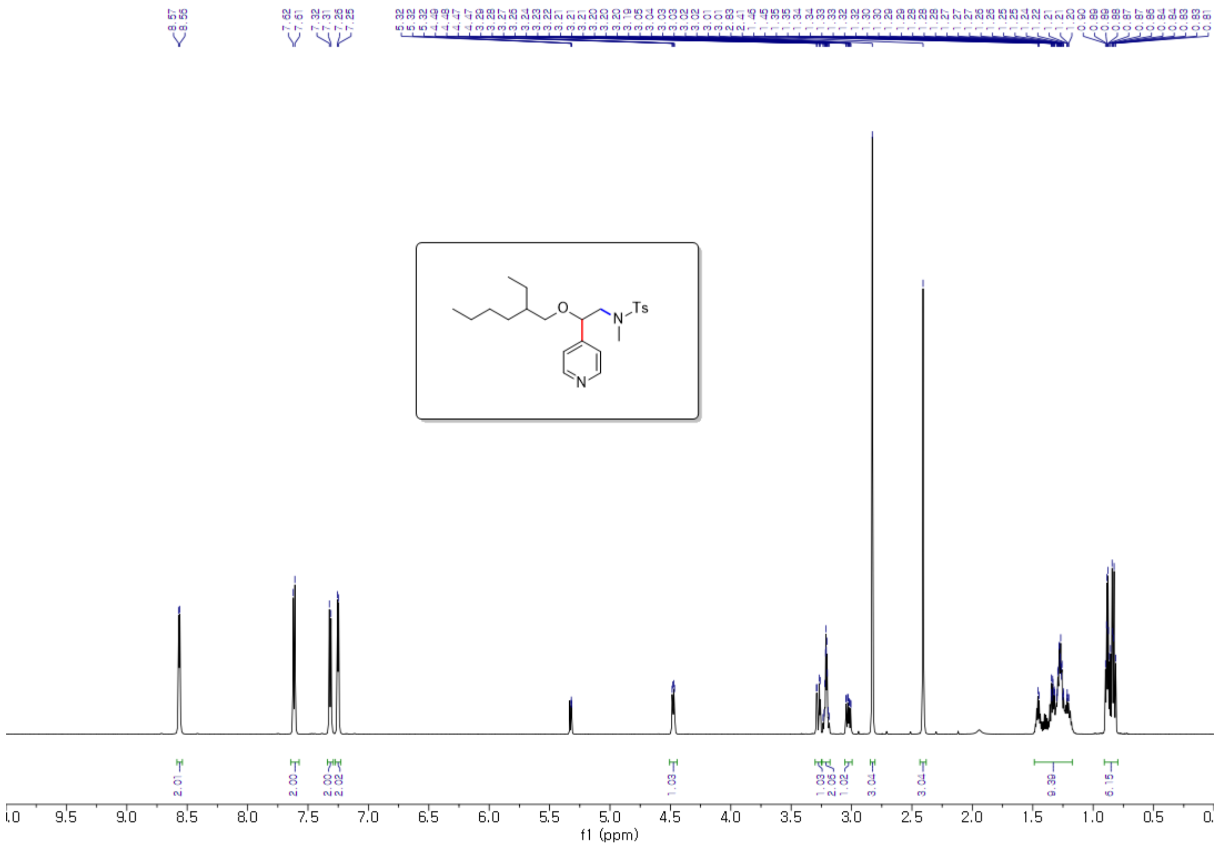

600 MHz, ^{1}H NMR in CD_2Cl_2

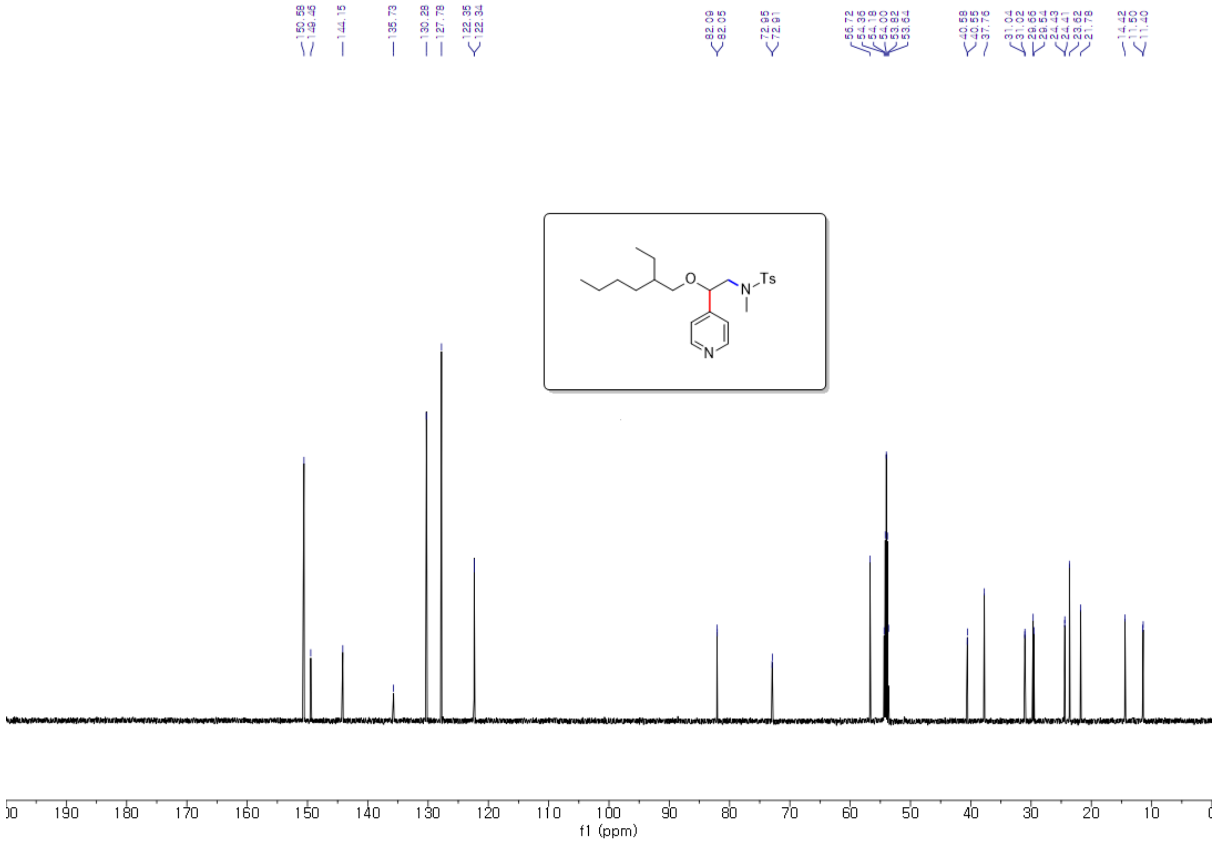

150 MHz, ^{13}C NMR in CD_2Cl_2

Supplementary Figure 24. 1H and 13C NMR of **3e**

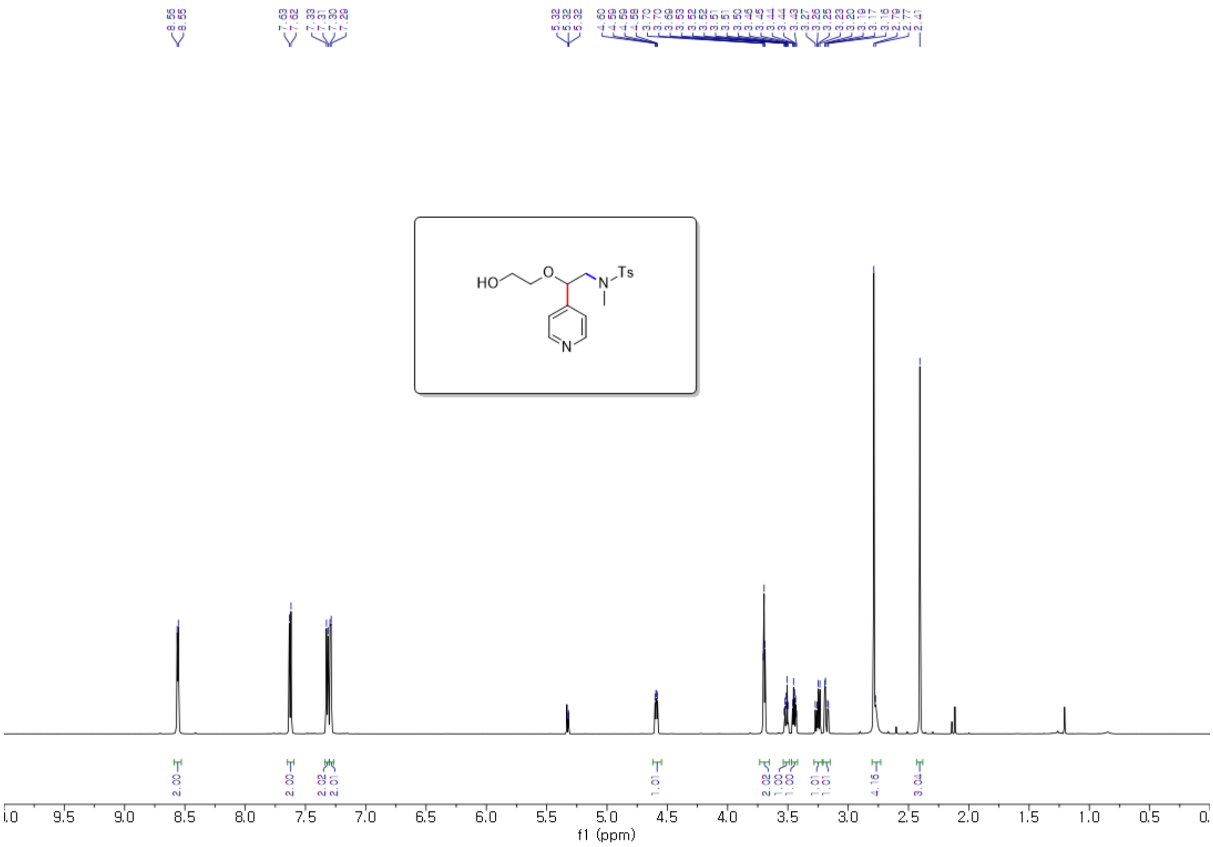

600 MHz, ^{1}H NMR in CD_2Cl_2

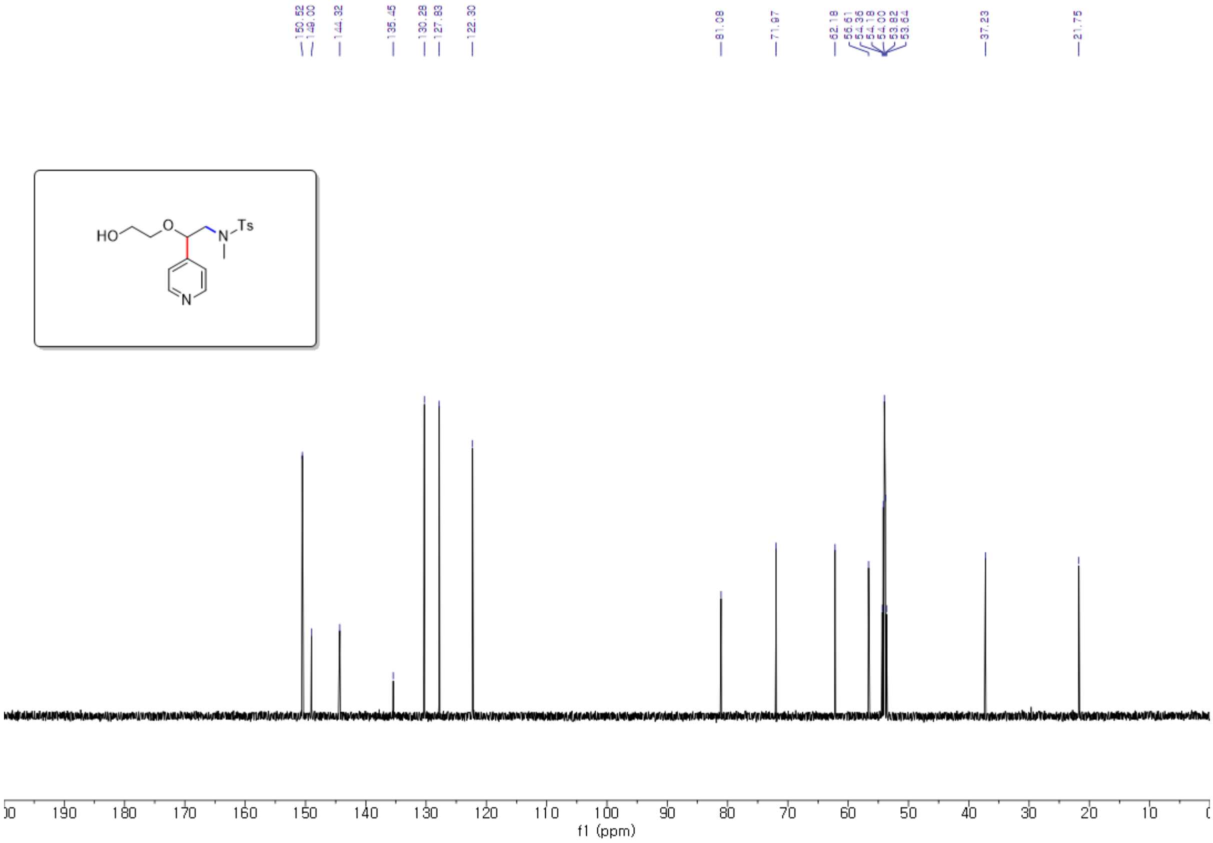

150 MHz, ^{13}C NMR in CD_2Cl_2

Supplementary Figure 25. 1H and 13C NMR of **3f**

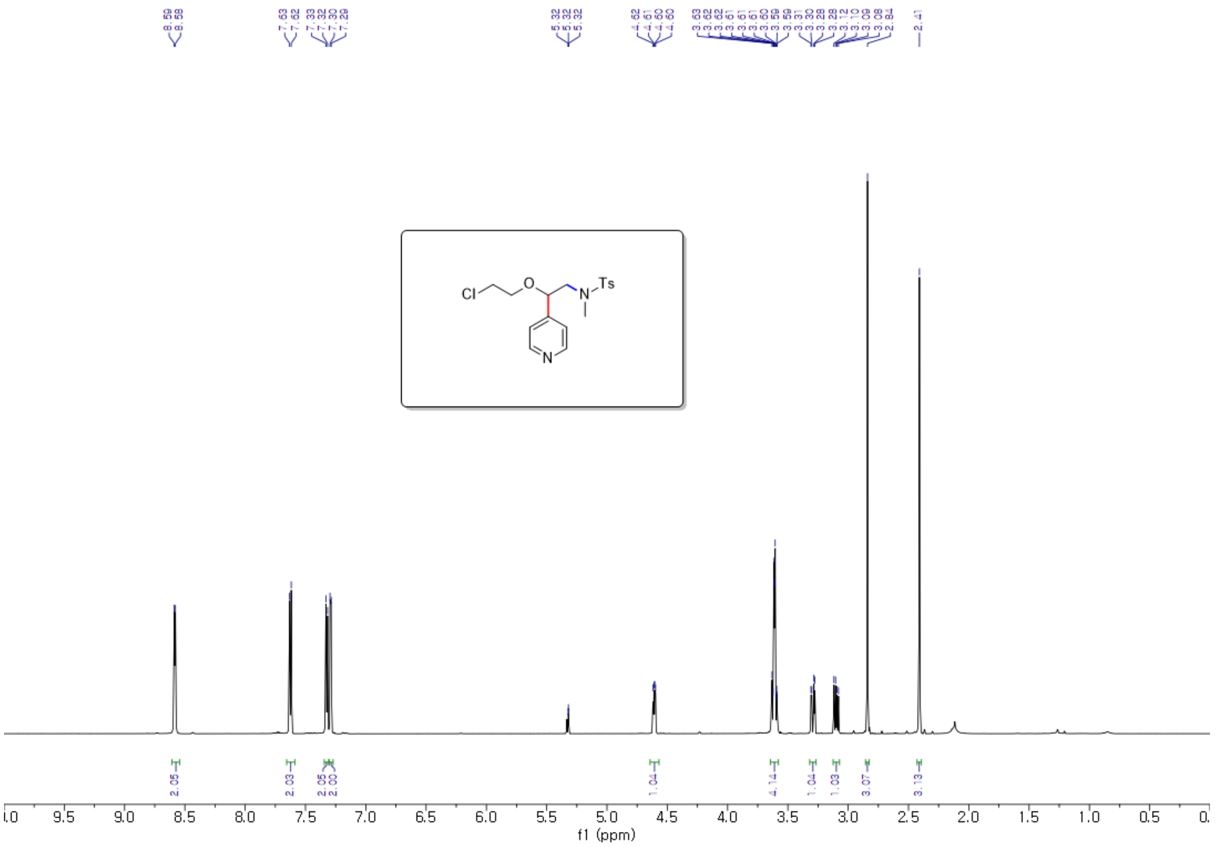

600 MHz, ^{1}H NMR in CD_2Cl_2

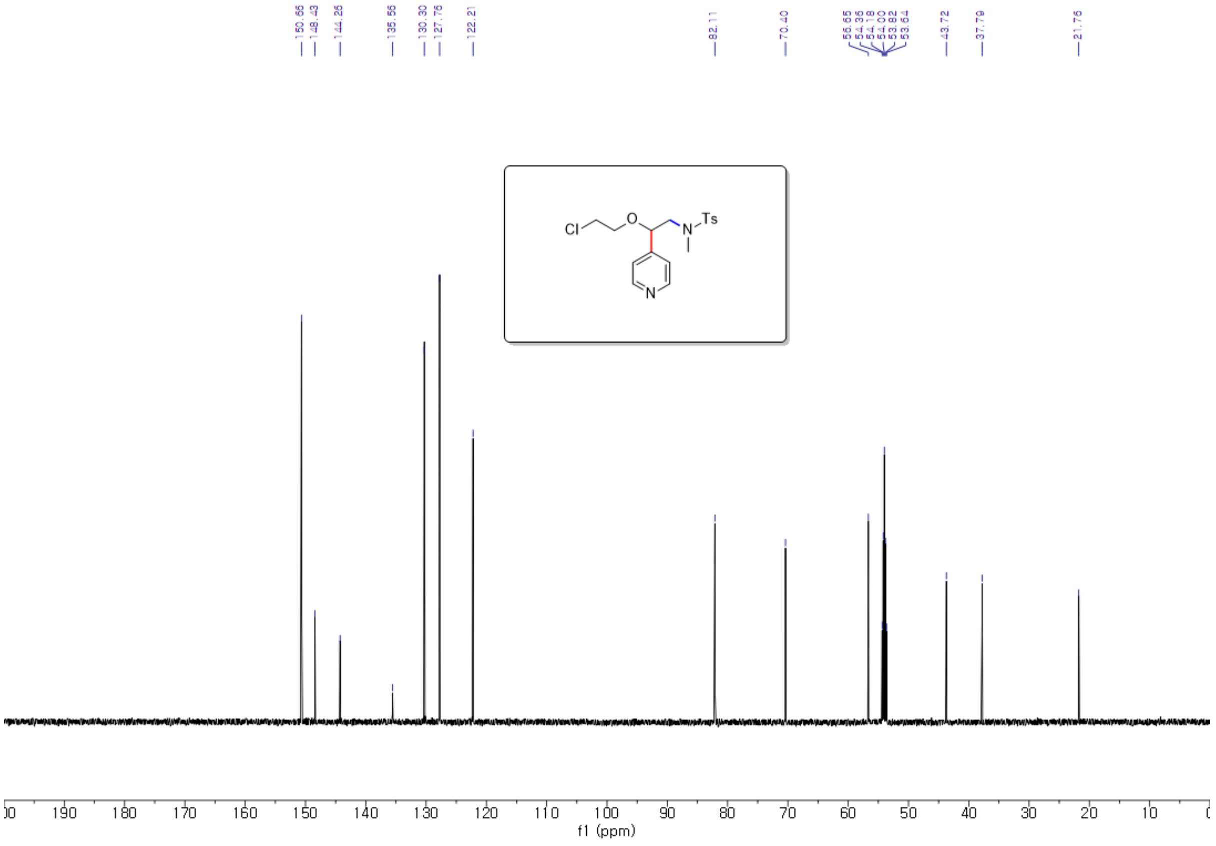

150 MHz, ^{13}C NMR in CD_2Cl_2

Supplementary Figure 26. 1H and 13C NMR of **3g**

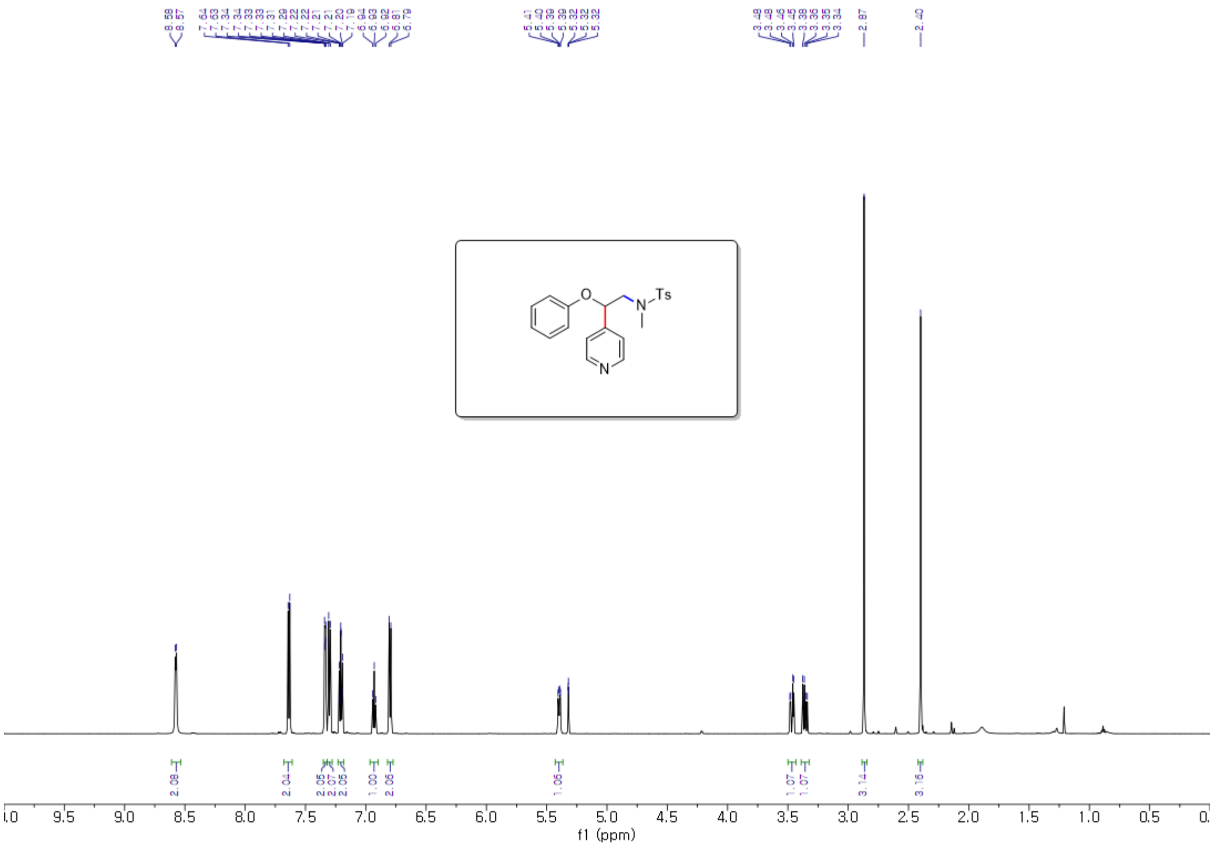

600 MHz, ^{1}H NMR in CD_2Cl_2

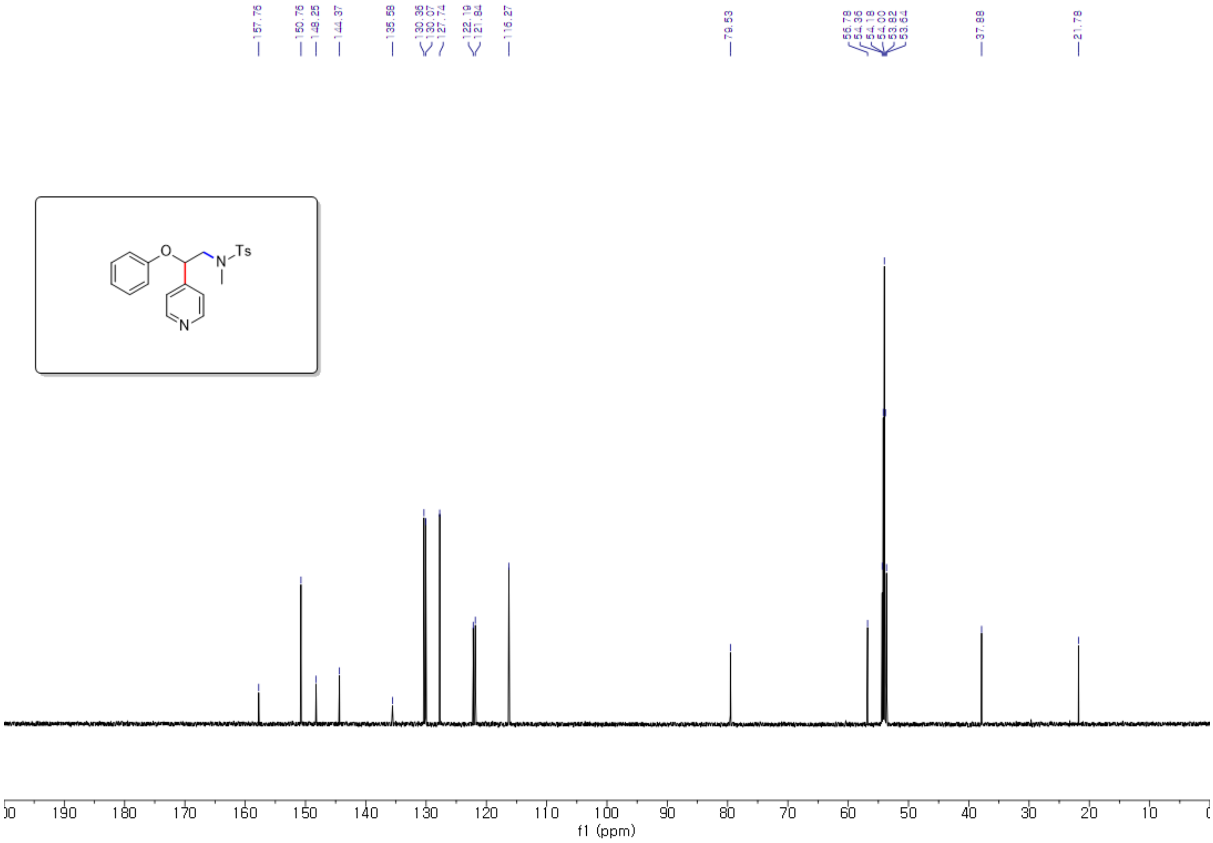

150 MHz, ^{13}C NMR in CD_2Cl_2

Supplementary Figure 27. 1H and 13C NMR of **3h**

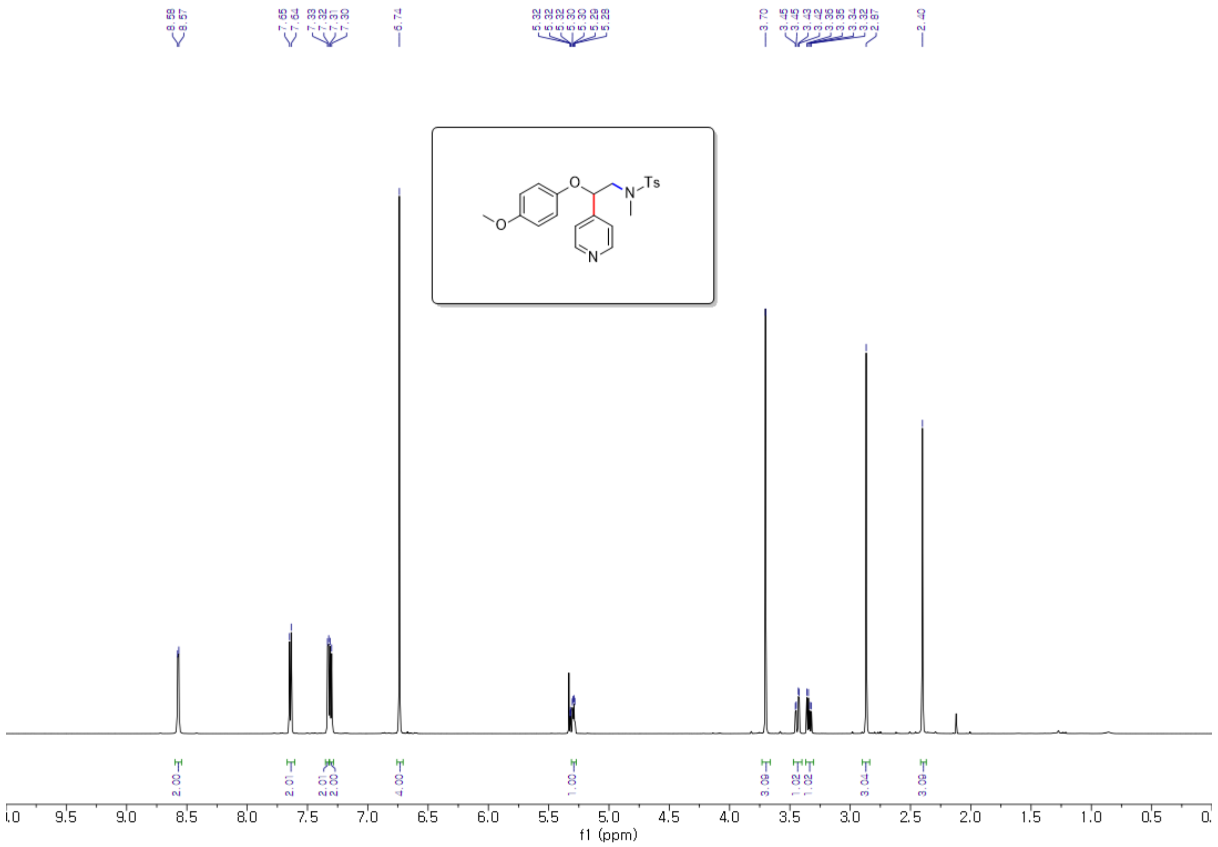

600 MHz, ^{1}H NMR in CD_2Cl_2

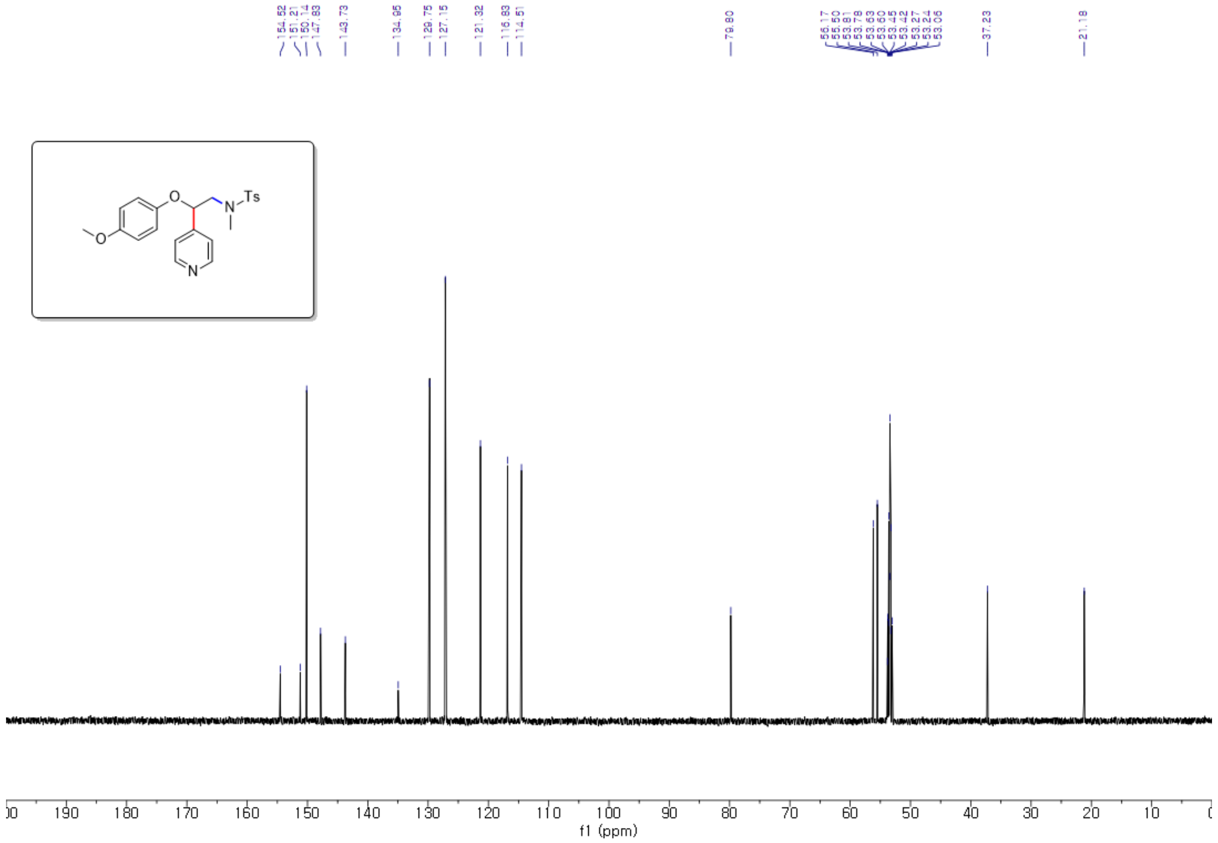

150 MHz, ^{13}C NMR in CD_2Cl_2

Supplementary Figure 28. 1H and 13C NMR of **3i**

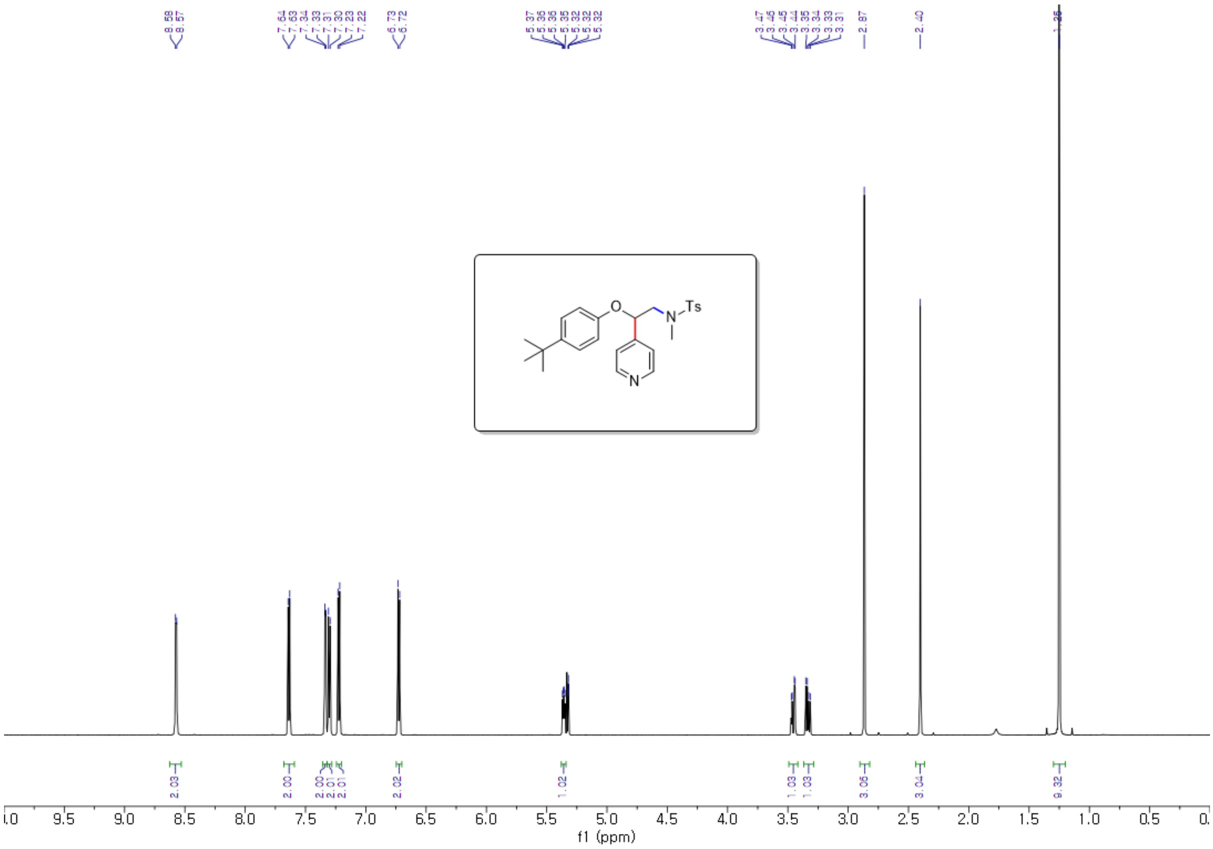

600 MHz, ^{1}H NMR in CD_2Cl_2

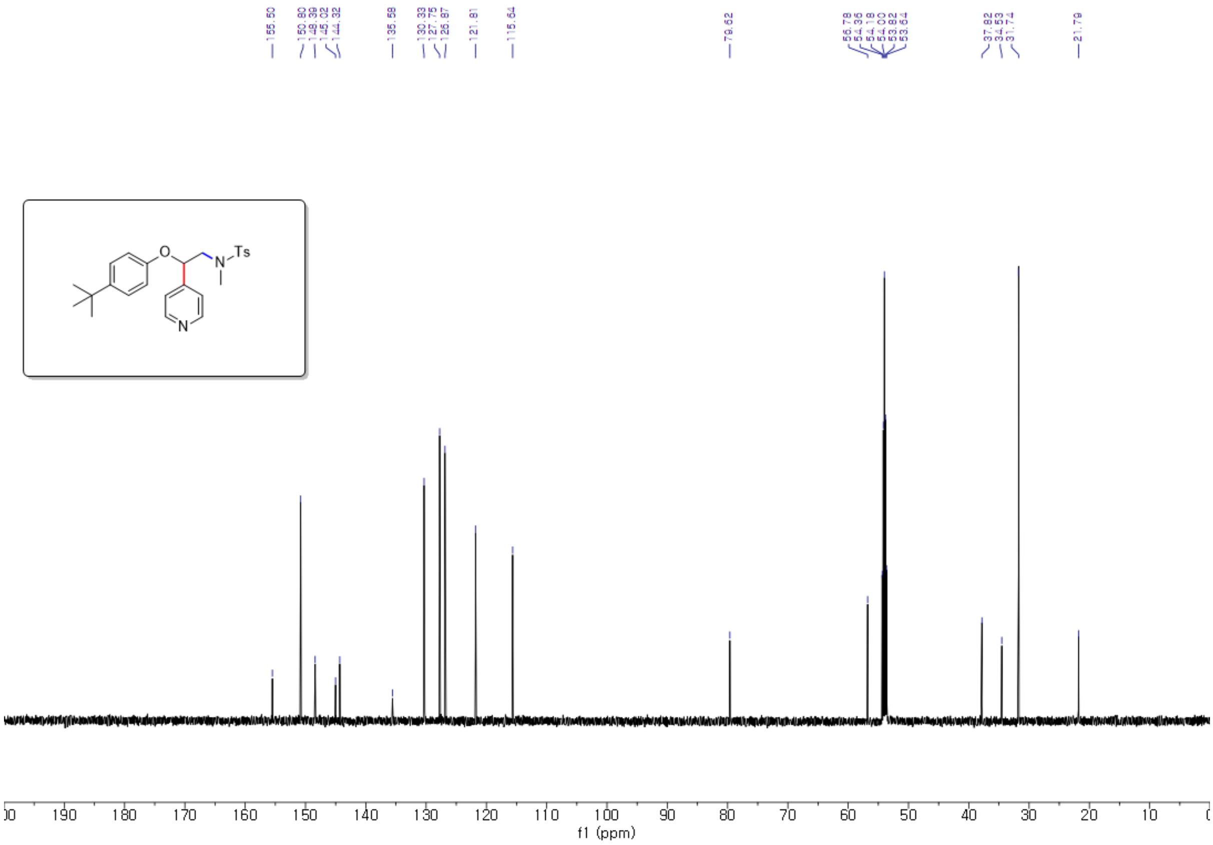

150 MHz, ^{13}C NMR in CD_2Cl_2

Supplementary Figure 29. 1H and 13C NMR of **3j**

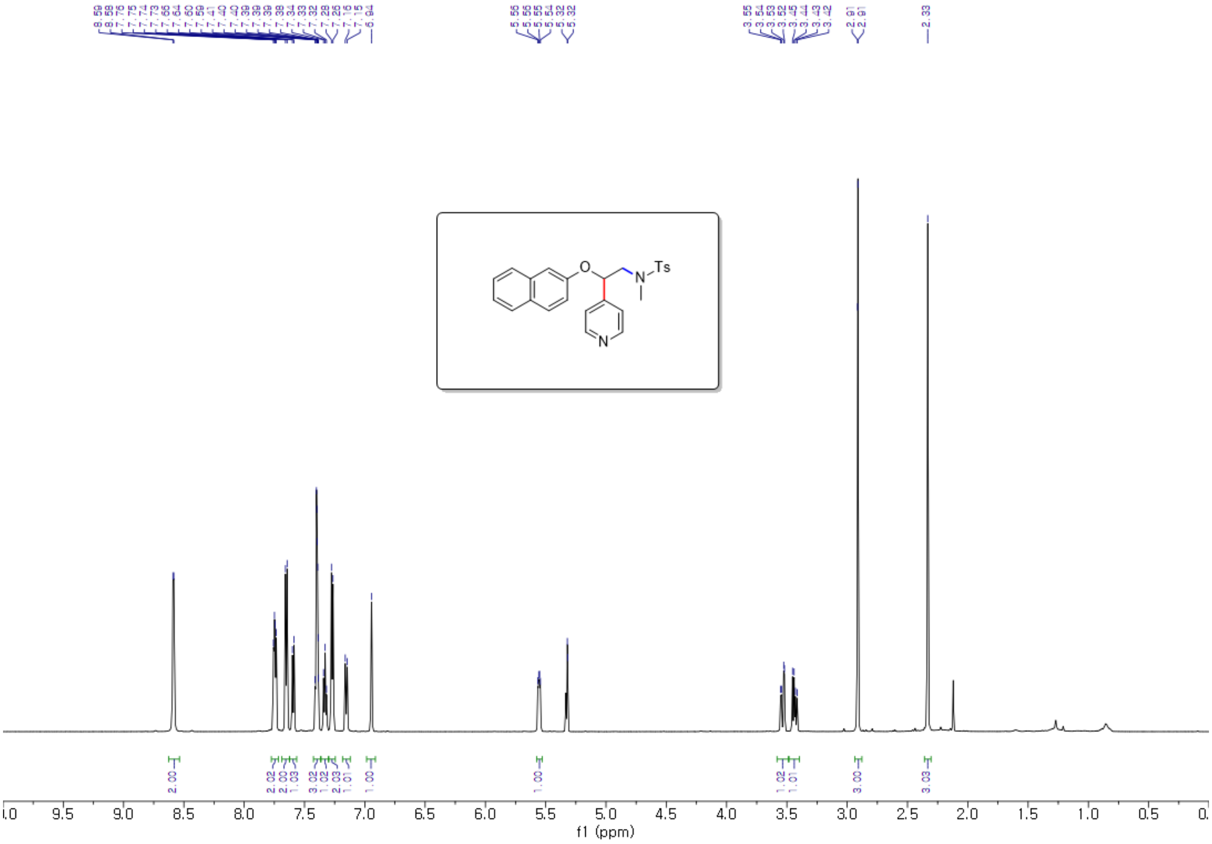

600 MHz, ^{1}H NMR in CD_2Cl_2

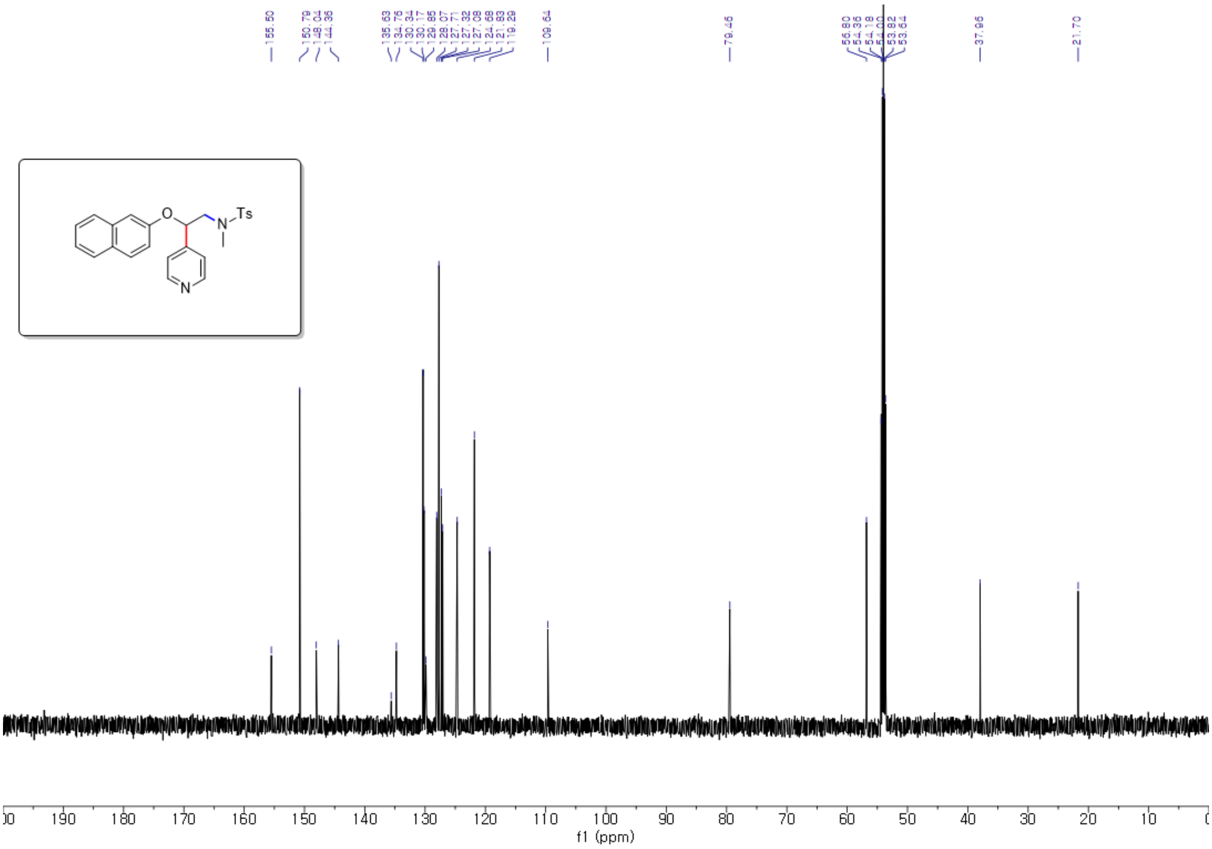

150 MHz, ^{13}C NMR in CD_2Cl_2

Supplementary Figure 30. 1H and 13C NMR of **3k**

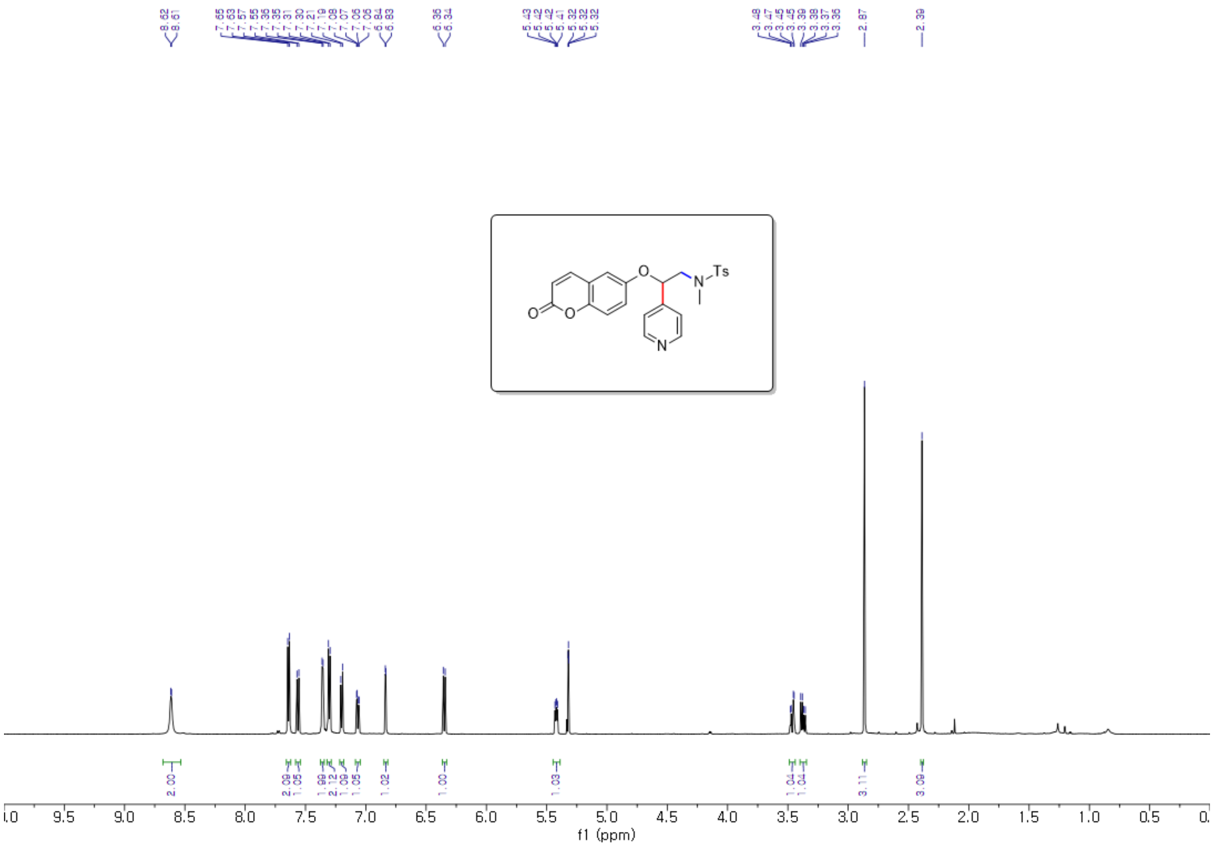

600 MHz, ^{1}H NMR in CD_2Cl_2

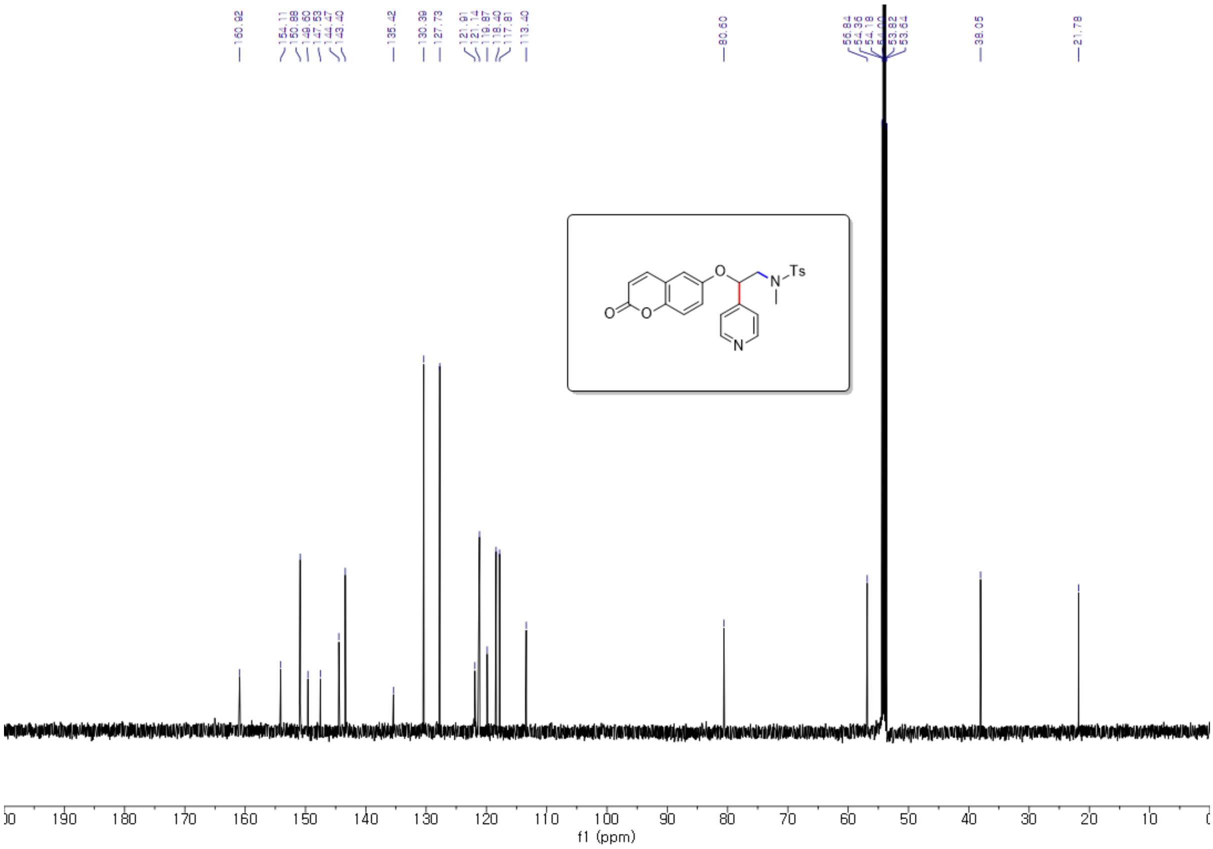

150 MHz, ^{13}C NMR in CD_2Cl_2

Supplementary Figure 31. 1H and 13C NMR of **3l**

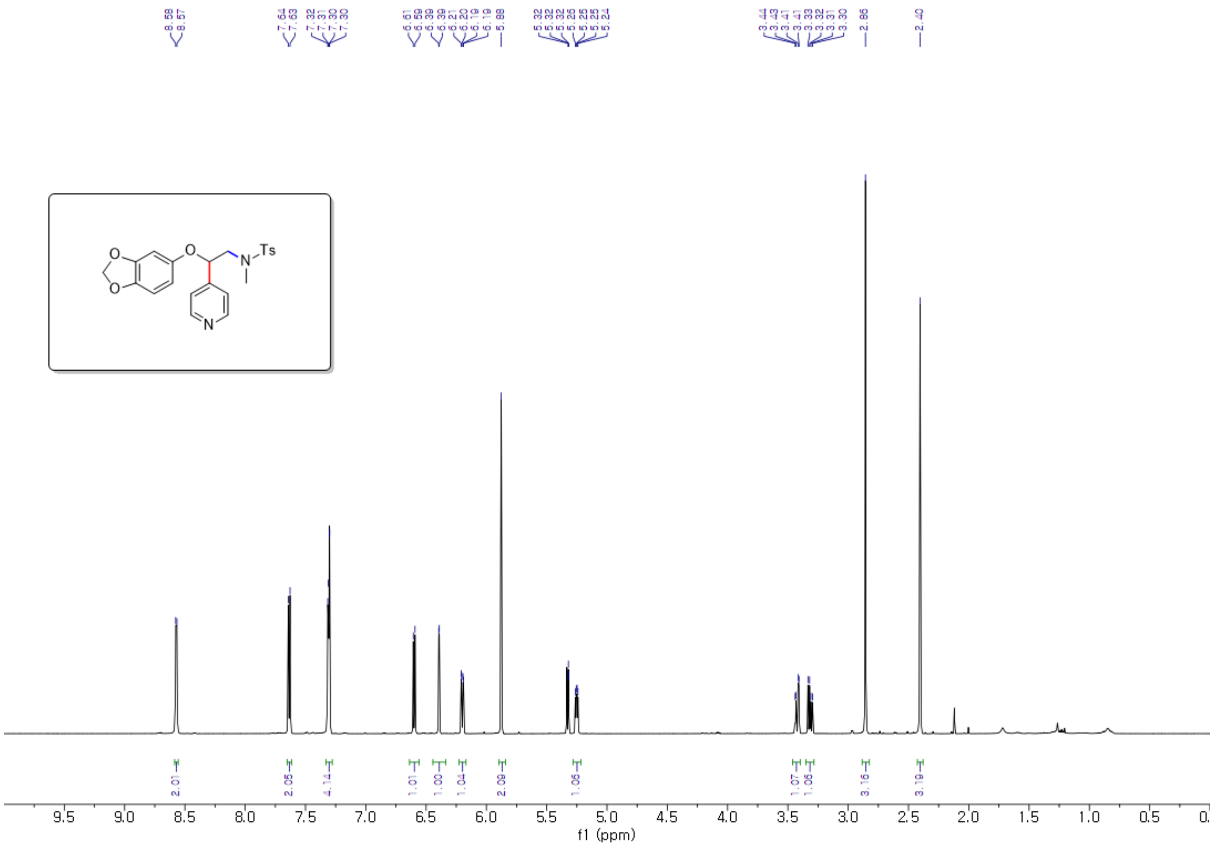

600 MHz, ^{1}H NMR in CD_2Cl_2

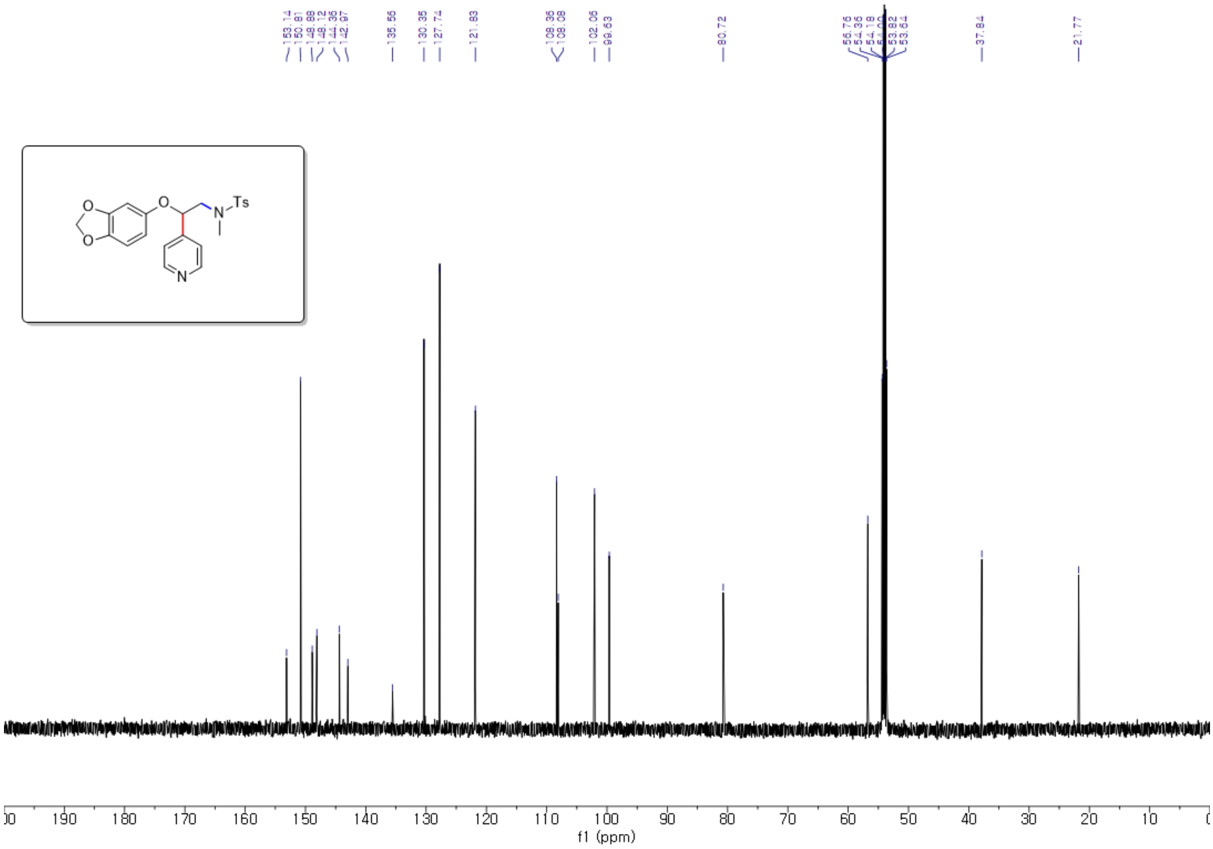

150 MHz, ^{13}C NMR in CD_2Cl_2

Supplementary Figure 32. 1H and 13C NMR of **3m**

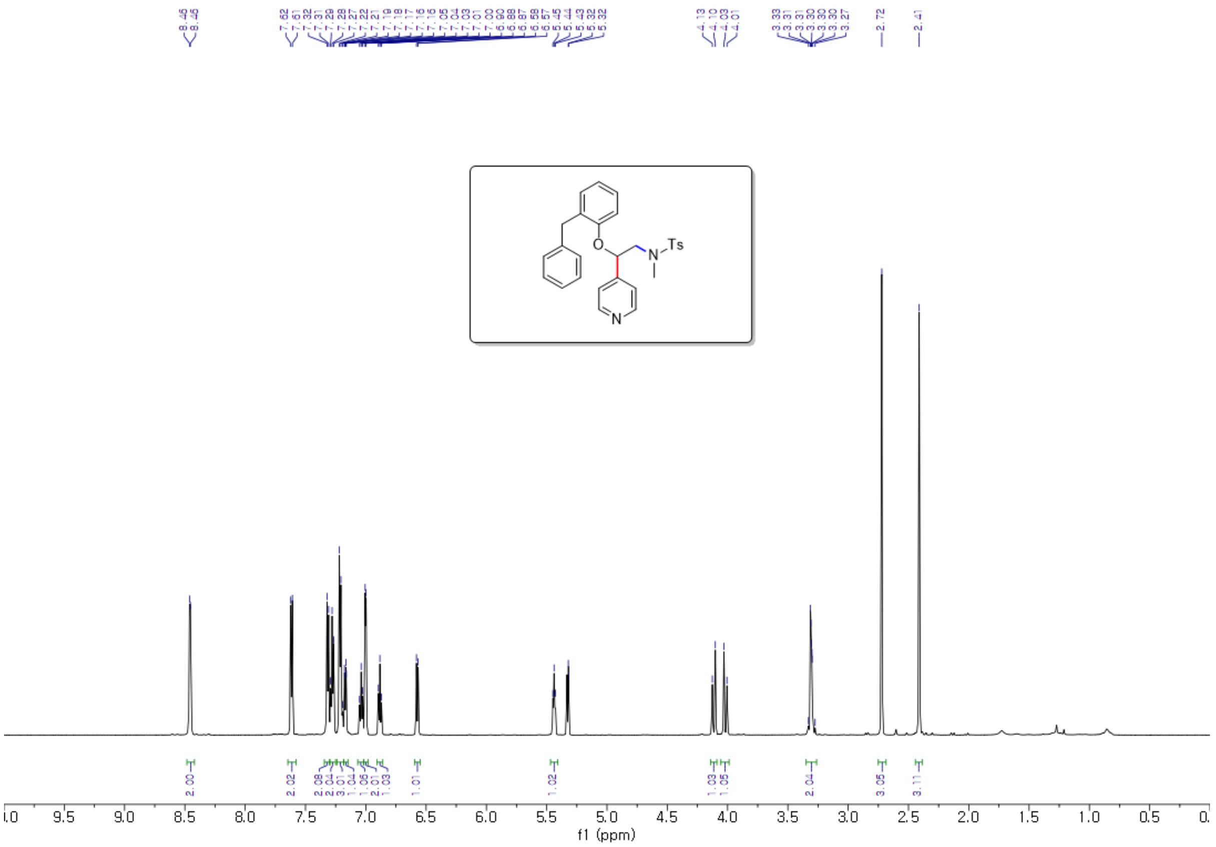

600 MHz, ^{1}H NMR in CD_2Cl_2

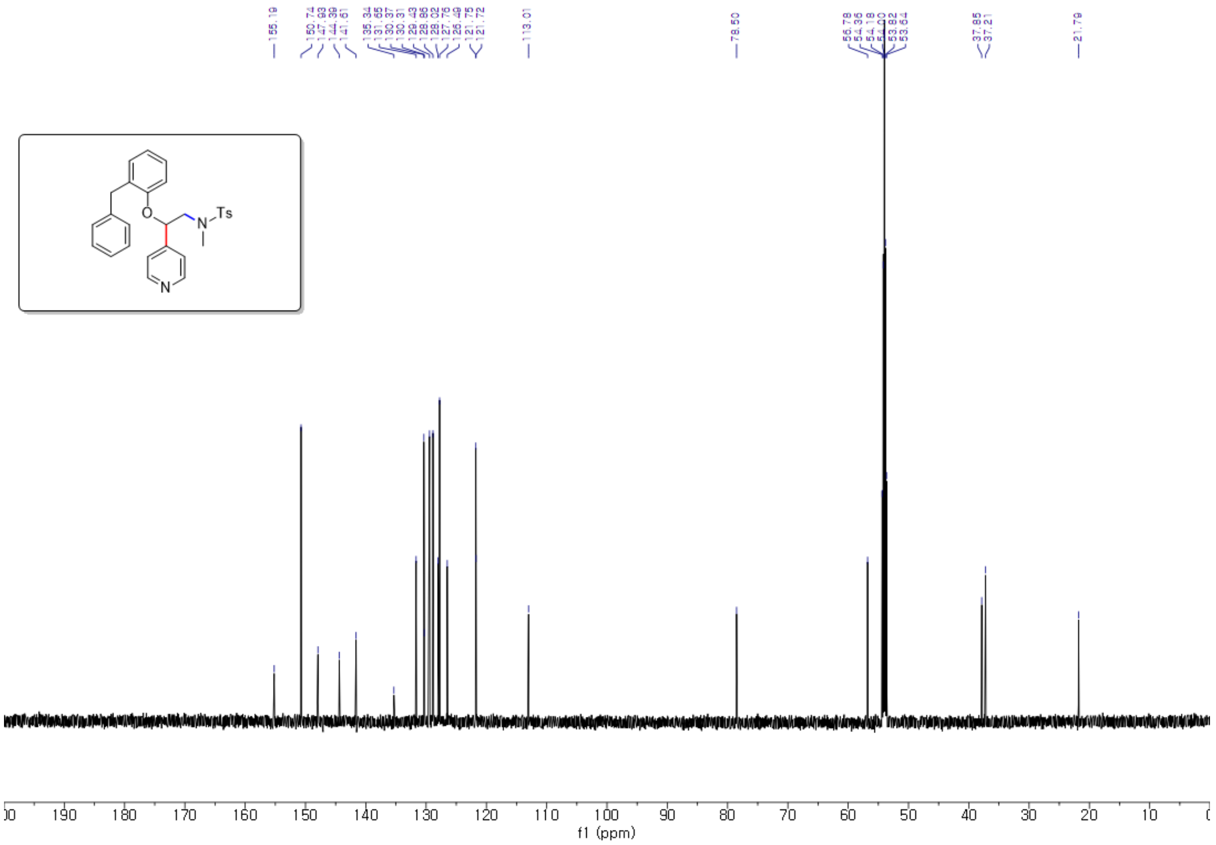

150 MHz, ^{13}C NMR in CD_2Cl_2

Supplementary Figure 33. 1H and 13C NMR of **3n**

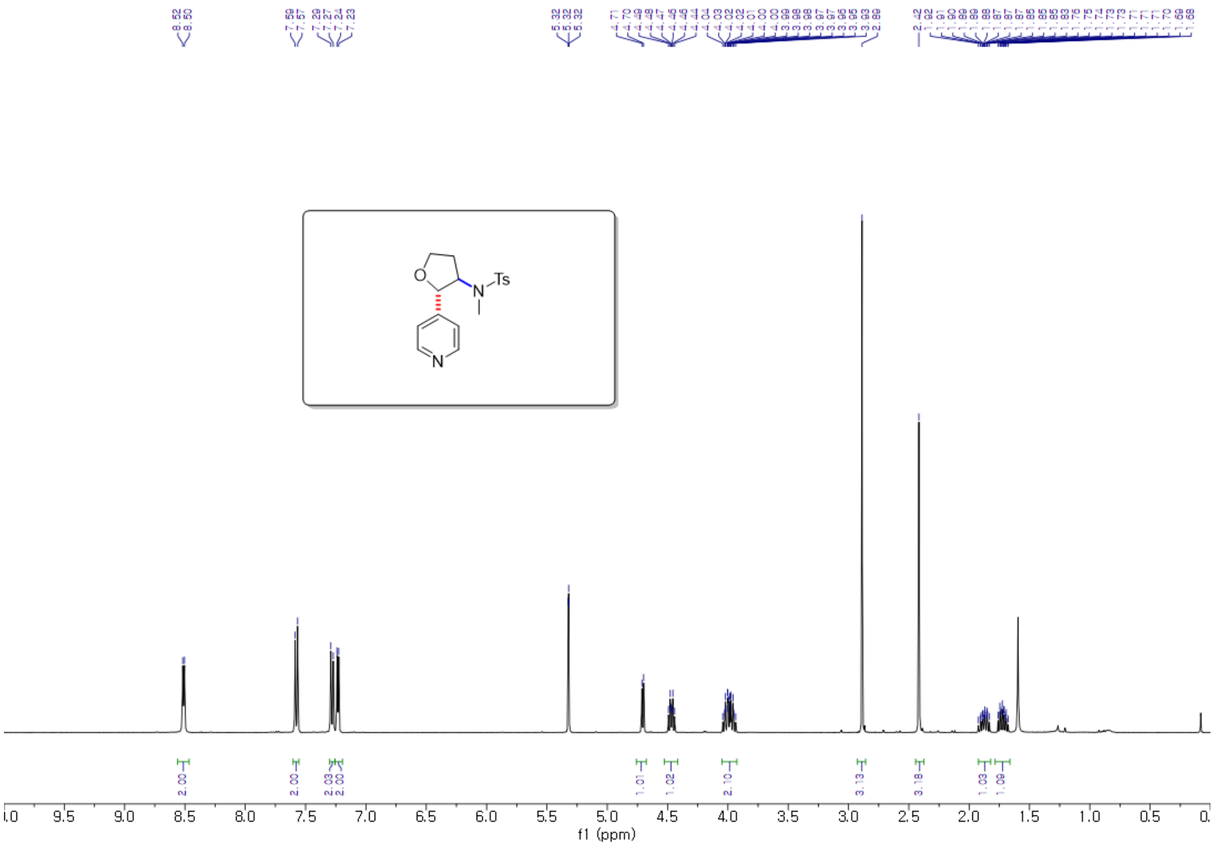

400 MHz, ^{1}H NMR in CD_2Cl_2

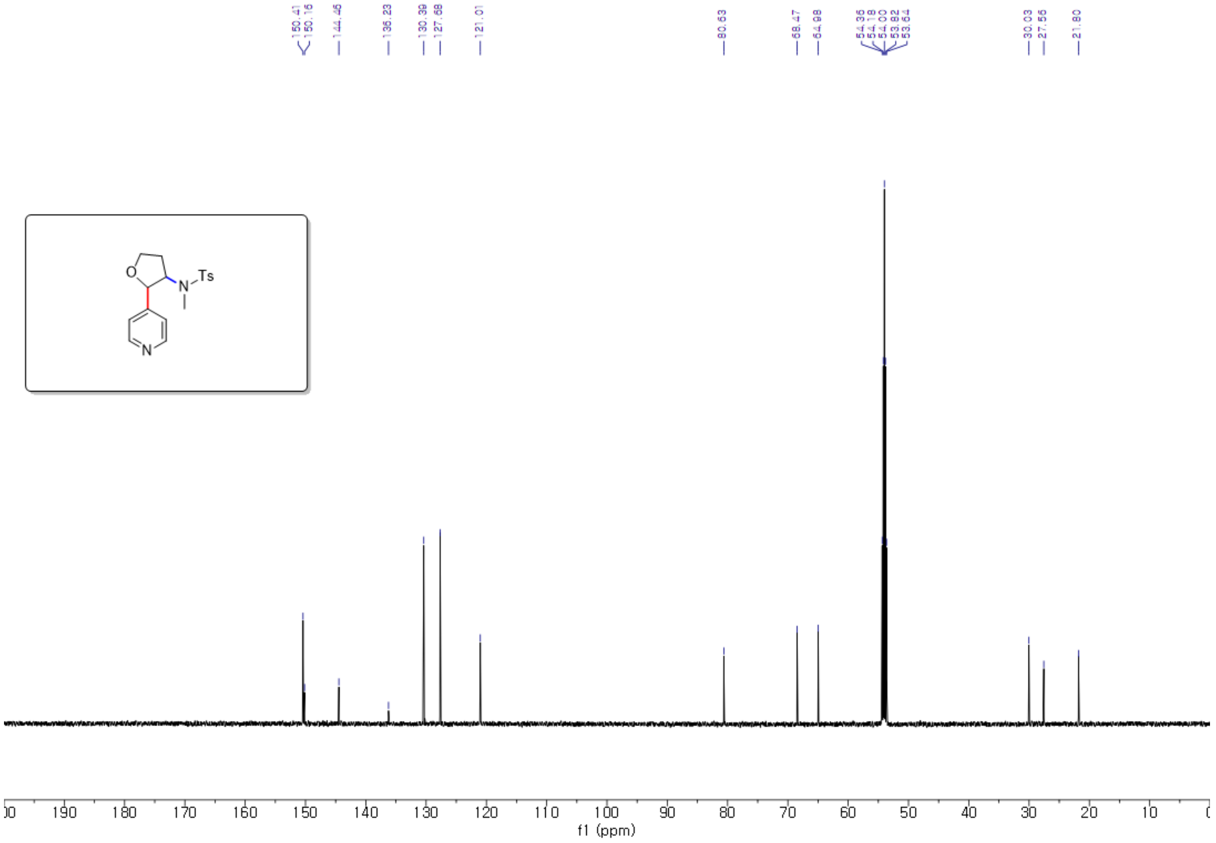

150 MHz, ^{13}C NMR in CD_2Cl_2

Supplementary Figure 34. 1H and 13C NMR of **3o**

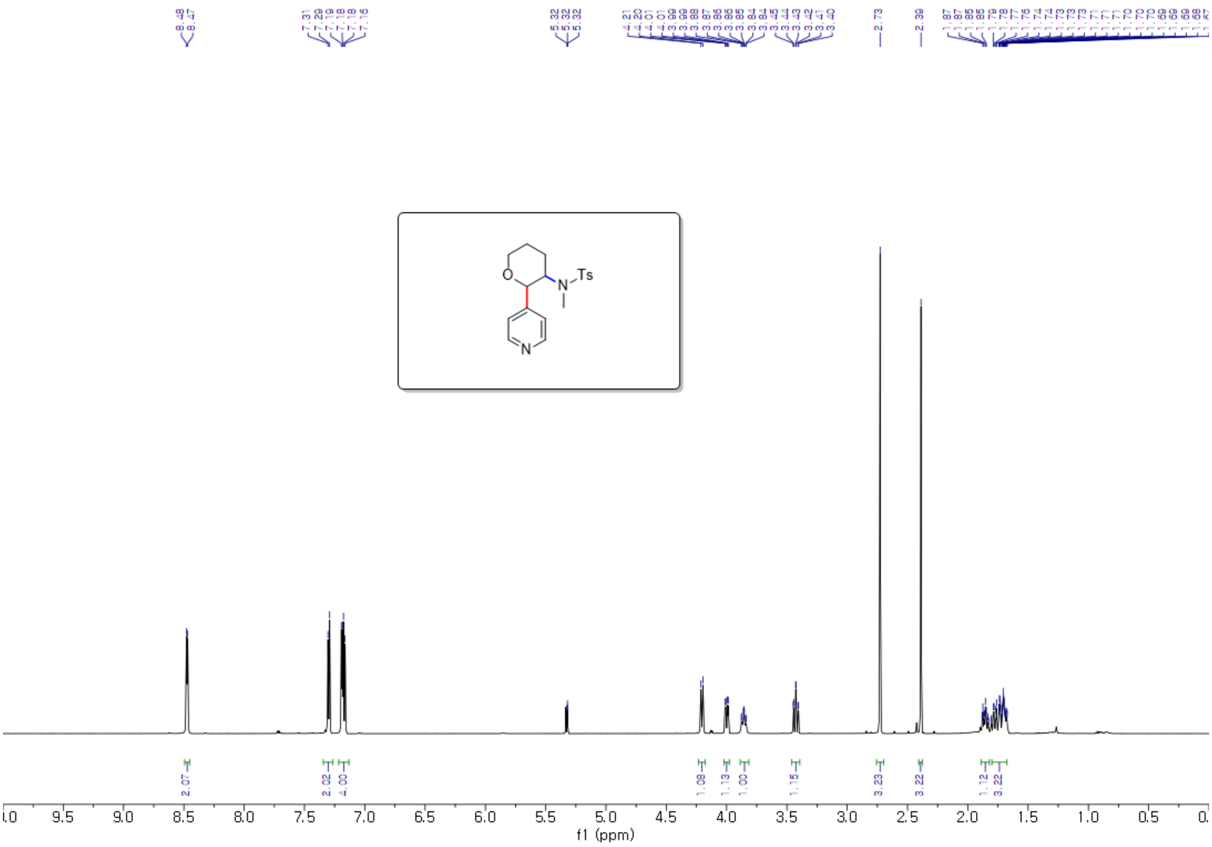

600 MHz, ^{1}H NMR in CD_2Cl_2

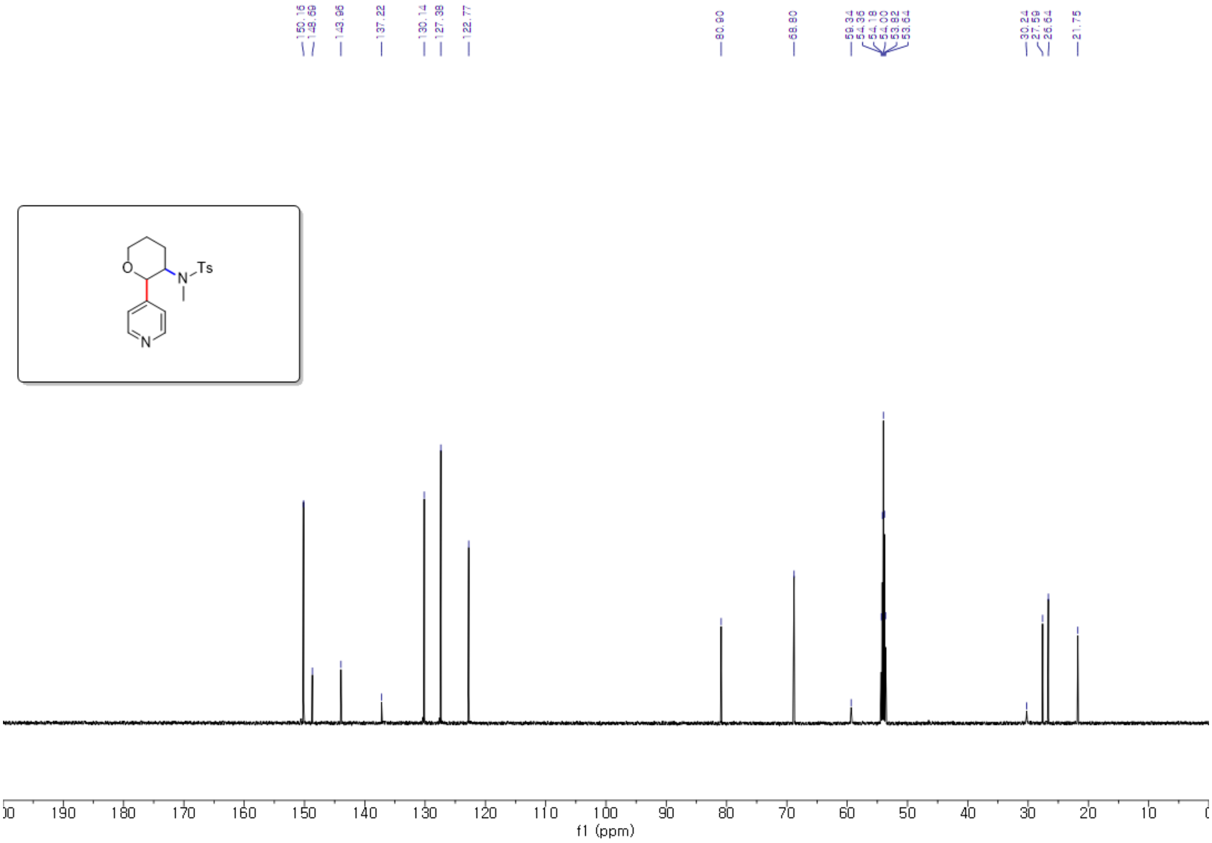

150 MHz, ^{13}C NMR in CD_2Cl_2

Supplementary Figure 35. 1H and 13C NMR of **3p**

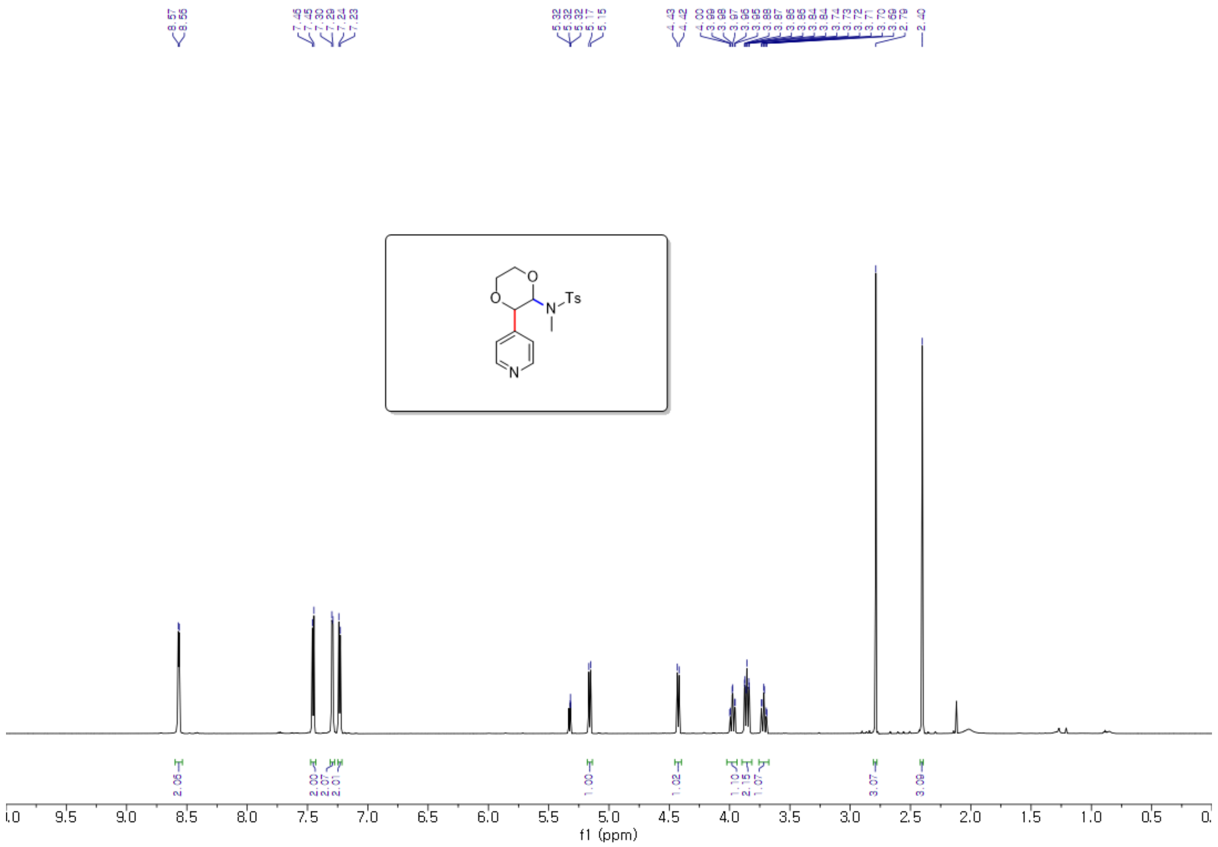

600 MHz, ^{1}H NMR in CD_2Cl_2

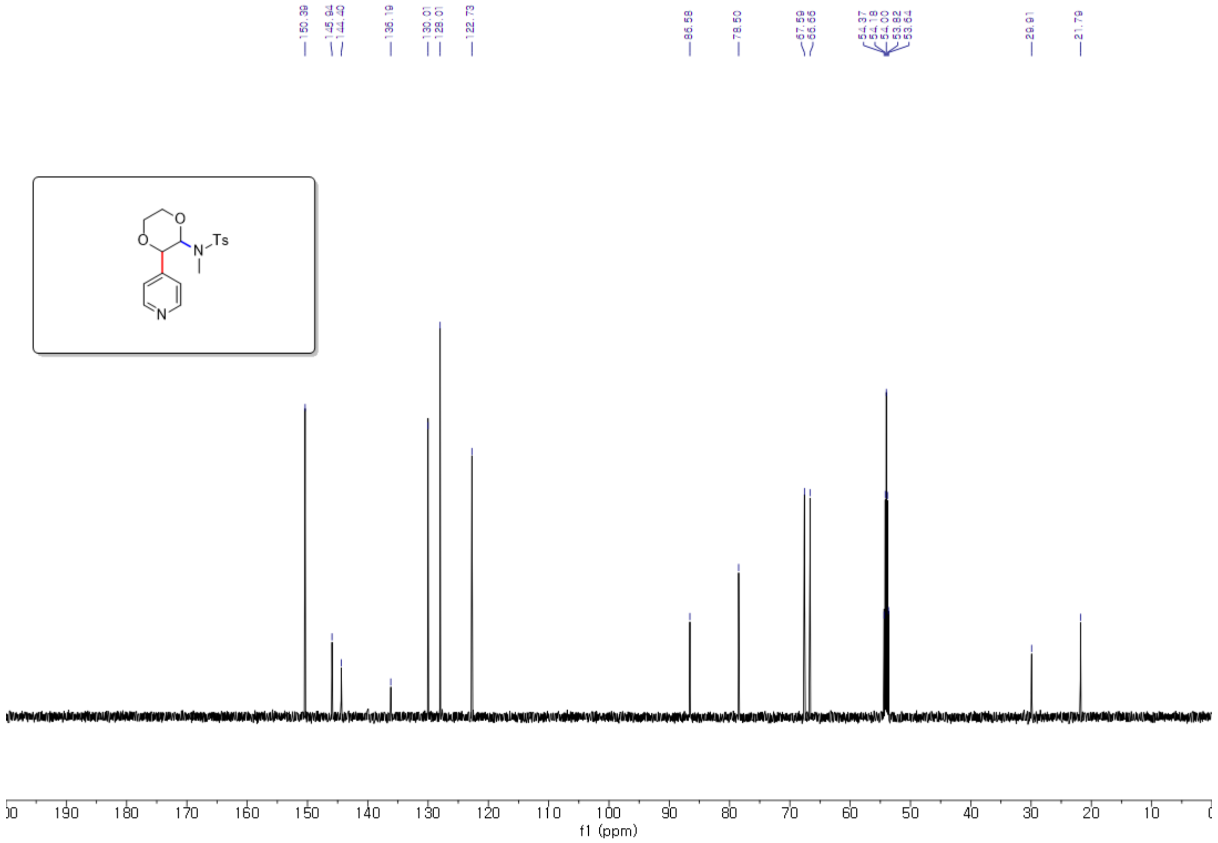

150 MHz, ^{13}C NMR in CD_2Cl_2

Supplementary Figure 36. 1H and 13C NMR of **3q**

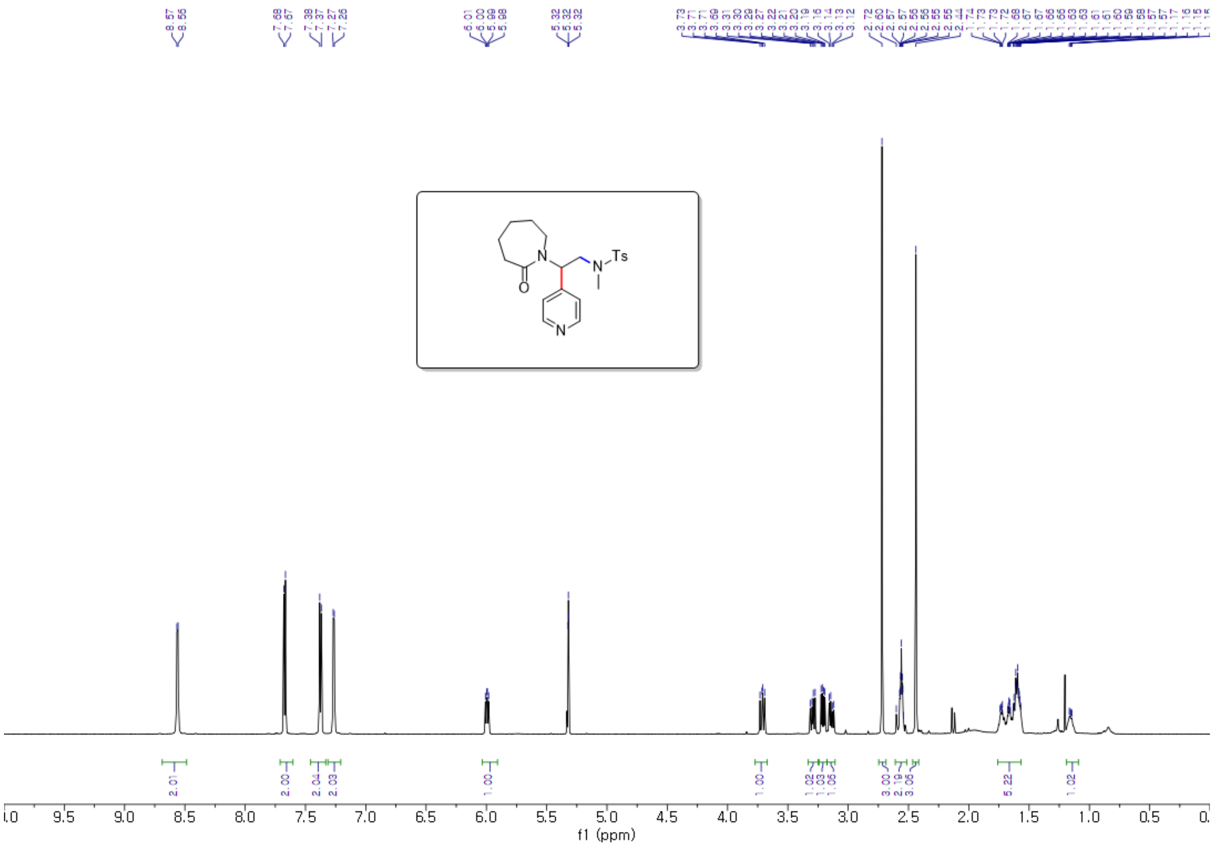

600 MHz, ^{1}H NMR in CD_2Cl_2

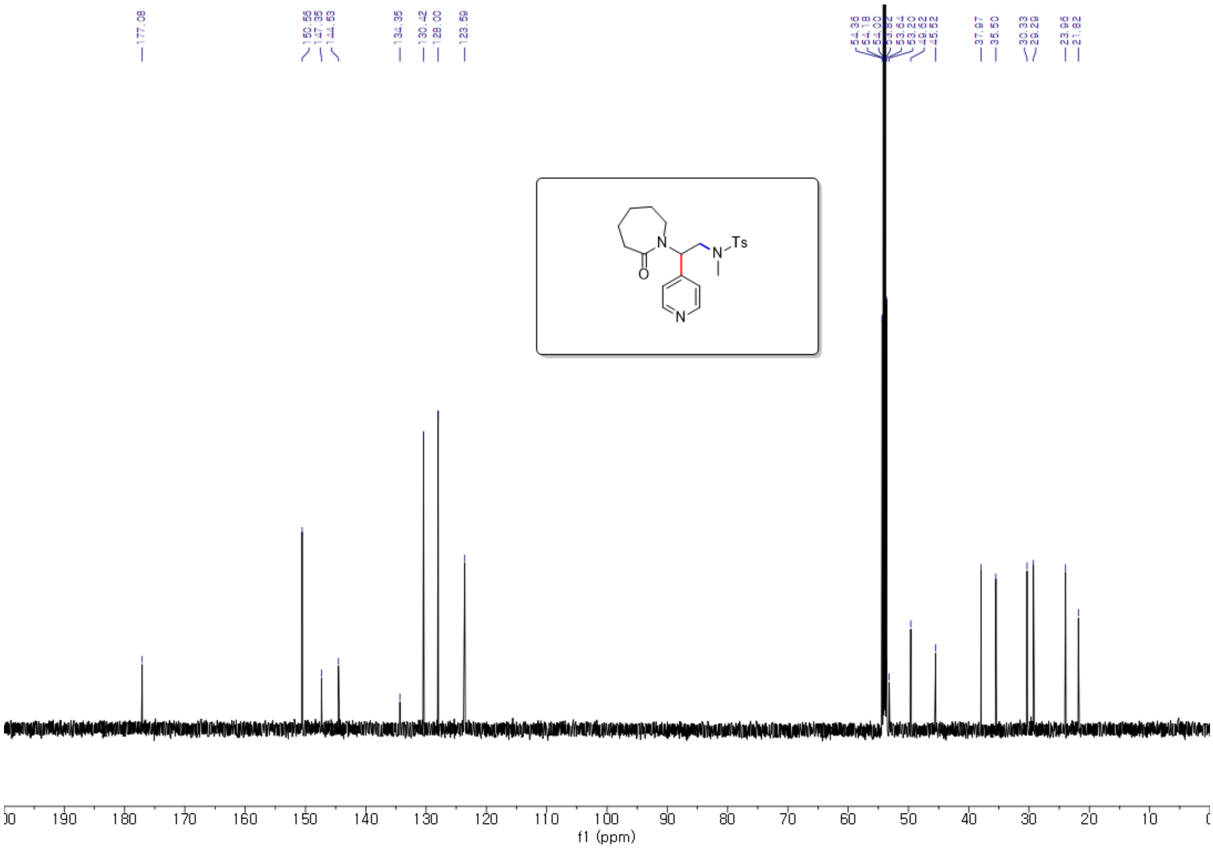

150 MHz, ^{13}C NMR in CD_2Cl_2

Supplementary Figure 37. 1H and 13C NMR of **3r**

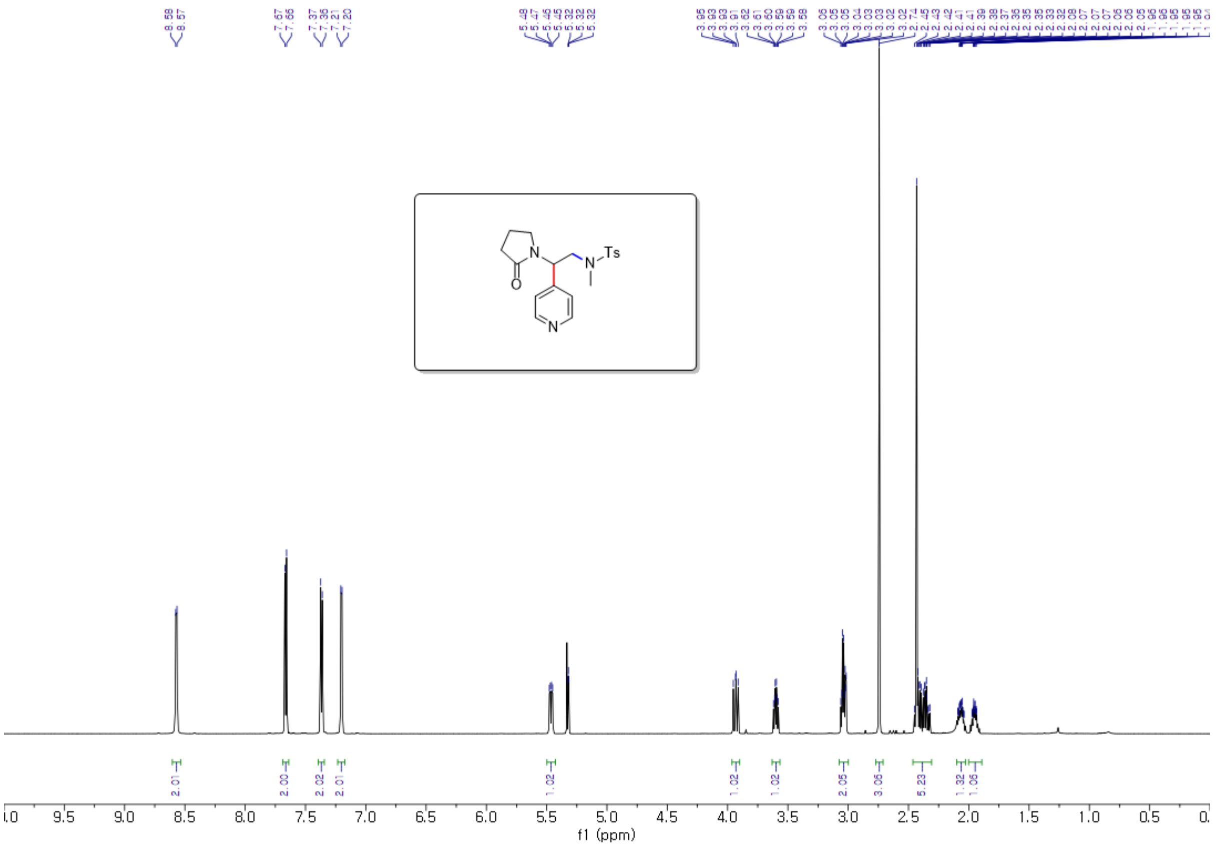

600 MHz, ¹H NMR in CD_2Cl_2

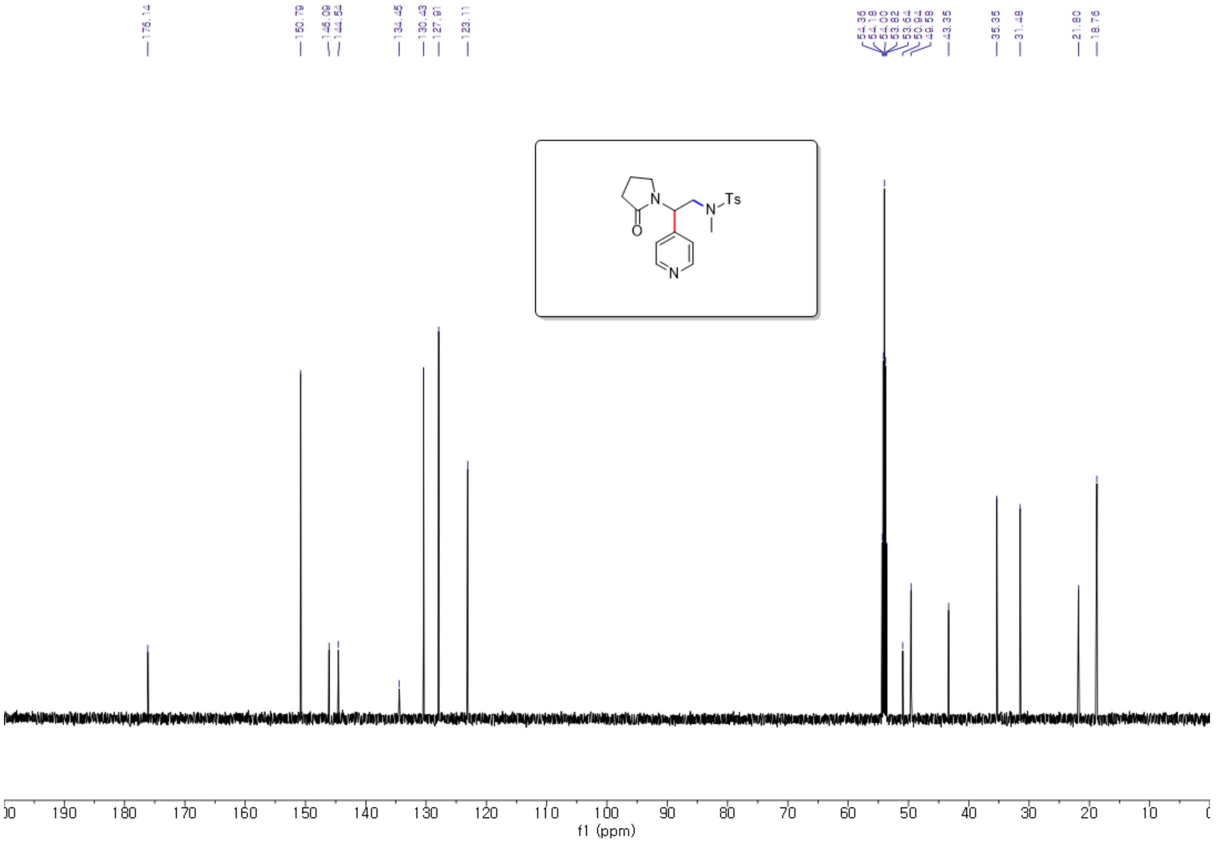

150 MHz, ¹³C NMR in CD_2Cl_2

Supplementary Figure 38. 1H and 13C NMR of **3s**

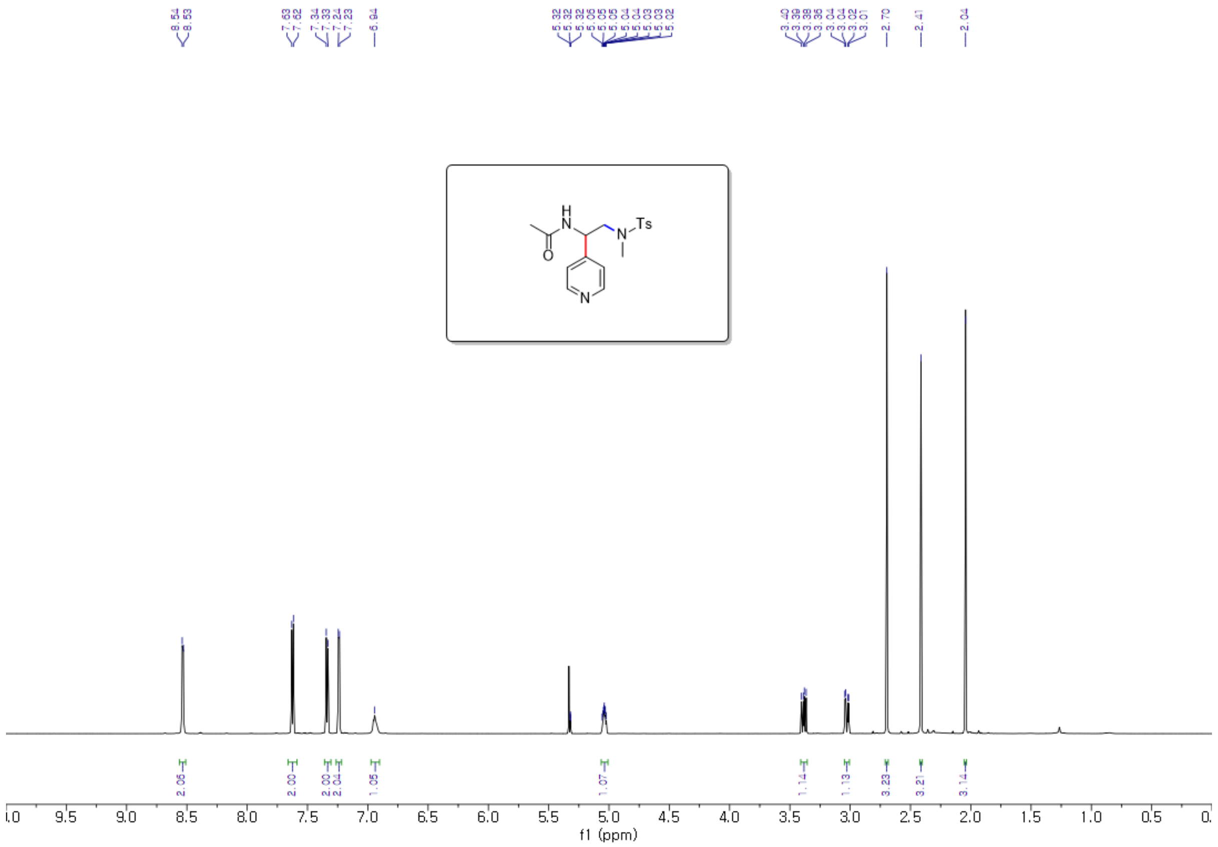

600 MHz, ^{1}H NMR in CD_2Cl_2

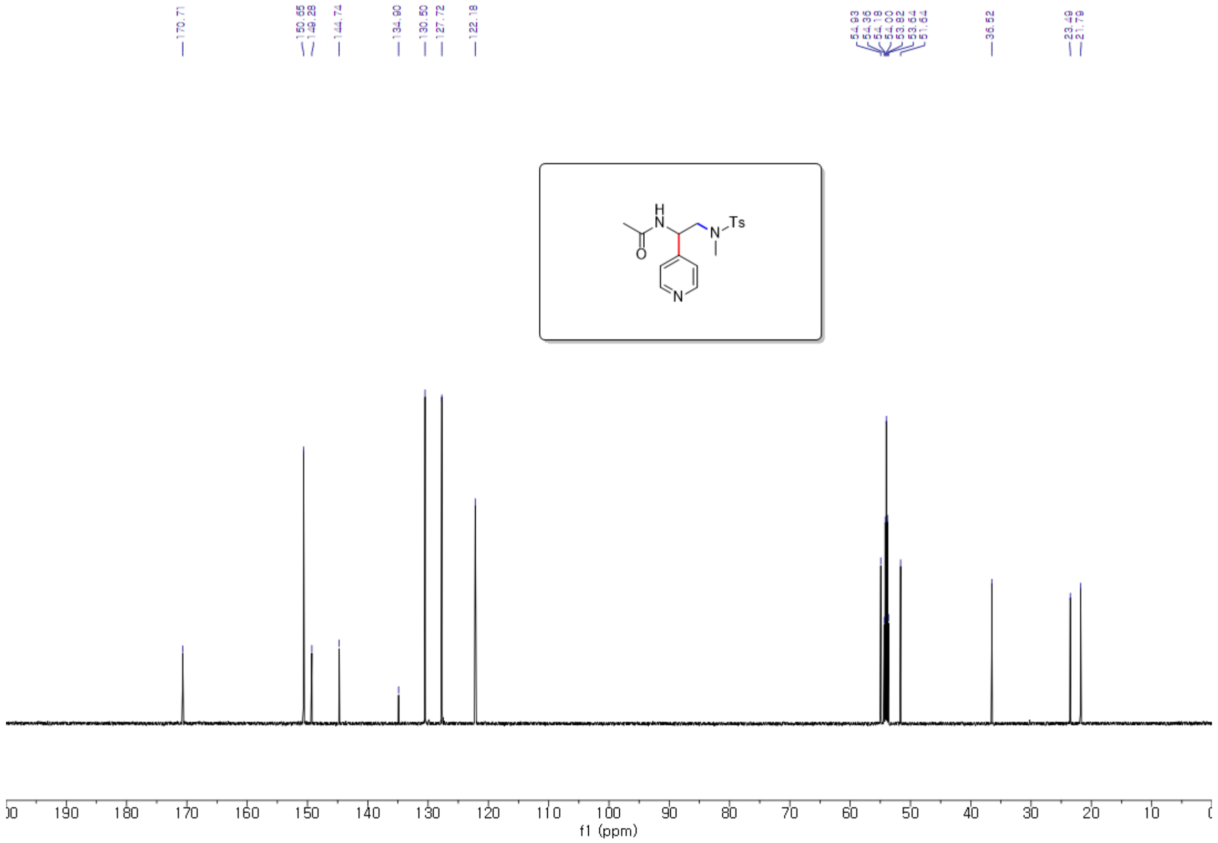

150 MHz, ^{13}C NMR in CD_2Cl_2

Supplementary Figure 39. 1H and 13C NMR of **3t**

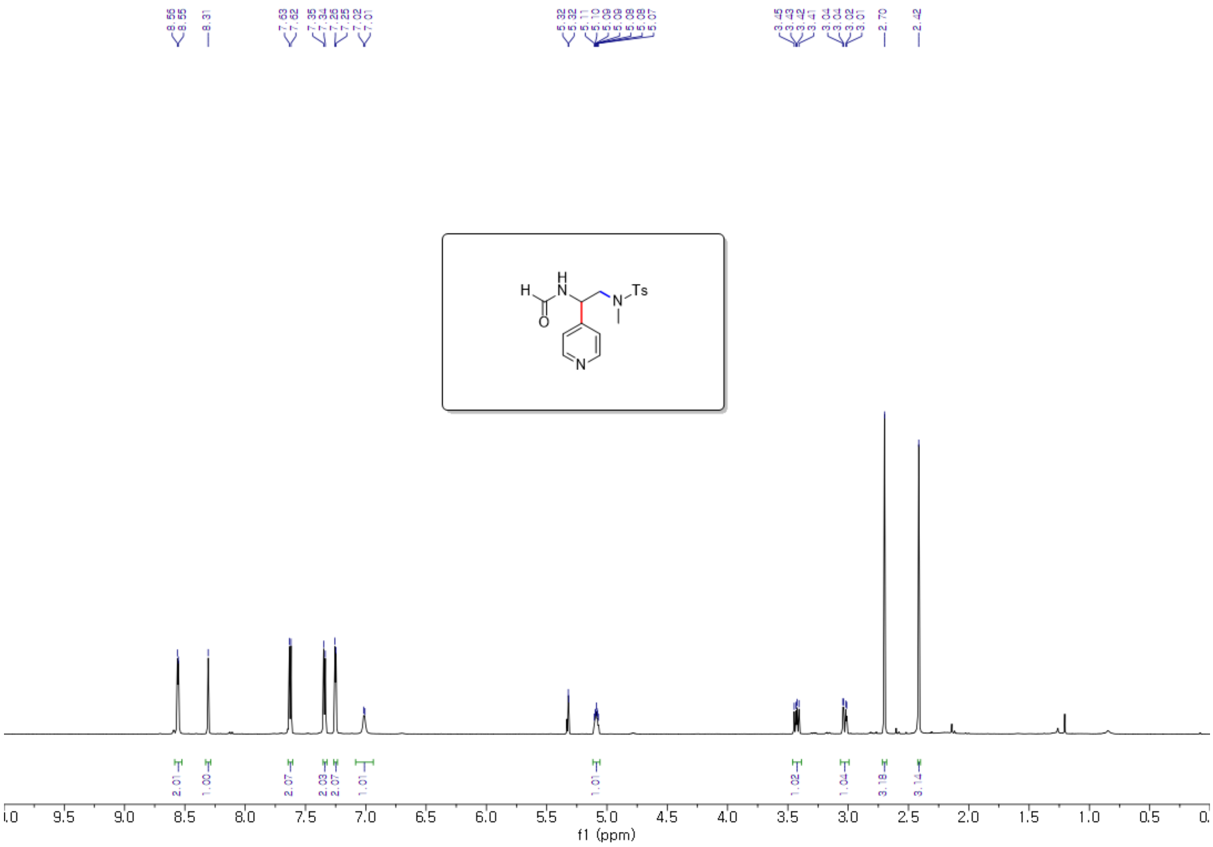

600 MHz, ^{1}H NMR in CD_2Cl_2

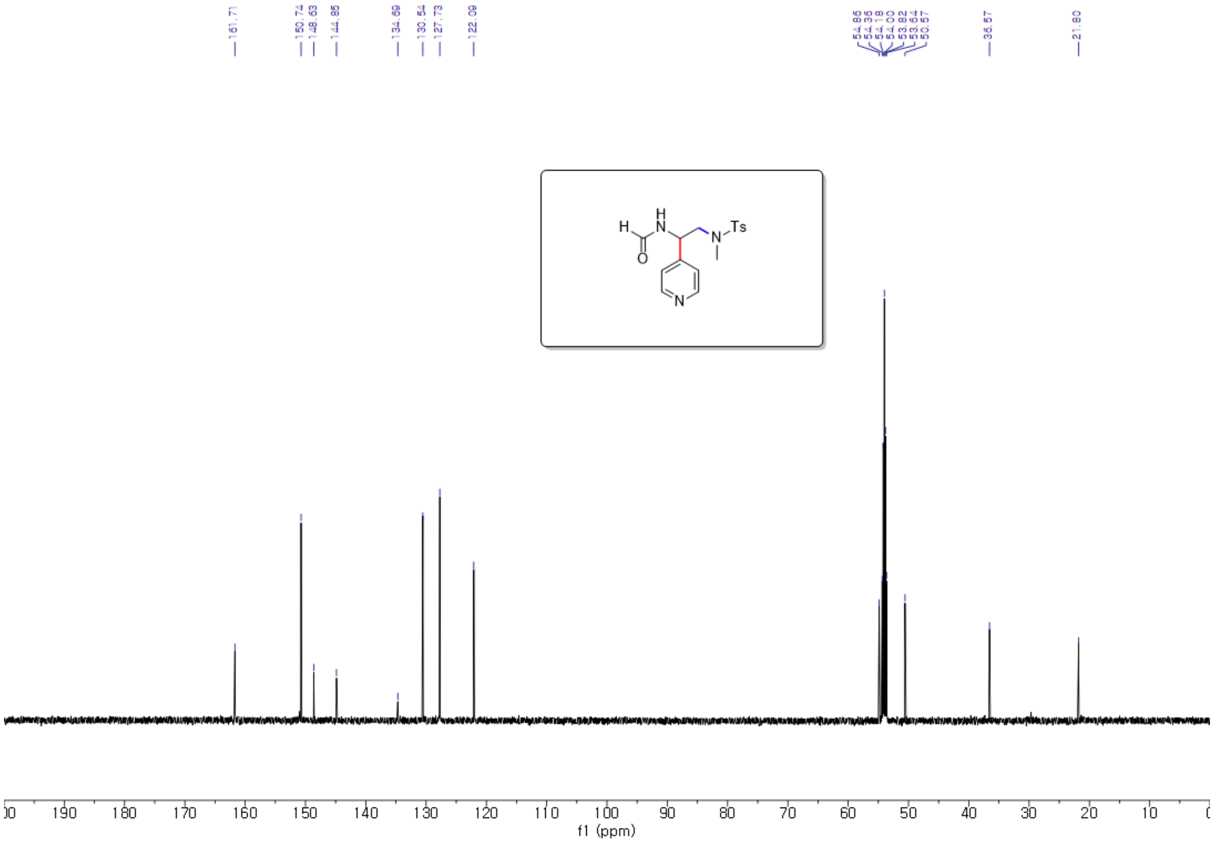

150 MHz, ^{13}C NMR in CD_2Cl_2

Supplementary Figure 40. 1H and 13C NMR of **3u**

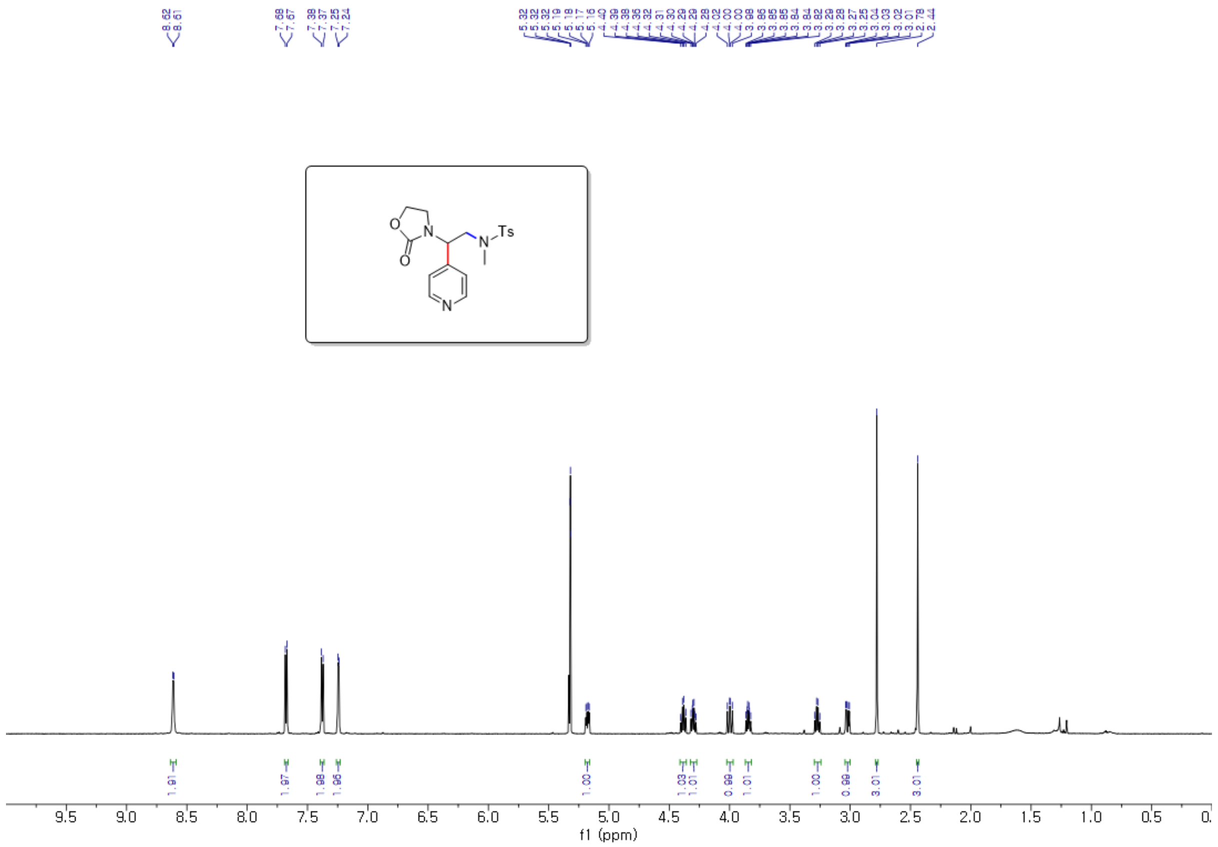

600 MHz, ^{1}H NMR in CD_2Cl_2

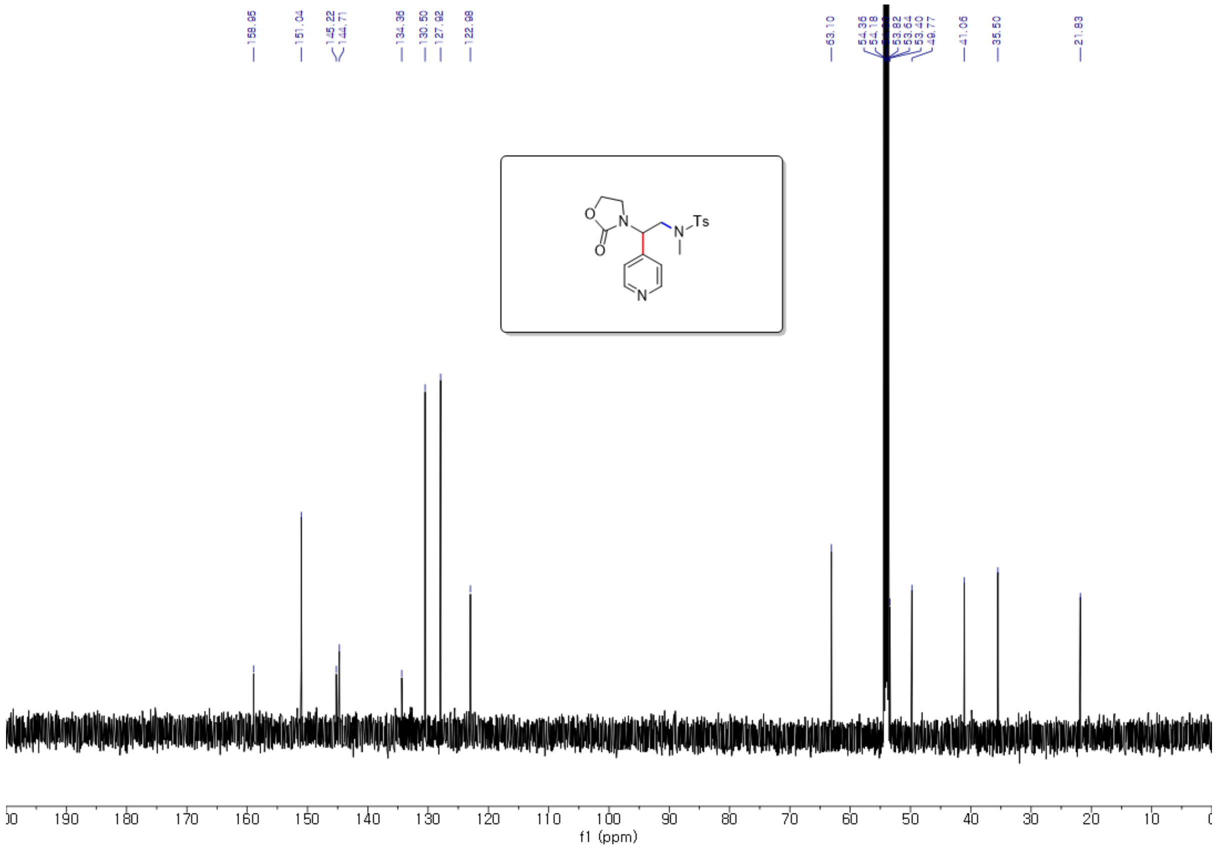

150 MHz, ^{13}C NMR in CD_2Cl_2

Supplementary Figure 41. 1H and 13C NMR of **3v**

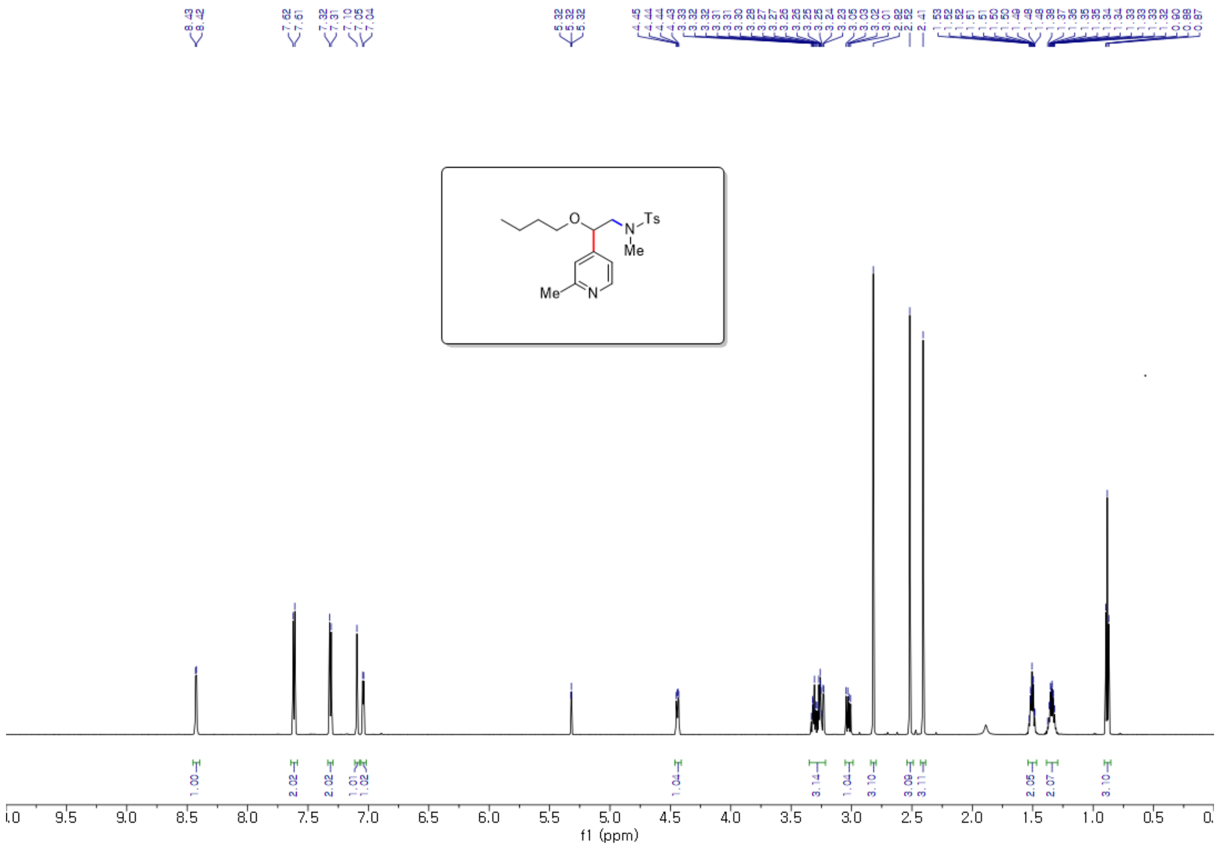

600 MHz, ^{1}H NMR in CD_2Cl_2

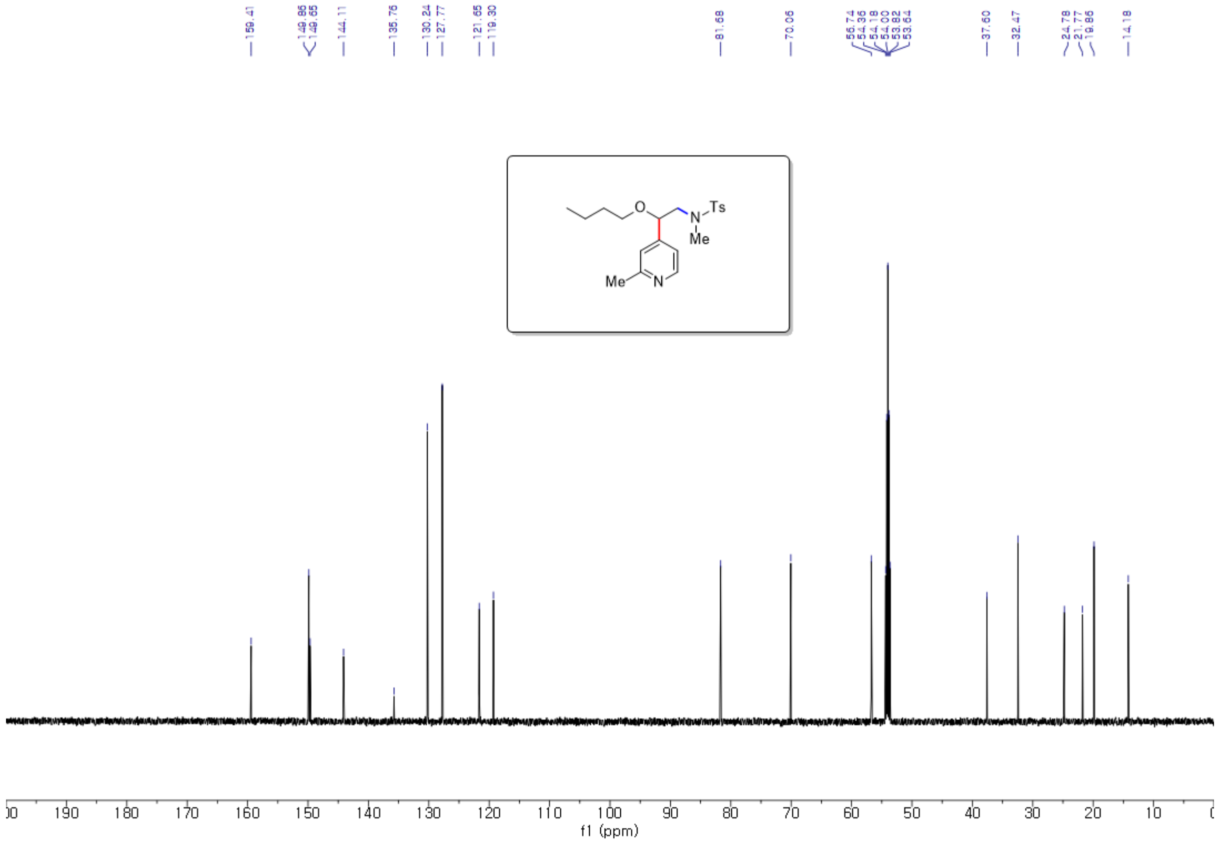

150 MHz, ^{13}C NMR in CD_2Cl_2

Supplementary Figure 42. 1H and 13C NMR of **4a**

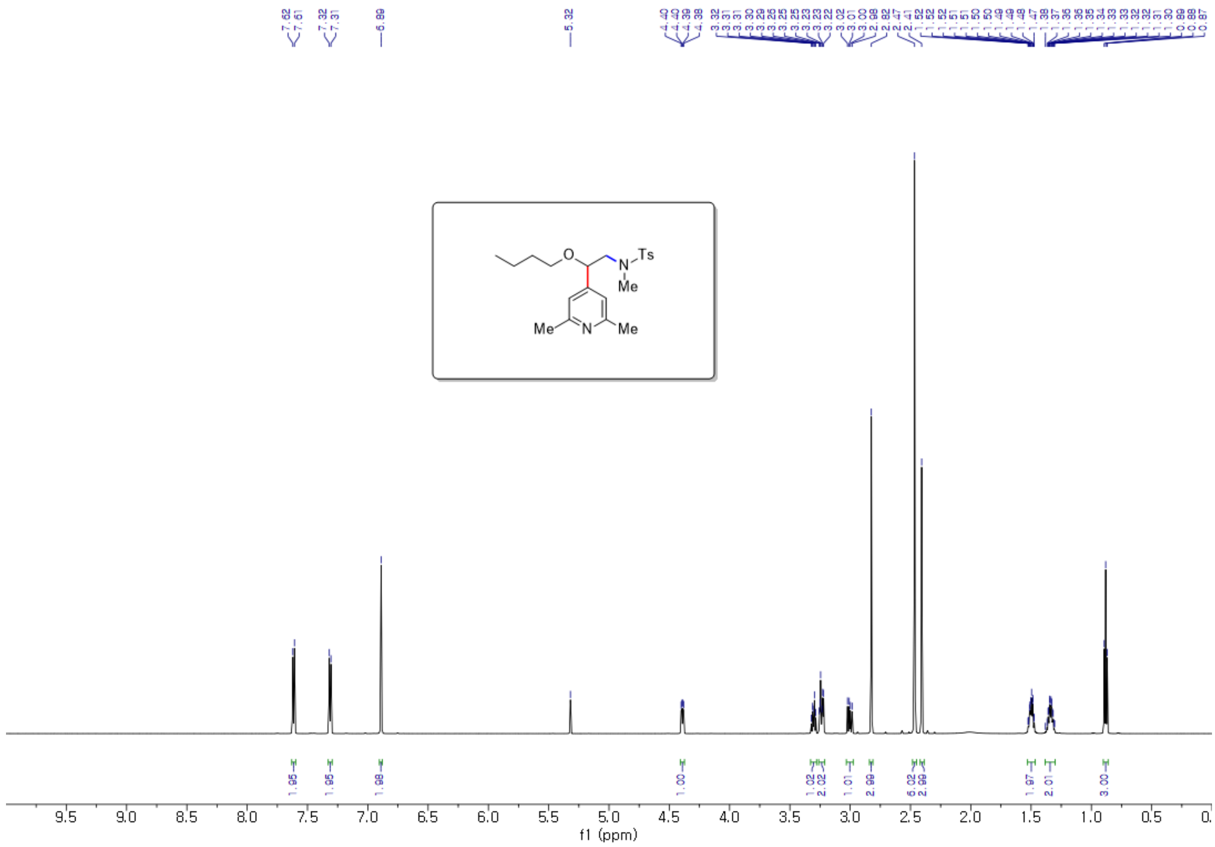

600 MHz, ^{1}H NMR in CD_2Cl_2

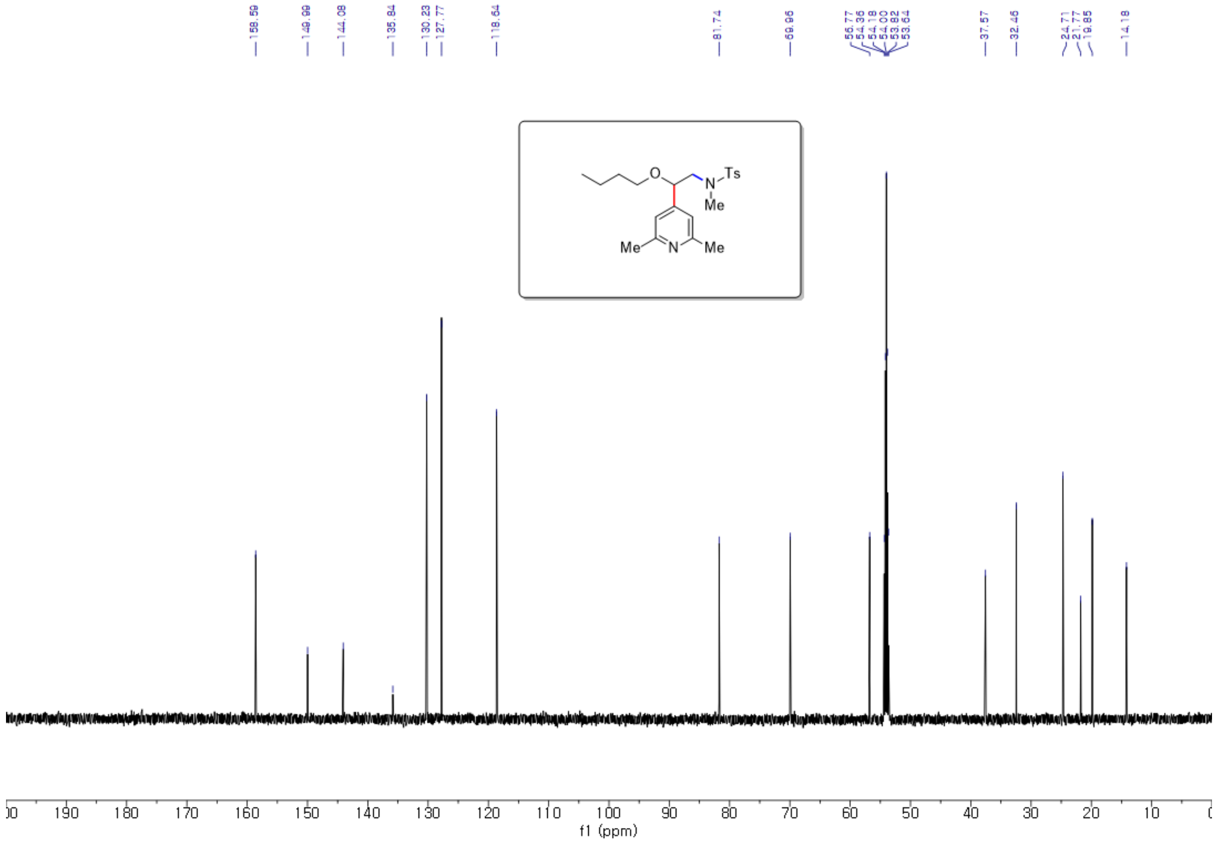

150 MHz, ^{13}C NMR in CD_2Cl_2

Supplementary Figure 43. 1H and 13C NMR of **4b**

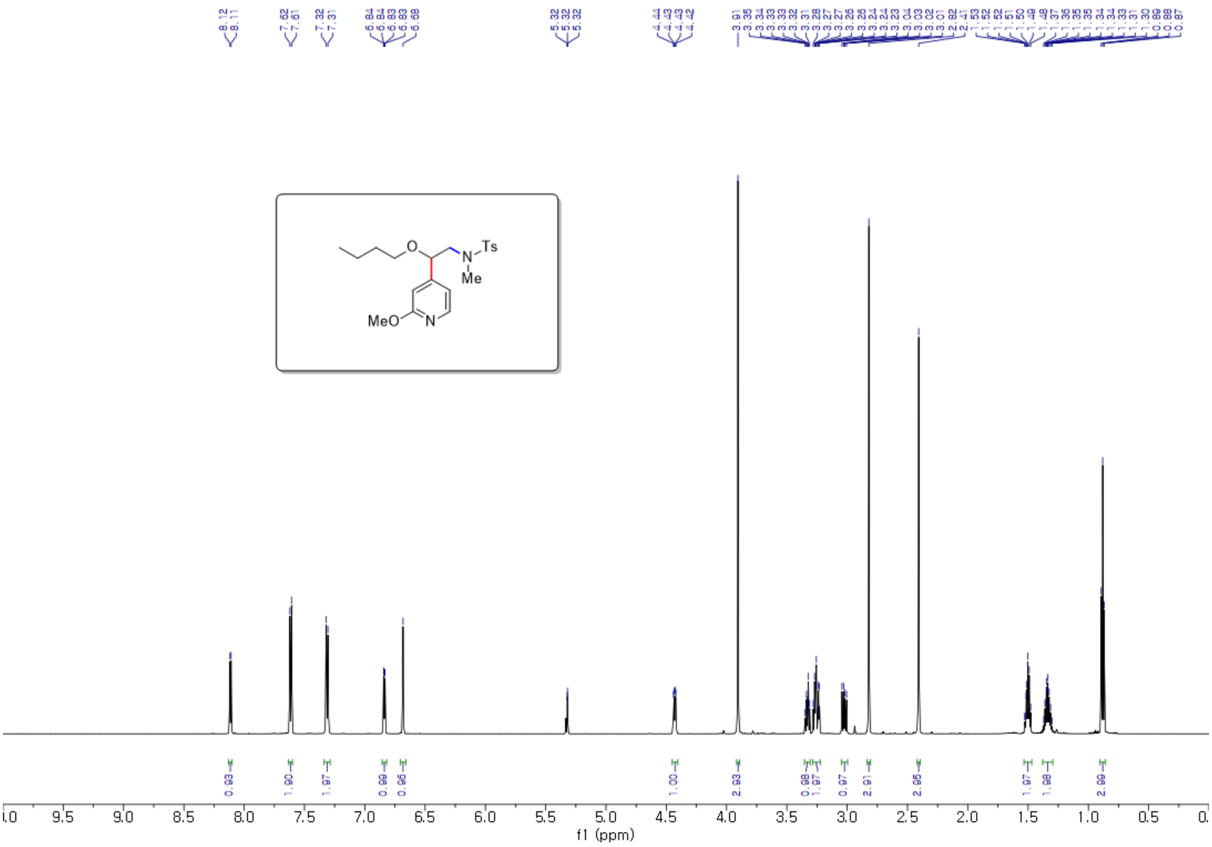

600 MHz, ^{1}H NMR in CD_2Cl_2

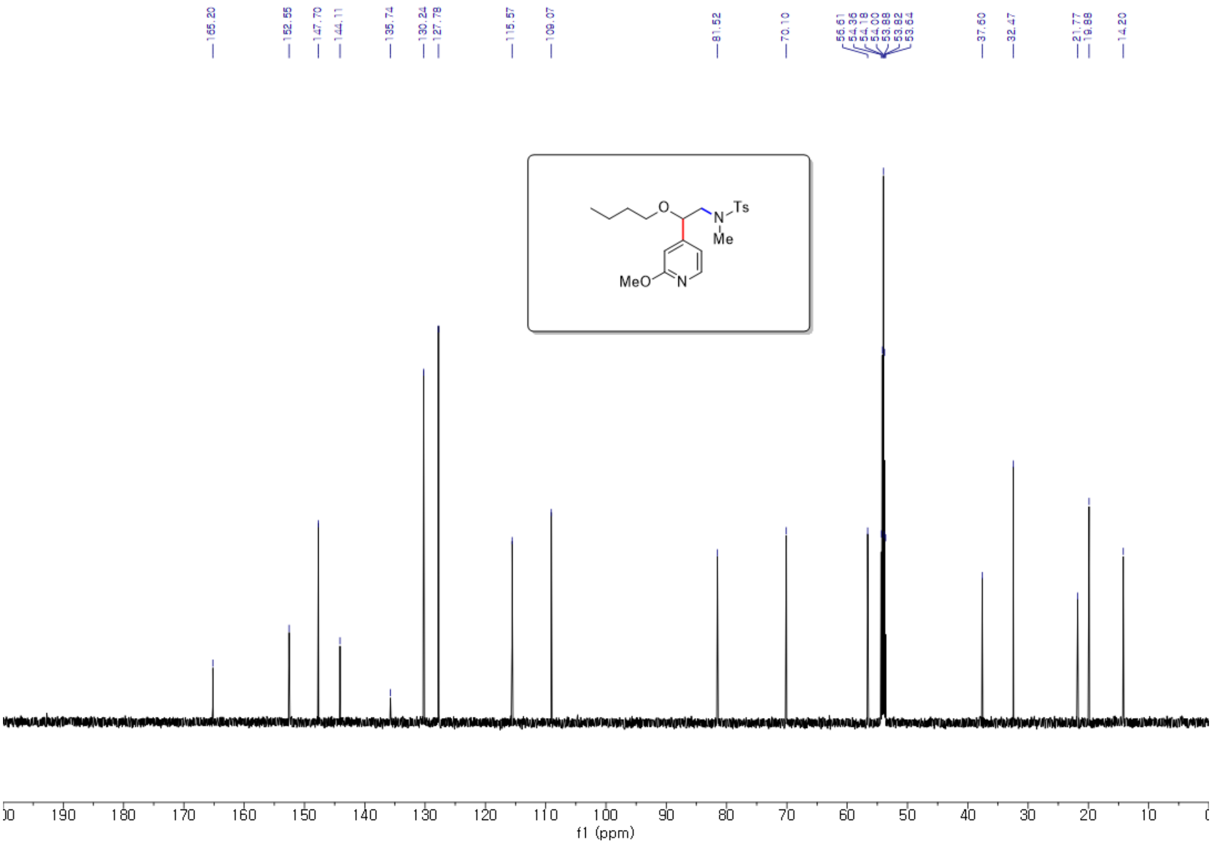

150 MHz, ^{13}C NMR in CD_2Cl_2

Supplementary Figure 44. 1H and 13C NMR of **4c**

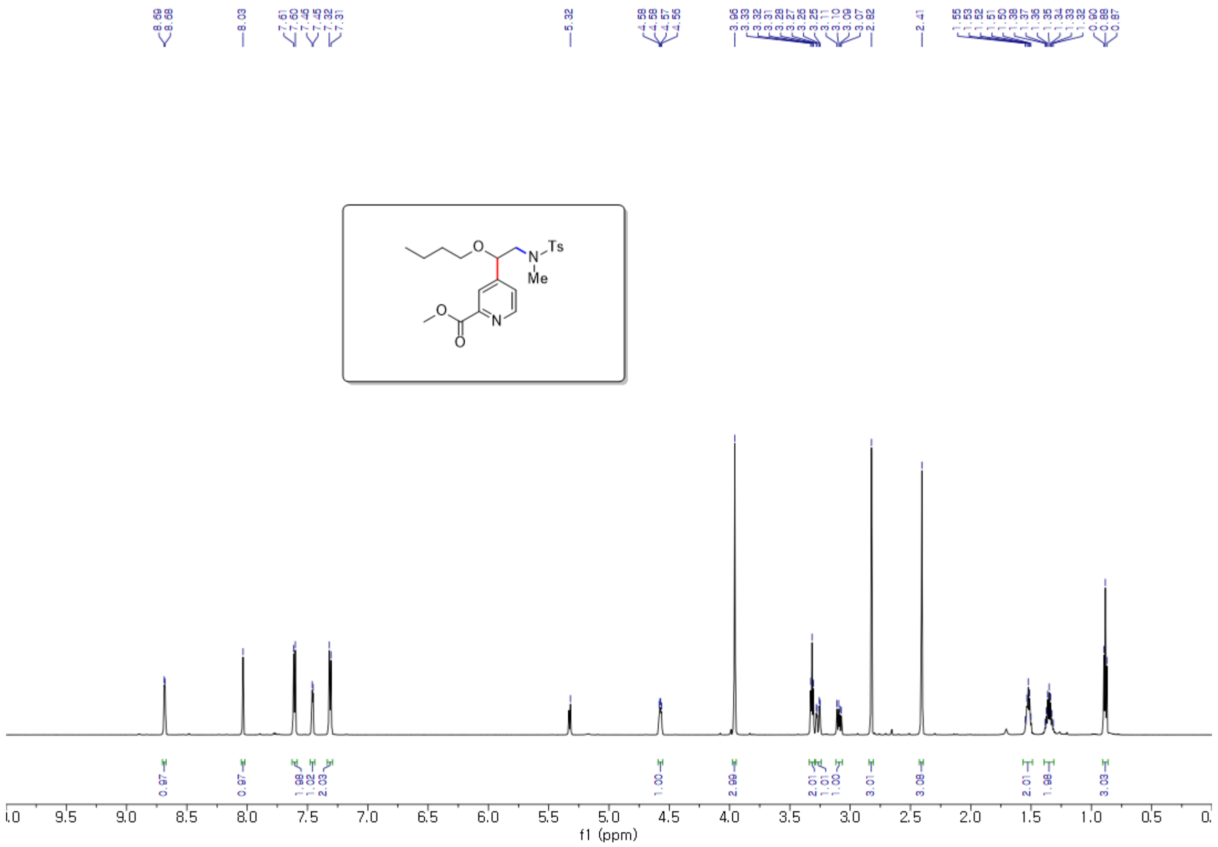

600 MHz, ^{1}H NMR in CD_2Cl_2

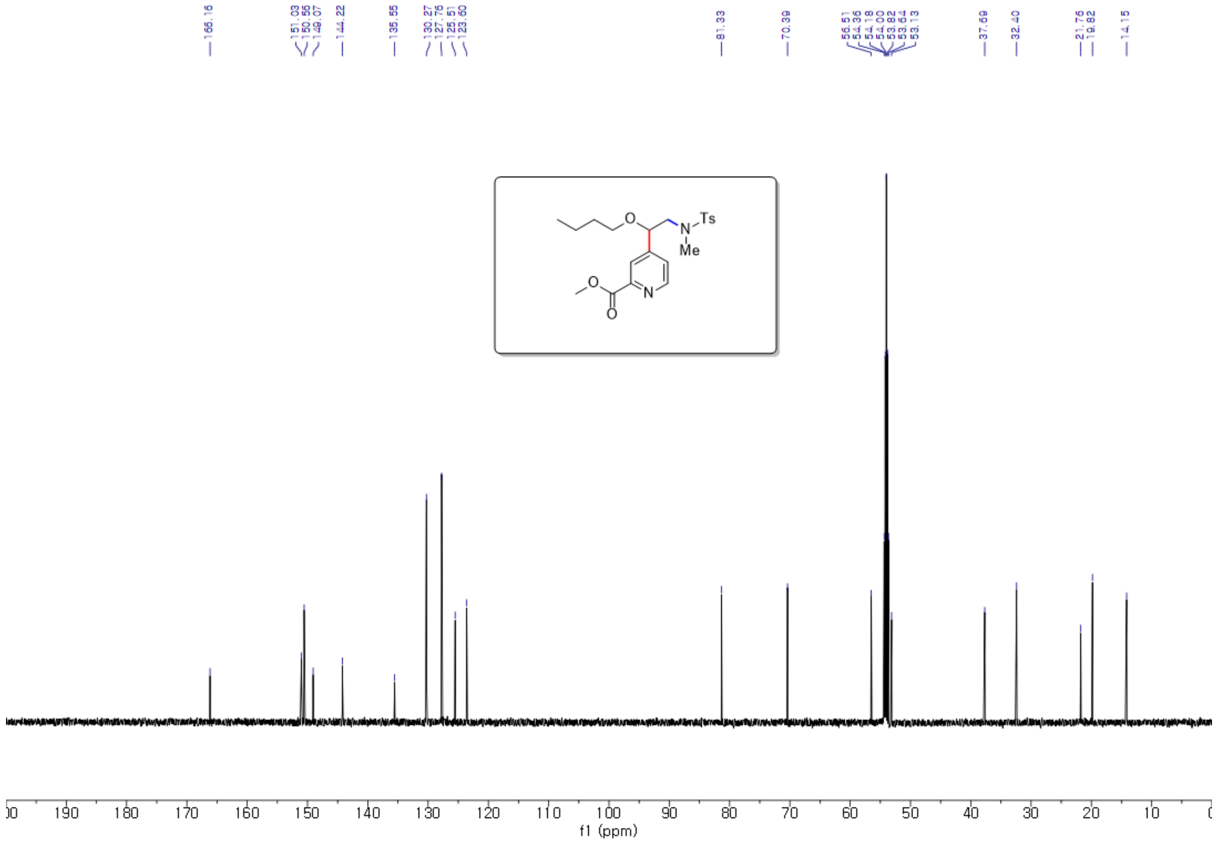

150 MHz, ^{13}C NMR in CD_2Cl_2

Supplementary Figure 45. 1H and 13C NMR of **4d**

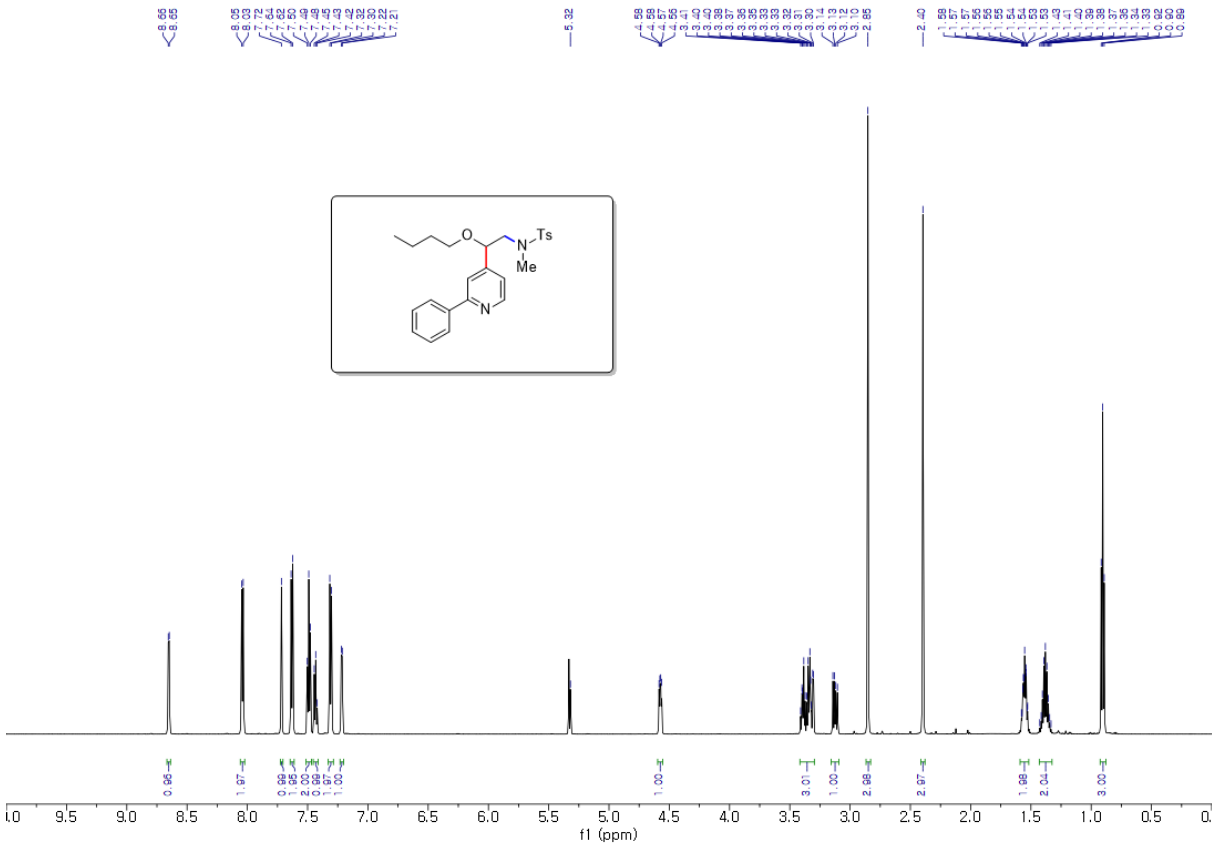

600 MHz, ^{1}H NMR in CD_2Cl_2

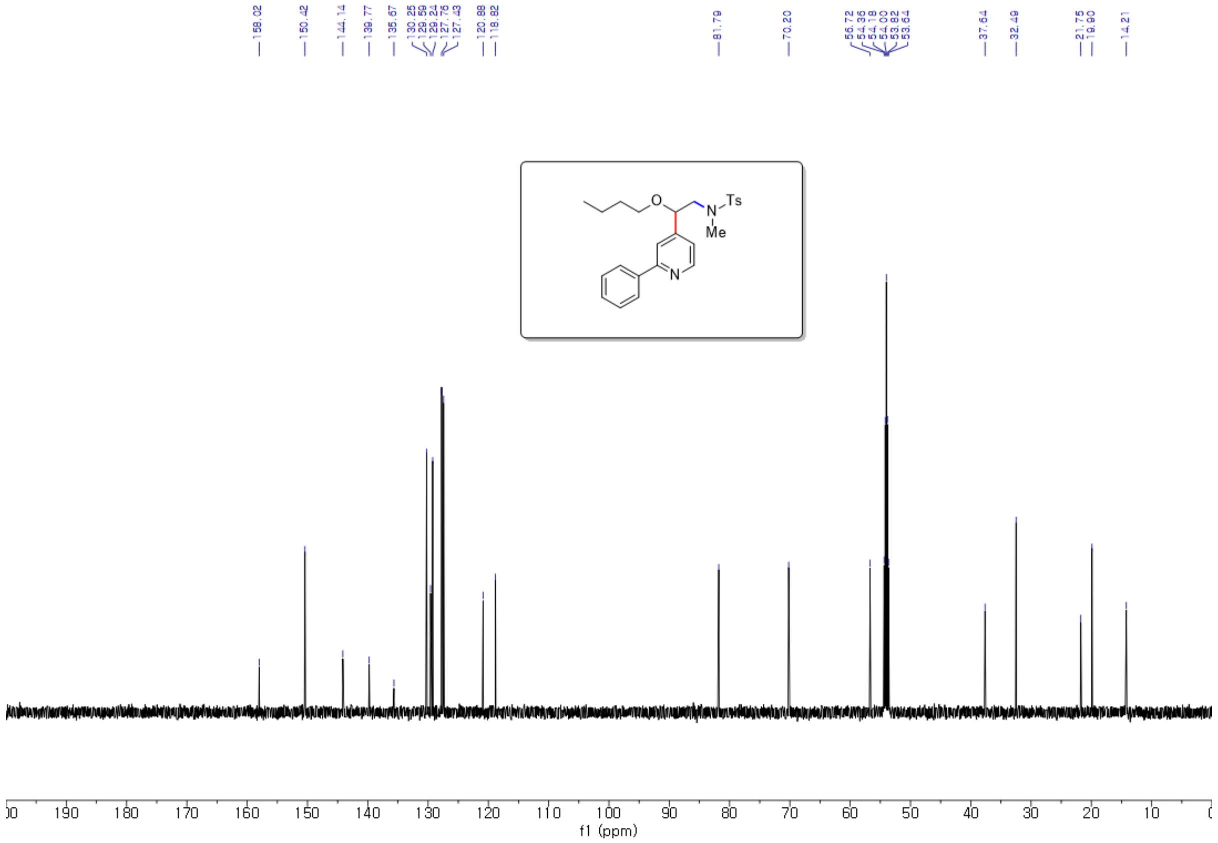

150 MHz, ^{13}C NMR in CD_2Cl_2

Supplementary Figure 46. 1H and 13C NMR of **4e**

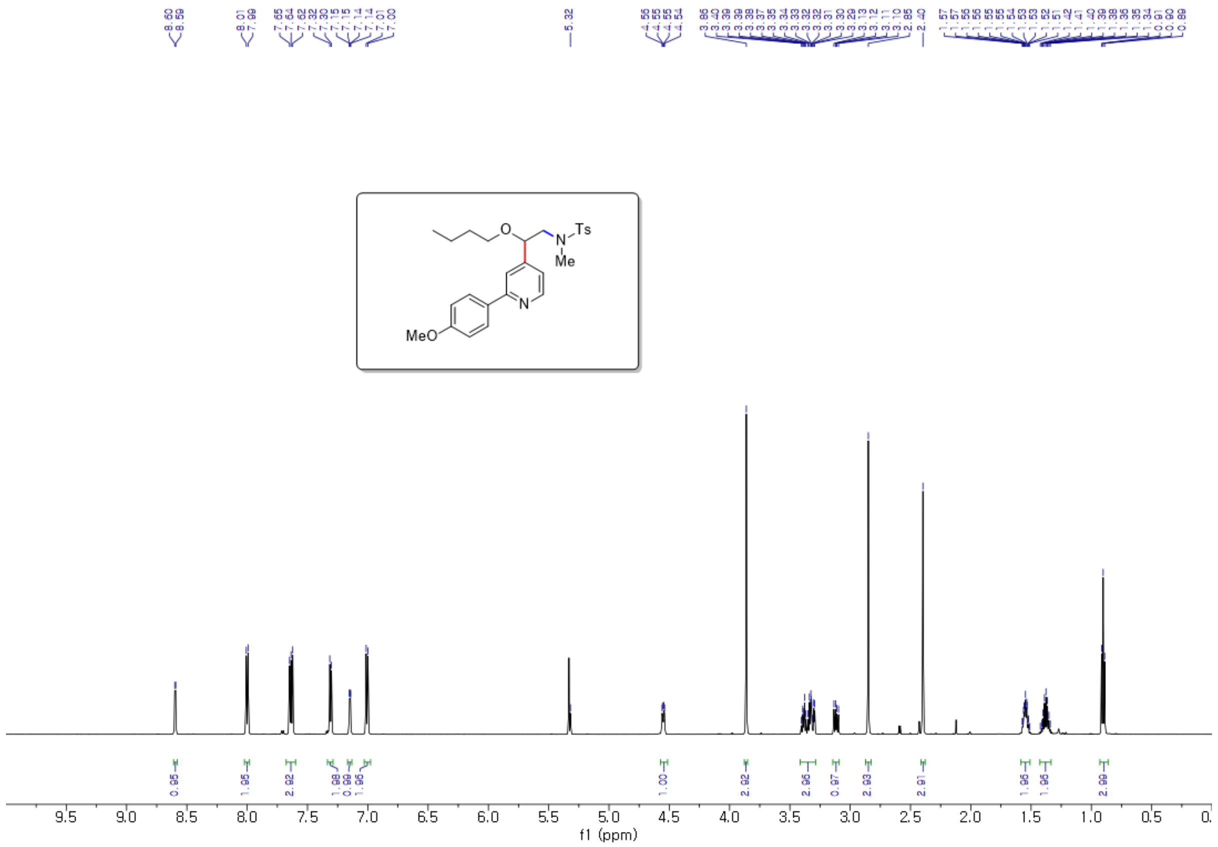

600 MHz, ^{1}H NMR in CD_2Cl_2

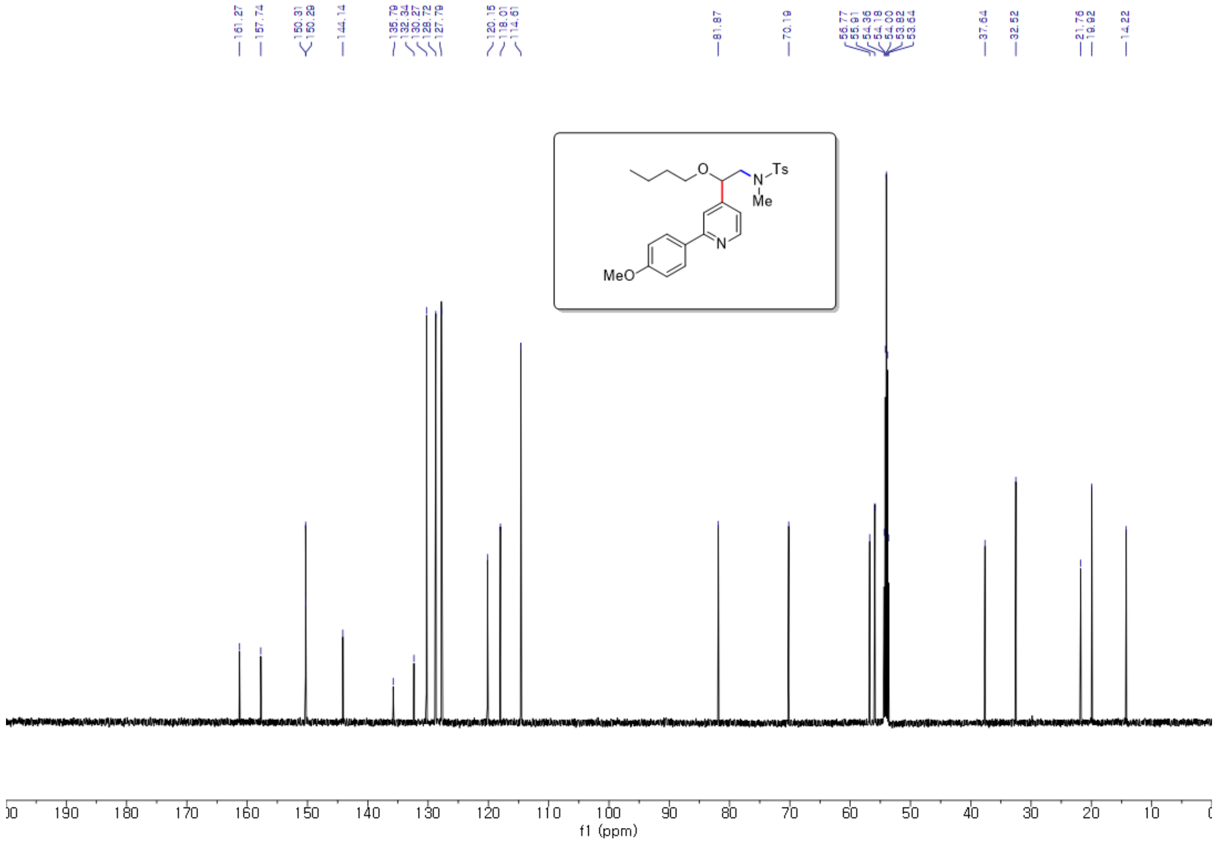

150 MHz, ^{13}C NMR in CD_2Cl_2

Supplementary Figure 47. 1H and 13C NMR of **4f**

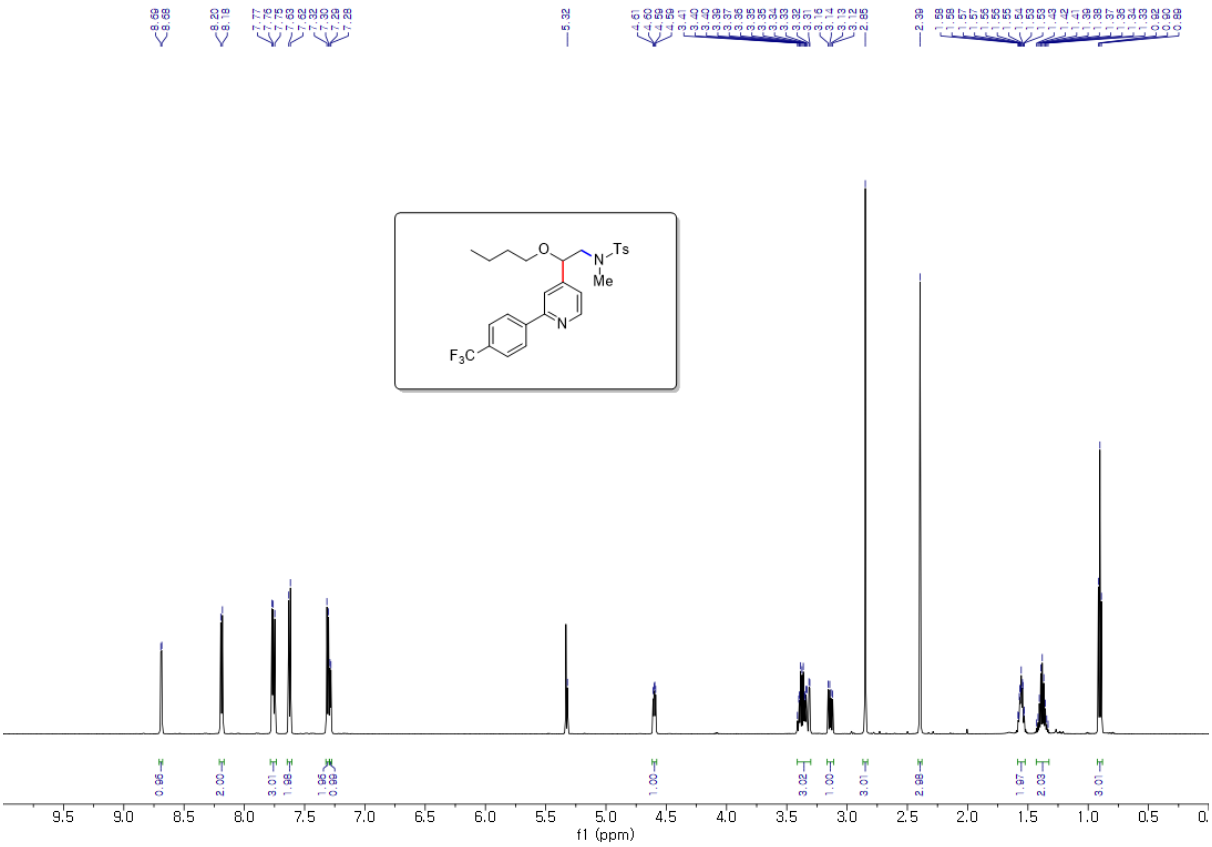

600 MHz, ^{1}H NMR in CD_2Cl_2

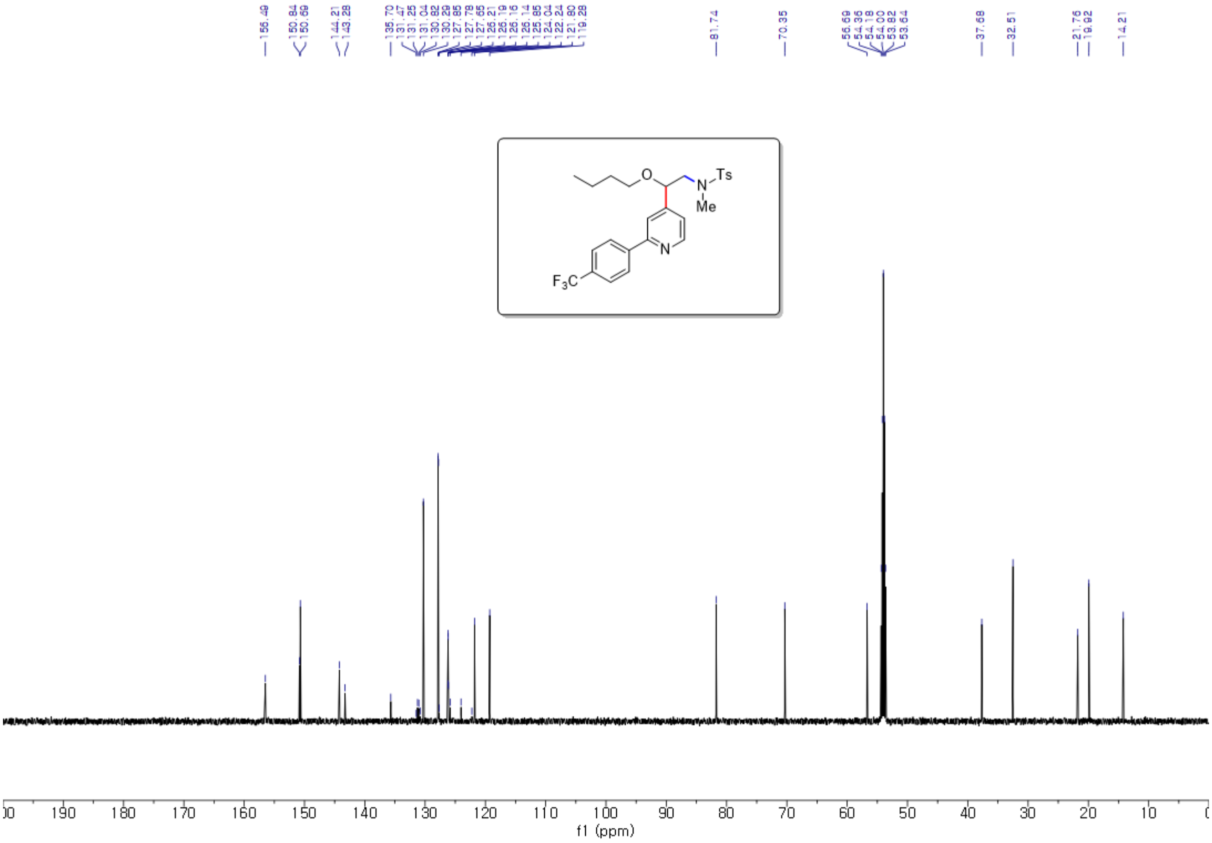

150 MHz, ^{13}C NMR in CD_2Cl_2

Supplementary Figure 48. 1H and 13C NMR of **4g**

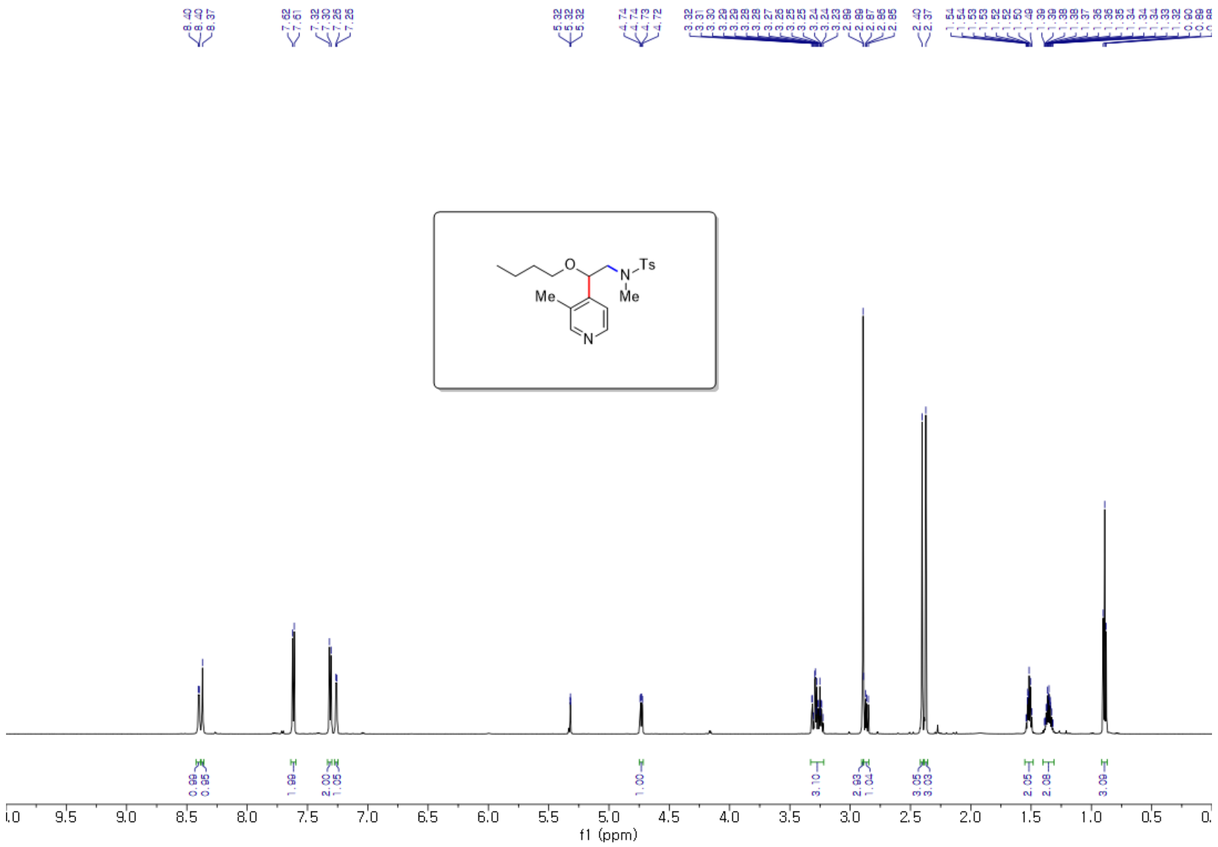

600 MHz, ^{1}H NMR in CD_2Cl_2

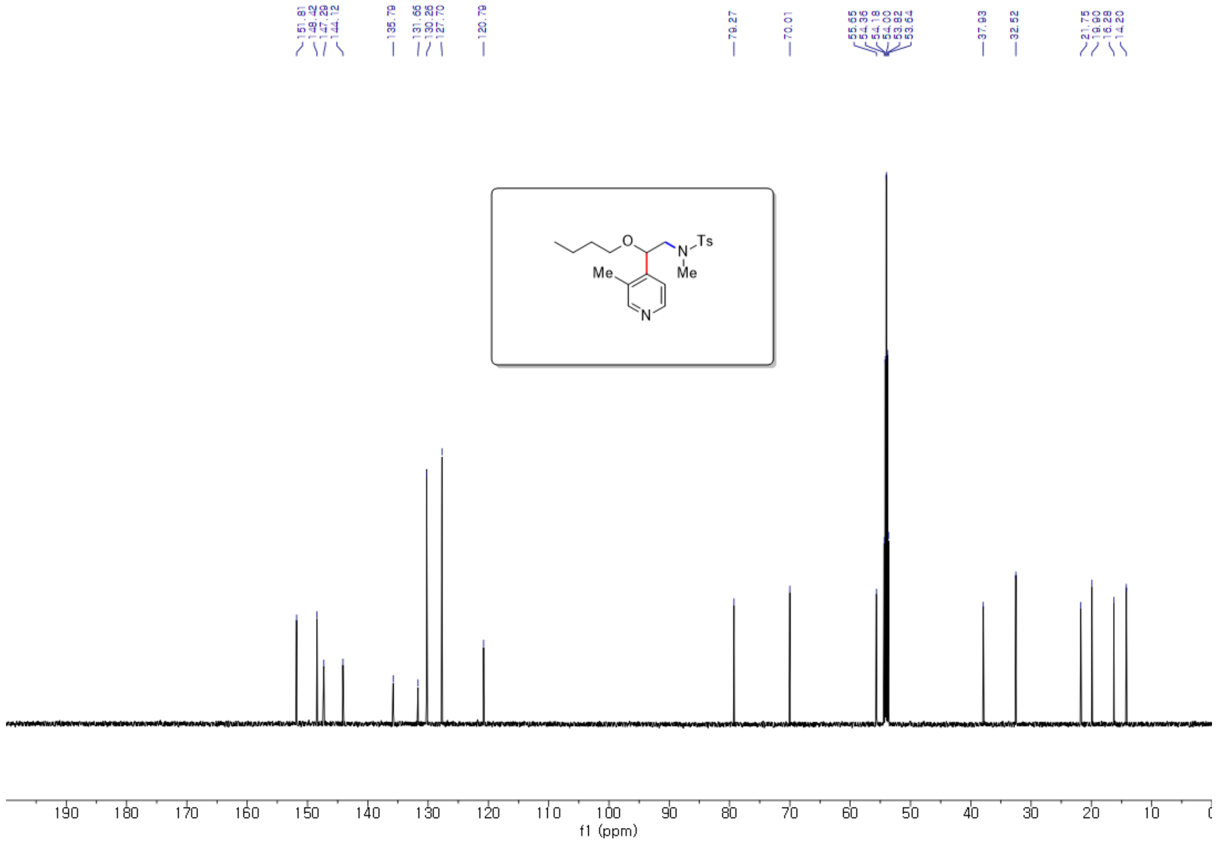

150 MHz, ^{13}C NMR in CD_2Cl_2

Supplementary Figure 49. 1H and 13C NMR of **4h**

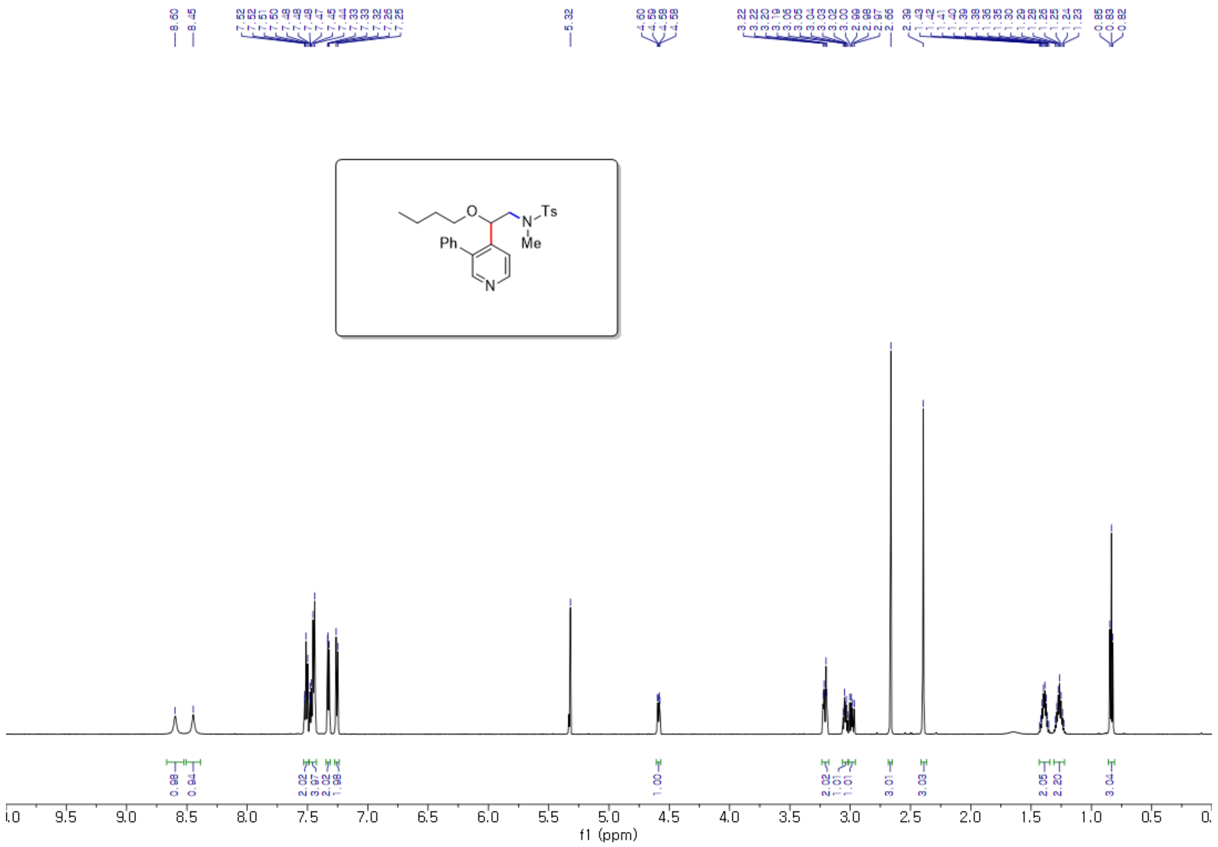

600 MHz, ¹H NMR in CD_2Cl_2

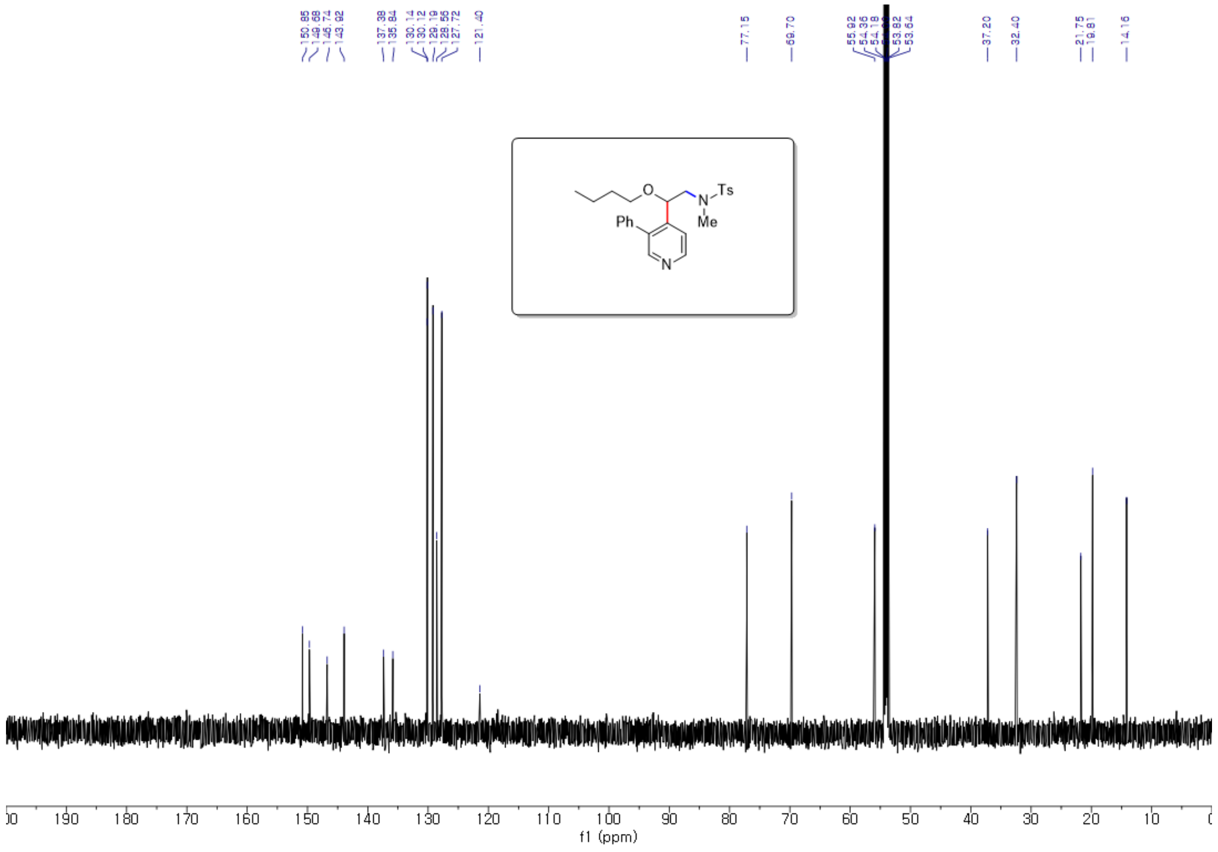

150 MHz, ¹³C NMR in CD_2Cl_2

Supplementary Figure 50. 1H and 13C NMR of **4i**

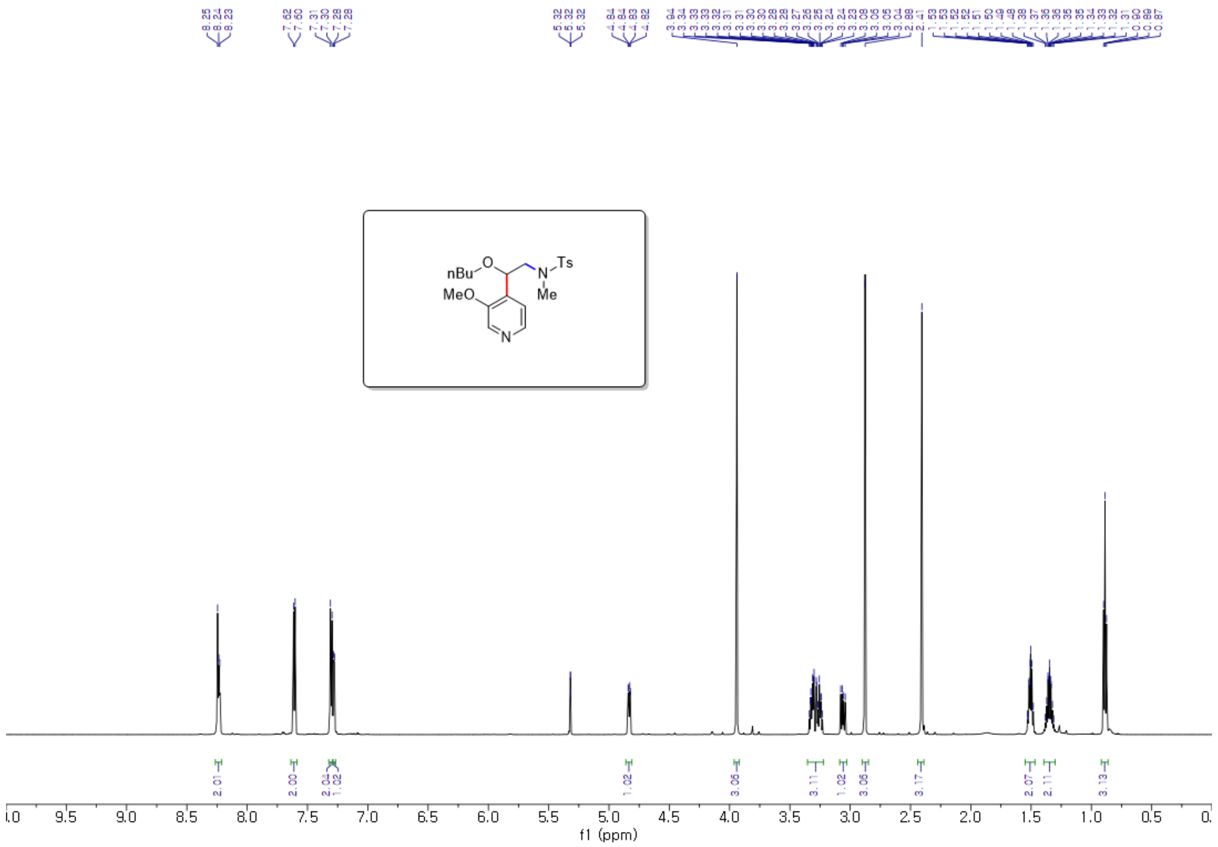

600 MHz, ^{1}H NMR in CD_2Cl_2

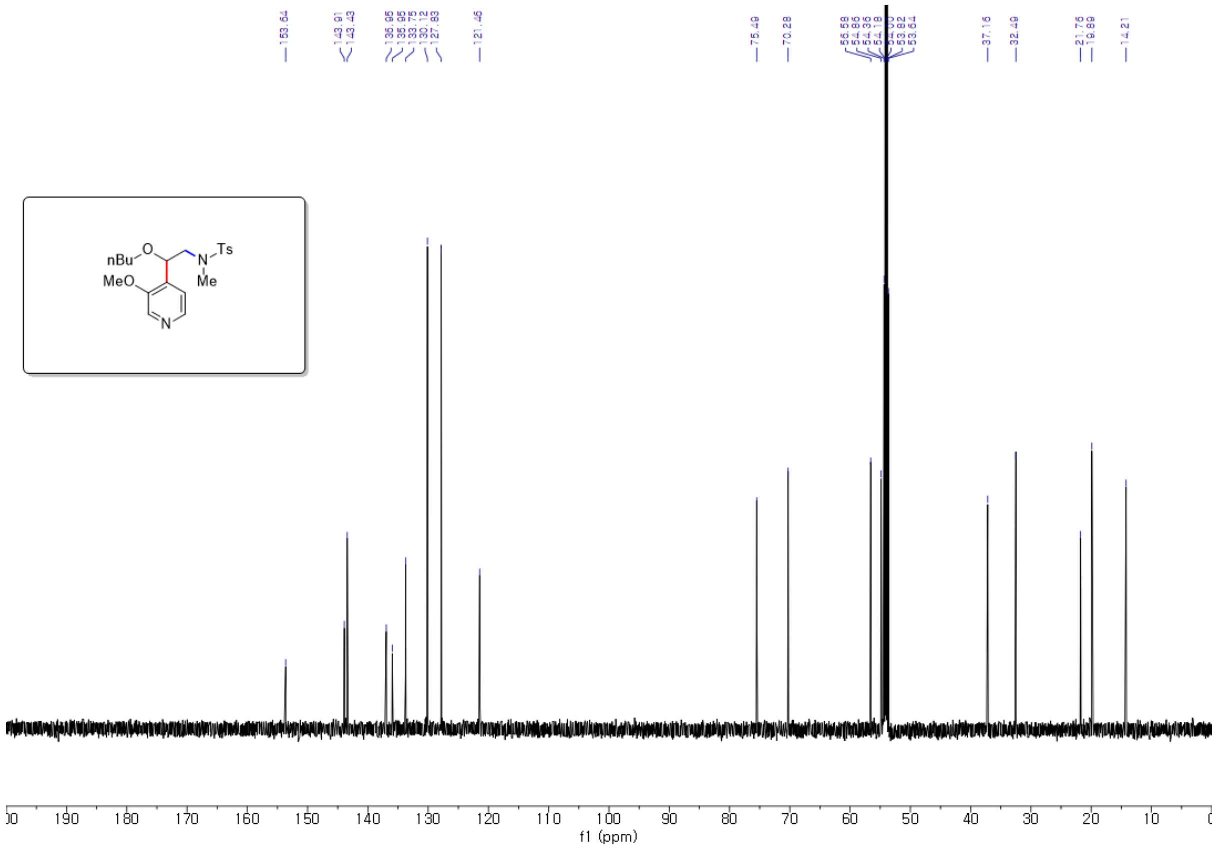

150 MHz, ^{13}C NMR in CD_2Cl_2

Supplementary Figure 51. 1H and 13C NMR of **4j**

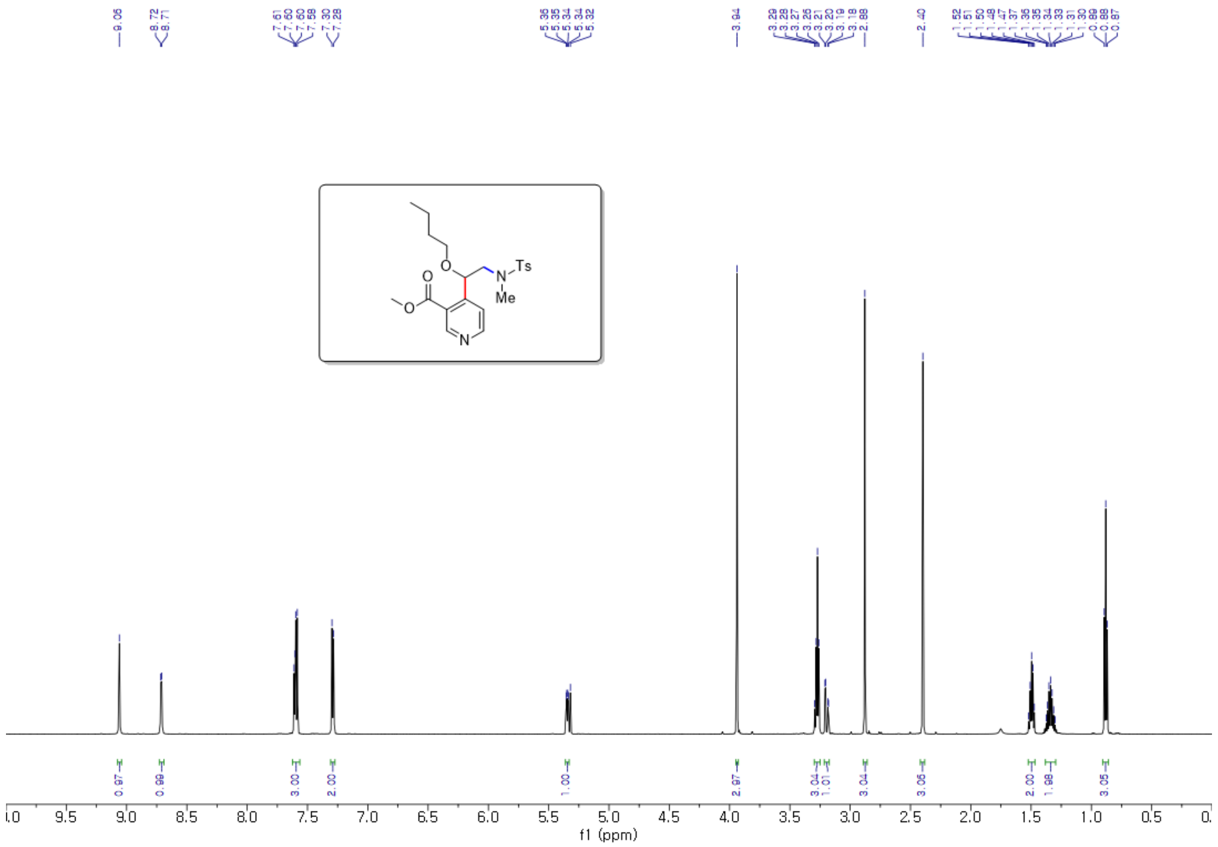

600 MHz, ^{1}H NMR in CD_2Cl_2

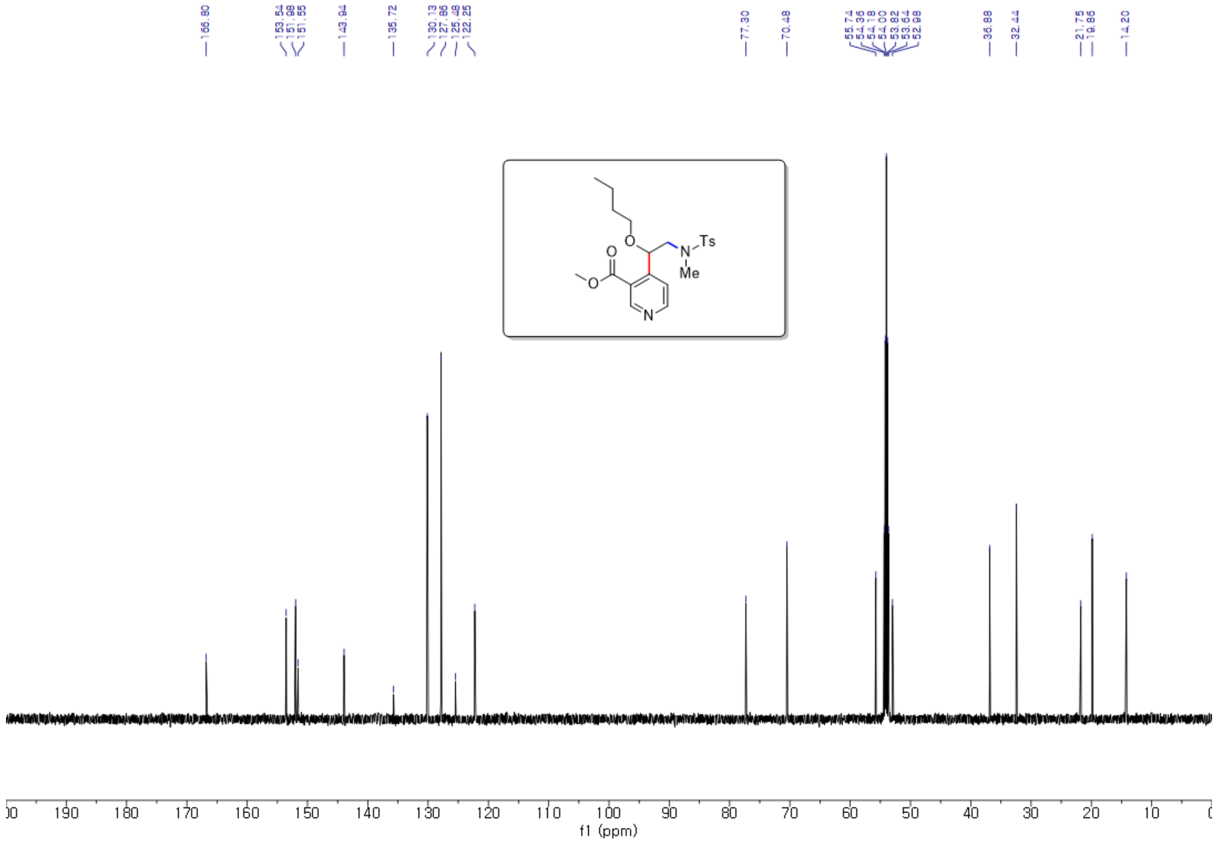

150 MHz, ^{13}C NMR in CD_2Cl_2

Supplementary Figure 52. 1H and 13C NMR of **4k**

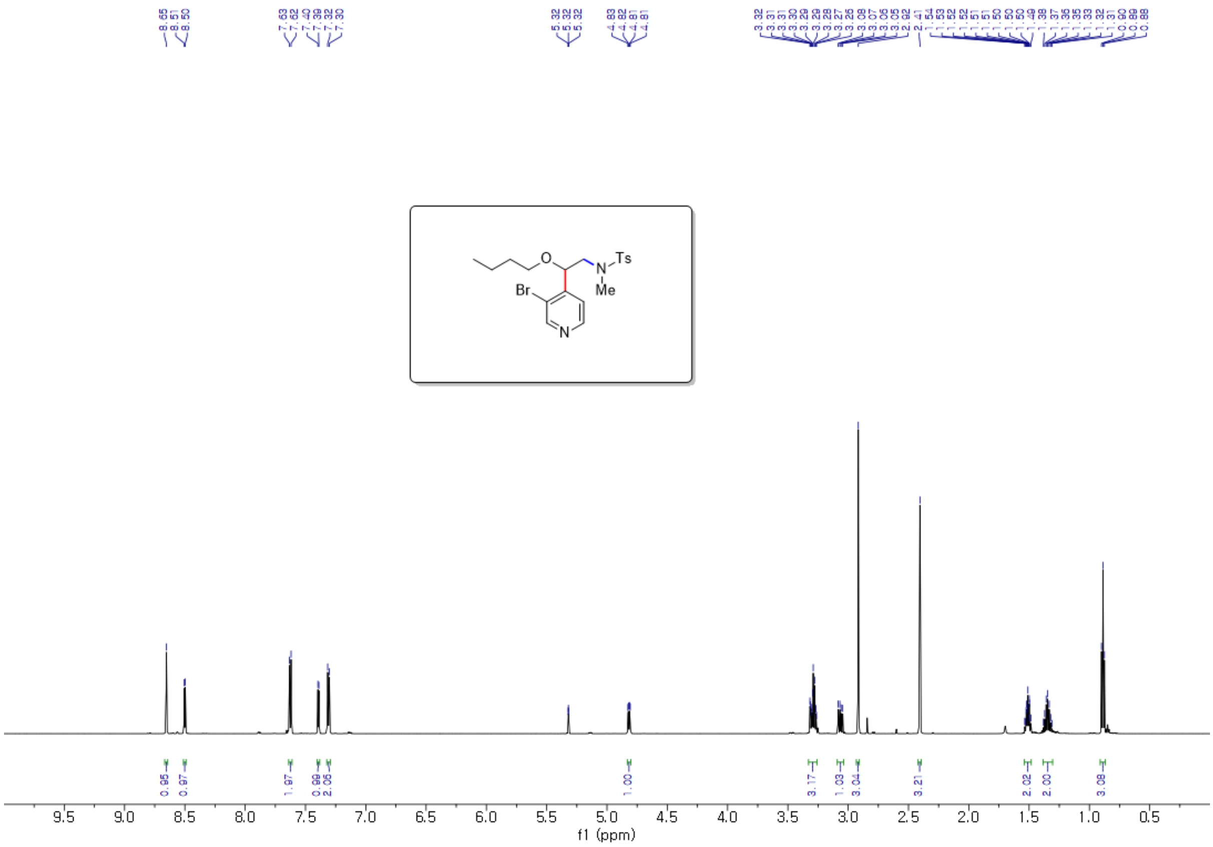

600 MHz, ^{1}H NMR in CD_2Cl_2

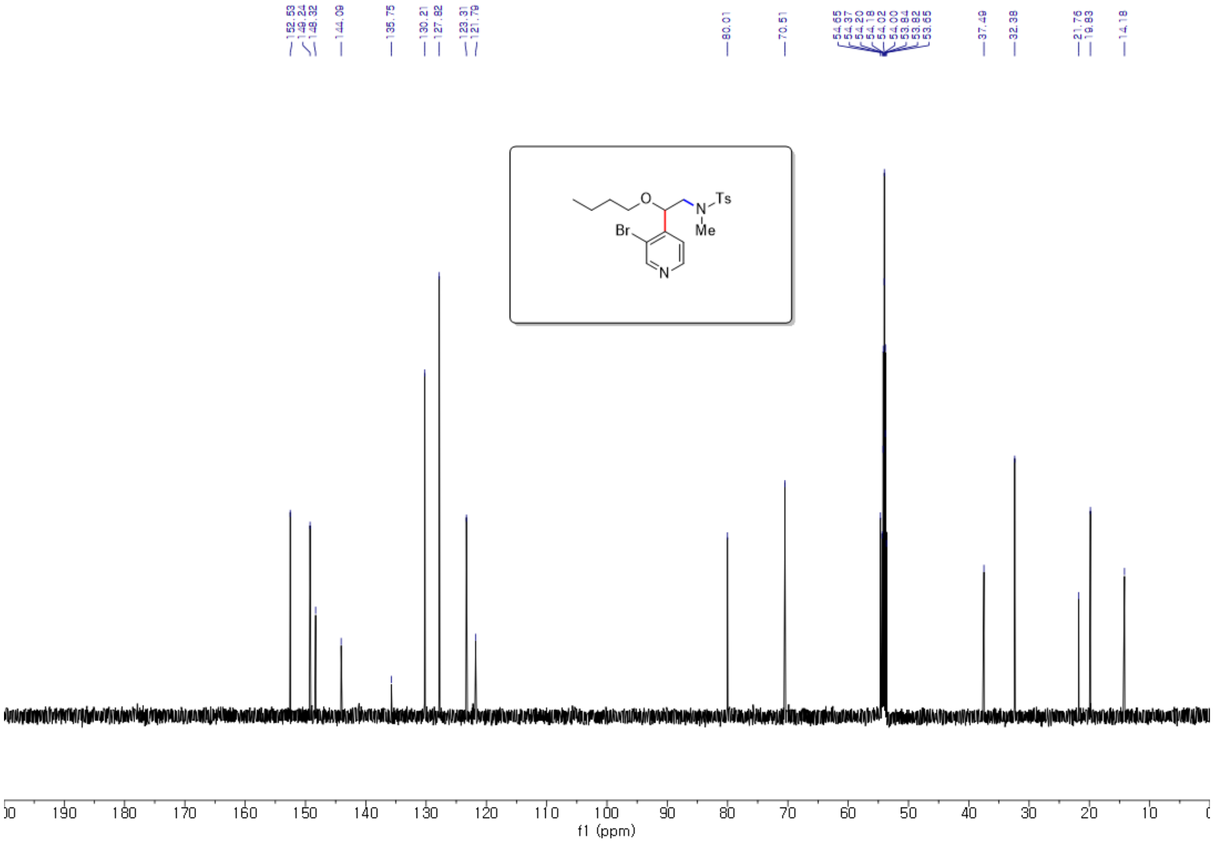

150 MHz, ^{13}C NMR in CD_2Cl_2

Supplementary Figure 53. 1H and 13C NMR of **4l**

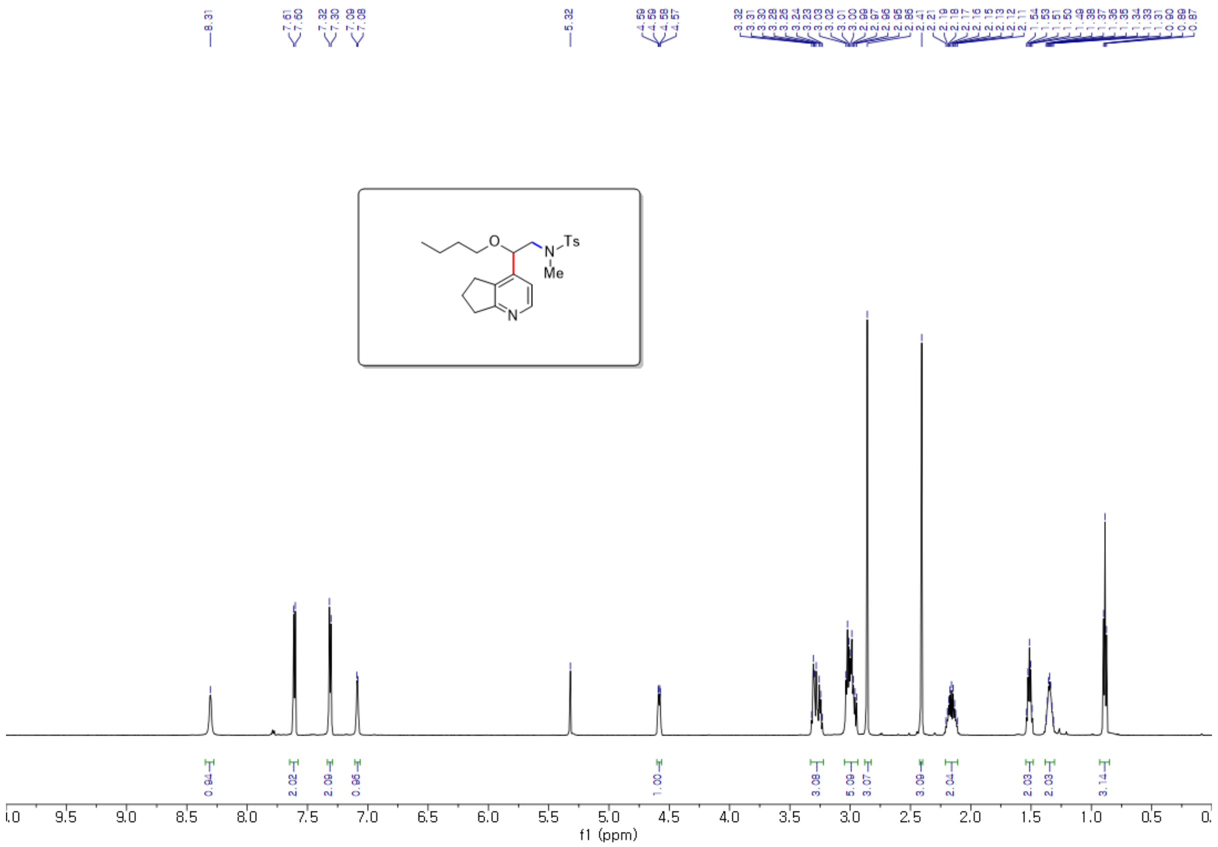

600 MHz, ^{1}H NMR in CD_2Cl_2

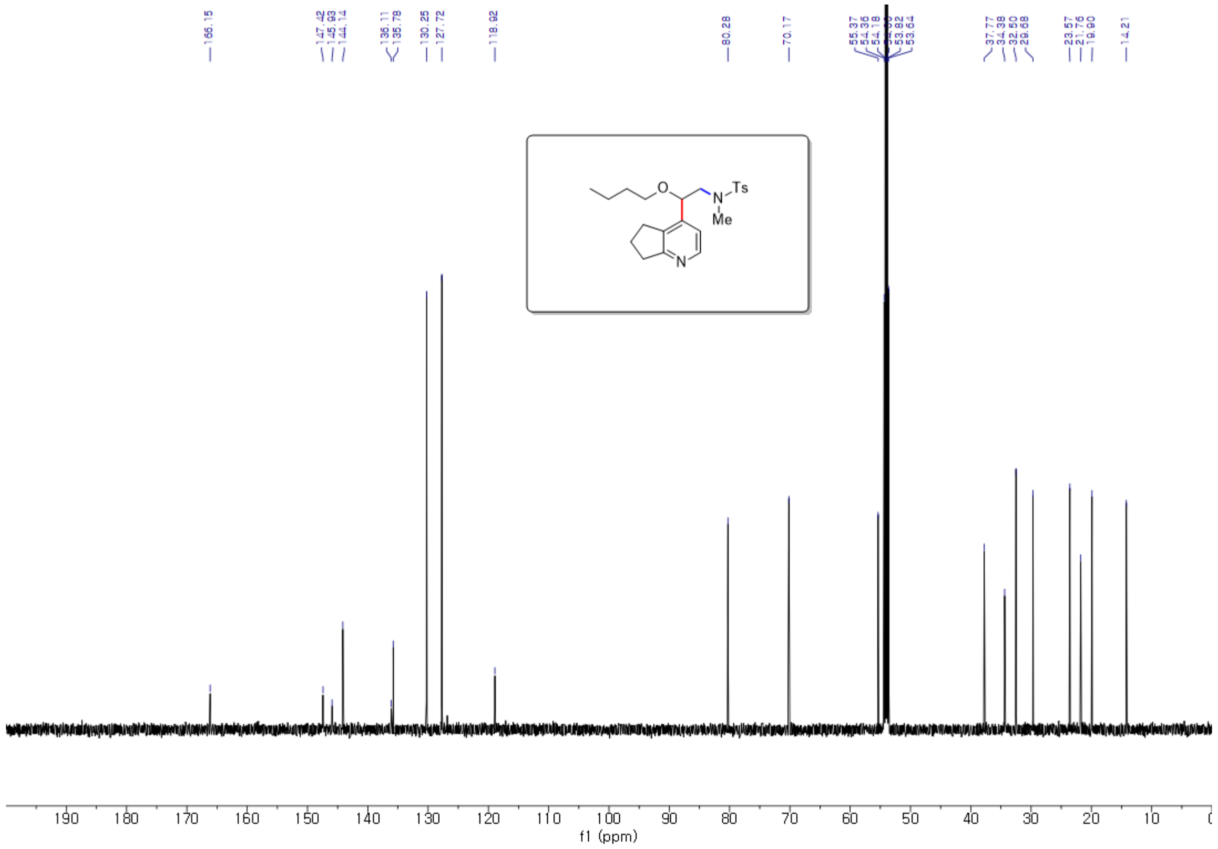

150 MHz, ^{13}C NMR in CD_2Cl_2

Supplementary Figure 54. 1H and 13C NMR of **4m**

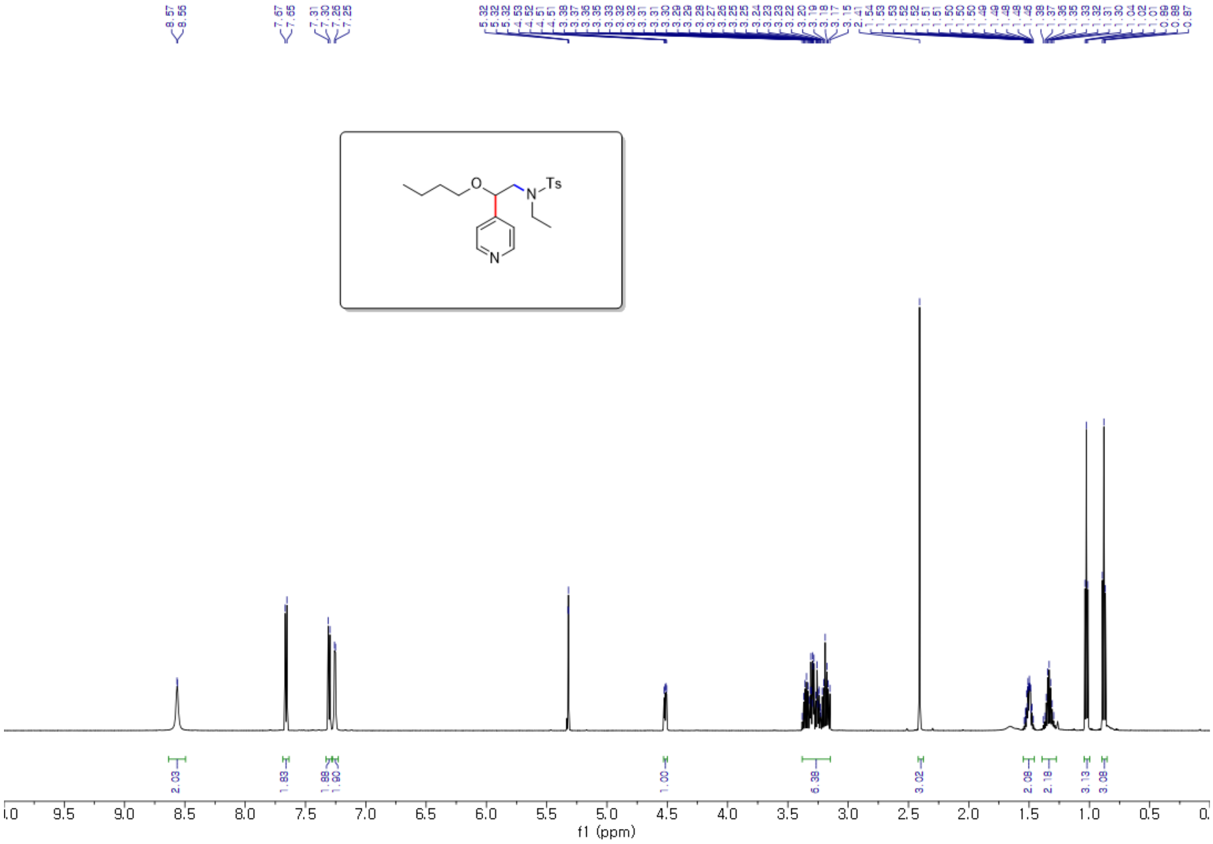

600 MHz, ^{1}H NMR in CD_2Cl_2

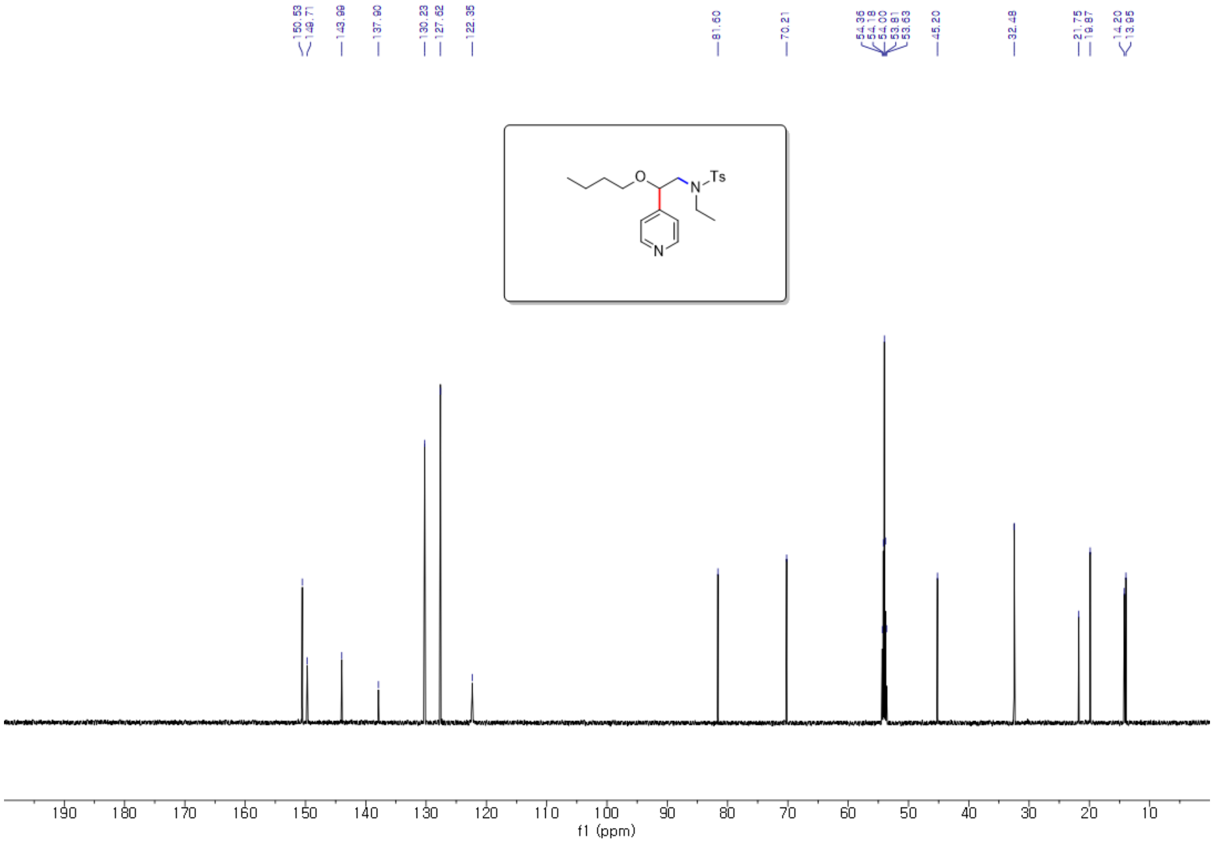

150 MHz, ^{13}C NMR in CD_2Cl_2

Supplementary Figure 55. 1H and 13C NMR of **4n**

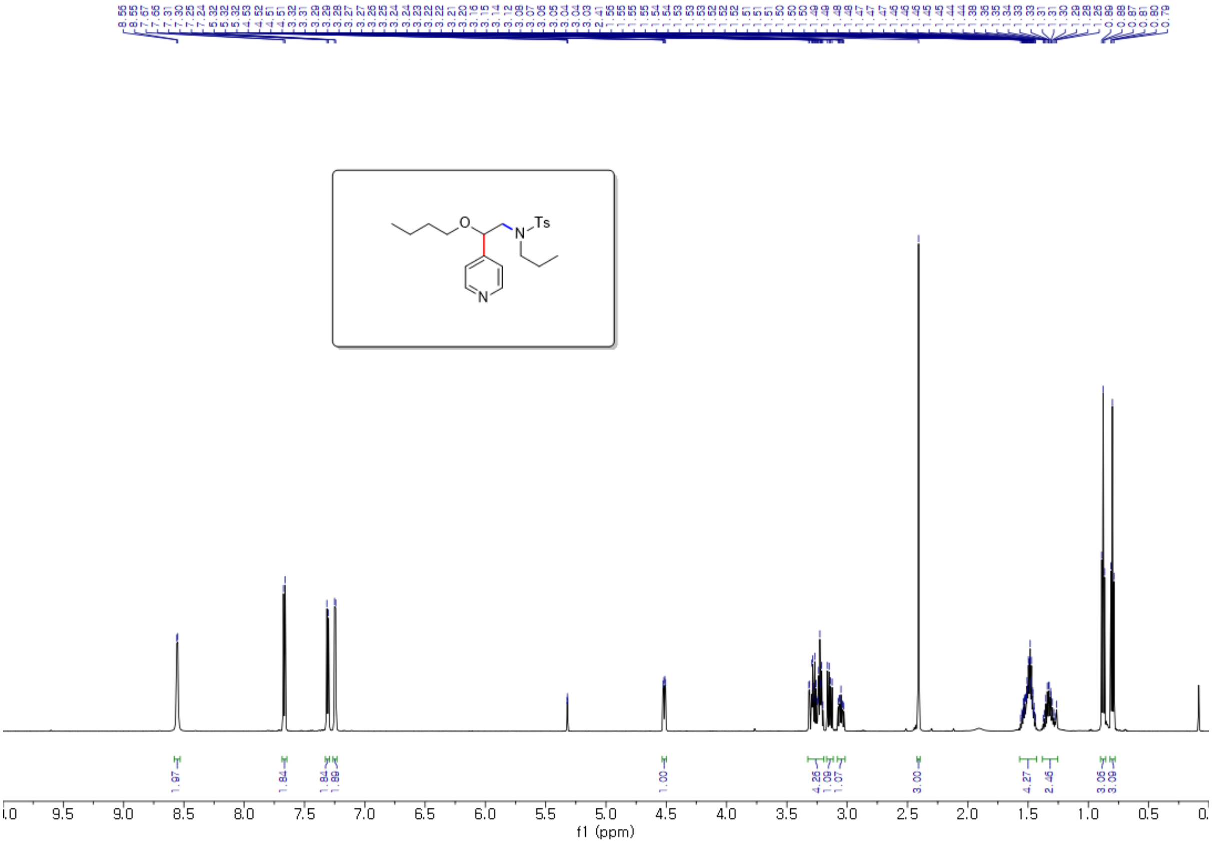

600 MHz, ^{1}H NMR in CD_2Cl_2

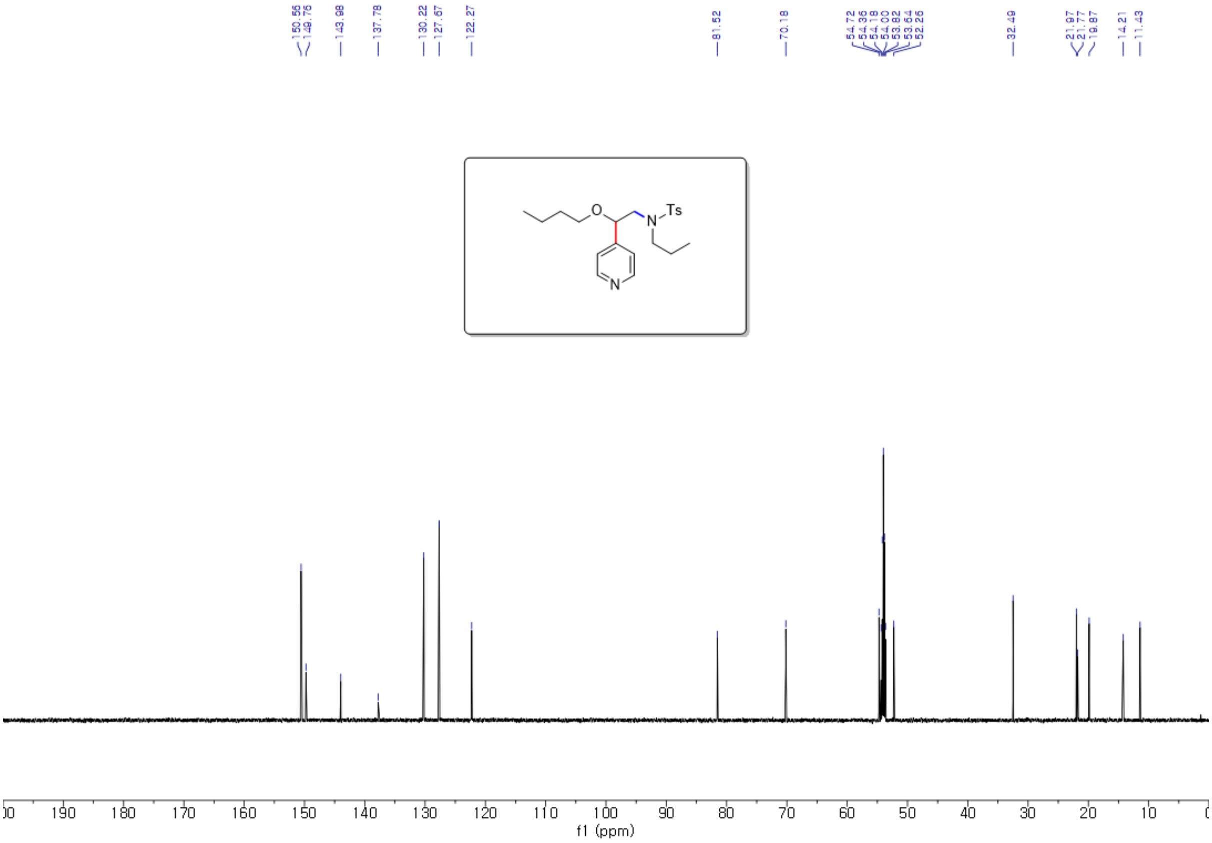

150 MHz, ^{13}C NMR in CD_2Cl_2

Supplementary Figure 56. 1H and 13C NMR of **4o**

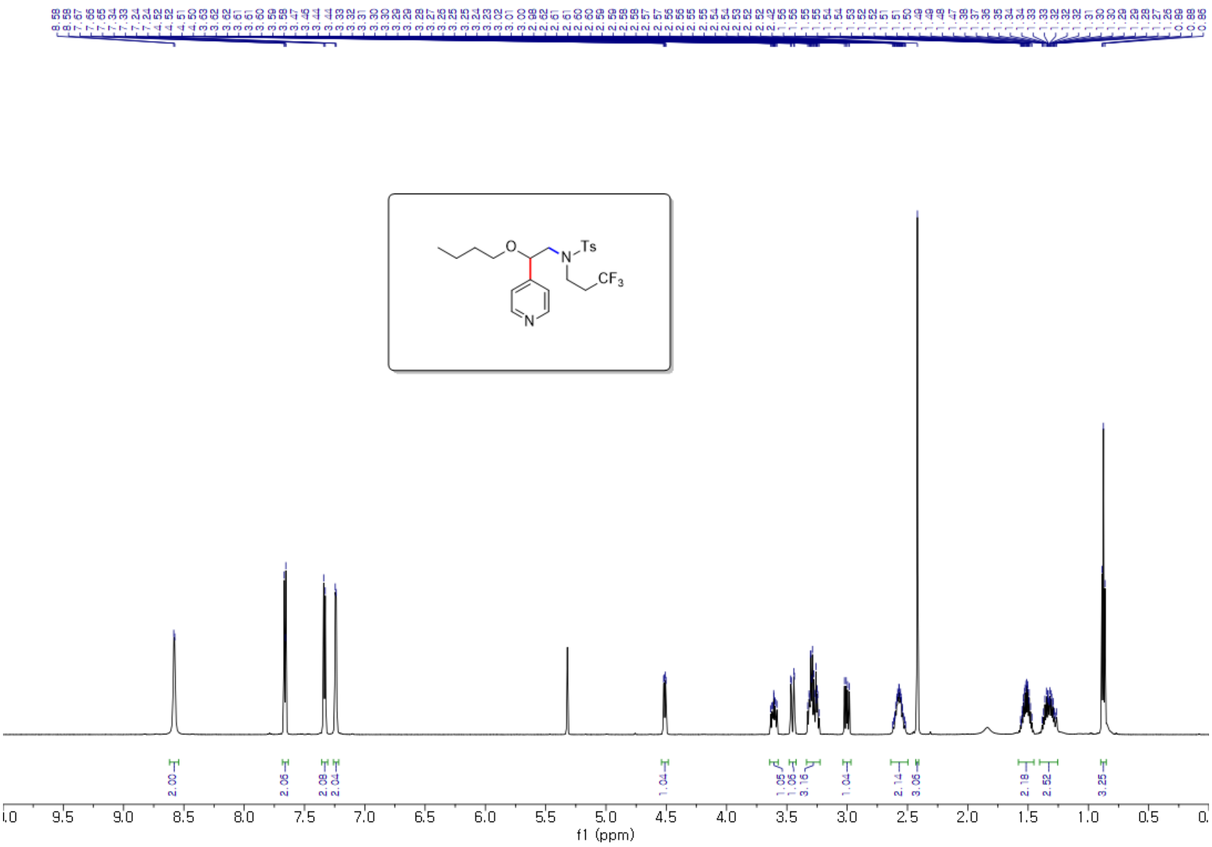

600 MHz, ^{1}H NMR in CD_2Cl_2

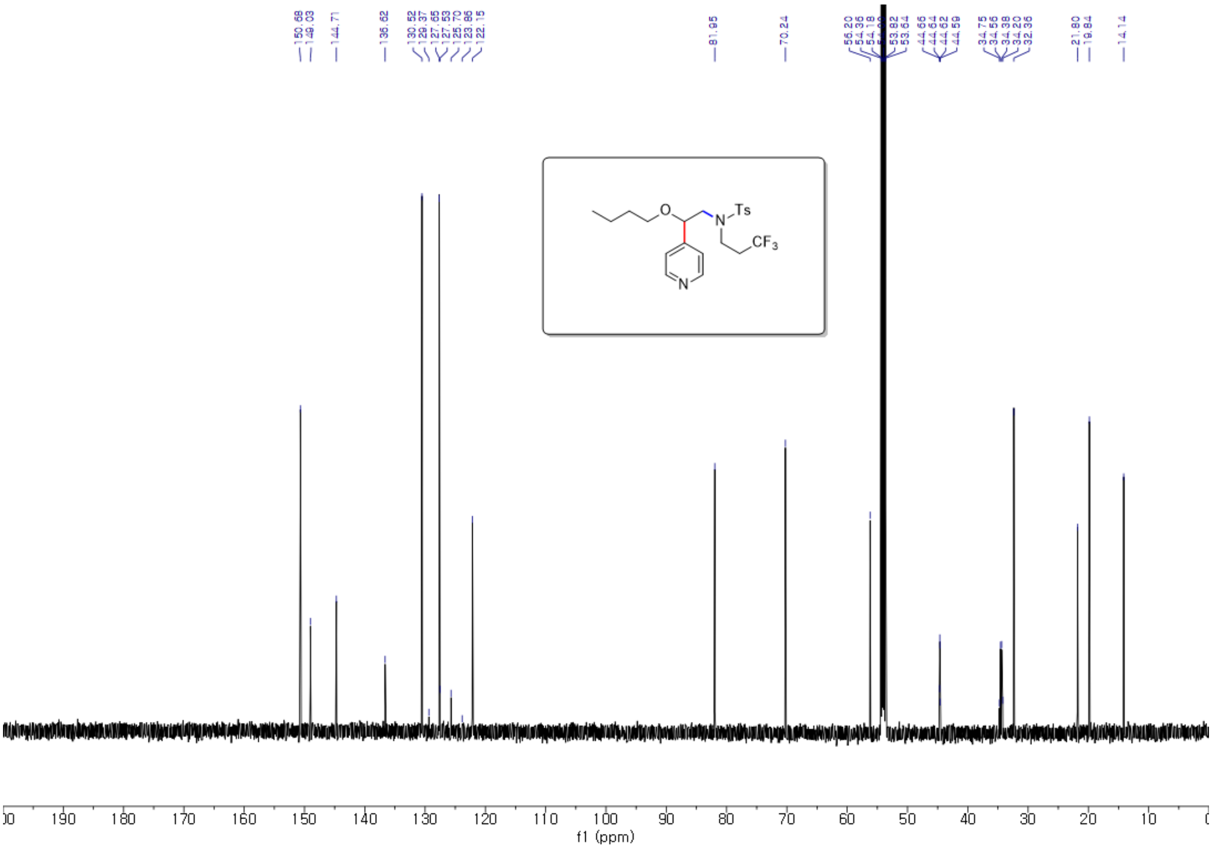

150 MHz, ^{13}C NMR in CD_2Cl_2

Supplementary Figure 57. 1H and 13C NMR of **4p**

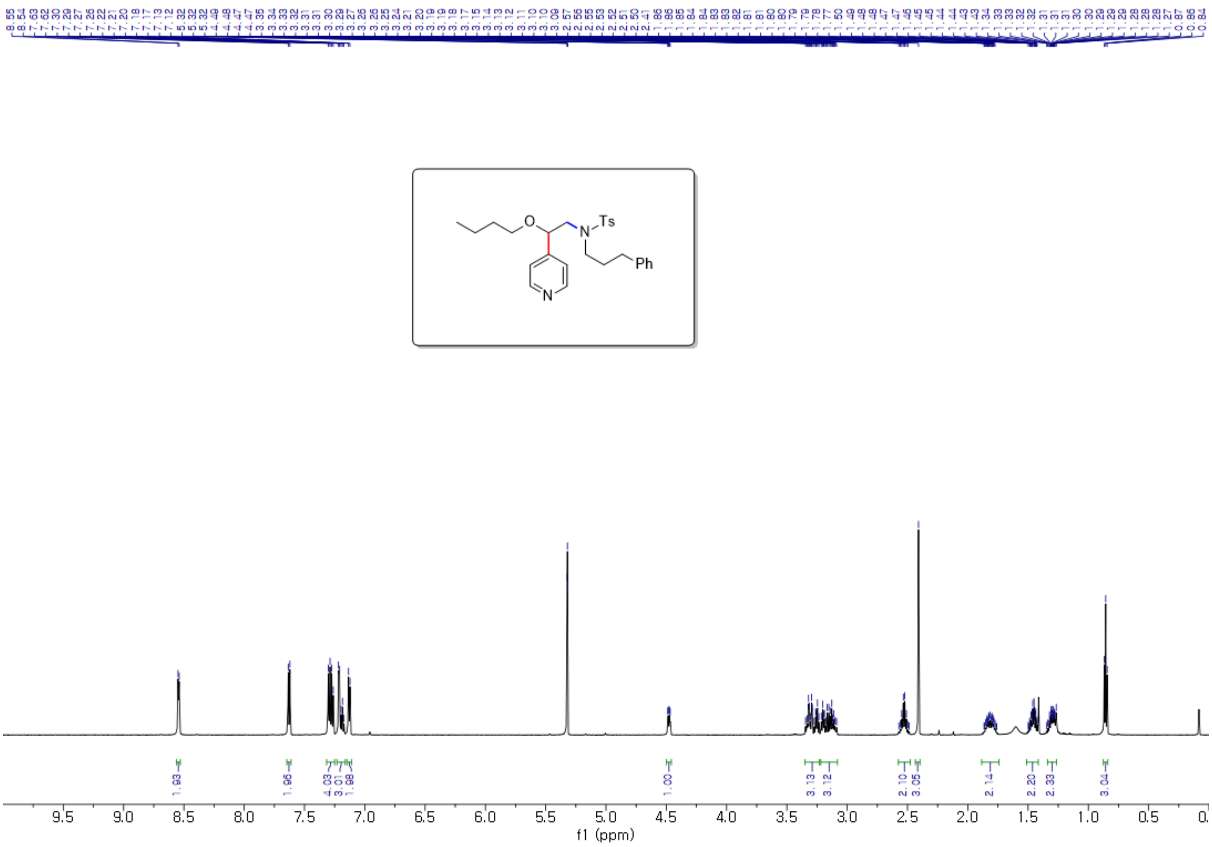

600 MHz, ^{1}H NMR in CD_2Cl_2

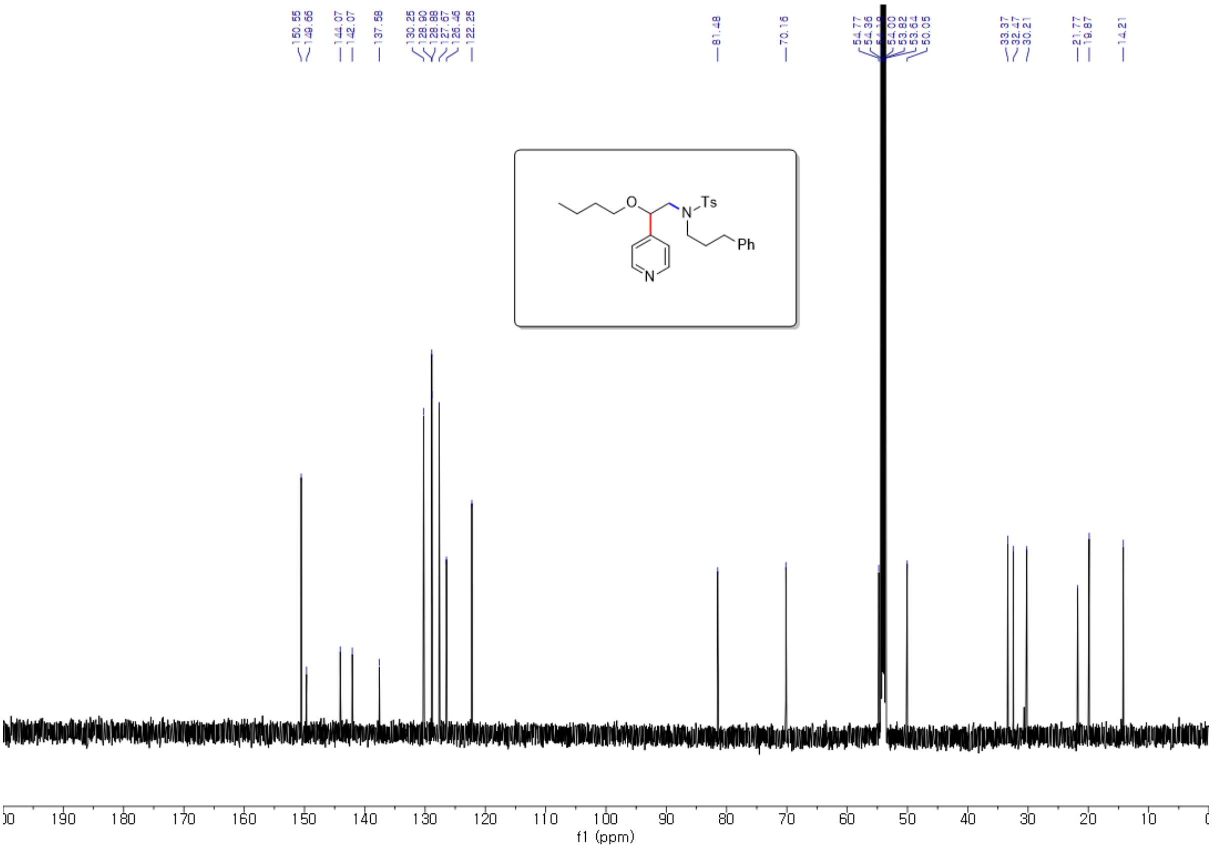

150 MHz, ^{13}C NMR in CD_2Cl_2

Supplementary Figure 58. 1H and 13C NMR of **4q**

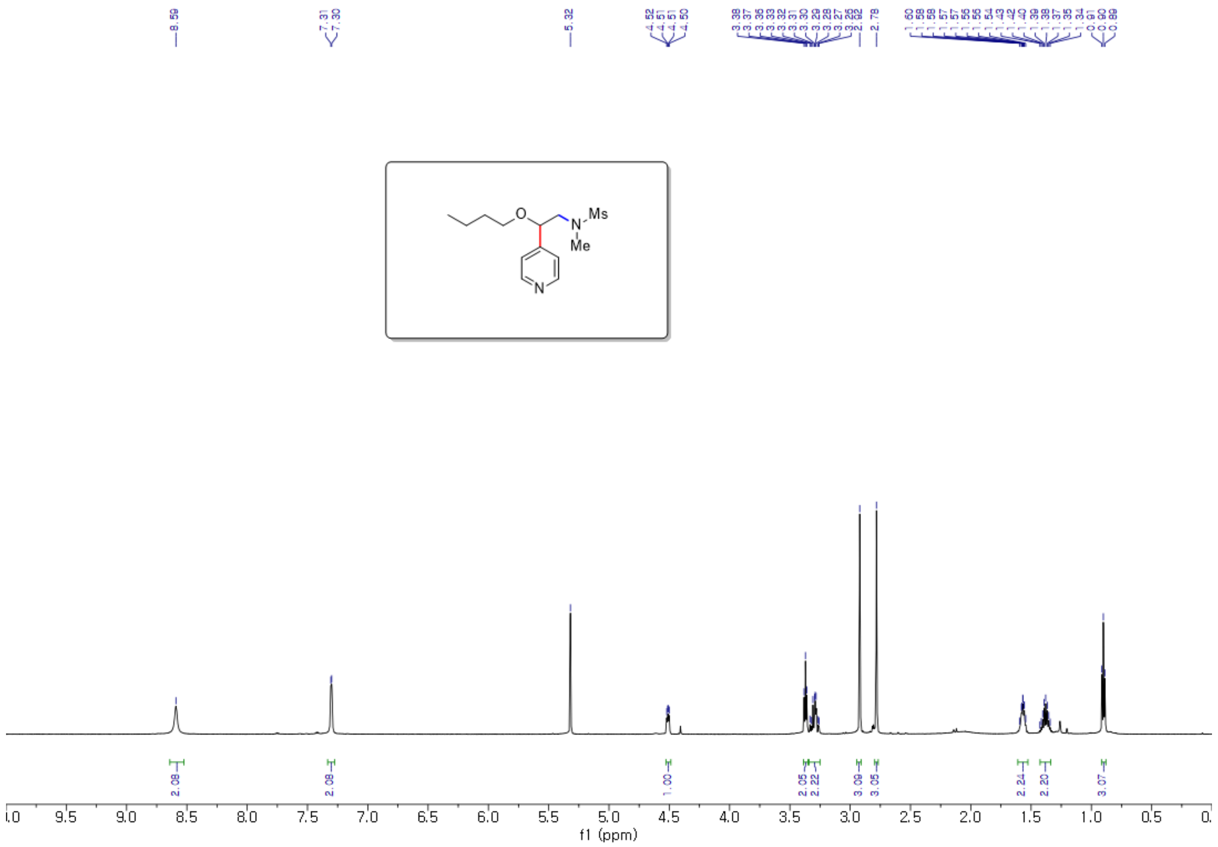

600 MHz, ^{1}H NMR in CD_2Cl_2

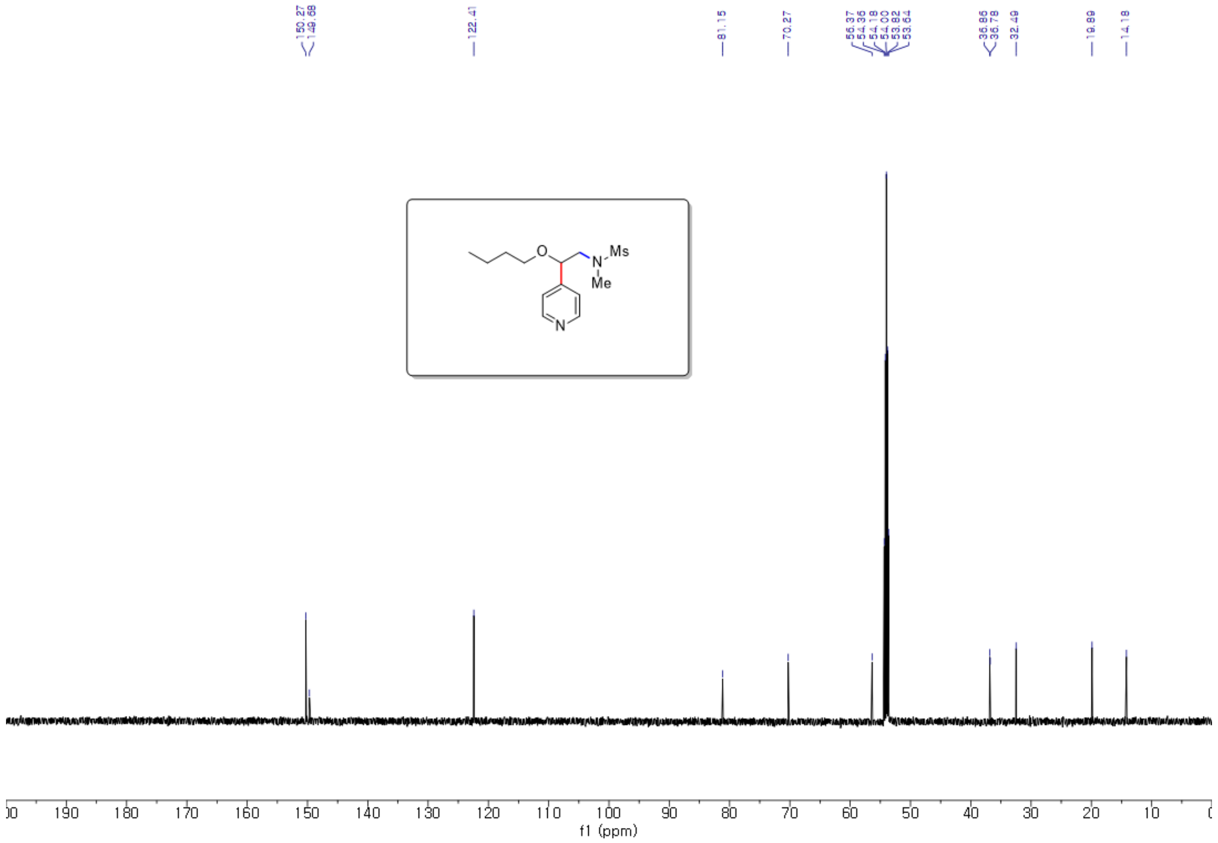

150 MHz, ^{13}C NMR in CD_2Cl_2

Supplementary Figure 59. 1H and 13C NMR of **4r**

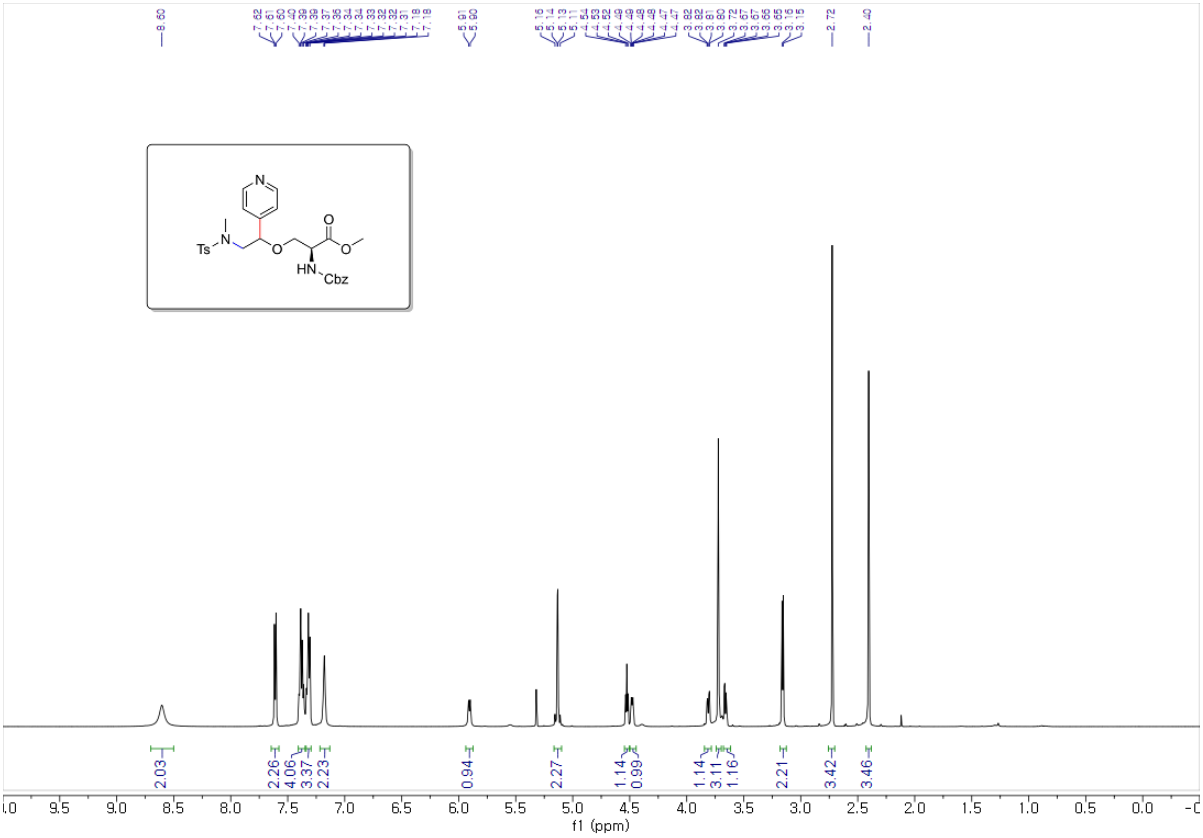

600 MHz, ^{1}H NMR in CD_2Cl_2

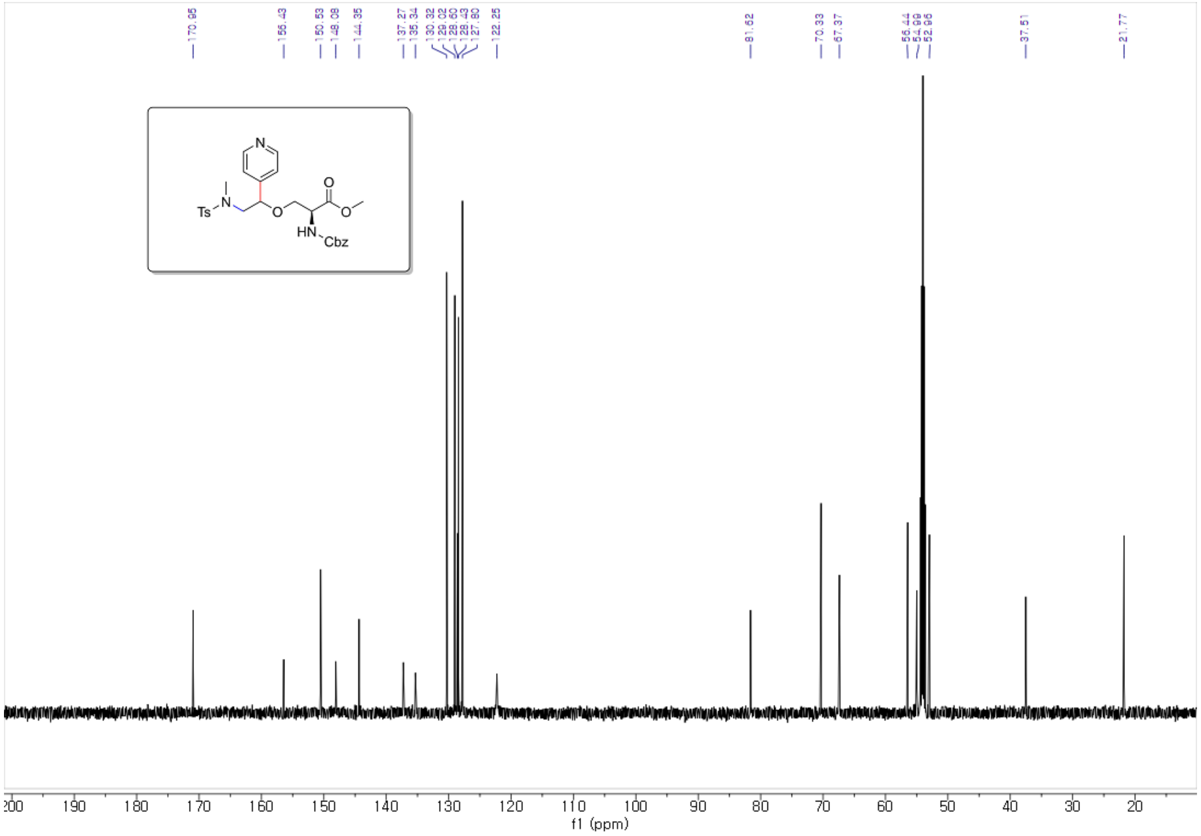

150 MHz, ^{13}C NMR in CD_2Cl_2

Supplementary Figure 60. 1H and 13C NMR of **5a**

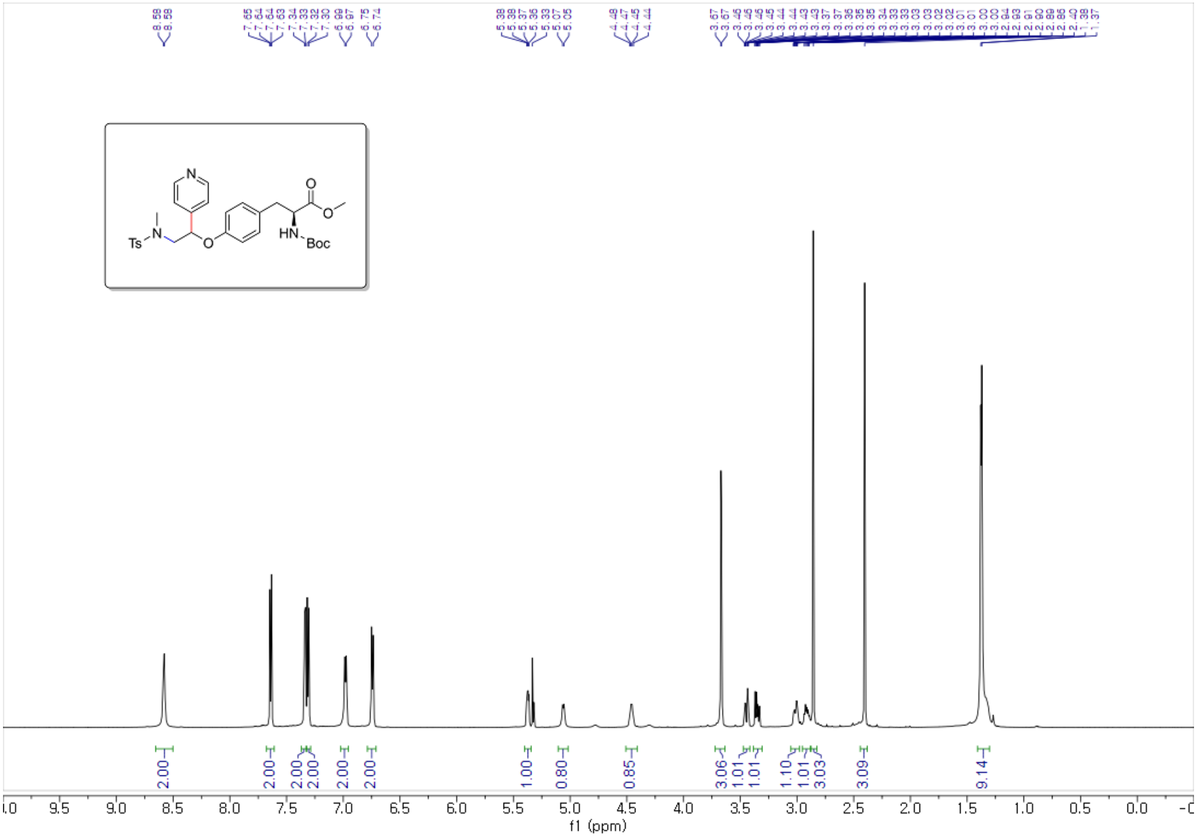

600 MHz, ^{1}H NMR in CD_2Cl_2

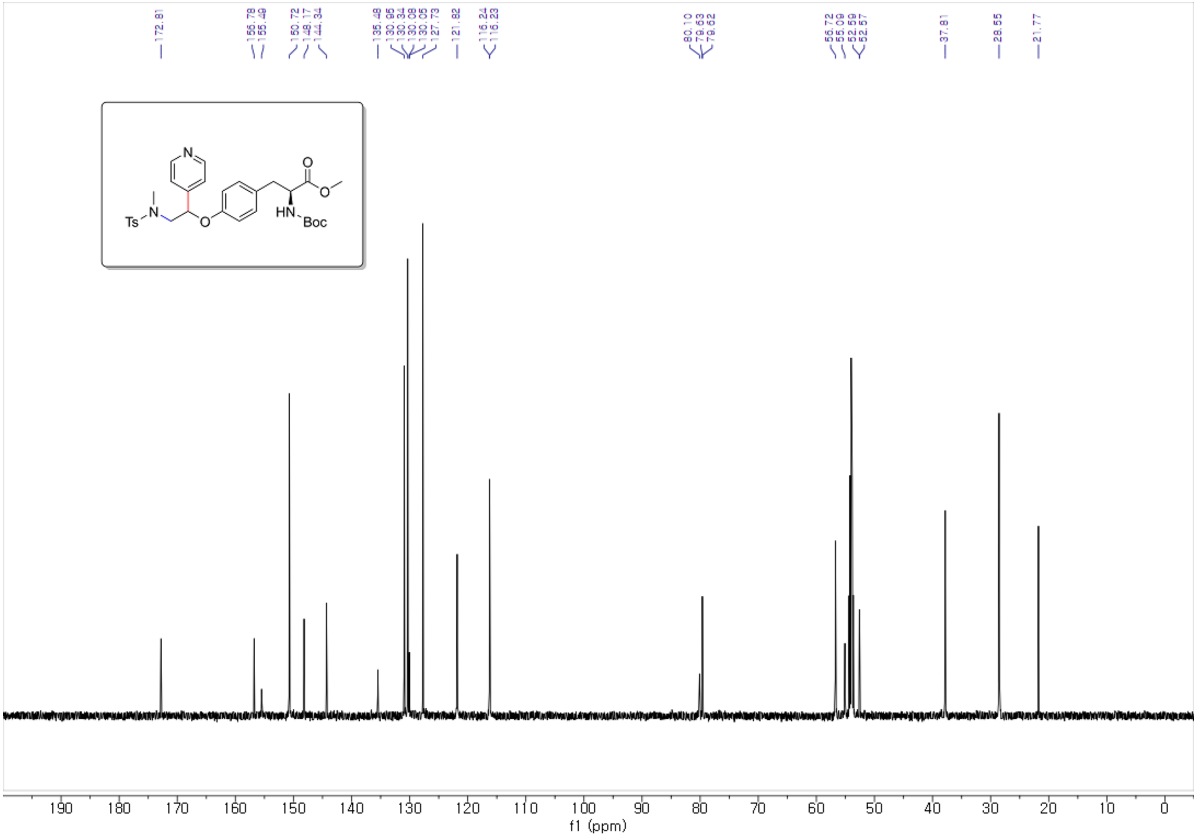

150 MHz, ^{13}C NMR in CD_2Cl_2

Supplementary Figure 61. 1H and 13C NMR of **5b**

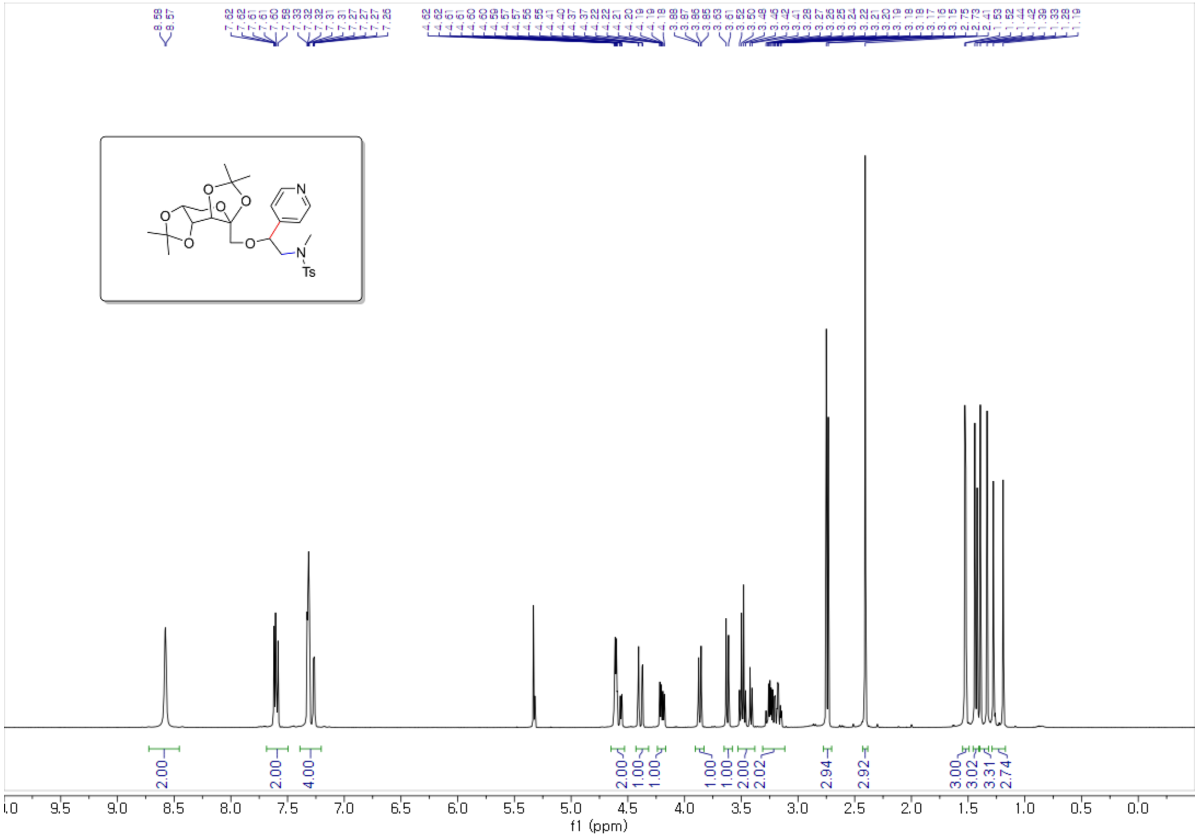

600 MHz, ^{1}H NMR in CD_2Cl_2

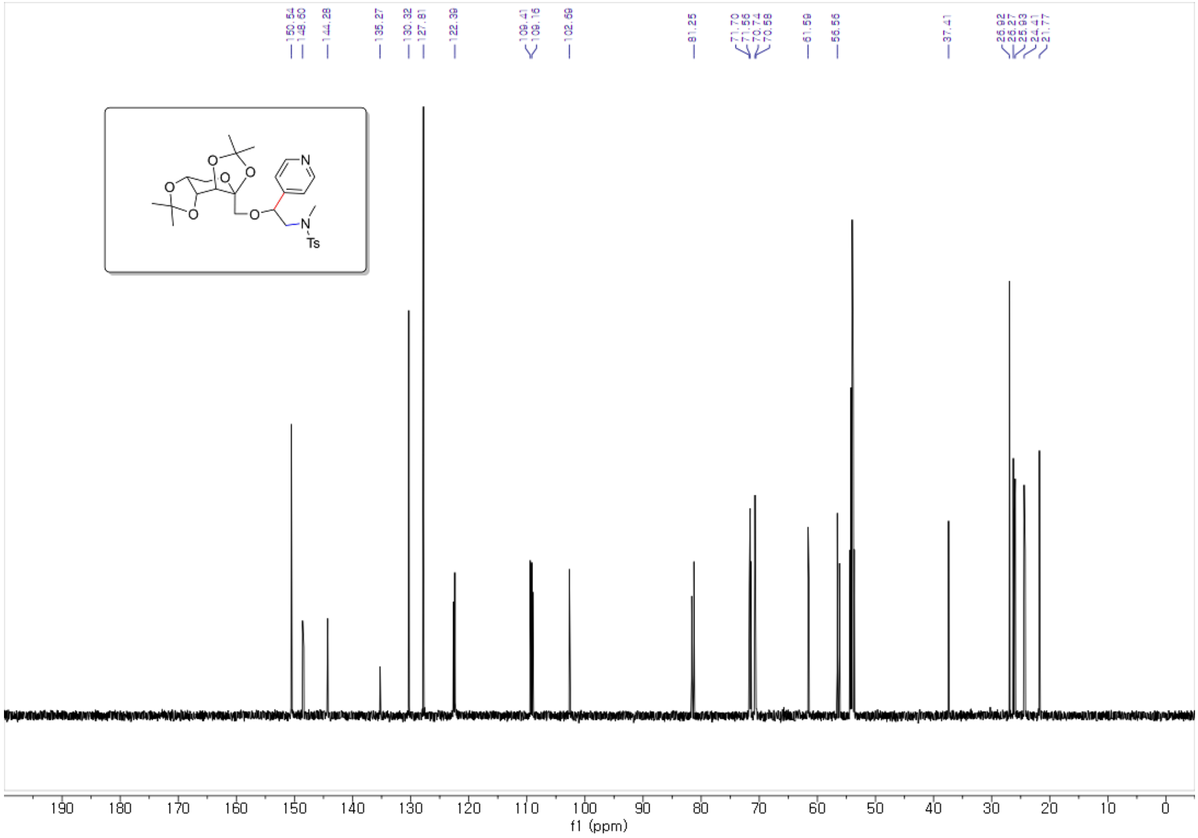

150 MHz, ^{13}C NMR in CD_2Cl_2

Supplementary Figure 62. 1H and 13C NMR of **5c**

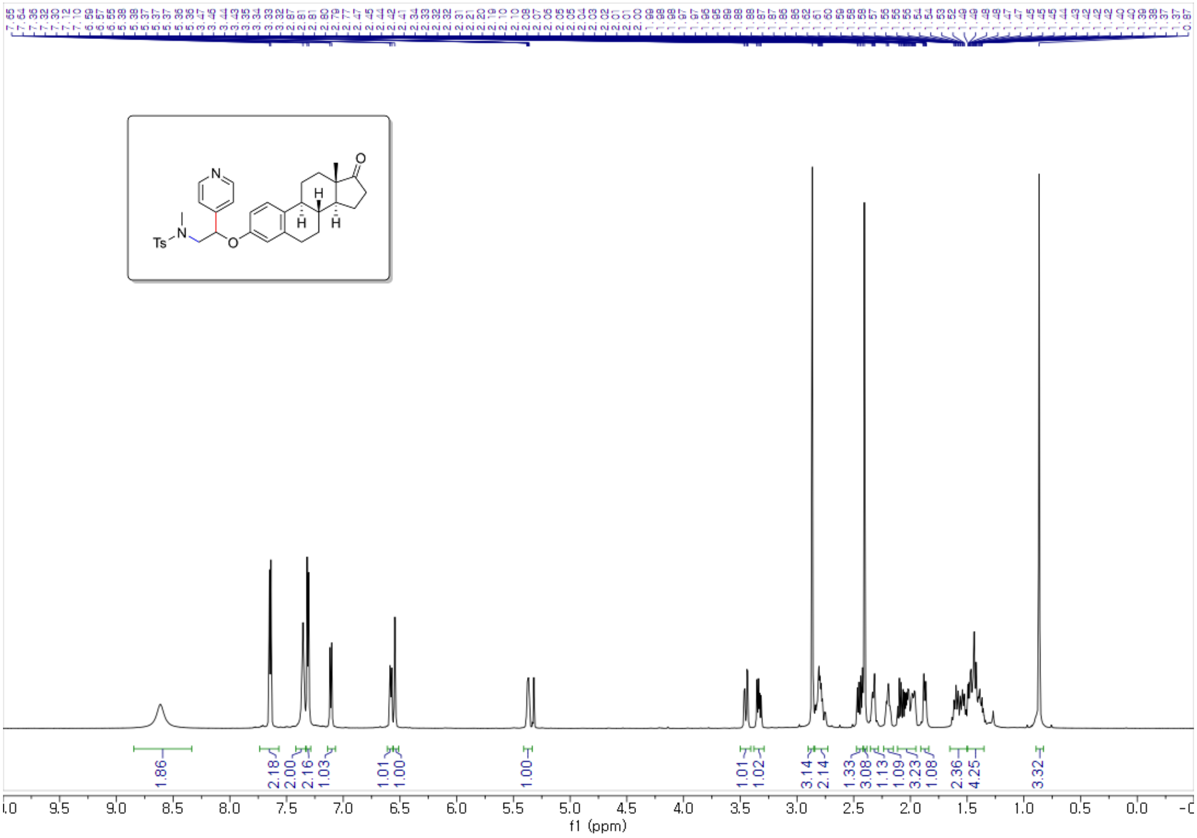

600 MHz, ^{1}H NMR in CD_2Cl_2

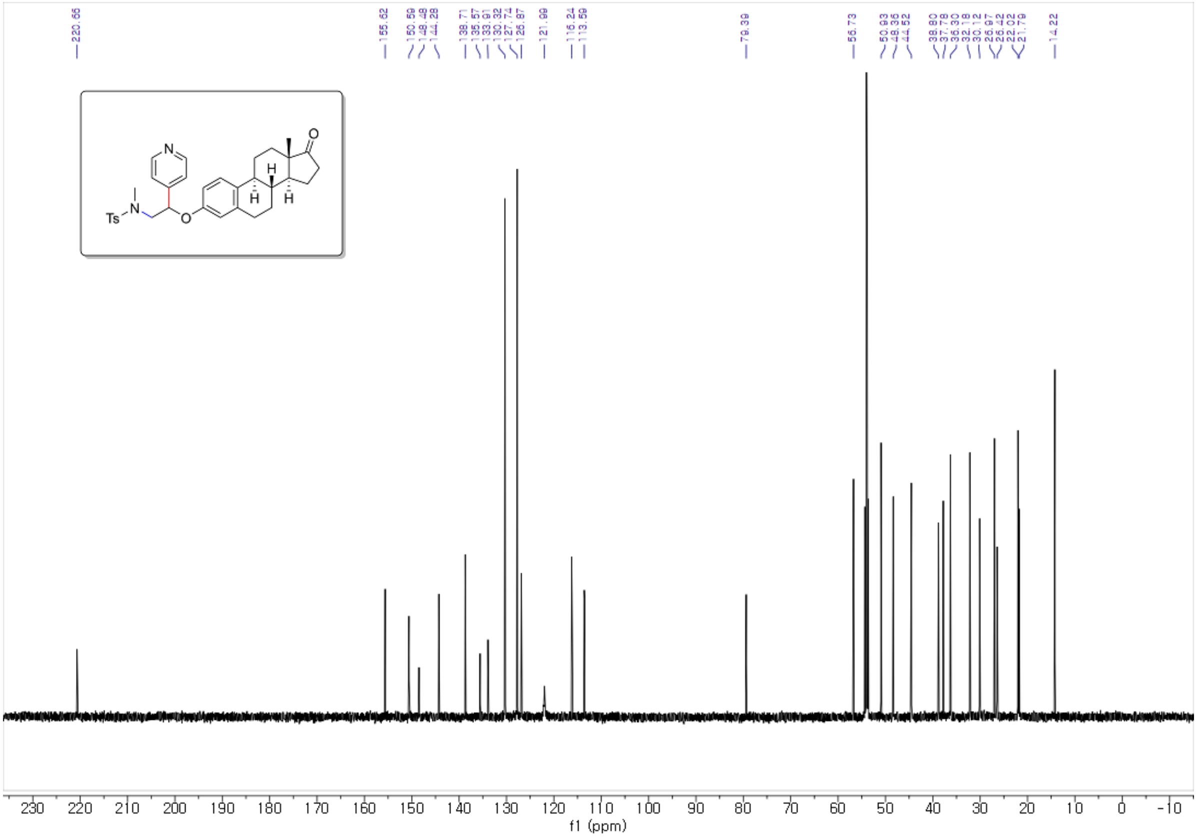

150 MHz, ^{13}C NMR in CD_2Cl_2

Supplementary Figure 63. 1H and 13C NMR of **5d**

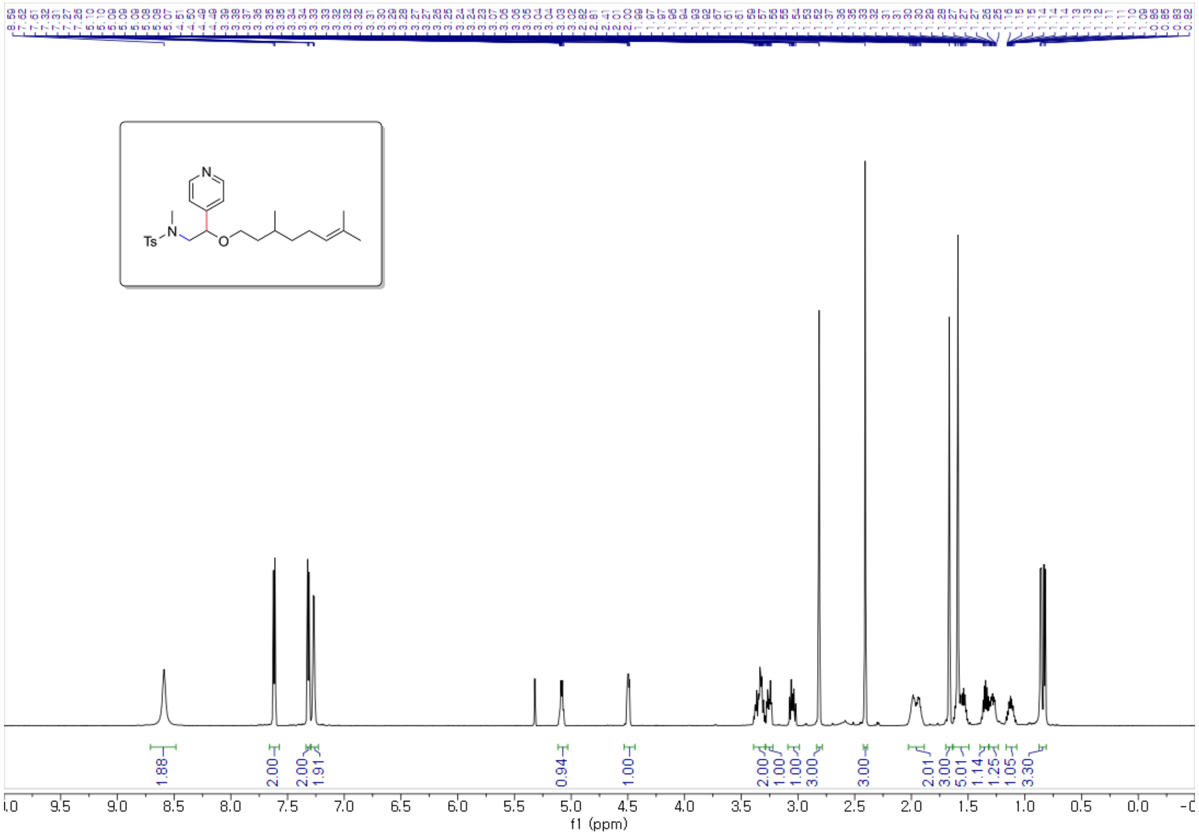

600 MHz, ^{1}H NMR in CD_2Cl_2

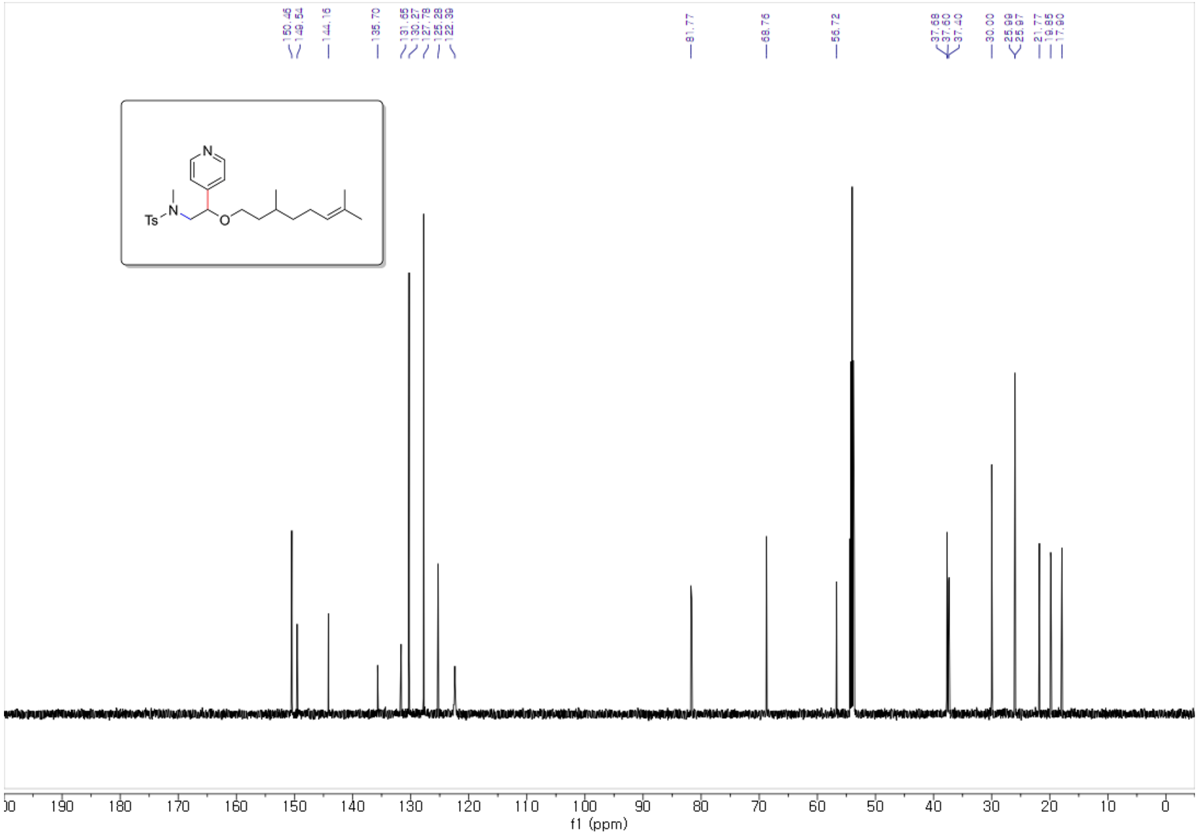

150 MHz, ^{13}C NMR in CD_2Cl_2

Supplementary Figure 64. 1H and 13C NMR of **5e**

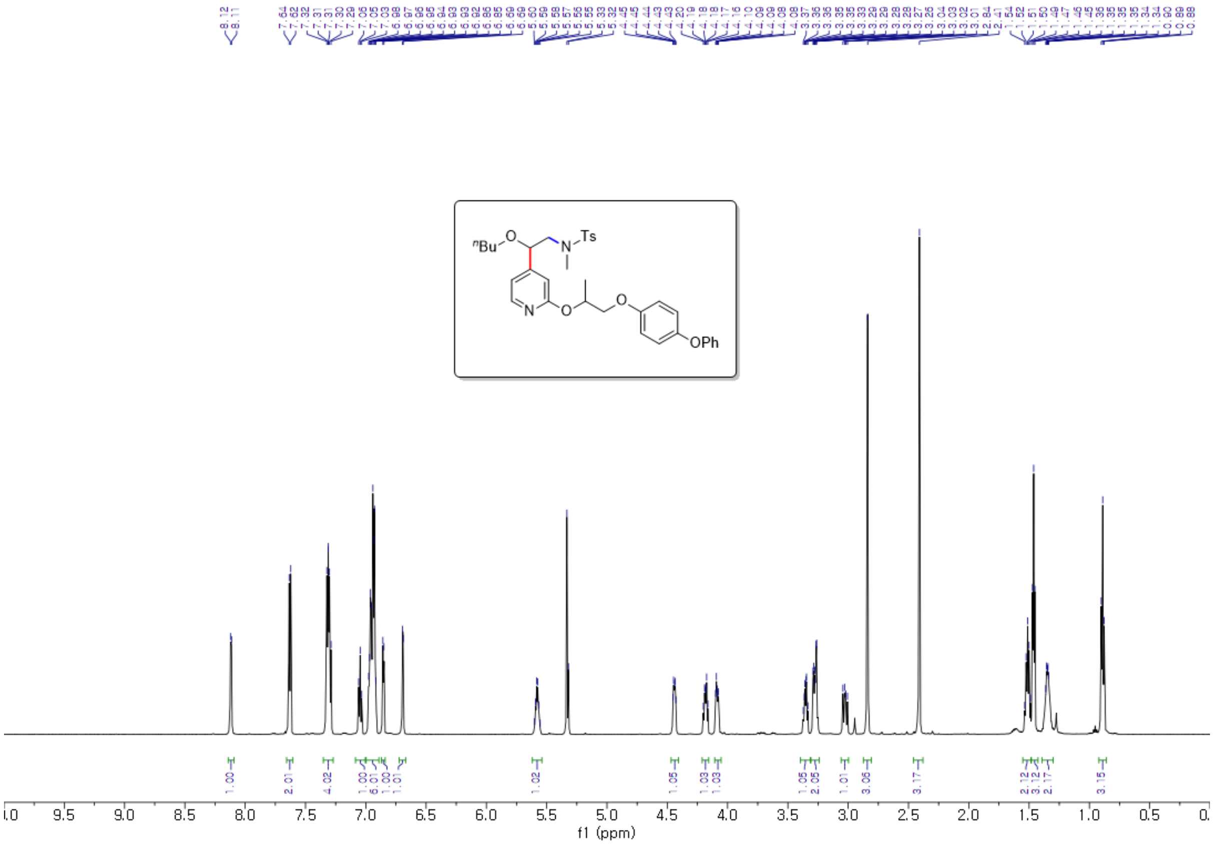

600 MHz, ^{1}H NMR in CD_2Cl_2

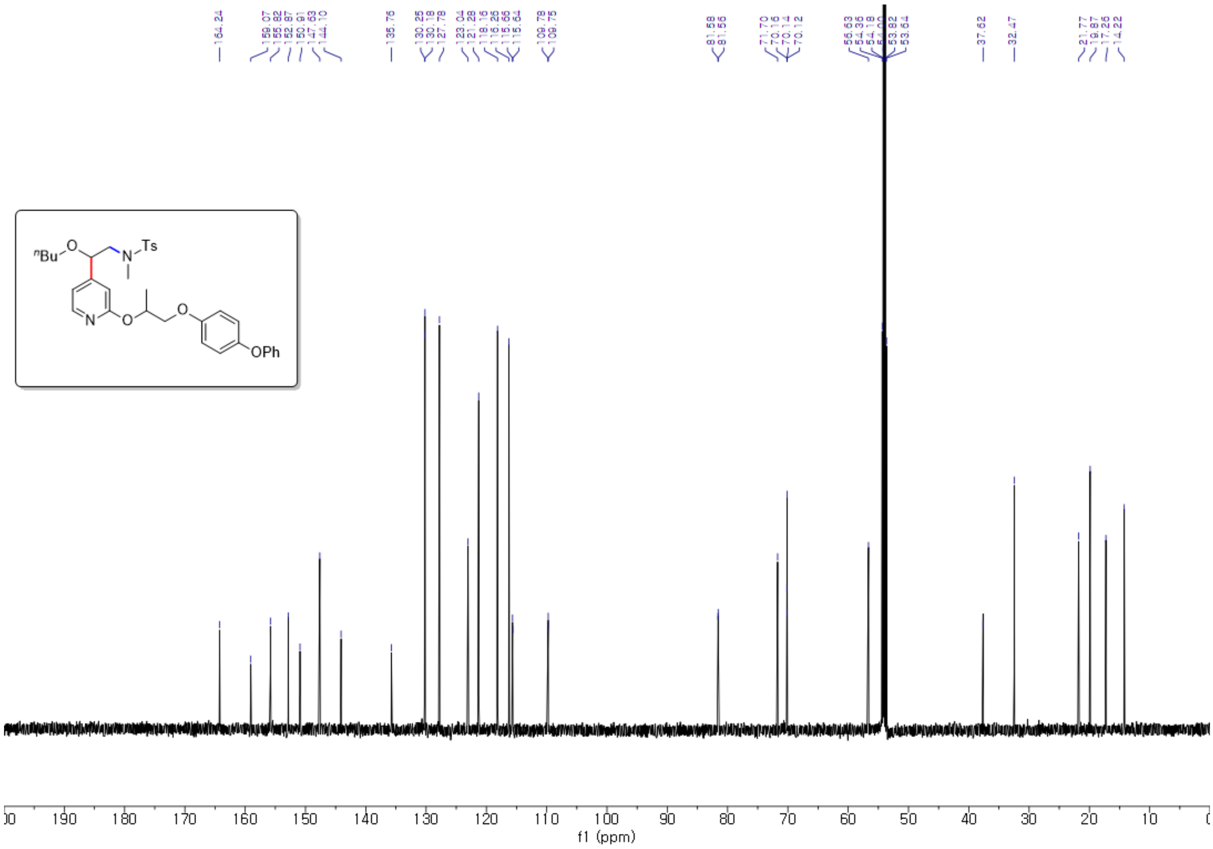

150 MHz, ^{13}C NMR in CD_2Cl_2

Supplementary Figure 65. 1H and 13C NMR of **5f**

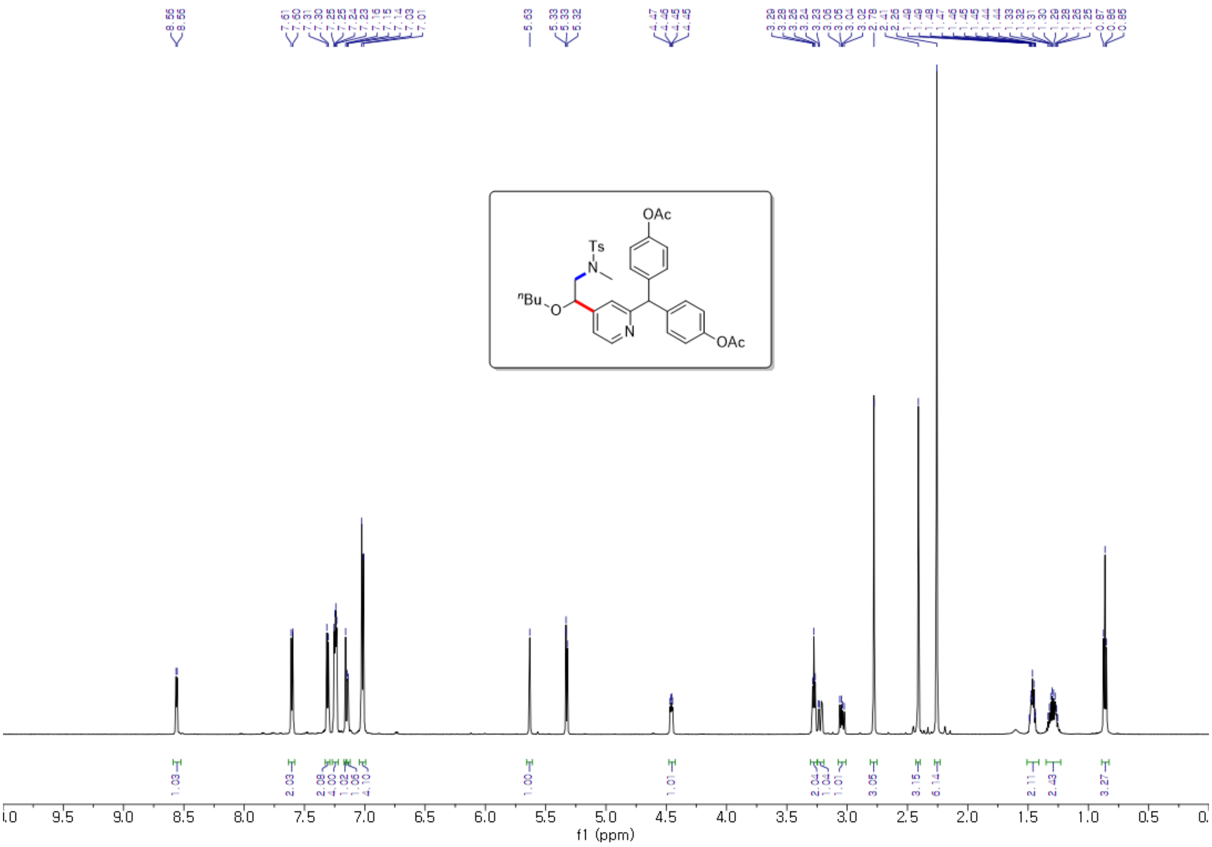

600 MHz, ^{1}H NMR in CD_2Cl_2

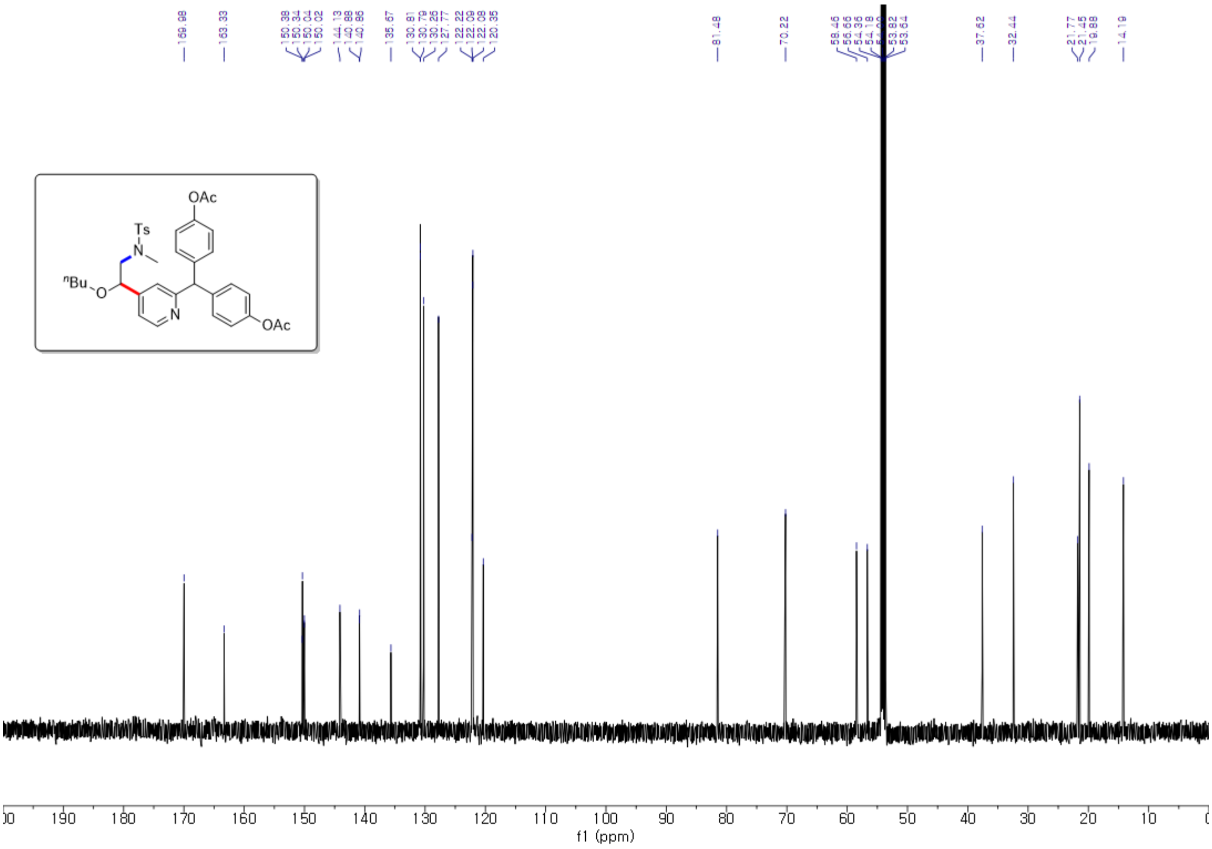

150 MHz, ^{13}C NMR in CD_2Cl_2

Supplementary Figure 66. 1H and 13C NMR of **5g**

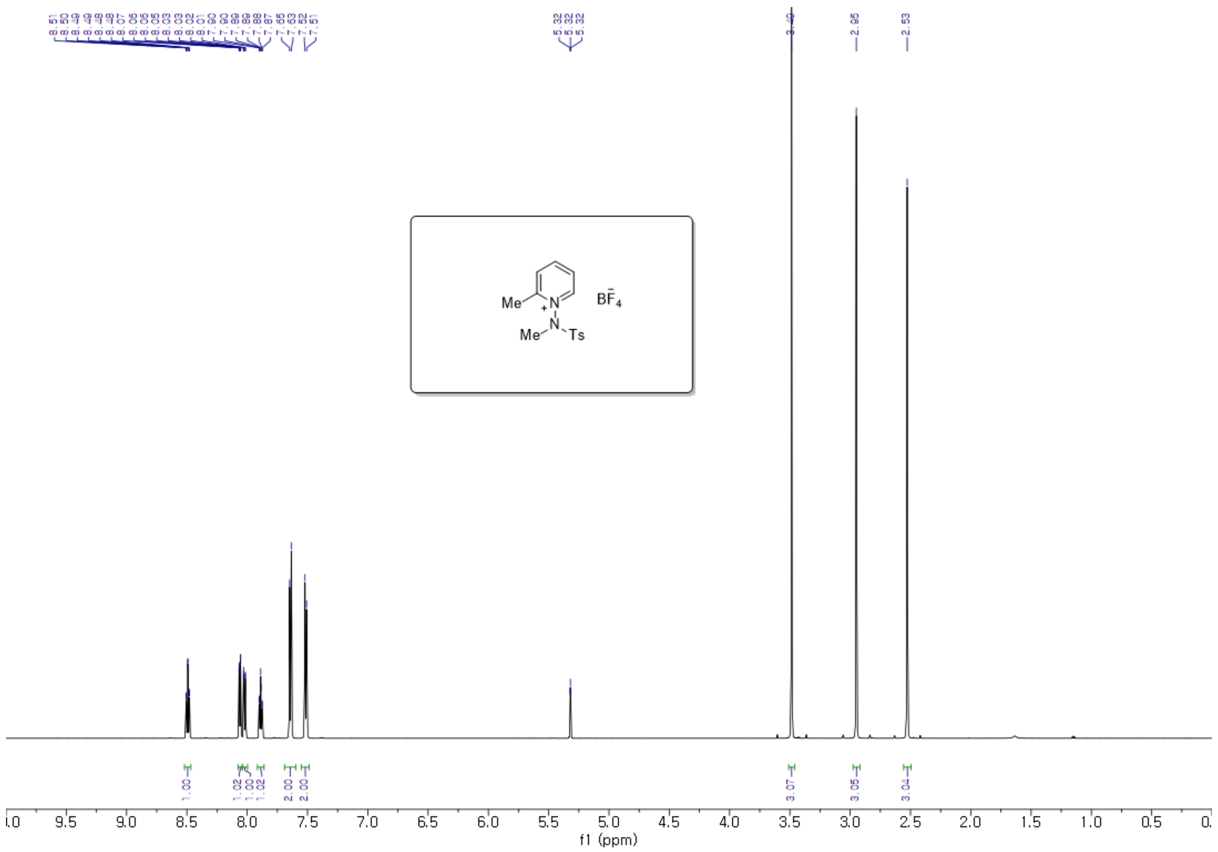

600 MHz, ^{1}H NMR in CD_2Cl_2

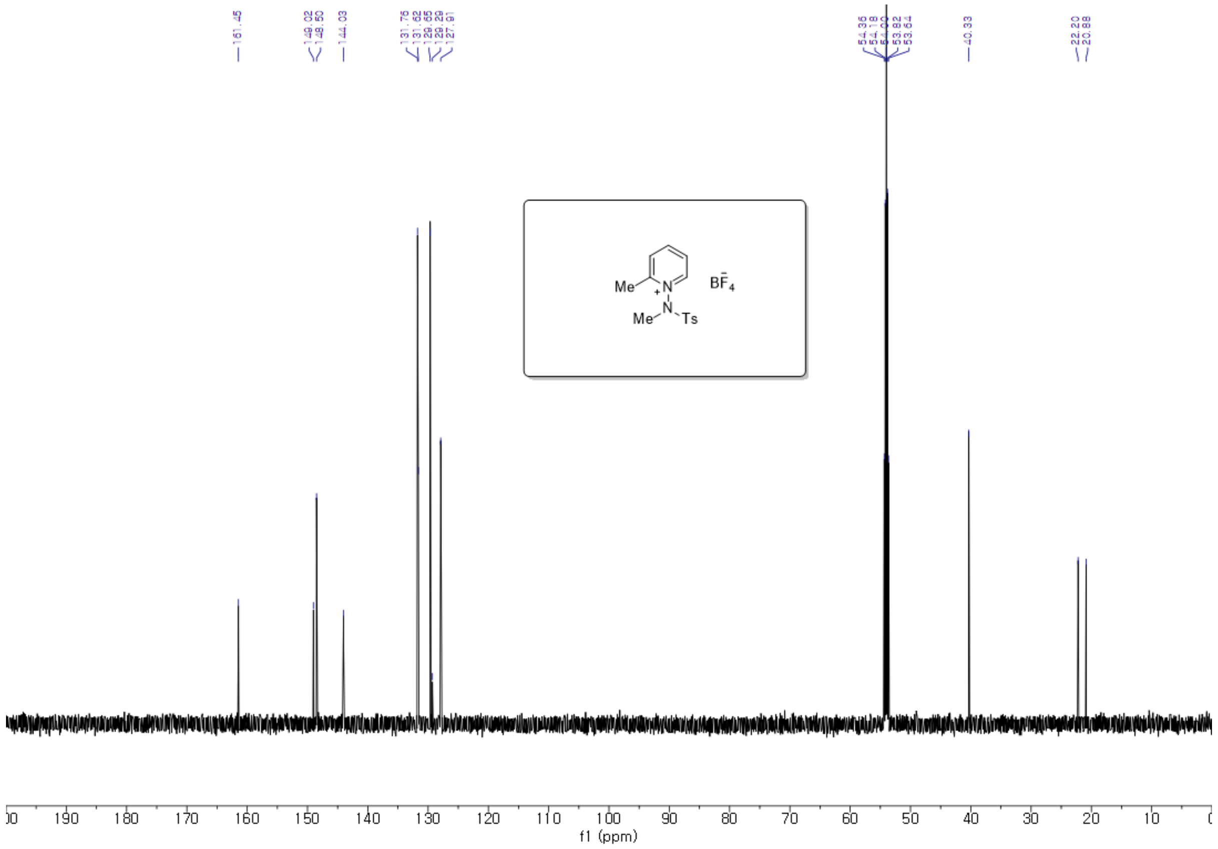

150 MHz, ^{13}C NMR in CD_2Cl_2

Supplementary Figure 67. 1H and 13C NMR of **2b**

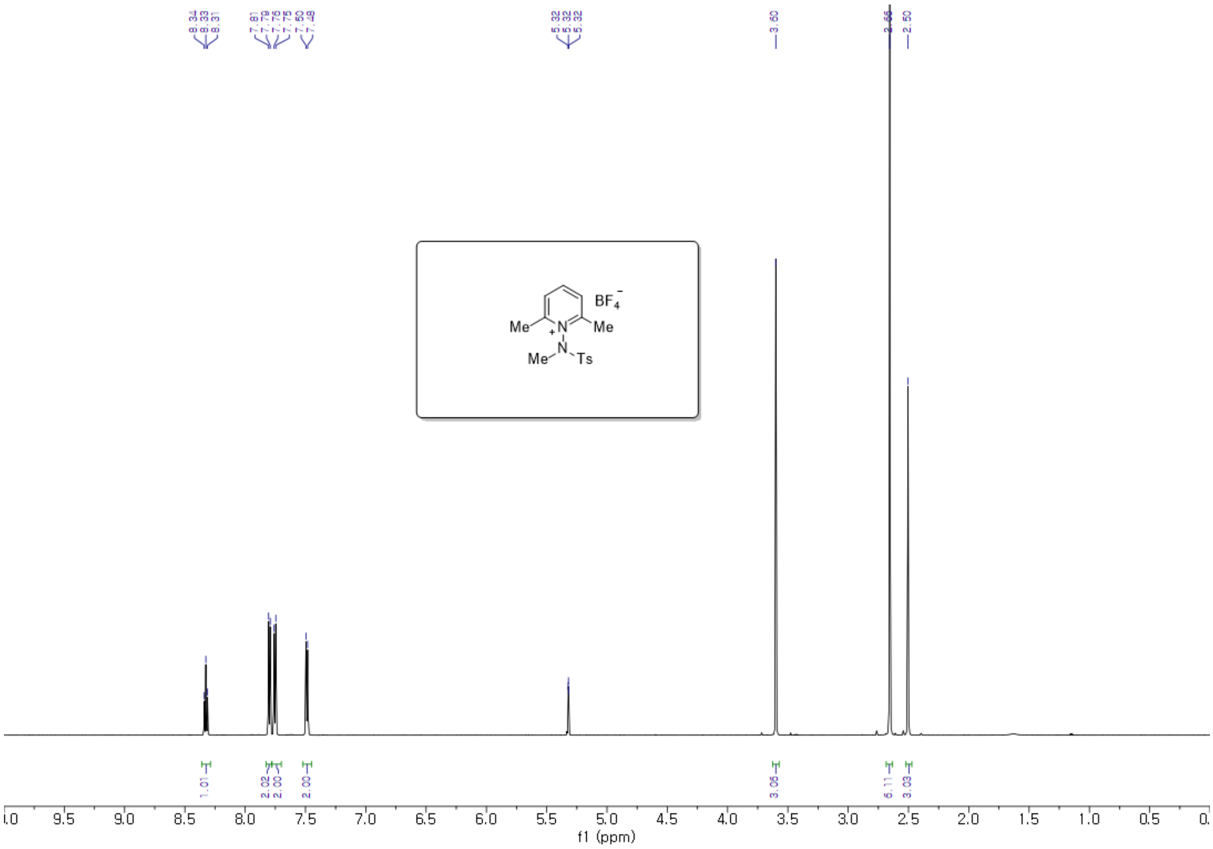

600 MHz, ¹H NMR in CD_2Cl_2

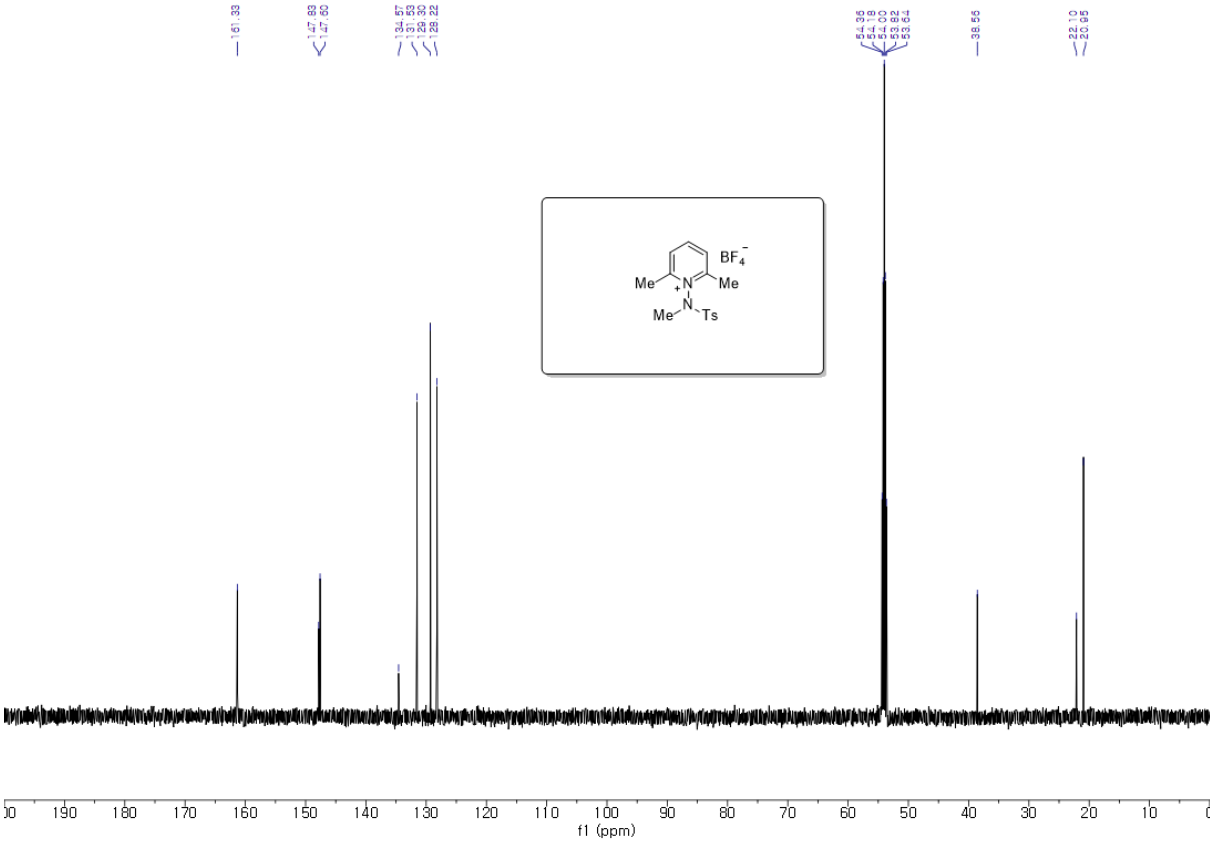

150 MHz, ¹³C NMR in CD_2Cl_2

Supplementary Figure 68. 1H and 13C NMR of **2c**

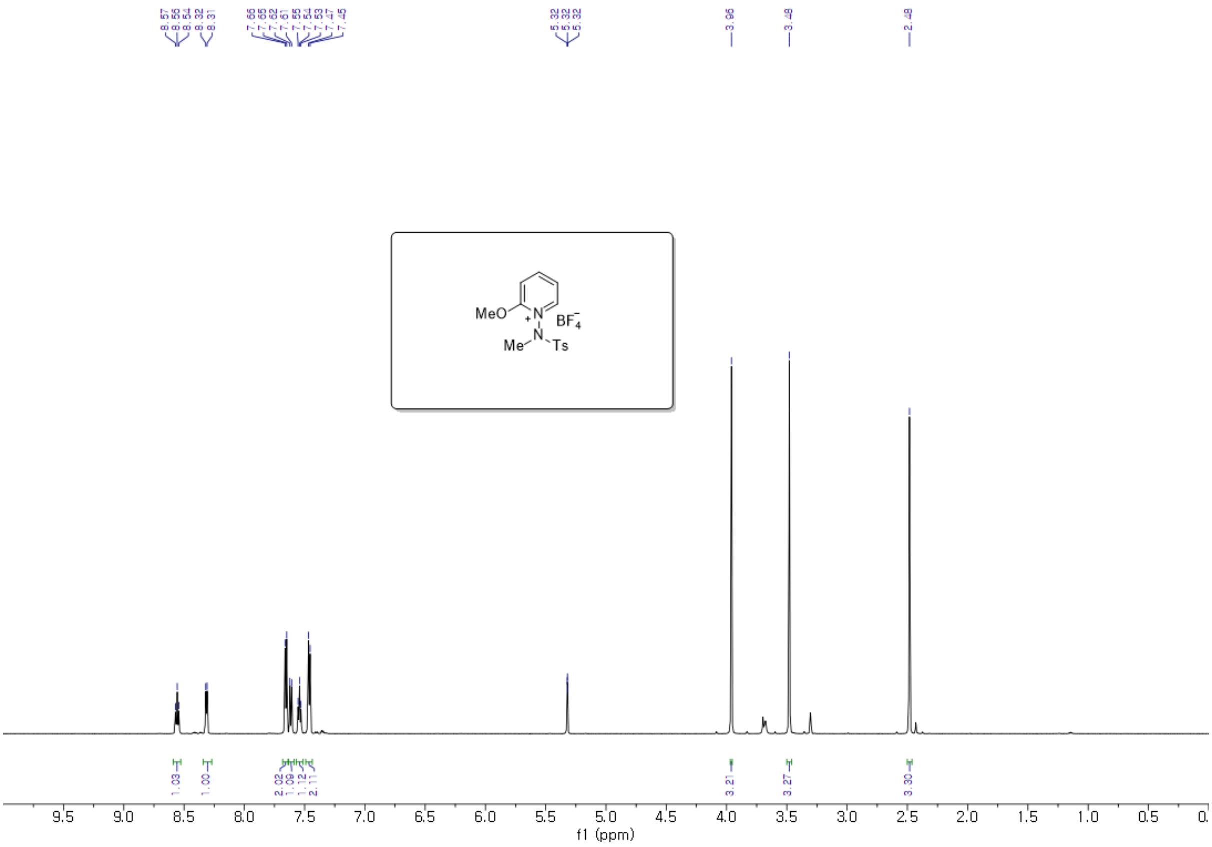

600 MHz, ^{1}H NMR in CD_2Cl_2

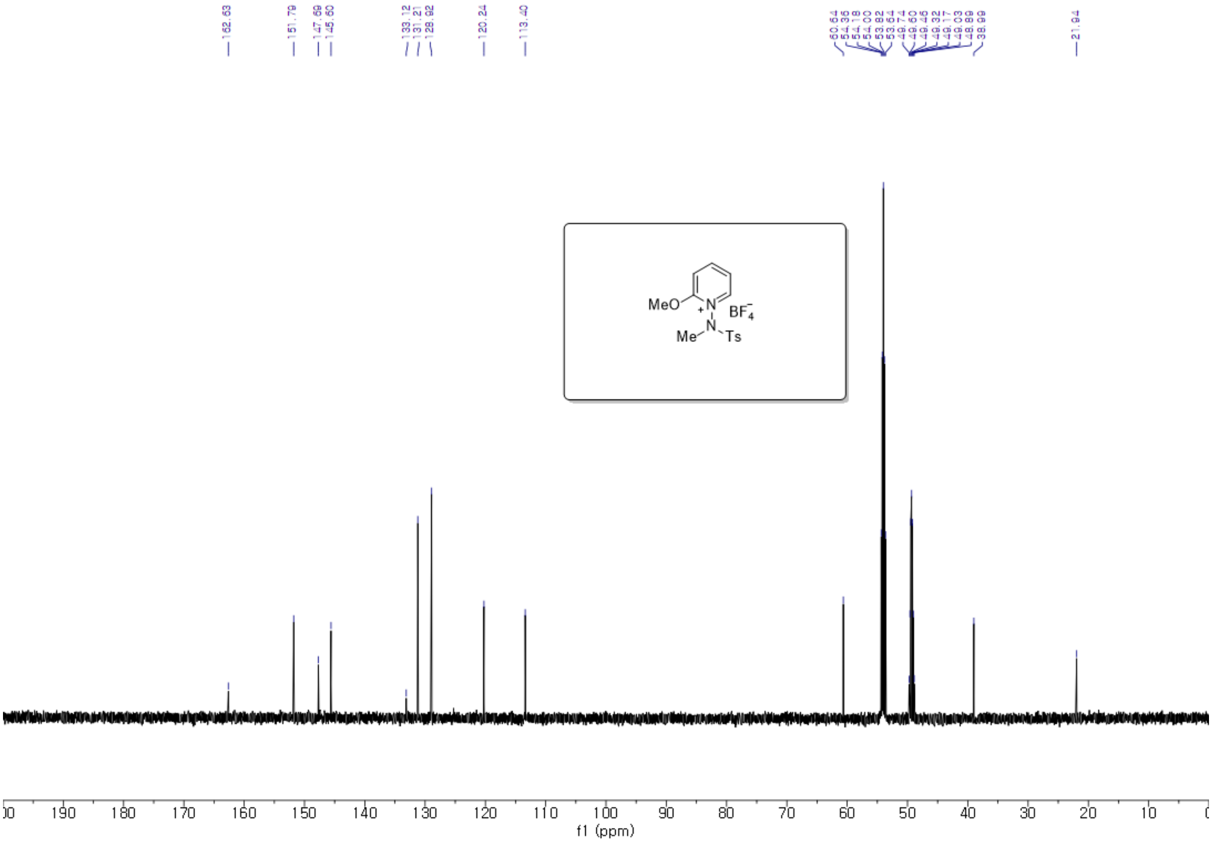

150 MHz, ^{13}C NMR in CD_2Cl_2

Supplementary Figure 69. 1H and 13C NMR of **2d**

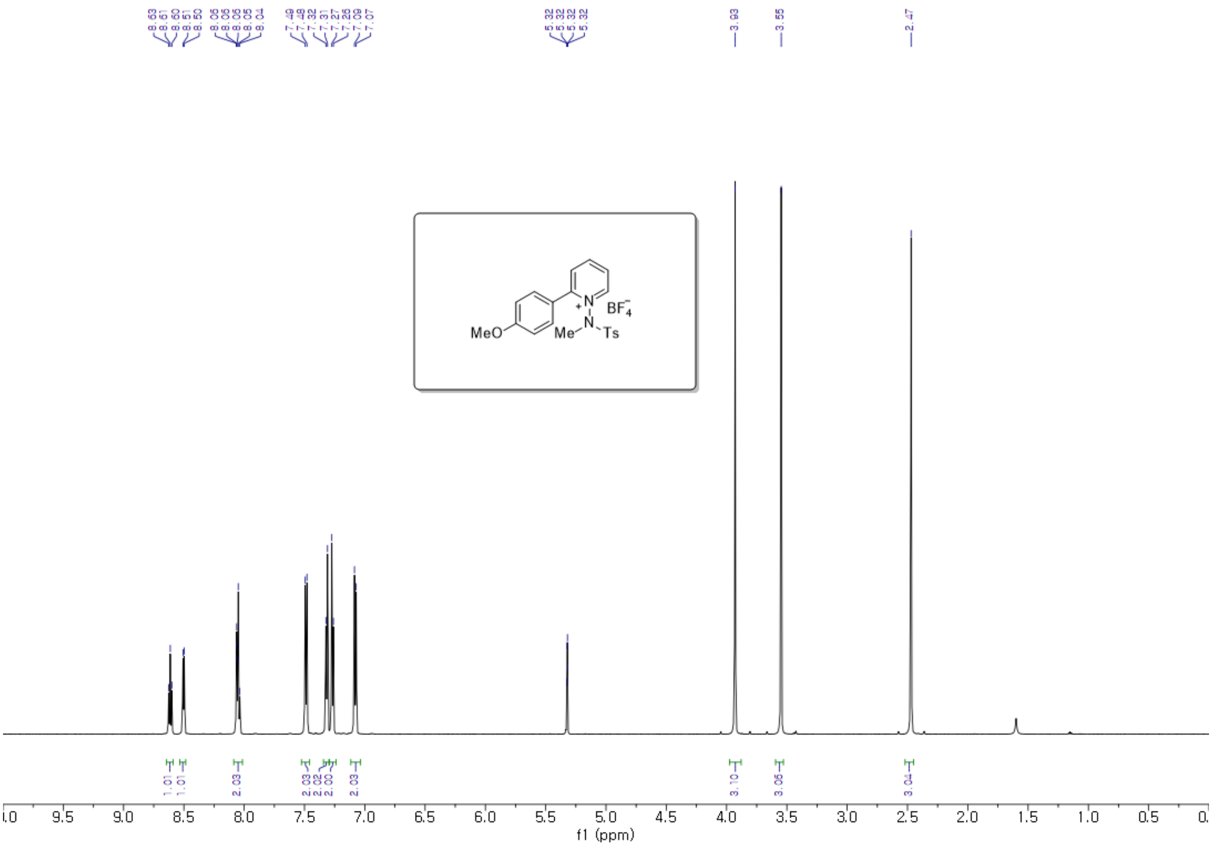

600 MHz, ^{1}H NMR in CD_2Cl_2

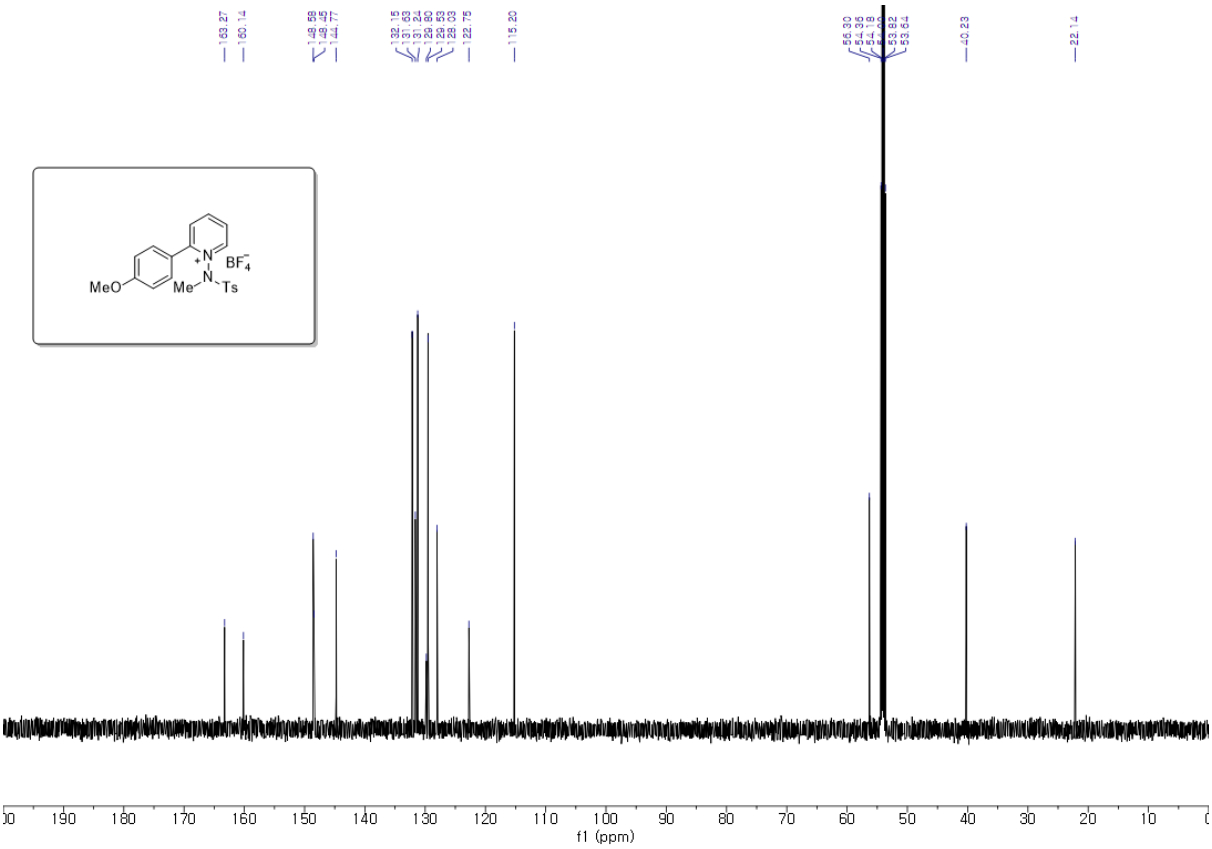

150 MHz, ^{13}C NMR in CD_2Cl_2

Supplementary Figure 70. 1H and 13C NMR of **2e**

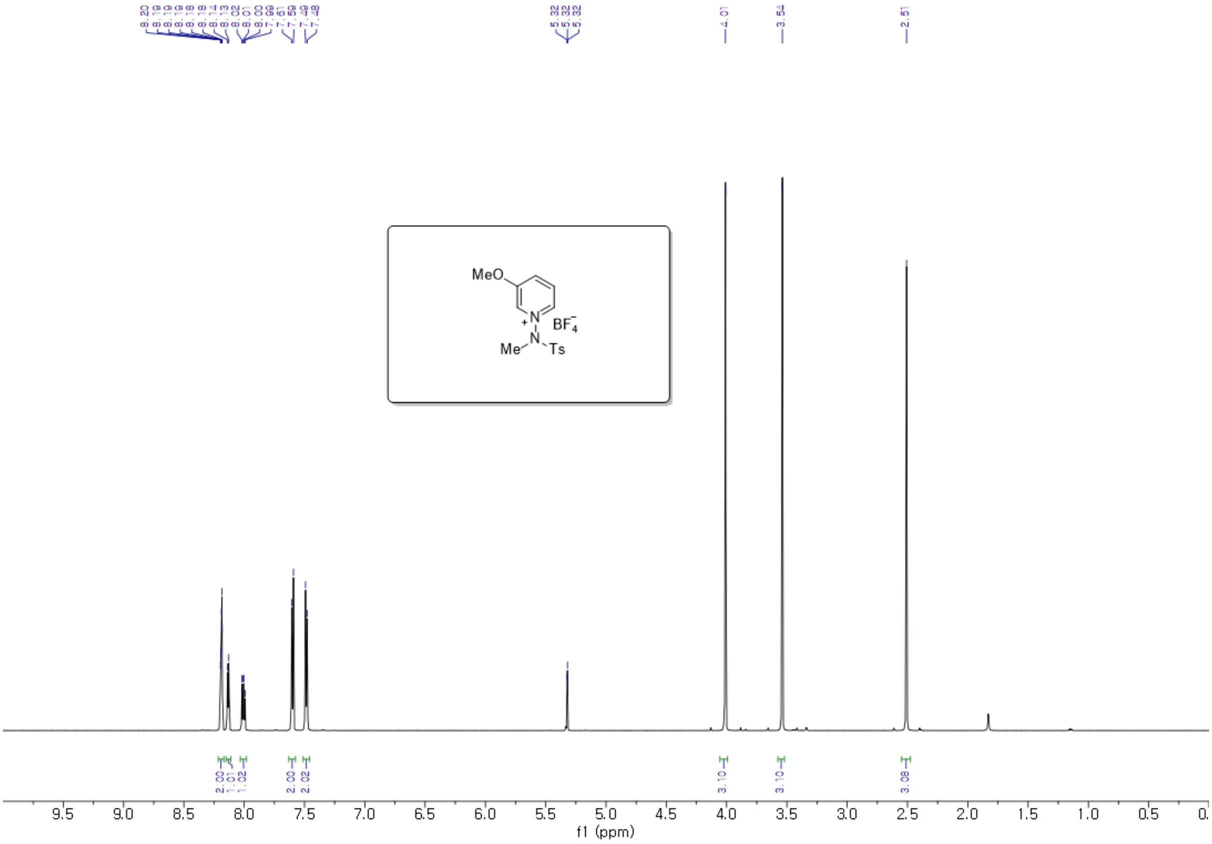

600 MHz, ^{1}H NMR in CD_2Cl_2

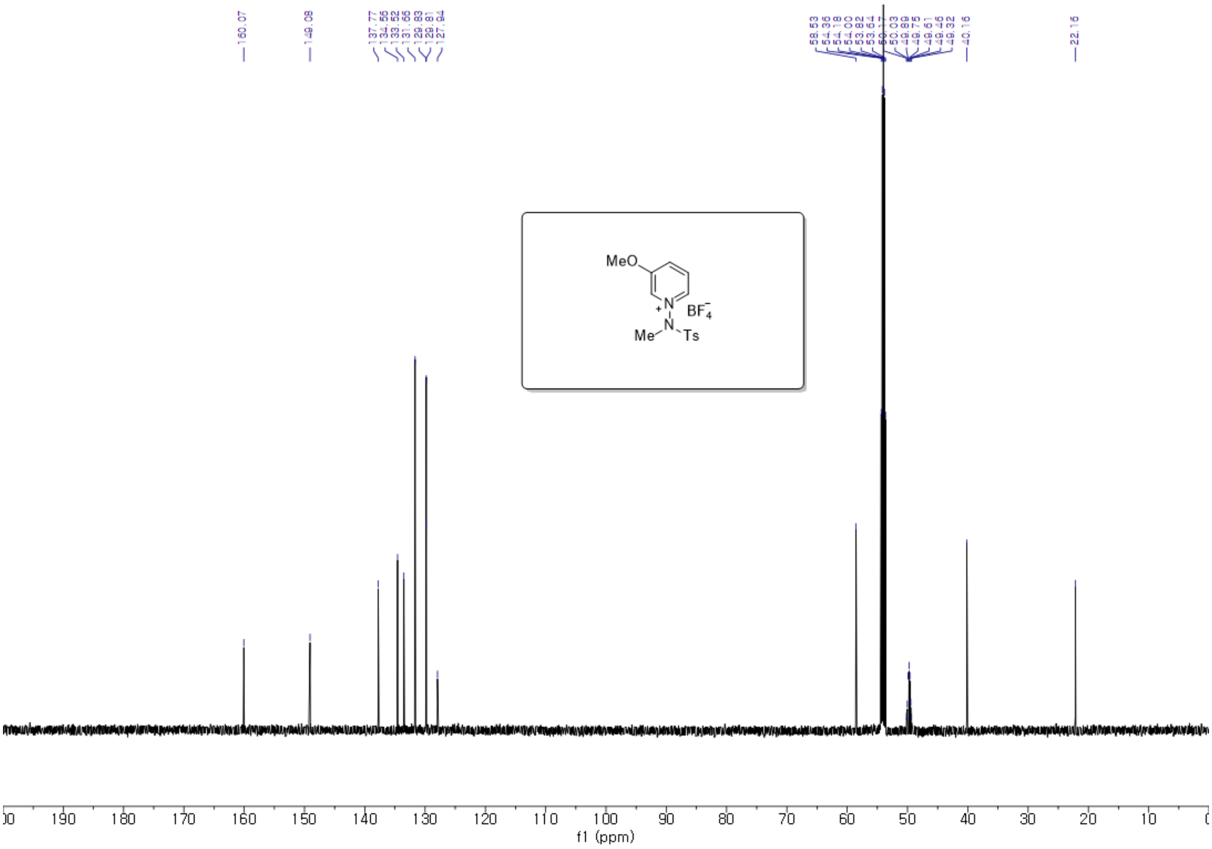

150 MHz, ^{13}C NMR in CD_2Cl_2

Supplementary Figure 71. 1H and 13C NMR of **2f**

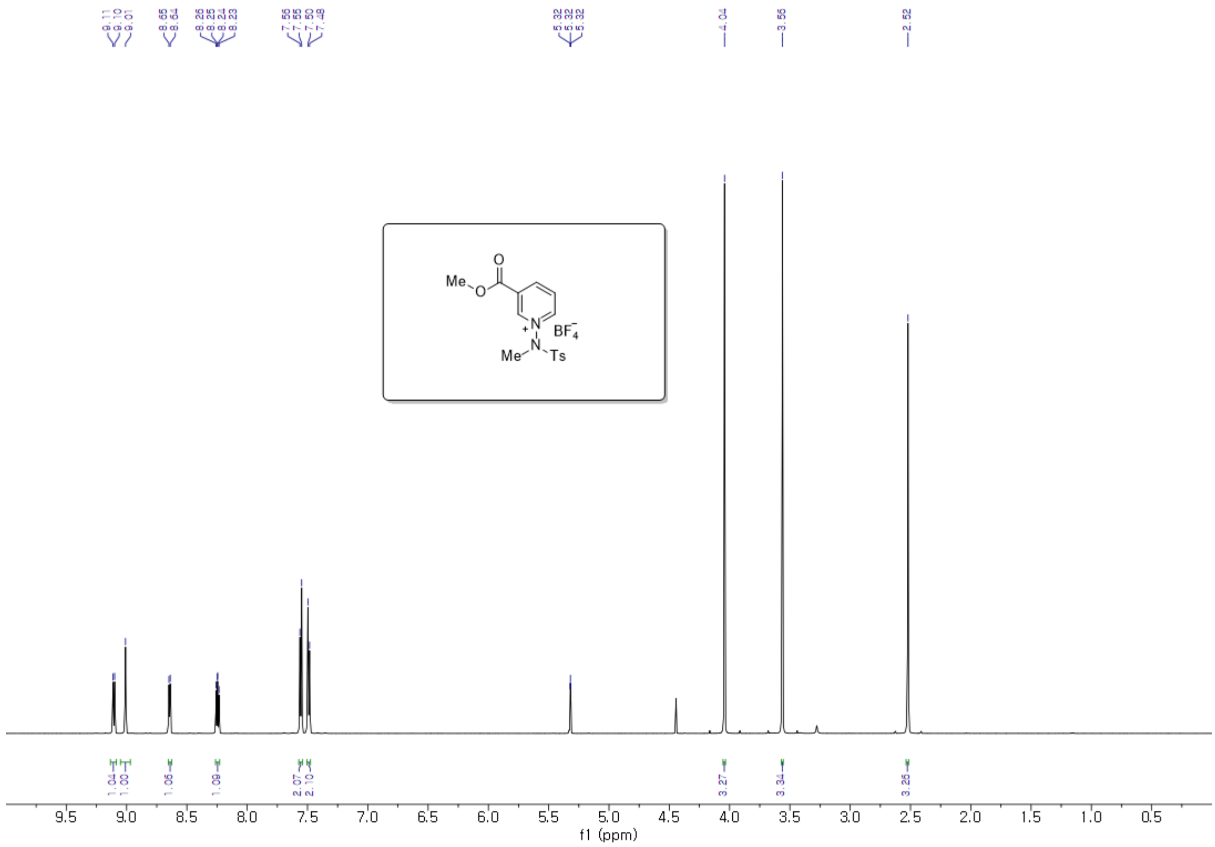

600 MHz, ^{1}H NMR in CD_2Cl_2

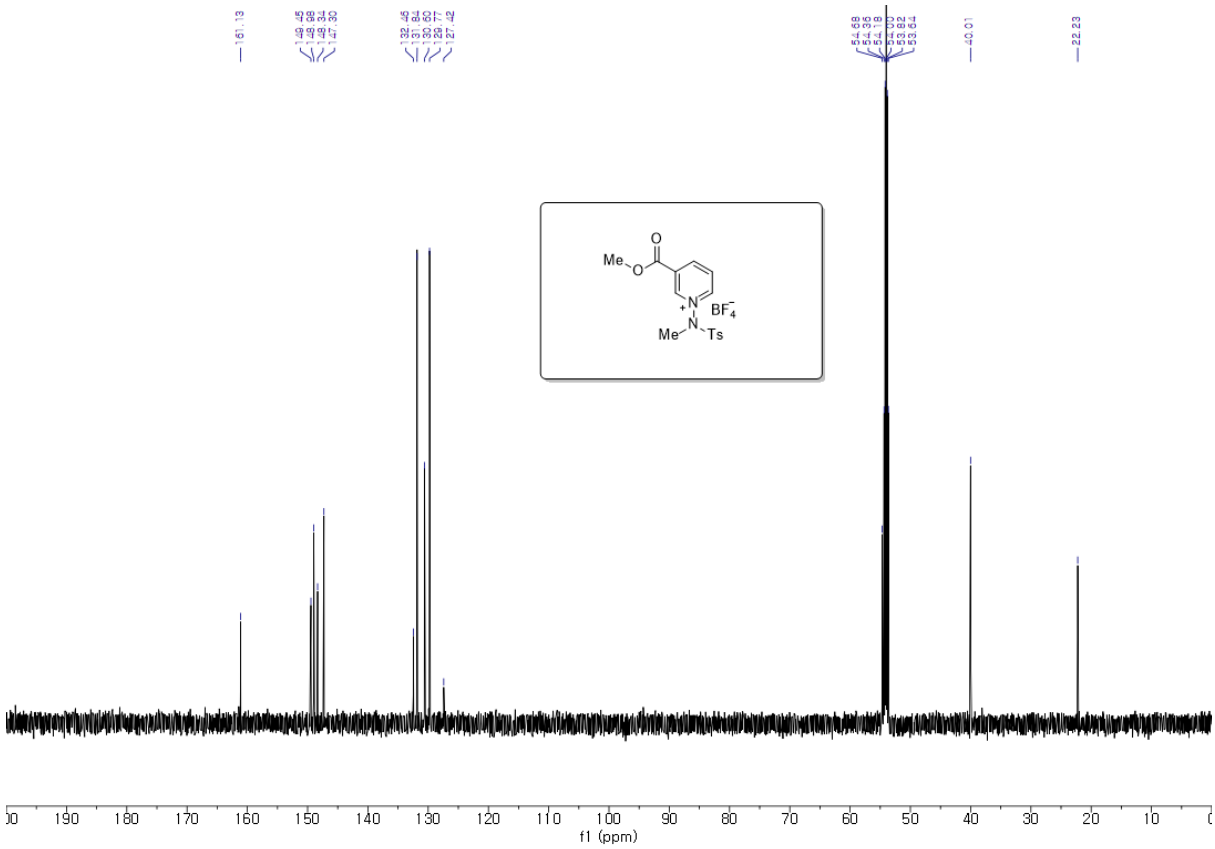

150 MHz, ^{13}C NMR in CD_2Cl_2

Supplementary Figure 72. 1H and 13C NMR of **2g**

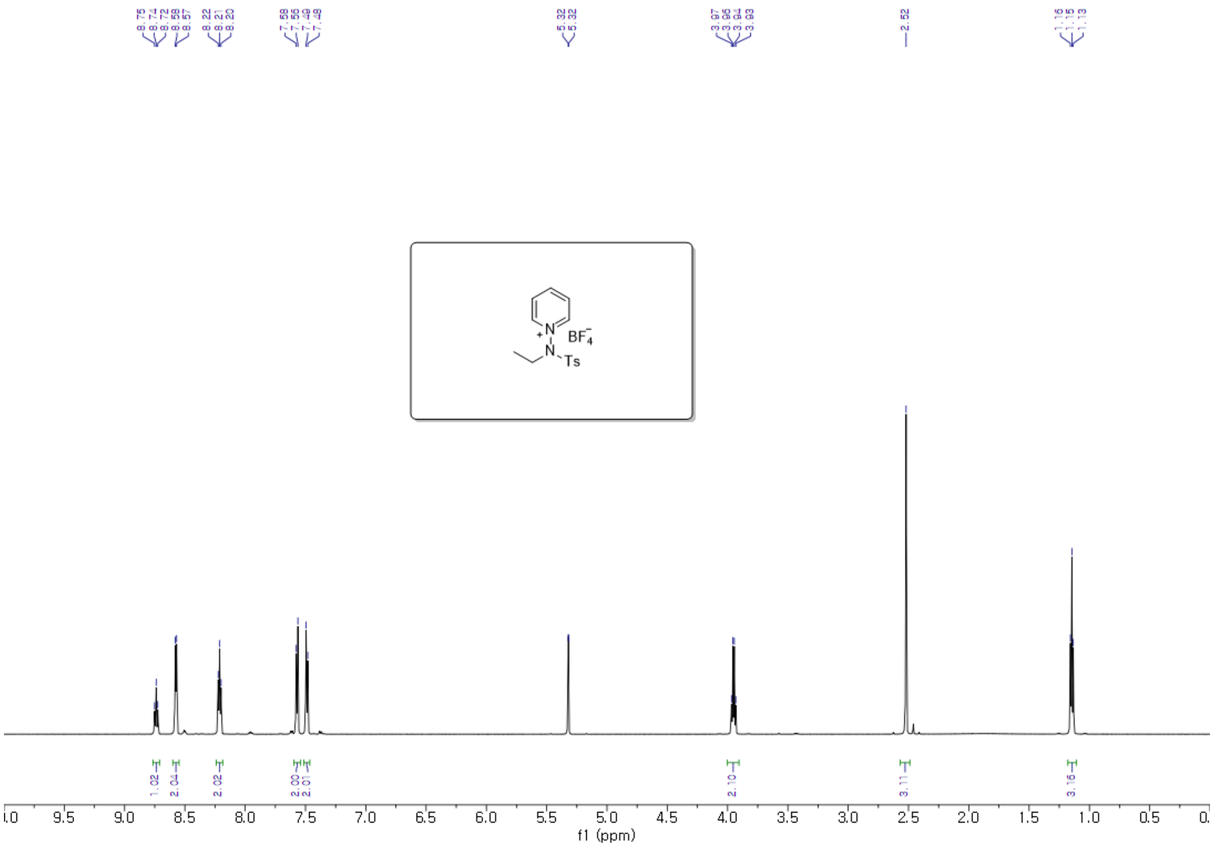

600 MHz, ¹H NMR in CD_2Cl_2

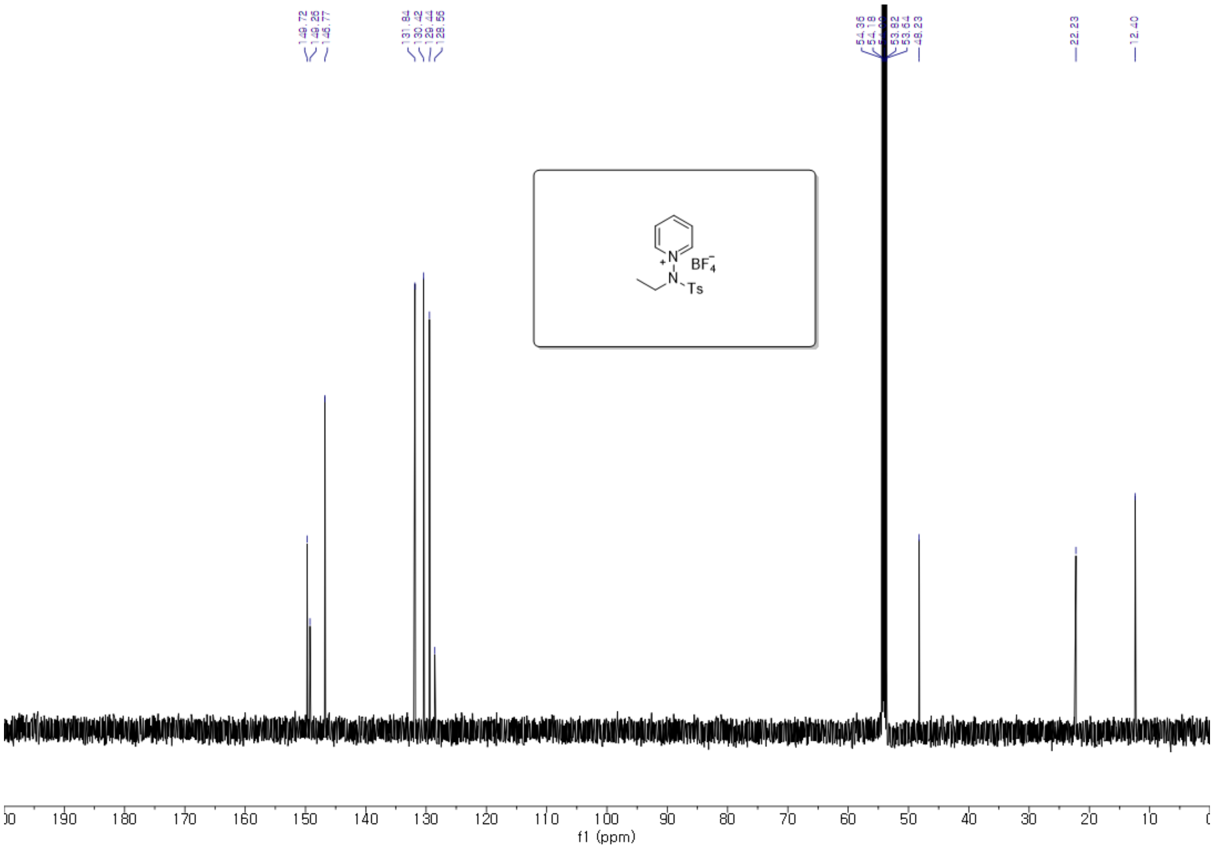

150 MHz, ¹³C NMR in CD_2Cl_2

Supplementary Figure 73. 1H and 13C NMR of **2h**

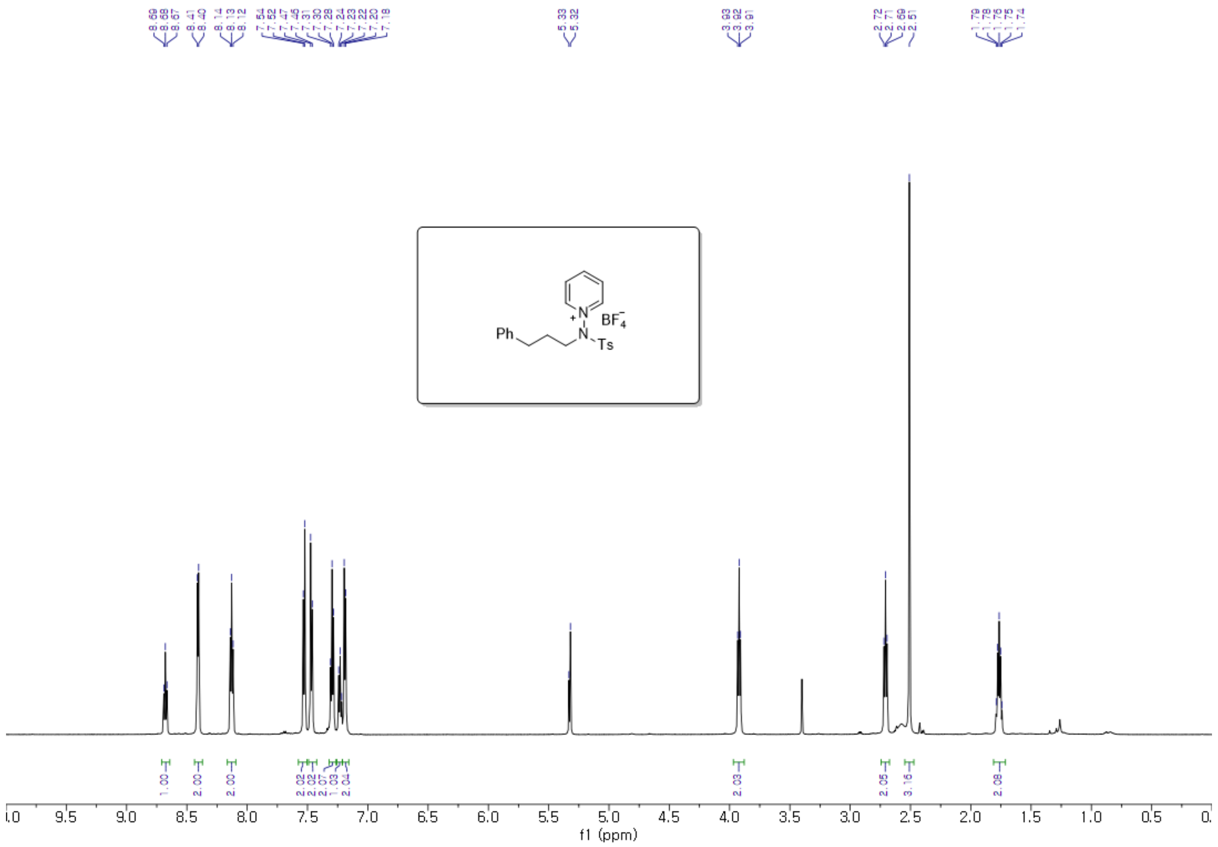

600 MHz, ^{1}H NMR in CD_2Cl_2

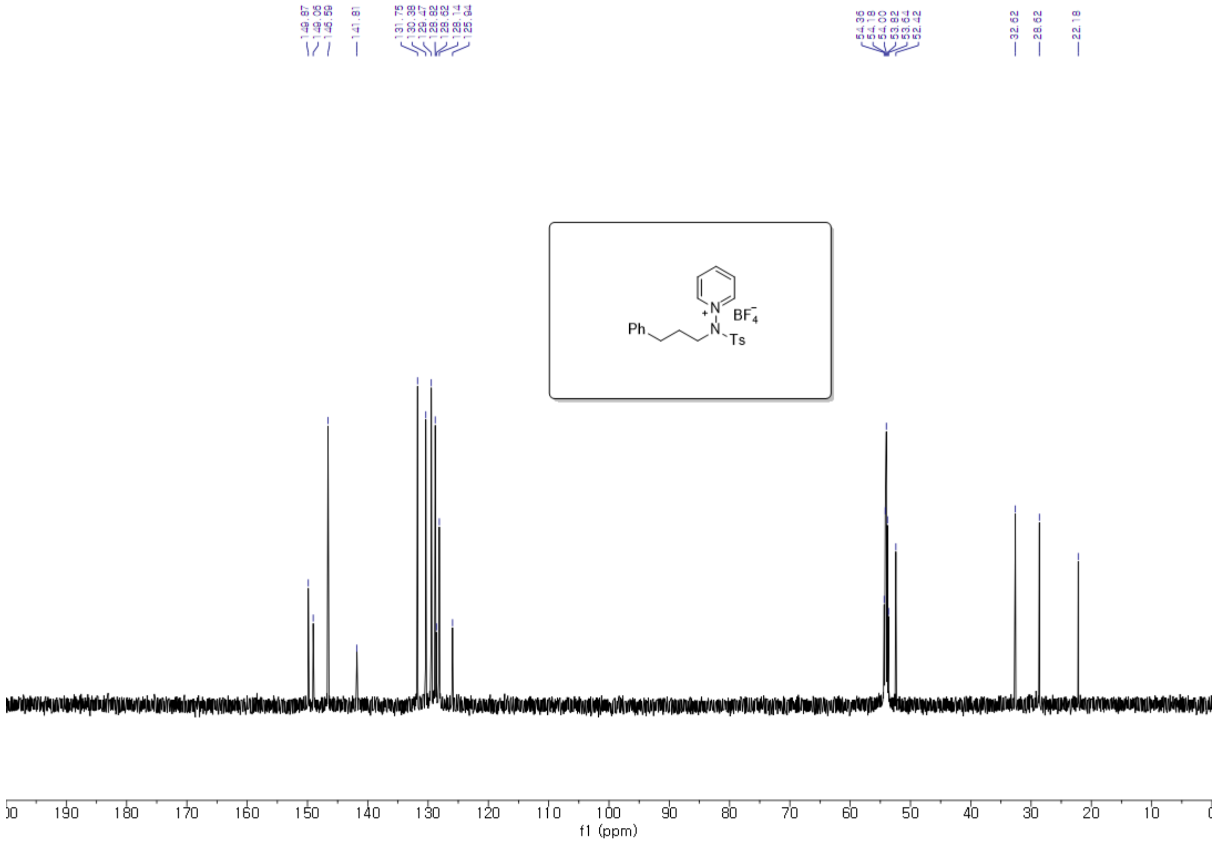

150 MHz, ^{13}C NMR in CD_2Cl_2

Supplementary Figure 74. 1H and 13C NMR of **2i**

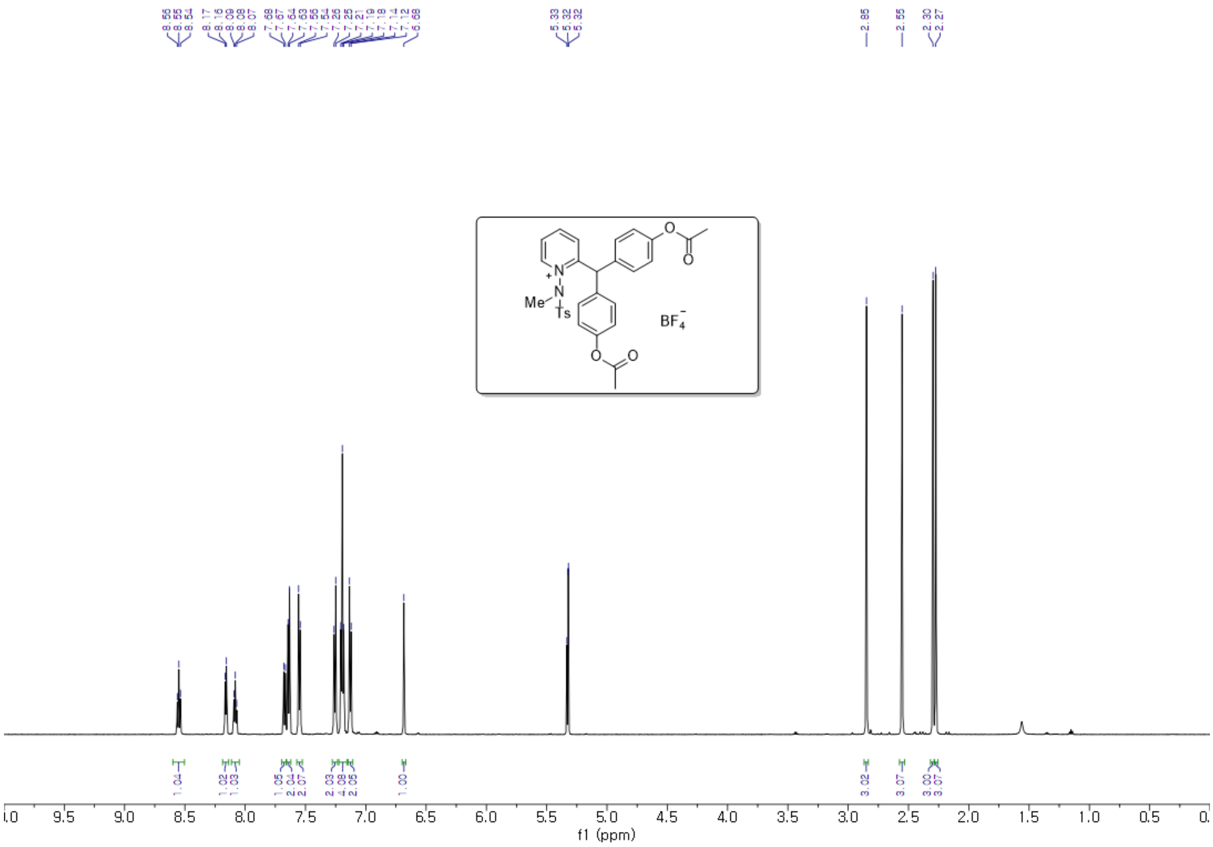

600 MHz, ^{1}H NMR in CD_2Cl_2

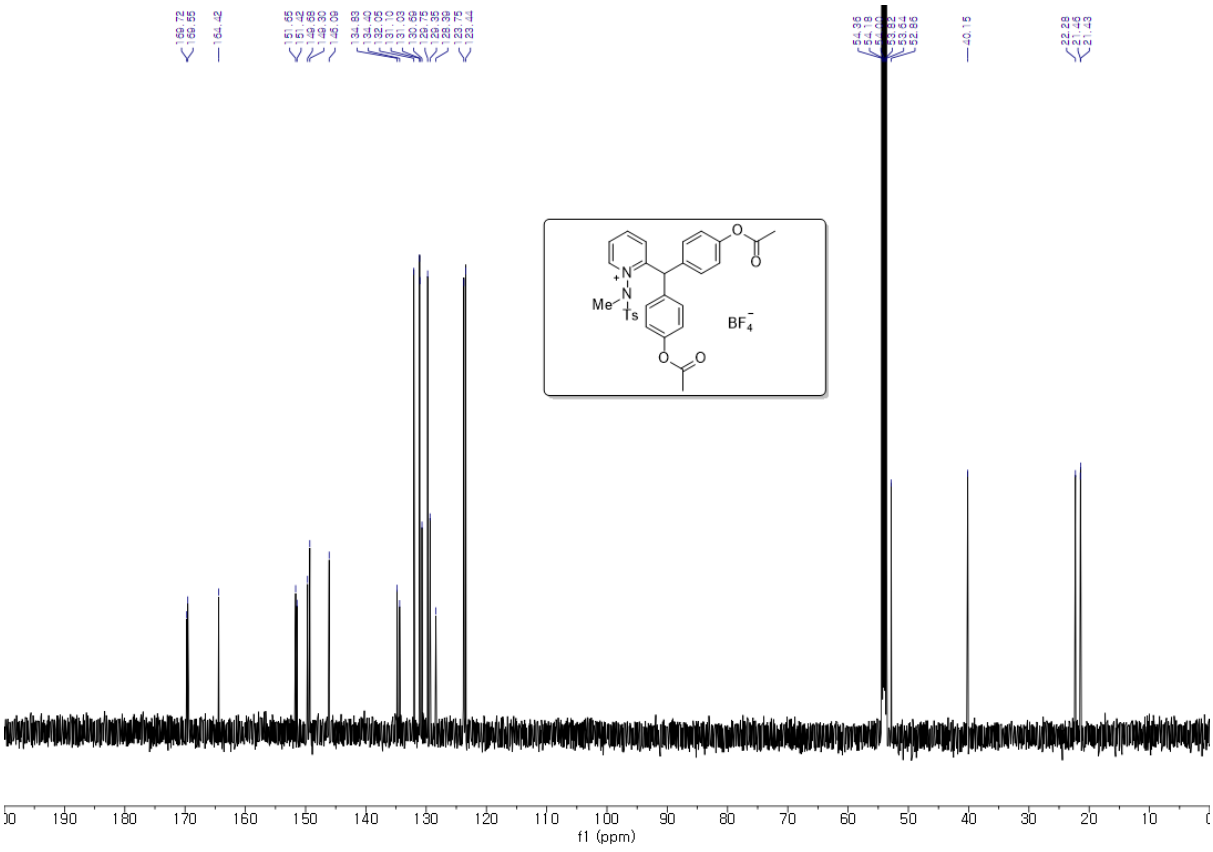

150 MHz, ^{13}C NMR in CD_2Cl_2

Supplementary Figure 75. 1H and 13C NMR of **2j**

Supplementary References

[1] Okimoto, Y., Sakaguchi, S., and Ishii, Y. (2002). Development of a Highly Efficient Catalytic Method for Synthesis of Vinyl Ethers. J. Am. Chem. Soc. 124, 1590.

[2] Klinger, D., Nilles, K., and Theato, P. (2010). Synthesis of polymeric 1-iminopyridinium ylides as photoreactive polymers. J. Polym. Sci. : Part A: Polymer Chemistry, 48, 832.

[3] Johnston, K. A., Allcock, R. W., Jiang, Z., Collier, I. D., Blakli, H., Rosair, G. M., Bailey, P. D., Morgan, K. M., Kohno, Y., and Adams, D. R. (2008). Concise routes to pyrazolo[1,5-a]pyridin-3-yl pyridazin-3-ones. Org. Biomol. Chem. 6, 175.

[4] Grayson, E. J., Bernardes, G. J. L., Chalker, J. M., Boutureira, O., Koeppe, J. R., and Davis, B. G. (2011). A Coordinated Synthesis and Conjugation Strategy for the Preparation of Homogeneous Glycoconjugate Vaccine Candidates. Angew. Chem. Int. Ed. 50, 4127.

[5] Cismesia, M. A., and Yoon, T. P. (2015). Characterizing chain processes in visible light photoredox catalysis. Chem. Sci. 6, 5426.

[6] Kuhn, H. J., Braslavsky, S. E., and Schmidt, R. (2004). Chemical actinometry (IUPAC Technical Report) Pure Appl. Chem. 76, 2105.

[7] Demas, J. N., Bowman, W. D., Zalewski, E. F., and Velapoldi, R. A. (1981). Determination of the Quantum Yield of the Ferrioxalate Actinometer with Electrically Calibrated Radiometers. Phys. Chem. 85, 2766.

[8] Parr, R. G., and Yang, W. (1989). Density Functional Theory of Atoms and Molecules, Oxford University Press, New York.

[9] Bochevarov, A. D., Harder, E., Hughes, T. F., Greenwood, J. R., Braden, D. A., Philipp, D. M., Rinaldo, D., Halls, M. D., Zhang, J., Friesner, R. A. (2013). Jaguar: A high-performance quantum chemistry software program with strengths in life and materials sciences. Int. J. Quantum Chem. 113, 2110.

[10] Becke, A. D. (1993). A new mixing of Hartree-Fock and local density-functional theories. J. Chem. Phys. 98, 1372.

[11] Marten, B., Kim, K., Cortis, C., Friesner, R. A., Murphy, R. B., Ringnalda, M. N., Sitkoff, D., and Honig, B. (1996). New Model for Calculation of Solvation Free Energies: Correction of Self-Consistent Reaction Field Continuum Dielectric Theory for Short-Range Hydrogen-Bonding Effects. J. Phys. Chem. 100, 11775.

[12] Friedrichs, M., Zhou, R., Edinger, S. R., and Friesner, R. A. (1999). Poisson–Boltzmann Analytical Gradients for Molecular Modeling Calculations. J. Phys. Chem. B 103, 3057.

[13] Edinger, S. R., Cortis, C., Shenkin, P. S., and Friesner, (1997). R. A. Solvation Free Energies of Peptides: Comparison of Approximate Continuum Solvation Models with Accurate Solution of the Poisson–Boltzmann Equation. J. Phys. Chem. B 101, 1190.

[14] Rashin, A. A., and Honig, B. (1985). Reevaluation of the Born model of ion hydration. J. Phys. Chem. 89, 5588.

[S15] Neese, F. (2002) Software update: the ORCA program system, version 4.0. Wiley Interdiscip. Rev.: Comput. Mol. Sci. 2, 73.

[16] Zhao, Y., and Truhlar, D. G. (2005). Design of Density Functionals That Are Broadly Accurate for Thermochemistry, Thermochemical Kinetics, and Nonbonded Interactions. J. Phys. Chem. A 109, 5656.

[17] Weigend, F. (2006). Accurate Coulomb-fitting basis sets for H to Rn. Phys. Chem. Chem. Phys. 8, 1057.

[18] Weigend, F. (2005). Ahlrichs, R. Balanced basis sets of split valence, triple zeta valence and quadruple zeta valence quality for H to Rn: Design and assessment of accuracy. Phys. Chem. Chem. Phys. 7, 3297.

[19] Breneman, C. M., and Wiberg, K. B. (1990). Determining Atom-Centered Monopoles from Molecular Electrostatic Potentials. The Need of High Sampling Density in Formamide Conformational Analysis. J. Comput. Chem. 11, 361.

[20] Bickelhaupt, F. M., and Baerends, E. J. (2000). Kohn-Sham Density Functional Theory: Predicting and Understanding Chemistry. Reviews in Computational Chemistry, Lipkowitz, K. B., Boyd, D. B., Eds., Wiley-VCH: New York, 15, 1.

[21] te Velde, G., Bickelhaupt, F. M., Baerends, E. J., Fonseca Guerra, C., Van Gisbergen, S. J. A., Snijders, J. G., and Ziegle, (2001). T. Chemistry with ADF. J. Comp. Chem. 22, 931.

[22] Guerra, C. F., Snijders, J. G., te Velde, G, and Baerends, E. J. (1998). Towards an order-N DFT method. Theor. Chem. Acc. 99, 391.
