# Supplementary material for: Visible light induced alkene aminopyridylation using N-aminopyridinium salts as bifunctional reagents
Source: Nat Commun. 2019 Sep 11;10:4117. doi: 10.1038/s41467-019-12216-3 (PMC6739411; doi:10.1038/s41467-019-12216-3)
Supplement: Supplementary file 2 — Description of Additional Supplementary Files [file 41467_2019_12216_MOESM2_ESM.pdf]

Description of Additional Supplementary Files

File Name: Supplementary Data 1
Description: Computed energy components for optimized structures

File Name: Supplementary Data 2
Description: Cartesian coordinates of the optimized geometries

File Name: Supplementary Data 3
Description: Vibrational frequencies (in cm-1) of the optimized structures
