# Supplementary material for: Visible light induced alkene aminopyridylation using N-aminopyridinium salts as bifunctional reagents
Source: Nat Commun. 2019 Sep 11;10:4117. doi: 10.1038/s41467-019-12216-3 (PMC6739411; doi:10.1038/s41467-019-12216-3)
Supplement: Supplementary file 3 — Supplementary Data 1 [file 41467_2019_12216_MOESM3_ESM.pdf]

Supplementary Data 1. Computed energy components for optimized structures

	E(SCF)/(eV)	ZPE/(kcal/mol)	S(gas)/(cal/mol ·K)	G(solv)/(kcal/mol)
	PW6B95-D3/def2-QZVP	BHandHLYP/6-311G**	BHandHLYP/6-311G**	BHandHLYP/6-311G**
**2a**	–31671.683	168.907	126.80	–55.55
**1b**	–6335.310	71.615	75.26	–4.79
**PO_4^{3-}**	–17490.280	9.212	68.88	–556.79
**HPO_4^{2-}**	–17516.302	16.733	71.16	–255.66
**Pyridine**	–6767.777	56.723	66.94	–6.73
**2a**	–31671.683	168.907	126.80	–55.55
**2a***	–31676.598	165.978	133.02	–16.43
**2a*-TS**	–31676.274	165.109	129.98	–16.17
**A**	–24909.265	107.701	106.05	–14.25
**A-TS**	–31244.914	181.357	146.01	–15.28
**B**	–31245.684	183.502	146.04	–15.51
***p*-B-TS**	–62917.980	353.495	231.16	–54.49 (ε=46.48)
				–48.15 (ε=9.08)
***p*-C**	–62917.874	354.323	230.23	–62.96
**D**	–62907.595	345.718	228.95	–29.69
**D-TS**	–62907.476	345.259	232.13	–27.87
**3b**	–37998.758	236.253	164.96	–21.29
***p*-B-TS'**	–62917.560	353.216	235.72	–63.98 (ε=46.48)
				–56.28 (ε=9.08)
***o*-B-TS**	–62917.864	353.377	229.72	–56.23 (ε=46.48)
				–49.33 (ε=9.08)
***p*-B-TS** [**B**]	–31245.479			

***p*-B-TS** [**2a**]	–31671.603			
***o*-B-TS** [**B**]	–31245.493			
***o*-B-TS** [**2a**]	–31671.601			
