# Supplementary material for: Visible light induced alkene aminopyridylation using N-aminopyridinium salts as bifunctional reagents
Source: Nat Commun. 2019 Sep 11;10:4117. doi: 10.1038/s41467-019-12216-3 (PMC6739411; doi:10.1038/s41467-019-12216-3)
Supplement: Supplementary file 4 — Supplementary Data 2 [file 41467_2019_12216_MOESM4_ESM.pdf]

Supplementary Data 2. Cartesian coordinates of the optimized geometries

The cartesian coordinates of optimized geometries are given below in the standard XYZ format, and units are in Å

==============================

2a

==============================

C	-3.238234282	2.353718758	-2.399677277
C	-3.616653919	1.837414622	-1.186119318
N	-4.905610561	1.853019714	-0.825371683
C	-5.871739864	2.328366518	-1.630365372
C	-5.539466858	2.858640909	-2.850185633
C	-4.208284855	2.875728846	-3.240129709
N	-5.211015701	1.256165743	0.401322573
C	-5.939377308	2.134108067	1.309777737
C	-5.824773312	-1.072486401	1.626095057
C	-6.947949409	-1.185238004	2.435585499
C	-6.824119091	-1.839743137	3.642549753
C	-5.604196072	-2.382641315	4.046051502
C	-4.500622272	-2.258298397	3.205343008
C	-4.599786282	-1.610068202	1.990988374
C	-5.504498005	-3.112030983	5.346496105
S	-5.973864555	-0.279928148	0.087472007
O	-7.356118679	0.001600291	-0.191427723
O	-5.114068508	-0.854086876	-0.902779698
H	-2.190780163	2.339692831	-2.679698706
H	-2.940809011	1.402765989	-0.459268004
H	-6.890534401	2.235874653	-1.268430471
H	-6.325387001	3.235021591	-3.495765924
H	-3.928914547	3.286869764	-4.206219673
H	-5.364959240	3.055113316	1.437422395
H	-6.957653999	2.360611439	0.970104396
H	-5.993395329	1.628726602	2.276880264
H	-7.900571823	-0.780256510	2.105844498
H	-7.694345474	-1.942790151	4.286246300
H	-3.549366951	-2.689027786	3.505520105
H	-3.745268106	-1.539611936	1.324178934
H	-6.035304070	-2.521192312	6.176560879
H	-6.025702953	-4.132892609	5.251307487
H	-4.415789604	-3.276464701	5.630959511

==============================

1b

==============================

O	-6.270089149	3.659555197	-0.277650744
C	-6.359457970	2.431304932	0.408357382
H	-5.526432037	1.771080494	0.105461396
H	-7.306400776	1.923856854	0.150100052
C	-6.297122478	2.725144386	1.883926272
H	-7.126342773	3.383729935	2.178579092
H	-5.352324486	3.228348970	2.133560896
H	-6.364945889	1.795050025	2.461691856
C	-6.227948666	3.585955858	-1.617879391
H	-6.148918152	2.563967705	-2.036489725
C	-6.263458729	4.665998459	-2.391380310
H	-6.341561317	5.678010941	-1.965487719
H	-6.207339287	4.545993328	-3.470139742

==============================

PO_4^{3-}

==============================

P	-1.300442219	-0.000504146	-0.348098963
O	-2.584758043	0.021658769	0.558772385
O	-1.281712413	1.284660697	-1.253638864
O	-0.018052476	-0.019379083	0.561126351
O	-1.321100116	-1.285964131	-1.254795432

==============================

HPO_4^{2-}

==============================

P	-1.201405287	0.015607816	-0.413150281
O	-2.544663906	-0.008364116	0.671998620
O	-1.410439372	1.248905063	-1.270921230
O	0.024542592	0.047877491	0.478944212
O	-1.459967971	-1.320400596	-1.116970778
H	-2.895731688	-0.850919306	0.381699711

==============================

Pyridine

==============================

C	-1.356752992	-0.000000942	-1.997717023
C	-1.356752872	1.189448953	-1.290419340
C	-1.356752872	1.132487535	0.093765229
N	-1.356754303	-0.000000133	0.779927909
C	-1.356753588	-1.132486105	0.093763813
C	-1.356752276	-1.189449191	-1.290418267
H	-1.356753945	-0.000001296	-3.084689856
H	-1.356753230	2.149493456	-1.797906518
H	-1.356750846	2.049374819	0.681577027
H	-1.356753230	-2.049373150	0.681575179
H	-1.356750846	-2.149494886	-1.797903657

==============================

2a

==============================

C	-3.238234282	2.353718758	-2.399677277
C	-3.616653919	1.837414622	-1.186119318
N	-4.905610561	1.853019714	-0.825371683
C	-5.871739864	2.328366518	-1.630365372
C	-5.539466858	2.858640909	-2.850185633
C	-4.208284855	2.875728846	-3.240129709
N	-5.211015701	1.256165743	0.401322573
C	-5.939377308	2.134108067	1.309777737
C	-5.824773312	-1.072486401	1.626095057
C	-6.947949409	-1.185238004	2.435585499
C	-6.824119091	-1.839743137	3.642549753
C	-5.604196072	-2.382641315	4.046051502
C	-4.500622272	-2.258298397	3.205343008
C	-4.599786282	-1.610068202	1.990988374
C	-5.504498005	-3.112030983	5.346496105
S	-5.973864555	-0.279928148	0.087472007
O	-7.356118679	0.001600291	-0.191427723
O	-5.114068508	-0.854086876	-0.902779698
H	-2.190780163	2.339692831	-2.679698706
H	-2.940809011	1.402765989	-0.459268004
H	-6.890534401	2.235874653	-1.268430471
H	-6.325387001	3.235021591	-3.495765924
H	-3.928914547	3.286869764	-4.206219673
H	-5.364959240	3.055113316	1.437422395
H	-6.957653999	2.360611439	0.970104396
H	-5.993395329	1.628726602	2.276880264
H	-7.900571823	-0.780256510	2.105844498
H	-7.694345474	-1.942790151	4.286246300
H	-3.549366951	-2.689027786	3.505520105
H	-3.745268106	-1.539611936	1.324178934
H	-6.035304070	-2.521192312	6.176560879
H	-6.025702953	-4.132892609	5.251307487
H	-4.415789604	-3.276464701	5.630959511

==============================

2a*

==============================

C	-3.056315422	2.308579206	-2.231616497
C	-3.534279346	1.778759480	-1.083031654
N	-4.896375656	1.808039308	-0.815700352
C	-5.784711838	2.320905685	-1.762111545
C	-5.305957317	2.848734140	-2.915191174
C	-3.925033092	2.879295349	-3.190374851
N	-5.344161034	1.230065823	0.339160353
C	-6.010610104	2.141166449	1.256478190
C	-5.898410320	-1.058904648	1.618860364
C	-6.946030140	-1.210630894	2.511681080
C	-6.715022087	-1.864530087	3.706808329
C	-5.454730034	-2.368572474	4.016873837
C	-4.424290180	-2.206441879	3.096420527
C	-4.637265205	-1.556103230	1.895390749
C	-5.231063843	-3.094474316	5.307468891
S	-6.175103664	-0.235939994	0.087540463
O	-7.589306831	0.026107773	-0.022490939
O	-5.460174084	-0.902722716	-0.960001171
H	-1.984236360	2.278741598	-2.402451992

H	-2.931344748	1.317888498	-0.311303258
H	-6.836810112	2.235928774	-1.517652273
H	-6.022367477	3.239031315	-3.632422924
H	-3.544923544	3.302443981	-4.111878395
H	-5.326556206	2.969339371	1.454630494
H	-6.956626892	2.525160551	0.855221391
H	-6.209064484	1.617389798	2.195794582
H	-7.927898407	-0.824979484	2.251752377
H	-7.530963421	-1.991238117	4.415146828
H	-3.437641382	-2.602100849	3.323987484
H	-3.838483810	-1.443606377	1.167751908
H	-5.712857246	-2.520279884	6.177875042
H	-5.715117455	-4.136431694	5.256578922
H	-4.115430355	-3.214607239	5.505271435

==============================

2a*-TS

==============================

C	-3.265195847	1.854361534	-2.192295313
C	-3.628922224	2.021863937	-0.883050203
N	-4.908208847	2.290761471	-0.522778451
C	-5.846496105	2.397541523	-1.497833371
C	-5.537106514	2.220499754	-2.822757959
C	-4.227954865	1.937629700	-3.194869041
N	-5.343818665	1.306035042	0.816886306
C	-6.178170204	2.119371891	1.670985341
C	-5.884495258	-1.116232395	1.754834890
C	-7.001770496	-1.386034846	2.527132511
C	-6.860447884	-2.165018320	3.660607576
C	-5.619124413	-2.672388792	4.032137394
C	-4.516077518	-2.391950369	3.231352568
C	-4.639126778	-1.615902543	2.094636202
C	-5.477655888	-3.486134768	5.281703949
S	-6.043246269	-0.113698266	0.311590791
O	-7.455832958	0.066351086	0.059990045
O	-5.185767174	-0.631891072	-0.720452130
H	-2.223729849	1.659381747	-2.429156065
H	-2.926202297	1.939771652	-0.060732026
H	-6.855291843	2.622196198	-1.164959669
H	-6.324900150	2.315999031	-3.563735723
H	-3.964259624	1.785148382	-4.236144543
H	-5.627667904	3.035303354	1.896746159
H	-7.148066998	2.369673491	1.219502687
H	-6.365399361	1.586938858	2.611997366
H	-7.968166351	-0.998986125	2.216722727
H	-7.734237194	-2.391286612	4.268140316
H	-3.543712616	-2.796646118	3.501866579
H	-3.783386946	-1.407191992	1.459156990
H	-5.452706337	-2.791698217	6.198733807
H	-6.368948460	-4.199731350	5.399444580
H	-4.511169910	-4.089046001	5.252089024

==============================

A

==============================

N	-5.327513218	1.258847356	0.641536951
C	-6.128361225	2.450017929	0.656382978
C	-5.890857220	-1.093535781	1.664161086
C	-6.925561905	-1.262825131	2.570980549
C	-6.694407463	-2.002760887	3.713493586
C	-5.445771217	-2.567879915	3.961861372
C	-4.426907063	-2.378764391	3.033431292
C	-4.639366627	-1.644160986	1.882359028
C	-5.222974777	-3.383960009	5.197211742
S	-6.165129185	-0.140297174	0.215583861
O	-7.579903126	0.114254996	0.101844445
O	-5.431740284	-0.696387231	-0.886886120
H	-5.468479633	3.319391727	0.598170459
H	-6.899083138	2.475245953	-0.129343107
H	-6.667899609	2.491819382	1.625506759
H	-7.898316860	-0.828967929	2.358223438
H	-7.499566078	-2.148111343	4.430679798
H	-3.448775530	-2.816136837	3.217046738
H	-3.850144625	-1.502809286	1.149477243
H	-5.682261467	-2.861463785	6.108088493
H	-5.728636742	-4.408032894	5.083261490
H	-4.110605717	-3.540656805	5.373267174

==============================

A-TS

==============================

O	-6.290443897	3.266222239	-1.345752120
C	-6.878197670	2.337377310	-2.247512817
H	-6.118883133	2.025403500	-2.978695393
H	-7.694483757	2.841894388	-2.777887344
C	-7.367373943	1.164160967	-1.446130514
H	-8.127947807	1.480343819	-0.724611700
H	-6.542592525	0.698157370	-0.897053897
H	-7.812727928	0.414649904	-2.108965635
C	-8.180406570	5.637090206	-0.050046399
N	-7.474550247	6.485858917	-0.976441145
S	-8.031850815	6.381632805	-2.530238390
H	-7.733176231	5.761882782	0.939941883
H	-9.235945702	5.945982933	0.014499803
H	-8.155121803	4.577336311	-0.337449163
C	-9.192667007	8.840843201	-2.376552820
C	-9.417201996	7.473491192	-2.447237730
C	-10.697570801	6.954939842	-2.432369709
C	-10.274426460	9.690911293	-2.286860704
H	-8.175841331	9.221323967	-2.404338837
C	-11.578438759	9.196686745	-2.264671326
H	-10.110001564	10.765029907	-2.235078096
C	-11.773043633	7.824423313	-2.342898607
C	-12.737946510	10.138584137	-2.162373781
H	-12.784982681	7.426528931	-2.337851286
H	-10.837605476	5.880609512	-2.510128975
H	-12.689132690	10.720538139	-1.234171152
H	-12.738377571	10.852397919	-2.994573116
H	-13.691341400	9.602501869	-2.175487518
O	-7.030601978	6.962510586	-3.387409449
O	-8.540896416	5.056485176	-2.837614298
C	-5.785938740	4.360864639	-1.896798015
H	-5.734317780	4.383174419	-2.985387802
C	-5.445722580	5.434667110	-1.159034014
H	-5.436558247	5.377607346	-0.075815365
H	-4.995509624	6.289977551	-1.645182014

==============================

B

==============================

C	1.732350826	3.566847801	0.518268585
H	1.956438303	4.049649239	1.470815301
H	1.706971526	4.320882320	-0.286067814
N	2.712616444	2.528716087	0.259817511
S	4.290207863	2.967112780	0.462064028
O	5.077412128	1.786686420	0.230539531
O	4.354585648	3.696899891	1.697891235
C	4.650495052	4.107151031	-0.840058267
C	5.090097427	3.628968954	-2.064322472
C	4.436621189	5.461786747	-0.642584980
C	5.307240486	4.520252705	-3.098558187
H	5.283539772	2.567428589	-2.188070774
C	4.658982277	6.339699745	-1.687735677
H	4.121309757	5.816360950	0.334371984
C	5.093850136	5.884313583	-2.927732706
H	5.657938957	4.150655746	-4.059451103
H	4.497168064	7.404536247	-1.536378860
C	5.365090370	6.845930576	-4.043039799
H	4.718728542	7.726853371	-3.976731777
H	6.404633522	7.196091175	-4.006385803
H	5.209647179	6.378138065	-5.020350456
H	0.747384667	3.099828959	0.576076806
C	1.231666684	0.802590549	-0.642177761
H	1.237295032	0.062949650	0.160555452
C	2.418664694	1.652788401	-0.878000081
H	3.290316820	1.008230209	-1.019938111
H	2.257861137	2.245578766	-1.794242382
O	0.046039082	1.350734591	-0.962740779
C	-1.081835747	0.544024587	-0.710845053
H	-0.992462039	-0.389654130	-1.283693552
H	-1.109641314	0.281643450	0.357862562
C	-2.311434746	1.311203480	-1.112456203
H	-2.268791676	1.571600437	-2.174905777
H	-2.395210981	2.237483978	-0.534492373
H	-3.210457325	0.710851967	-0.937082410

==============================

p-B-TS

==============================

C	-2.328633547	-1.193438768	0.207031474
H	-2.071515322	-1.499874234	1.228003740
H	-3.310524940	-1.593264699	-0.057472296
N	-2.391880751	0.255440474	0.046605144
S	-3.681756973	1.036329508	0.878311276
O	-3.430764914	2.428755522	0.643639922
O	-3.734744072	0.506131411	2.214509249
C	-5.071157455	0.479293138	-0.020312993
C	-5.322299480	1.013035774	-1.275801420
C	-5.916977882	-0.452103347	0.559057653
C	-6.439009190	0.587435246	-1.963164449
H	-4.659010410	1.764036059	-1.694663644
C	-7.031971455	-0.862827361	-0.146916047
H	-5.703238487	-0.831248522	1.554274082
C	-7.308809757	-0.352866918	-1.411337972
H	-6.651317120	0.997273922	-2.947331905
H	-7.706634045	-1.590748906	0.295989275
C	-8.536977768	-0.774137020	-2.152330160
H	-8.868811607	-1.770266056	-1.845894456
H	-9.358589172	-0.074914038	-1.951215863
H	-8.369229317	-0.780877054	-3.233662367
N	-1.200940490	0.915773749	0.321504742
C	-0.669954956	0.912361979	1.565427780
C	-0.663818359	1.652037144	-0.670939505
C	0.413751572	1.682789326	1.848538518
H	-1.183575988	0.307735443	2.304708958
C	0.419058830	2.441305399	-0.441674322
H	-1.183108091	1.586413264	-1.618808270
C	1.007920623	2.470907211	0.840798497
H	0.784402370	1.698816299	2.867399931
H	0.808269083	3.051908970	-1.248595953
H	1.599839807	3.332851171	1.130613446
H	-1.583159804	-1.574466467	-0.491426319
C	3.339773178	-0.981871605	2.040757895
H	2.778273821	-1.522071123	2.806337357
H	4.162664413	-1.612498879	1.672224641
N	2.440380812	-0.581572294	0.973184049
S	1.354735613	-1.733572125	0.490305275
O	0.682001173	-1.169533491	-0.656778336
O	0.608133376	-2.095108271	1.665813208
C	2.279311895	-3.131359100	-0.032362454
C	2.788261175	-3.163126469	-1.322815537
C	2.520935535	-4.161769867	0.862179160
C	3.555970430	-4.243032455	-1.711230874
H	2.557846785	-2.363798618	-2.021360874
C	3.290695190	-5.234777451	0.453154296
H	2.086768866	-4.127928734	1.857258916
C	3.819529533	-5.292017937	-0.832893491
H	3.952986717	-4.281289101	-2.722616673
H	3.480013609	-6.051095009	1.145805836
C	4.619969845	-6.474488735	-1.277341604
H	5.138317108	-6.946046352	-0.437137514
H	3.963899136	-7.230858803	-1.726676226
H	5.363267422	-6.195192814	-2.030219316
H	3.764256954	-0.090603121	2.510659218
C	3.091088295	1.701491237	0.462866962
H	3.439716578	1.877116084	1.485021114
C	2.979182959	0.300330877	-0.035570592
H	2.325483799	0.295948386	-0.913840592
H	3.982405424	-0.029092539	-0.366547853
O	3.536514997	2.533656120	-0.466202736
C	4.133481503	3.752262115	-0.033729069
H	3.386101484	4.350186348	0.507127583
H	4.948910713	3.513422251	0.661830902
C	4.638897419	4.483637333	-1.243946910
H	3.818569660	4.716931343	-1.929826021
H	5.377682209	3.878710270	-1.778121829
H	5.113362312	5.422895908	-0.942843676

==============================

p-C

==============================

C	-3.341004133	2.529545307	-1.274532676
C	-3.875593901	1.987166166	-0.155554578
N	-5.191080093	1.665169358	-0.109347805
C	-6.020094395	1.838400602	-1.187370181
C	-5.545650959	2.358361244	-2.335055828
C	-4.135402679	2.769881010	-2.494904518
N	-5.647678375	1.062588930	1.052895904
C	-6.764258862	1.761066079	1.678581595
C	-5.903323174	-1.293123364	2.351815224
C	-7.102370262	-1.673625469	2.939368486
C	-7.069127083	-2.233364582	4.199621677
C	-5.862733364	-2.418915749	4.873349667
C	-4.677464485	-2.033737898	4.250181198
C	-4.685233089	-1.473605275	2.989064932
C	-5.851318359	-3.034554482	6.234894276
S	-5.926939487	-0.627894044	0.744486094
O	-7.249180317	-0.735095501	0.191529036
O	-4.767619610	-1.002160311	-0.008461169
H	-2.295783281	2.818535089	-1.289774179
H	-3.324764490	1.780452967	0.753879070
H	-7.038564682	1.489342809	-1.058212280
H	-6.232068539	2.442984343	-3.171477318
H	-3.678601980	2.171429634	-3.311424255
H	-6.457715034	2.788587570	1.888817549
H	-7.678370953	1.746927023	1.073063612
H	-6.965034008	1.263679504	2.630774021
H	-8.036233902	-1.549017310	2.398781776
H	-7.998023033	-2.544879198	4.670650482
H	-3.730981112	-2.188763618	4.760731220
H	-3.758314133	-1.202075362	2.492407084
H	-6.444111347	-2.436633825	6.936841011
H	-6.293207645	-4.037331104	6.209177971
H	-4.836424351	-3.119860649	6.631373882
C	-2.151085854	5.776846886	-2.609598875
O	-2.582724094	4.469439507	-2.963932753
C	-3.943495750	4.244904041	-2.988494873
H	-2.291219950	5.914438725	-1.523923635
H	-2.762864590	6.520038128	-3.131731033
C	-0.707170904	5.904718399	-3.003605127
H	-0.614904344	5.788502216	-4.087367058
H	-0.097000197	5.141441822	-2.507956505
H	-0.323455751	6.890156269	-2.719686270
N	-4.566418171	5.814601898	-4.751297474
C	-5.836054802	6.466140747	-4.506330013
H	-6.122087479	6.305513859	-3.461184025
H	-5.728878498	7.540443897	-4.663578510
H	-6.634856701	6.072856426	-5.155536175
S	-3.661820173	6.352900028	-6.037191391
C	-4.487240314	4.416882992	-4.402128696
C	-4.439534664	5.726447105	-7.486558437
O	-3.806209803	7.779992580	-6.022915363
O	-2.387937546	5.702914238	-5.903186321
C	-5.497246265	6.427970886	-8.049048424
C	-6.124662399	5.911868095	-9.164953232
H	-5.793531895	7.387419224	-7.634573936
C	-3.998952389	4.534640789	-8.037377357
C	-4.639807701	4.034360409	-9.157302856
H	-3.133857012	4.032424927	-7.613969326
C	-5.710175514	4.708404064	-9.733185768
H	-6.945217133	6.462162971	-9.619516373
C	-6.393675804	4.175957680	-10.953157425
H	-4.289027691	3.107966661	-9.605126381
H	-7.468537331	4.050827503	-10.778496742
H	-6.279959679	4.868318081	-11.795575142
H	-5.982323170	3.208671331	-11.254495621
H	-4.471463680	4.915616989	-2.289770603
H	-3.783924341	3.902860403	-5.066005230
H	-5.478891850	3.954419613	-4.523234844

==============================

D

==============================

C	-3.642536163	1.117790818	-2.746591806
C	-3.801855564	0.792400658	-1.447227955
N	-4.345690250	1.704338789	-0.552988648
C	-4.799399376	2.939184904	-1.015768170
C	-4.651351452	3.264724970	-2.324086905
C	-4.061194420	2.379357338	-3.246995687
N	-4.685328960	1.253184080	0.696514964
C	-4.044270992	1.962898612	1.790243506
C	-6.386185169	-0.259923548	2.118232489

C	-6.835075378	0.026545949	3.396203995
C	-6.881565571	-0.991018057	4.330622673
C	-6.482718468	-2.283568144	4.003671646
C	-6.042573452	-2.541295528	2.708179235
C	-5.993055344	-1.538011074	1.759972930
C	-6.509501934	-3.367271662	5.036371231
S	-6.359882355	1.013234138	0.904251099
O	-6.943747997	2.194157839	1.491061807
O	-6.823432446	0.500570893	-0.351378530
H	-3.183623075	0.396179259	-3.416326523
H	-3.519041538	-0.155998498	-1.008316040
H	-5.272944927	3.576704741	-0.278693616
H	-5.009418964	4.238766670	-2.649031878
H	-2.963780880	1.898973584	1.643440604
H	-4.354188919	3.013722897	1.851167798
H	-4.298280716	1.466104507	2.731045485
H	-7.158661842	1.035977483	3.634321928
H	-7.242151737	-0.780102074	5.335173130
H	-5.744012356	-3.550789595	2.436305523
H	-5.673292160	-1.742953777	0.742069960
H	-5.579212189	-3.292227030	5.701291084
H	-7.431239605	-3.261966467	5.703829288
H	-6.519534111	-4.387478828	4.538609505
C	-1.684029102	3.252983093	-4.802459717
O	-2.694493771	2.380574703	-5.228686333
C	-3.964696169	2.686742306	-4.709174156
H	-1.692807674	3.331542969	-3.705056667
H	-1.872461796	4.262240410	-5.207993031
C	-0.355192125	2.724890471	-5.276643753
H	-0.315399945	2.667196751	-6.370019436
H	-0.183159426	1.719932437	-4.877888203
H	0.458015621	3.377820969	-4.939622879
N	-5.076994896	2.312435627	-6.881305695
C	-5.914919853	3.455336094	-7.164270401
H	-5.733441830	4.221349716	-6.404387474
H	-5.655031204	3.886772633	-8.136240005
H	-6.981716156	3.195001364	-7.170861244
S	-4.850113392	1.262148261	-8.122423172
C	-4.972990990	1.871136308	-5.506347179
C	-3.642491817	2.094921827	-9.107922554
O	-4.271674633	0.072121263	-7.558856010
O	-6.049998760	1.182652235	-8.917605400
C	-2.433265924	2.443631887	-8.523606300
C	-1.488162875	3.092179298	-9.289157867
H	-2.268546820	2.222769022	-7.471001625
C	-3.912794113	2.382755518	-10.430185318
C	-2.948629856	3.035060167	-11.184941292
H	-4.873456001	2.100440502	-10.850962639
C	-1.730395913	3.397176027	-10.629141808
H	-0.537776053	3.378988266	-8.841993332
C	-0.689552367	4.107549667	-11.438556671
H	-3.151829958	3.268641472	-12.227382660
H	-0.448172837	5.083252907	-10.999710083
H	0.240697205	3.528217554	-11.476292610
H	-1.026249766	4.273617268	-12.466062546
H	-4.167919636	3.763393164	-4.856291294
H	-4.652439117	0.825441778	-5.495821953
H	-5.955426693	1.928863764	-5.017542839

==============================

D-TS

==============================

C	-4.140444279	0.865505576	1.336726308
H	-4.098326206	1.069662094	2.415295839
H	-5.145426273	1.121142983	0.977489531
N	-3.156047583	1.603629112	0.578638494
S	-3.225272894	3.251098394	0.771207511
O	-2.012811899	3.776303768	0.195677385
O	-3.587433338	3.605550289	2.125820398
C	-4.582374573	3.671625376	-0.273235679
C	-4.414251804	3.620569706	-1.648247957
C	-5.793269634	4.030636311	0.289498121
C	-5.481354713	3.935736895	-2.463967800
H	-3.449148417	3.344090700	-2.062804699
C	-6.853733063	4.342479229	-0.544584870
H	-5.885359764	4.075008392	1.370980263
C	-6.714397430	4.300318718	-1.926270843
H	-5.359773159	3.900481462	-3.544262171
H	-7.809904099	4.627743244	-0.111960873
C	-7.854138374	4.670121670	-2.824176311
H	-8.818919182	4.507400513	-2.333849430
H	-7.798367500	5.730643749	-3.101549387
H	-7.836610794	4.087486267	-3.750982285
N	-1.615291953	0.985901237	1.031193852
C	-1.091683626	1.525585651	2.158051968
C	-0.828834355	0.914214253	-0.074441612
C	0.167007342	2.075681210	2.174503088
H	-1.739716649	1.536779881	3.029501677
C	0.439249307	1.417690873	-0.098911591
H	-1.289696097	0.447427779	-0.938561916
C	0.956394374	2.058670998	1.032103777
H	0.529577494	2.534389496	3.091803074
H	1.035385489	1.301823616	-0.999616385
H	-3.968402624	-0.197223276	1.153666615
C	3.914348364	0.499382794	2.261585712
H	3.869442225	-0.549046576	2.560083389
H	4.938097477	0.885196149	2.398488045
N	3.465351820	0.629606962	0.892271519
S	3.937483788	-0.544750810	-0.162355959
O	3.225529909	-0.301320344	-1.387895823
O	3.837593555	-1.791177273	0.544698417
C	5.650717258	-0.244828776	-0.473364472
C	6.021677971	0.610434473	-1.496743679
C	6.603592873	-0.819046259	0.355159461
C	7.362484932	0.900386453	-1.680844665
H	5.262726784	1.023178101	-2.154846430
C	7.937159538	-0.518559515	0.157103494
H	6.292044163	-1.515528083	1.128224134
C	8.335846901	0.348000050	-0.857271791
H	7.659555912	1.567692161	-2.486333370
H	8.689472198	-0.970899582	0.799693763
C	9.787281990	0.652614176	-1.064613938
H	10.268932343	0.939566910	-0.123065479
H	10.319032669	-0.226565391	-1.448811412
H	9.926530838	1.467624426	-1.780812740
H	3.235126257	1.062726259	2.908226490
C	2.266740322	2.792062283	0.984488547
H	2.526512861	3.092426777	2.015993118
C	3.394479752	1.975856423	0.368564546
H	3.195273399	1.927416205	-0.704037845
H	4.342449665	2.518229246	0.513203382
O	2.208264112	3.930827856	0.175470725
C	1.290353894	4.913889885	0.595009446
H	0.265389472	4.518823147	0.570116699
H	1.517713189	5.205905437	1.635458350
C	1.426346064	6.092701435	-0.330472261
H	1.204302907	5.789309502	-1.358913779
H	2.444530010	6.495970249	-0.301577002
H	0.724623144	6.884202480	-0.046310768

==============================

3b

==============================

C	-3.539353848	2.992577314	-1.828609586
C	-3.976193666	2.044603825	-0.917891085
N	-4.771069050	1.033767939	-1.232128382
C	-5.164034367	0.940041482	-2.493110418
C	-4.787278175	1.833402276	-3.480986357
C	-3.952145815	2.888432741	-3.146683931
H	-2.890828133	3.809641838	-1.525653839
H	-3.666532040	2.105935335	0.124526724
H	-5.822110653	0.105395757	-2.730958223
H	-5.155098915	1.709790230	-4.497556210
C	-4.205793381	5.947812080	-3.388922215
O	-3.119399309	5.094345093	-3.630815268
C	-3.458648920	3.868364811	-4.182053089
H	-4.910850525	5.479244709	-2.682472229
H	-4.749634743	6.123614788	-4.332135677
C	-3.671653509	7.240822315	-2.835027695
H	-2.962153912	7.687325478	-3.538681030
H	-3.148217440	7.065677643	-1.889285922
H	-4.486989975	7.949965954	-2.655349493
N	-1.802796006	4.123228550	-5.973890305
C	-2.499491453	3.891173363	-7.219873428
H	-3.577260256	3.939373016	-7.039152622
H	-2.243573666	4.676753044	-7.932709217

H	-2.260643244	2.905072212	-7.651518822
S	-0.225586921	4.582641125	-6.087316036
C	-2.204255104	3.313869476	-4.846051216
C	0.639560163	3.160199881	-6.681302547
O	-0.171233311	5.574965000	-7.124568462
O	0.233916238	4.836771488	-4.751911640
C	0.775352776	2.963005304	-8.047462463
C	1.408224940	1.823401809	-8.504129410
H	0.412917465	3.720926046	-8.735873222
C	1.138049603	2.237755537	-5.777917862
C	1.769404650	1.101109147	-6.252615929
H	1.057843447	2.431117058	-4.712125301
C	1.909648180	0.874744713	-7.616415501
H	1.527693033	1.669183850	-9.574462891
C	2.596770287	-0.352381587	-8.130747795
H	2.172045708	0.378508508	-5.546821594
H	1.917788863	-0.953402817	-8.747434616
H	3.456807375	-0.087047890	-8.756743431
H	2.956095219	-0.982326210	-7.311908722
H	-4.249557495	4.001049995	-4.941689014
H	-1.413183570	3.334741354	-4.090424538
H	-2.372524738	2.269786835	-5.153207302

==============================

p-B-TS'

==============================

C	-3.026009321	2.581121206	-1.940585136
C	-3.524307251	2.012685776	-0.809343874
N	-4.857329845	1.937231421	-0.611641407
C	-5.735321522	2.355267286	-1.556369781
C	-5.284668922	2.925531864	-2.705553770
C	-3.903403044	3.112691641	-2.903265715
N	-5.289813042	1.276302814	0.533192277
C	-6.154055595	2.083507538	1.382880688
C	-5.907347679	-1.126338363	1.605596662
C	-7.101316452	-1.349270105	2.274803877
C	-7.072850704	-2.067359447	3.454181671
C	-5.876452923	-2.565486670	3.963645458
C	-4.694902420	-2.327298164	3.262143612
C	-4.699223995	-1.615001082	2.081513882
C	-5.865934372	-3.378458023	5.217490196
S	-5.938643932	-0.268426389	0.089837439
O	-7.302293777	-0.050897196	-0.313244760
O	-4.960875988	-0.772726417	-0.827016890
H	-1.952215791	2.597449303	-2.091672897
H	-2.921498775	1.573528528	-0.023857364
H	-6.783221245	2.157572985	-1.354743600
H	-6.003592014	3.211951733	-3.465319633
H	-3.527498960	3.386762381	-3.883032560
H	-5.611438751	2.987271786	1.670643568
H	-7.106475830	2.343828440	0.904345214
H	-6.361462116	1.506997824	2.287693501
H	-8.034235001	-0.979565144	1.858903766
H	-8.001013756	-2.255445480	3.987634182
H	-3.757676363	-2.720865965	3.646650791
H	-3.783102036	-1.457168579	1.520095348
H	-6.644102573	-3.051172733	5.913465977
H	-6.056055546	-4.433733463	4.983385563
H	-4.898748398	-3.322307110	5.725175381
C	-1.201448202	5.735077858	-2.653346539
O	-2.383049726	5.574587822	-3.431465626
C	-3.532216549	5.485893250	-2.790394783
H	-1.187945127	4.971944809	-1.862229824
H	-1.237062931	6.722907066	-2.176853895
C	-0.013768650	5.608610630	-3.563432693
H	-0.040165618	6.381654263	-4.337810516
H	0.004833304	4.629423618	-4.052722931
H	0.911871016	5.728818893	-2.991534233
N	-4.627578259	7.301475525	-3.952727795
C	-5.066668987	8.286231041	-2.985956669
H	-4.619733810	8.054641724	-2.013801813
H	-4.714317799	9.279615402	-3.278476477
H	-6.160102844	8.317077637	-2.891818285
S	-4.855992794	7.705356598	-5.551644802
C	-4.724524021	5.907166958	-3.581666946
C	-3.512209654	8.795929909	-5.860893726
O	-4.660322666	6.479950428	-6.282122612
O	-6.074099064	8.456715584	-5.679739475
C	-2.220631361	8.287796021	-5.848621368
C	-1.168055415	9.140915871	-6.097989559
H	-2.058102608	7.231108665	-5.651839256
C	-3.760151863	10.128797531	-6.122373104
C	-2.687765360	10.972249031	-6.369011879
H	-4.784126282	10.490493774	-6.142795563
C	-1.384541512	10.495526314	-6.359984398
H	-0.150321901	8.755734444	-6.102066994
C	-0.228030831	11.400194168	-6.647982121
H	-2.871865511	12.022822380	-6.578265667
H	0.565059602	11.279221535	-5.901717186
H	0.207751080	11.169265747	-7.627752781
H	-0.532356143	12.450351715	-6.656649590
H	-3.503441811	5.601572990	-1.703821778
H	-4.768111229	5.323276997	-4.509015083
H	-5.640934467	5.716640949	-3.004473448

==============================

o-B-TS

==============================

C	2.418424606	-1.167401671	-1.787084103
H	3.214156628	-1.917651296	-1.867540240
H	1.515151739	-1.576123357	-2.248298883
N	2.087840557	-0.818869233	-0.414661258
S	1.550105572	-2.160356283	0.548598826
O	1.629091620	-1.650239706	1.883787394
O	2.309754372	-3.304831266	0.127981514
C	-0.107149668	-2.324923754	0.030507872
C	-1.046828628	-1.417287230	0.498686284
C	-0.454686970	-3.378005743	-0.800916195
C	-2.360335827	-1.571248770	0.107006468
H	-0.776424587	-0.611212015	1.175048113
C	-1.777806878	-3.512809992	-1.176865935
H	0.301352948	-4.087964058	-1.124344707
C	-2.746050119	-2.618182898	-0.729549885
H	-3.103340149	-0.869370639	0.477836311
H	-2.068368673	-4.337898731	-1.821932197
C	-4.181908131	-2.803495169	-1.102253556
H	-4.285258293	-3.296385288	-2.073462248
H	-4.685899734	-3.432895422	-0.357750595
H	-4.714051723	-1.848141909	-1.137064576
N	3.057544708	-0.098153070	0.266289920
C	4.332500935	-0.538463175	0.361948013
C	2.615856647	1.013884068	0.914975762
C	5.224156857	0.122362480	1.148742795
H	4.572125435	-1.440979600	-0.189415127
C	3.488468409	1.642707825	1.815414548
H	1.540082455	1.084343791	1.004039645
C	4.784603119	1.224432349	1.908491254
H	6.240988731	-0.245972648	1.222665429
H	3.085649729	2.446267843	2.423328400
H	5.468703747	1.710927725	2.597274542
H	2.697150230	-0.258516252	-2.327004910
C	-0.878885388	2.427846670	-1.003954172
H	-1.591248512	1.627318382	-0.796915591
H	-1.417489290	3.361009359	-1.228006840
N	0.024815114	2.577057123	0.123137571
S	-0.658164501	2.625577450	1.637353301
O	0.470142573	2.648217916	2.534582376
O	-1.610983849	1.549059153	1.647351384
C	-1.521846533	4.145416737	1.781884789
C	-0.871454239	5.252372265	2.306243896
C	-2.834420919	4.225347519	1.339702725
C	-1.546300650	6.456087112	2.369914055
H	0.139245018	5.157416344	2.691713572
C	-3.490578890	5.438770771	1.410609007
H	-3.340573788	3.336699486	0.974288225
C	-2.859350204	6.569784164	1.921647072
H	-1.047998667	7.326798916	2.788589001
H	-4.521413803	5.509591579	1.072782874
C	-3.592825413	7.868426800	2.029922962
H	-4.344228745	7.970990181	1.241252184
H	-4.115285397	7.930436611	2.992973566
H	-2.909561872	8.720840454	1.970816970
H	-0.296441972	2.141546249	-1.884504557
C	2.330797672	2.585061312	-0.645887196
H	2.110611916	1.841374040	-1.420273542
C	1.196954370	3.394529581	-0.099777974

H	1.526454091	3.863288403	0.832745552
H	0.975725591	4.212721348	-0.806373358
O	3.435628653	3.291572094	-0.784226775
C	4.483626842	2.736650944	-1.569920063
H	4.870288849	1.843497276	-1.062412858
H	4.069431305	2.437122107	-2.543313503
C	5.554793358	3.777188301	-1.727121115
H	5.948516846	4.075477123	-0.750610709
H	5.160659790	4.666001797	-2.228919506
H	6.380043030	3.379427433	-2.326334238
