# Supplementary material for: Visible light induced alkene aminopyridylation using N-aminopyridinium salts as bifunctional reagents
Source: Nat Commun. 2019 Sep 11;10:4117. doi: 10.1038/s41467-019-12216-3 (PMC6739411; doi:10.1038/s41467-019-12216-3)
Supplement: Supplementary file 5 — Supplementary Data 3 [file 41467_2019_12216_MOESM5_ESM.pdf]

Supplementary Data 3. Vibrational frequencies (in cm^{-1}) of the optimized structures

==============================

2a

==============================

30.50	41.63	55.15	59.20	99.76	166.60
186.22	207.67	238.72	241.29	288.54	296.49
325.92	352.32	386.40	422.95	425.16	434.81
470.52	515.33	524.05	564.47	593.36	604.42
608.15	657.68	666.13	690.83	710.02	723.37
746.36	824.16	845.39	860.35	862.27	880.82
895.03	1008.51	1012.36	1031.67	1039.71	1054.78
1076.17	1077.99	1100.08	1123.89	1125.73	1133.15
1140.36	1166.85	1168.82	1171.28	1203.97	1208.27
1226.69	1235.96	1243.17	1271.15	1293.89	1351.17
1362.15	1381.03	1395.29	1418.21	1465.92	1476.87
1493.77	1497.74	1515.30	1523.70	1534.94	1547.57
1562.60	1563.30	1680.07	1687.90	1702.43	1734.77
2642.01	2680.03	2775.84	3061.31	3151.94	3178.64
3209.03	3215.00	3230.15	3231.52	3232.42	3247.64
3251.70	3259.03	3274.69			

==============================

1b

==============================

107.30	194.92	237.81	309.26	457.70	525.54
751.76	850.50	899.50	922.86	1023.31	1047.44
1132.17	1207.58	1211.95	1277.96	1332.91	1365.77
1415.88	1447.34	1473.17	1490.24	1515.80	1544.48
1757.65	2980.08	3016.66	3024.21	3033.79	3078.33
3107.20	3122.03	3232.65			

==============================

PO_4^{3-}

==============================

402.52	403.33	568.19	568.30	568.52	887.03
1014.13	1015.60	1016.37			

==============================

HPO_4^{2-}

==============================

283.92	355.92	371.66	517.13	532.91	548.65
701.26	988.37	1052.40	1201.26	1217.42	3934.20

==============================

Pyridine

==============================

401.11	433.40	627.63	685.14	736.69	790.30
923.14	991.64	1042.85	1045.13	1049.57	1077.10
1105.07	1119.25	1178.89	1266.62	1321.56	1400.41
1510.08	1551.48	1695.56	1701.39	3181.65	3184.04
3204.69	3223.28	3230.40			

==============================

2a

==============================

30.50	41.63	55.15	59.20	99.76	166.60
186.22	207.67	238.72	241.29	288.54	296.49
325.92	352.32	386.40	422.95	425.16	434.81
470.52	515.33	524.05	564.47	593.36	604.42
608.15	657.68	666.13	690.83	710.02	723.37
746.36	824.16	845.39	860.35	862.27	880.82
895.03	1008.51	1012.36	1031.67	1039.71	1054.78
1076.17	1077.99	1100.08	1123.89	1125.73	1133.15
1140.36	1166.85	1168.82	1171.28	1203.97	1208.27
1226.69	1235.96	1243.17	1271.15	1293.89	1351.17
1362.15	1381.03	1395.29	1418.21	1465.92	1476.87
1493.77	1497.74	1515.30	1523.70	1534.94	1547.57
1562.60	1563.30	1680.07	1687.90	1702.43	1734.77
2642.01	2680.03	2775.84	3061.31	3151.94	3178.64
3209.03	3215.00	3230.15	3231.52	3232.42	3247.64
3251.70	3259.03	3274.69			

==============================

2a*

==============================

19.30	29.01	40.79	59.44	88.98	162.92
173.11	193.73	201.58	221.62	257.51	286.28
306.19	344.81	366.69	388.00	427.47	444.89
458.47	499.67	514.33	533.02	583.72	592.53
604.02	629.43	644.18	657.10	661.40	683.39
717.47	720.06	742.21	756.31	834.01	851.95
862.20	882.37	955.94	962.96	1004.15	1012.94
1027.48	1051.70	1059.21	1093.32	1109.93	1115.24
1122.68	1140.62	1159.66	1167.37	1171.39	1202.93
1219.58	1223.87	1237.44	1268.31	1334.64	1346.65
1356.39	1369.50	1377.41	1399.55	1407.98	1465.69
1473.24	1492.66	1492.73	1501.38	1509.46	1525.92
1534.58	1569.90	1583.53	1687.37	1712.41	1717.70
2637.61	2675.21	2753.85	3058.58	3141.16	3170.57
3197.96	3204.75	3213.73	3216.91	3227.86	3229.22
3247.49	3255.78	3266.83			

==============================

2a*-TS

==============================

-766.60	30.58	37.68	55.88	62.21	85.21
166.79	174.85	191.75	218.03	226.81	256.65
287.19	303.23	347.88	374.07	401.29	422.82
428.38	452.97	502.23	525.17	557.78	582.89
593.72	603.07	661.36	661.77	679.02	689.87
732.09	755.78	762.58	844.33	852.83	859.63
880.36	901.40	939.49	986.43	1000.67	1002.71
1024.05	1058.58	1059.30	1079.44	1113.87	1121.34
1128.09	1135.60	1158.16	1168.10	1170.89	1174.73
1192.12	1209.57	1221.61	1226.49	1267.84	1335.36
1344.78	1365.29	1381.31	1384.66	1453.11	1472.43
1491.68	1493.26	1495.11	1502.55	1512.28	1526.89
1533.59	1568.57	1611.25	1652.39	1685.80	1711.96
2634.51	2678.47	2752.03	3037.79	3104.78	3158.23
3196.22	3201.61	3212.87	3218.63	3229.51	3229.69
3230.59	3236.43	3242.46			

==============================

A

==============================

38.53	62.16	109.37	164.53	183.33	222.39
293.98	294.85	337.77	379.59	407.32	426.73
481.17	506.82	551.26	585.63	590.57	660.19
684.25	732.63	755.90	846.40	862.36	879.87
1005.73	1010.87	1025.12	1058.92	1110.77	1120.53
1141.30	1159.52	1164.99	1167.55	1206.16	1226.74
1268.60	1346.92	1369.38	1395.81	1422.79	1459.95
1473.88	1490.38	1491.58	1525.07	1533.37	1568.41
1683.91	1710.80	2655.27	2694.61	2772.73	2951.29
3052.94	3150.12	3197.15	3205.63	3230.44	3231.33

==============================

A-TS

==============================

-173.78	25.88	29.58	32.70	47.29	54.12
67.97	101.87	129.76	143.66	169.02	180.28
202.60	226.14	259.14	266.74	270.53	289.43
310.07	329.96	365.61	399.38	428.44	449.22
487.45	511.85	518.95	556.18	582.42	661.71
676.11	738.90	761.00	794.09	836.38	845.11
848.98	878.18	885.06	929.49	988.99	998.78
1020.20	1021.95	1022.48	1060.12	1080.99	1082.01
1128.95	1133.49	1142.30	1148.34	1177.70	1182.09
1192.78	1206.13	1220.94	1268.23	1290.24	1334.51
1341.34	1343.97	1357.97	1371.85	1411.62	1428.87
1438.26	1448.86	1465.65	1468.65	1483.76	1489.55
1492.98	1498.21	1508.02	1512.97	1541.19	1569.44
1676.31	1686.11	1713.88	3014.49	3044.03	3055.43
3063.49	3083.25	3108.90	3117.23	3140.86	3143.37
3144.93	3145.97	3185.03	3197.38	3200.19	3201.96
3230.52	3231.60	3310.23			

==============================

B

==============================

17.48	34.53	38.94	50.28	56.06	70.54
83.90	135.84	147.24	170.46	184.66	191.47
237.20	251.83	265.79	290.32	310.65	333.06
343.70	385.92	406.49	430.44	442.95	482.30
506.37	537.38	561.00	574.64	642.17	662.59

687.88	744.86	793.10	831.10	847.69	854.22
882.27	887.74	963.93	1003.30	1019.74	1021.07
1027.20	1059.28	1080.73	1105.35	1141.73	1144.83
1150.22	1168.80	1175.53	1203.69	1206.26	1223.12
1231.61	1268.11	1269.82	1307.54	1327.18	1337.18
1344.50	1361.25	1398.12	1409.68	1419.55	1428.65
1446.63	1463.88	1471.30	1483.26	1489.38	1497.29
1501.72	1504.89	1510.56	1512.30	1519.72	1540.58
1570.29	1684.34	1715.18	3009.42	3015.52	3026.59
3054.75	3064.30	3068.03	3119.83	3140.30	3140.94
3143.50	3144.93	3146.03	3155.33	3187.94	3199.45
3199.93	3229.80	3230.69			

==============================

p-B-TS

==============================

-245.98	16.80	18.67	24.67	28.35	31.74
36.90	37.40	41.54	45.21	49.23	52.60
58.79	72.76	78.13	85.85	95.29	118.54
129.36	142.90	155.73	171.59	175.52	178.84
188.36	206.10	231.69	238.81	249.74	263.58
278.84	282.81	287.65	296.99	313.93	316.15
328.33	338.55	343.96	355.22	360.39	378.96
407.15	418.08	426.87	427.80	432.15	446.30
467.57	488.30	501.71	513.46	523.34	547.85
553.99	567.69	589.20	595.95	604.99	659.29
660.46	662.73	682.23	684.80	721.92	726.40
739.99	740.82	780.43	816.85	823.26	835.73
842.69	847.17	848.51	852.04	854.41	865.67
879.98	882.08	929.73	945.96	996.45	1003.98
1004.52	1009.75	1015.02	1023.90	1025.11	1030.73
1030.94	1032.28	1056.34	1056.71	1064.57	1080.81
1081.04	1087.90	1104.45	1118.15	1123.50	1131.17
1136.05	1136.77	1155.55	1156.60	1161.67	1168.88
1177.72	1184.20	1195.65	1197.80	1207.40	1226.89
1228.55	1229.97	1241.32	1245.16	1271.40	1271.91
1295.10	1314.77	1319.88	1331.99	1341.93	1347.59
1348.39	1363.25	1370.46	1372.25	1375.23	1385.09
1388.70	1411.16	1416.41	1430.39	1430.82	1446.92
1464.51	1466.24	1470.89	1473.75	1486.14	1490.24
1497.44	1497.66	1501.56	1503.24	1504.44	1505.87
1508.01	1509.61	1516.47	1517.12	1534.15	1537.65
1563.06	1566.89	1567.56	1616.24	1681.76	1683.83
1708.07	1709.13	1728.10	2974.34	3034.40	3041.29
3059.52	3060.03	3070.08	3072.64	3087.83	3118.81
3126.61	3127.42	3127.71	3133.68	3153.36	3154.31
3155.00	3157.26	3161.07	3171.55	3198.78	3207.06
3208.02	3209.51	3212.11	3219.53	3228.17	3231.09
3232.29	3233.54	3243.81	3247.60	3263.77	3278.36

==============================

p-C

==============================

16.31	19.27	23.61	28.52	30.86	34.74
35.96	39.80	50.30	54.50	57.66	58.79
71.43	76.70	82.70	104.75	125.84	145.66
151.61	156.32	173.03	174.09	197.87	199.72
210.45	232.59	249.12	253.55	273.25	276.25
284.72	288.95	301.71	311.69	322.91	335.20
341.10	346.39	356.84	377.97	398.35	410.22
424.21	427.05	430.42	436.30	454.59	480.48
490.56	493.15	504.88	523.20	541.20	551.13
567.75	571.80	597.63	603.68	646.98	656.82
660.59	661.43	681.84	711.84	714.03	736.42
739.47	764.86	801.92	816.51	834.10	845.04
847.05	851.12	853.21	854.62	870.77	881.84
883.89	924.24	964.63	988.80	991.56	1005.56
1006.74	1017.84	1021.99	1022.63	1025.24	1028.92
1032.47	1058.05	1058.93	1064.03	1076.28	1082.17
1082.41	1098.66	1114.36	1118.55	1134.65	1141.54
1153.59	1154.56	1160.56	1163.10	1166.82	1170.71
1182.31	1199.04	1203.06	1208.23	1215.70	1227.16
1234.81	1235.28	1236.48	1253.14	1258.30	1270.56
1272.79	1306.22	1307.64	1311.65	1326.92	1347.09
1351.74	1351.96	1364.71	1374.36	1382.89	1397.30
1414.36	1417.66	1419.45	1431.25	1431.60	1431.78
1440.36	1464.43	1466.13	1467.88	1469.75	1470.46
1484.67	1489.63	1490.79	1491.27	1492.54	1503.73
1507.45	1508.53	1508.82	1512.02	1513.99	1518.87
1542.13	1564.93	1568.55	1587.82	1680.33	1682.41

1704.28	1711.57	1722.57	2934.52	3003.46	3015.50
3021.84	3035.84	3061.20	3064.19	3064.49	3064.80
3108.29	3114.35	3125.01	3128.30	3128.31	3142.88
3152.25	3153.80	3159.15	3162.09	3177.26	3181.08
3203.30	3207.71	3213.80	3219.47	3228.14	3229.44
3235.84	3236.77	3237.76	3252.31	3260.69	3274.22

==============================

D

==============================

12.48	14.28	18.29	26.06	26.98	37.26
39.74	41.04	50.44	58.22	60.02	66.99
72.43	84.54	95.01	126.58	137.40	149.88
157.93	163.80	167.70	177.35	191.10	204.47
209.05	222.44	240.91	259.97	271.65	281.65
284.43	289.82	314.85	318.90	334.52	353.06
357.83	362.97	376.78	389.99	392.42	412.60
428.19	429.33	444.62	458.90	483.53	501.39
510.70	514.04	531.57	533.46	545.37	575.60
582.47	583.54	591.79	614.14	661.62	661.98
668.48	671.02	680.06	691.86	715.42	718.65
739.78	755.68	761.22	784.17	827.90	831.97
844.73	850.62	853.54	854.75	861.70	882.43
883.60	893.02	942.89	967.89	968.89	1003.29
1004.51	1020.66	1021.94	1024.86	1027.38	1027.47
1058.89	1063.88	1080.11	1097.63	1108.90	1115.87
1118.00	1121.78	1139.91	1142.28	1154.38	1155.20
1158.68	1164.34	1167.60	1169.45	1189.34	1202.58
1204.83	1207.30	1215.31	1224.64	1227.16	1231.00
1240.46	1267.20	1269.00	1269.30	1282.37	1316.74
1325.82	1341.65	1346.94	1353.08	1355.09	1366.12
1368.79	1371.21	1376.95	1392.41	1392.76	1406.45
1407.57	1421.52	1428.56	1436.89	1465.83	1468.02
1468.12	1473.32	1475.53	1488.18	1488.41	1491.22
1493.00	1499.24	1500.42	1507.44	1507.65	1508.39
1516.15	1517.46	1525.70	1531.78	1539.92	1567.34
1569.66	1576.18	1686.62	1688.76	1712.30	1717.10
1726.07	2671.47	2718.80	2788.37	2985.85	2997.14
3045.02	3048.49	3054.66	3057.11	3057.55	3060.74
3115.43	3120.97	3134.84	3139.59	3142.64	3143.85
3145.88	3149.57	3169.61	3186.15	3198.46	3201.07
3202.94	3204.30	3211.57	3220.91	3227.13	3229.56
3232.24	3254.39	3264.49			

==============================

D-TS

==============================

-741.03	14.59	14.93	18.84	20.54	26.47
29.50	39.75	41.29	44.92	48.65	56.13
57.05	71.83	78.88	92.46	103.13	135.79
143.98	148.46	157.21	171.60	181.15	182.29
195.92	209.15	224.31	238.17	257.14	265.26
270.84	284.64	290.47	297.86	309.76	318.43
323.32	333.65	340.18	363.12	373.14	386.66
398.82	411.82	412.84	428.91	429.82	472.47
480.54	491.57	503.40	519.68	543.80	559.57
570.68	579.30	586.57	612.70	625.67	661.68
661.90	675.23	678.56	683.10	721.99	744.01
744.71	762.12	788.84	799.84	835.30	844.29
846.27	848.24	849.62	852.63	854.42	878.96
882.11	934.55	944.57	965.83	988.23	998.81
1002.97	1006.03	1020.94	1021.13	1025.01	1026.54
1030.33	1058.92	1060.25	1073.41	1080.23	1081.61
1094.32	1120.79	1126.13	1134.78	1142.65	1146.68
1148.79	1150.79	1165.81	1171.11	1173.72	1190.00
1203.31	1207.45	1208.95	1217.51	1220.90	1222.87
1224.58	1237.88	1269.21	1269.29	1279.18	1305.98
1312.01	1337.43	1343.48	1345.34	1351.99	1361.32
1361.69	1365.73	1366.72	1376.24	1406.83	1412.62
1426.67	1428.46	1429.23	1435.99	1453.36	1464.62
1464.77	1470.30	1474.66	1486.29	1488.28	1493.96
1494.16	1497.05	1505.28	1506.25	1506.89	1507.29
1510.62	1512.35	1516.80	1528.66	1552.14	1569.26
1570.28	1598.41	1669.25	1684.51	1686.30	1713.42
1715.12	2986.18	2992.97	3010.39	3029.82	3038.15
3054.34	3056.62	3059.94	3082.12	3104.47	3119.13
3119.23	3122.20	3138.64	3142.08	3145.45	3146.36
3160.55	3160.98	3180.14	3197.61	3197.69	3199.44
3201.20	3202.92	3221.58	3223.93	3229.54	3229.61
3230.35	3231.44	3239.41			

==============================

3b

==============================

8.24	11.57	14.51	27.13	40.51	45.21
55.42	63.18	78.35	87.13	139.99	163.97
172.48	177.65	185.49	235.75	242.39	268.97
275.73	299.25	309.24	333.94	344.70	367.58
392.64	406.87	409.22	429.53	480.86	496.27
521.33	525.93	568.23	585.39	603.29	657.99
662.64	685.39	699.65	743.05	775.70	798.86
834.20	847.40	852.72	857.45	871.18	882.10
914.33	927.58	972.52	1002.31	1017.54	1020.92
1025.39	1028.08	1045.30	1048.06	1060.46	1081.39
1104.44	1113.09	1123.43	1131.11	1144.73	1150.77
1164.64	1177.48	1206.26	1208.03	1224.15	1229.27
1243.35	1262.68	1269.45	1277.95	1305.33	1314.75
1328.18	1333.53	1344.82	1361.84	1366.61	1378.31
1405.71	1413.32	1422.61	1429.34	1436.69	1464.90
1467.56	1469.92	1482.79	1484.82	1487.87	1493.08
1507.61	1509.80	1510.55	1517.14	1541.78	1569.94
1570.55	1684.77	1686.99	1714.91	1715.40	2994.87
3002.97	3009.29	3032.20	3033.17	3056.34	3063.81
3118.08	3120.59	3141.17	3142.03	3145.80	3148.18
3177.38	3179.94	3184.43	3196.54	3201.88	3202.68
3227.01	3229.13	3229.93			

==============================

p-B-TS'

==============================

-103.59	12.24	15.39	22.88	23.05	29.19
33.14	35.82	37.44	45.58	47.93	53.45
57.34	62.56	67.73	76.45	88.21	106.85
130.15	138.91	152.94	166.49	172.79	177.62
185.33	187.64	209.89	235.36	240.30	259.98
272.42	284.41	287.67	299.44	304.56	308.17
321.52	335.60	342.77	360.84	364.95	370.69
407.22	418.95	428.21	431.02	447.77	450.23
465.56	488.29	501.94	509.34	519.80	530.84
555.70	569.65	589.67	596.78	606.42	658.52
660.83	663.83	681.89	686.96	720.59	724.31
733.29	738.64	747.54	804.10	823.11	826.57
842.37	844.76	848.71	853.54	859.66	872.07
880.46	881.29	881.61	971.47	983.02	996.76
1005.82	1005.88	1020.97	1022.31	1025.75	1025.96
1027.62	1033.10	1055.87	1059.39	1060.36	1081.02
1081.20	1085.92	1104.48	1119.35	1122.26	1129.01
1135.82	1140.93	1152.42	1155.28	1159.52	1166.63
1171.09	1192.46	1194.46	1202.50	1208.30	1225.65
1228.49	1234.71	1237.09	1242.99	1269.62	1272.24
1303.22	1306.39	1314.42	1331.00	1348.71	1350.59
1368.15	1372.96	1378.71	1381.59	1391.40	1395.76
1400.72	1416.11	1422.34	1430.30	1430.53	1442.11
1466.37	1466.48	1467.89	1477.56	1485.02	1489.56
1493.25	1497.80	1499.23	1499.49	1501.64	1504.25
1506.15	1507.98	1511.58	1513.12	1533.58	1536.61
1565.63	1571.32	1572.17	1620.61	1681.42	1687.29
1705.48	1713.26	1723.15	3041.51	3044.26	3047.50
3059.90	3060.64	3061.64	3069.90	3094.42	3107.66
3122.86	3124.58	3129.93	3142.15	3150.31	3150.47
3151.39	3152.73	3153.80	3156.55	3177.26	3193.36
3210.13	3212.86	3213.97	3220.14	3233.23	3234.27
3235.67	3236.95	3242.46	3246.63	3255.77	3274.83

==============================

o-B-TS

==============================

-215.96	18.33	19.93	22.56	27.25	33.05
36.43	46.07	48.49	49.37	53.98	59.86
64.08	71.36	77.09	92.91	100.86	108.14
119.51	138.98	150.56	170.04	176.21	177.52
186.36	208.09	215.21	224.62	247.48	263.68
269.59	282.46	288.04	296.04	314.56	315.67
321.80	336.67	341.58	360.22	372.44	377.35
407.41	424.93	427.77	429.86	434.07	448.95
464.52	485.31	503.93	508.22	519.39	544.06
550.81	569.41	591.47	594.53	599.79	658.16
660.15	661.03	680.77	682.78	712.15	722.27
736.92	739.62	770.10	795.96	827.04	838.88
844.18	847.02	853.59	854.58	861.60	877.60
880.02	887.44	925.22	954.00	998.69	1003.53

**1008.04**	**1014.03**	**1023.30**	**1024.47**	**1029.98**	**1032.39**
**1038.48**	**1044.99**	**1051.68**	**1056.58**	**1057.15**	**1079.74**
**1081.08**	**1084.18**	**1107.28**	**1113.91**	**1122.72**	**1132.63**
**1134.50**	**1137.08**	**1156.09**	**1159.05**	**1162.95**	**1166.73**
**1171.63**	**1194.90**	**1199.45**	**1200.89**	**1208.48**	**1219.83**
**1223.35**	**1227.61**	**1232.61**	**1233.97**	**1271.90**	**1272.16**
**1292.00**	**1311.68**	**1317.56**	**1332.61**	**1345.37**	**1346.64**
**1348.04**	**1365.84**	**1372.03**	**1372.18**	**1380.85**	**1388.62**
**1397.26**	**1415.54**	**1422.89**	**1429.86**	**1430.94**	**1445.08**
**1463.78**	**1464.92**	**1465.86**	**1467.56**	**1481.09**	**1489.74**
**1496.09**	**1497.05**	**1497.83**	**1500.61**	**1503.09**	**1503.65**
**1505.68**	**1509.17**	**1517.13**	**1518.87**	**1526.14**	**1535.99**
**1538.57**	**1563.66**	**1567.26**	**1642.17**	**1681.49**	**1681.61**
**1704.77**	**1707.66**	**1709.38**	**3009.92**	**3031.73**	**3043.43**
**3059.93**	**3060.24**	**3061.85**	**3072.03**	**3095.44**	**3106.70**
**3126.92**	**3127.33**	**3127.53**	**3131.06**	**3144.55**	**3153.87**
**3154.06**	**3154.21**	**3156.85**	**3167.46**	**3179.01**	**3208.80**
**3209.22**	**3210.90**	**3211.03**	**3229.12**	**3231.39**	**3232.20**
**3233.02**	**3233.92**	**3246.79**	**3249.45**	**3262.20**	**3274.96**
